# Leveraging the global genomic epidemiology of carbapenemase-producing *Klebsiella pneumoniae* to inform infection prevention in Tunisian hospitals

**DOI:** 10.1128/aac.00142-26

**Published:** 2026-05-06

**Authors:** Basma Menif, Jay N. Worley, Noura Ben Mansour, Faouzia Mahjoubi, Adnene Hammami, Lynn Bry

**Affiliations:** 1 Laboratory of Microbiology, Habib Bourguiba University Hospital, Sfax University, Sfax, Tunisia; 2 School of Medicine of Sfax, Sfax University, Sfax, Tunisia; 3 Massachusetts Host-Microbiome Center, Department of Pathology, Brigham and Women’s Hospital, Harvard Medical School 207095 , Boston, Massachusetts, USA; 4 Harvard Medical School 1811 , Boston, Massachusetts, USA; 5 National Center for Biotechnology Information, National Library of Medicine, National Institutes of Health 2511, Bethesda, Maryland, USA; 6 Clinical Microbiology Laboratory, Department of Pathology, Brigham and Women’s Hospital, Harvard Medical School 207095 , Boston, Massachusetts, USA; Universita degli Studi di Roma La Sapienza, Rome, Italy

**Keywords:** Tunisia, carbapenemase-producing *K. pneumoniae*, genomic epidemiology

## Abstract

Carbapenemase-producing *Klebsiella pneumoniae* (CPK) is a major threat in North Africa, yet long-term genomic surveillance efforts remain scarce. To address this, we analyzed 1,013 CPK clinical isolates collected at Habib Bourguiba Hospital (Sfax, Tunisia) from 2009 to 2022. Given limited genomic resources, clinical microbiologic and pulse-field gel electrophoresis (PFGE) profiles identified representative isolates for genomic analyses to resolve transmission dynamics and plasmid dissemination over time. Hospital-acquired CPK increased >10-fold, from 0.95 to 9.59 per 10,000 patient-days. OXA-48-like enzymes predominated, followed by NDMs and dual producers. Genomic analyses identified 23 sequence types, with ST101, ST147, and ST383 accounting for 70% of isolates. ST383 strains carrying *bla*_OXA-204_ on IncC plasmids occurred first, followed by ST101 with *bla*_OXA-48_ on IncL replicons and ST147 with *bla*_NDM-1_ on IncFIB/IncFII multi-replicons. Since 2019, epidemic ST383 co-harboring *bla*_OXA-48_ on IncL plasmids and *bla*_NDM-5_ with *rmpA/iuc* on hybrid IncFIB/IncHI1B plasmids became dominant. Comparison with 80,252 global genomes in NCBI Pathogen Detection showed that 82% of study isolates clustered with Mediterranean/global lineages, demonstrating frequent international dissemination. Findings reveal both local and international reservoirs driving CPK introductions, informing targeted infection-control strategies in regional hospitals.

## INTRODUCTION

Carbapenem resistance is a major global public health problem (1, 2). The World Health Organization (WHO) prioritized carbapenemase-producing *Klebsiella pneumoniae* (CPK) as a top multidrug-resistant pathogen for 2024 (3). Prevalent high-risk clones include clonal groups (CGs) 258 (ST258, ST1512, ST11), CG15 (ST15, ST14), ST101, and ST307 (4), which are frequently encountered in hospital outbreaks (5). These clusters share antimicrobial resistance profiles but differ from hypervirulent *K. pneumoniae* strains (HvKp) associated with community-acquired invasive infections, though some Asian ST11 and ST23 HvKP strains have demonstrated carbapenem resistance (6, 7). These dynamics led to the WHO’s 2024 guidance to implement measures for CPK surveillance to inform local prevention efforts (8).

An ongoing *K. pneumoniae* surveillance program at Habib Bourguiba Hospital (HBH) in Sfax, Tunisia, optimizes use of Clinical Microbiologic testing with pulse field gel electrophoresis (PFGE) to select isolates for whole-genome analyses, followed by genomic-epidemiologic investigation to identify putative transmission chains, reservoirs, and inform the implementation of improved prevention programs. The program’s prior successes include identifying and supporting containment of an outbreak of VIM-4-producing *K. pneumoniae* in 2005 (9) and confirming entry of the first OXA-48-like producing *K. pneumoniae* in 2009 (10). Since then, OXA-48- and NDM-producing CPK have become endemic in Tunisian hospitals (11–15).

Despite prior achievements, the genetic epidemiology of nosocomial CPKs in Tunisia remains limited and hampers efforts to identify reservoirs to inform prevention efforts (11, 13, 14). The Laboratory of Antibiotic Resistance in Tunisia (LART), which includes the 11 major university hospitals in the country (16), reported that carbapenem resistance among *K. pneumoniae* infections increased dramatically from 0.6% of infections in 2010 to 22.4% in 2019, and 18.5% in 2022, rates that are comparable to those seen across Africa and the Middle East (5.7%–26.9%) (17). Of note, HBH contributes more than 20% of all *K. pneumoniae* isolates reported to the LART network. HBH is experiencing sustained and frequent cross-transmission pressures, and systemic detection and description of newly introduced strains is not routinely implemented. Therefore, our aim extends beyond documenting cross-transmission to evaluating clonal and plasmid dynamics, as well as identifying evidence of introductions over time.

Over 14 years, from 2009 to 2022, we evaluated CPK dynamics in HBH. Publicly available genomic analysis tools in FDA GenomeTrakr (18, 19) and NCBI Pathogen Detection (20, 21) enabled high-resolution analyses of strain dynamics and their placement within a global context. Our analyses identified time-dependent emergence of genomic clones and antimicrobial resistance (AMR)-transmitting replicons, with recent and repeated emergence of global ST383 strains. Our findings demonstrate internal and external reservoirs of strains impacting patient infections within HBH, emphasizing the need to expand genomic surveillance efforts in low- and middle-income countries. The findings informed our infection prevention and control efforts and brought clarity to the active CPK crisis affecting a region with limited genomic resources.

## RESULTS

### Demographics and dynamics of patient-infecting CPKs

We evaluated all 1,013 unique patient CPK isolates, accounting for 77% of the 1,318 carbapenemase-producing Enterobacteriaceae (CPE) isolates identified at HBH from 2009 to 2022. Strains originated from diagnostic cultures ordered in the course of clinical care, as surveillance screening for CPE colonization was not in place. Other non-*K*. *pneumoniae* CPE included species of *Enterobacter* (*n* = 66, 5%), *Escherichia coli* (*n* = 56, 4.2%), and *Proteus* (*n* = 157, 11.9%).

**Fig 1:**
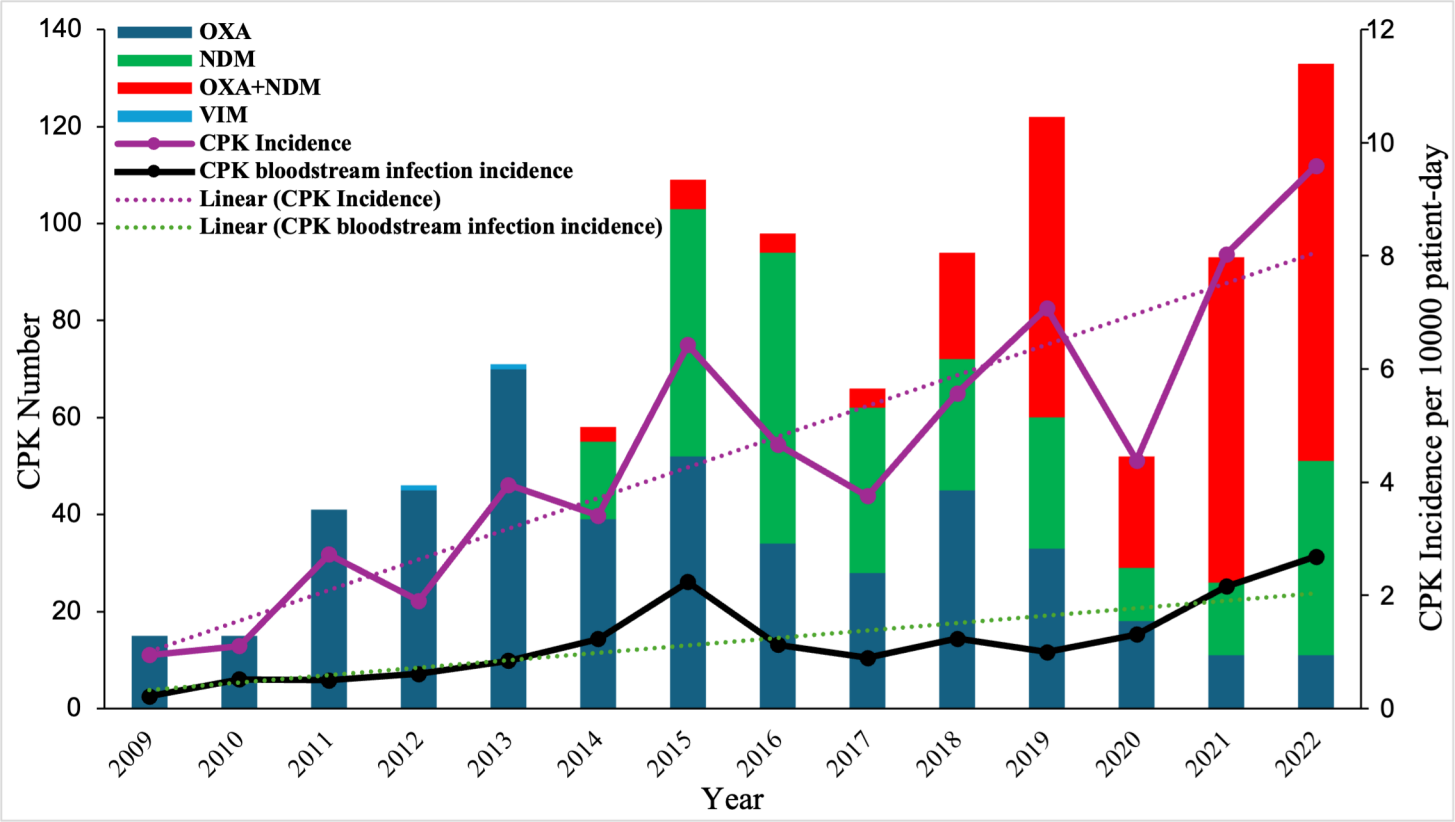
Annual incidence and carbapenemase profiles of CPK isolates in HBH, 2009–2022. The X-axis shows years; the Y-axis on the right shows the number of CPK isolated by year, and on the left shows the incidence of CPK per 10,000 patient-days. Sample key indicates the carbapenemase types and the linear regression lines representing the trend in CPK incidence.

Among CPK isolates and relative to the first positive culture post-admission, 115 isolates (11.3%) were obtained from outpatients, 114 (11.4%) from inpatients within 48 h of admission, and 748 (77.4%) from inpatients after 48 h of hospitalization. The CPK rates yielded an overall hospital-acquired incidence ( >48 h after admission) of 4.365 per 10,000 hospital days (Table 1). However, CPK rates increased >10-fold from 0.950 to 9.590 cases per 10,000 patient-days from 2009 to 2022 (*P* < 0.001, Fig. 1).

**TABLE 1:** Demographics of CPK-infected patients^*a*^

Demographic information	Data
Gender (% of cases)	
Male	658 (64.9)
Female	355 (35.1)
Age: years (standard deviation)	54.21 (18.3)
Hospital ward (% of cases)	
Intensive care	504 (49.8)
Medical	38 (3.8)
Surgical	423 (41.8)
Emergency room	48 (4.7)
Isolate site (% of cases)	
Urine	380 (37.5)
Blood	220 (21.7)
Sputum	152 (15.0)
Skin/soft tissue	198 (19.5)
Other^*b*^	63 (6.2)
Origin (% of cases)	
Hospital-acquired (≥48 h after admission)	784 (77.4)
Non-hospital-acquired (<48 h after admission)	114 (11.3)
Outpatient	115 (11.4)

^*a*^ Demographic data of CPK isolates from patients seen at Habib Bourguiba Hospital in Sfax, Tunisia, over 2009–2022. Numbers represent patients; percentages are in parentheses.

^*b*^ Catheter, drain, biopsy, material, and genital specimens.

CPK infections occurred most frequently in medical intensive care unit (ICU) patients (45.8%), with an average rate of 37.5 infections per 10,000 patient-days (range of 4.7 to 64.5 infections), 2009–2022 (*P* < 0.001). Urinary tract infections (*n* = 380, 37.5%) were most common, followed by bloodstream infections (220, 21.7%), wound infections (198, 19.5%), and pneumonias (152, 15.0%) (Table 1). Clinical PCR testing for carbapenemase carriage identified OXA-48-like enzymes (730, 72.1%) and NDMs (554, 54.7%) as the most prevalent, with 26.9% of CPKs encoding both (*n* = 273). Only two isolates expressed VIM enzymes, and no isolates produced KPCs (22). OXA-48 producers predominated until 2014, while NDM-carrying isolates predominated from 2017 (Fig. 1).

Antimicrobial susceptibility testing showed high levels of carbapenem resistance, with 84.8% of isolates resistant to imipenem (MIC > 2 mg/L) and 83.5% to meropenem (MIC > 2 mg/L). Resistance was lower among OXA-48 producers (71.5% and 66.7%, respectively) but >91% in NDM producers. Overall, 81% of isolates were resistant to both carbapenems, whereas only 13%, predominantly OXA-48 producers, remained susceptible to both. The OXA-48-like producers also remained susceptible to ceftazidime/avibactam, while all CPK isolates remained susceptible to aztreonam-avibactam. The CPKs were frequently resistant to gentamicin (89.5%) and amikacin (54.6%), with methyltransferase-encoding genes detected by PCR *armA*, *rmtF*, *rmtC*, *rmtB*, and *rmtE* detected in 219 (21.6%), 46 (4.5%), 23 (2.3%), 5 (0.5%), and 3 (0.3%) isolates, respectively. In addition, 57.3% of isolates were resistant to tigecycline (MIC > 0.5 mg/L), and 212 (20.9%) to colistin (MIC > 2 mg/L), with no *mcr* genes detected by PCR.

Given concerns for the spread of hypervirulent CPK, string-test and PCR evaluation of putative *K. pneumoniae* virulence loci *rmpA/rmpA2* and aerobactin (*iuc*) were performed. These studies identified 265 (26.2%) convergent CPK isolates that harbored r*mpA/rmpA2* and/or aerobactin. However, only four of them were string-test positive. These string-test-positive isolates belonged to classical hv-KP lineages, including ST23-KL1 in 2013 (*n* = 1), ST86-KL2 in 2010 (*n* = 1), and ST218-KL57 in 2010 (*n* = 2). The remaining 261 convergent string-test-negative strains lacked typical hypervirulent capsular types and, among sequenced strains, all harbored truncated or incomplete *rmpA2* genes. These convergent CPK isolates emerged in 2018 and rapidly expanded, from an initial prevalence of 11% in 2018 to 72% in 2021 and 63.9% in 2022.

### Bloodstream CPK isolate typing

Given limited resources for strain genomic analyses, we subjected 220 CPK bloodstream isolates to PFGE and selected 95 representative isolates for genomic analyses, including one to two isolates per PFGE and AMR profile per year (study flowchart, Fig. S1). The bloodstream isolates exhibited the same resistance and virulence profiles as those from other sites, indicating they are likely representative of the whole set (Table S1). While PFGE identified 2 to 12 pulsotypes per year, a single pulsotype accounted for >30% of bloodstream isolates each year (Fig. S1; Tables S2 and S3). This similarity reduced the number of isolates needed to build a representative set.

Genomic analyses revealed 23 multi-locus sequence types (STs), three of which accounted for 70% of the 220 bloodstream CPK isolates: ST101 (*n* = 73, [33.2%]), ST383 (*n* = 41 [18.6%]), and ST147 (*n* = 41 [18.6%]). Additional clusters ST11 (*n* = 9), ST15 (*n* = 9), ST13 (*n* = 5), ST307 (*n* = 5), and ST2096 (*n* = 5) occurred with less frequency and for shorter durations than the major three.

Carbapenemase gene detection by PCR-sequencing demonstrated that OXA-48 (*n* = 143 [52.6%]) occurred most frequently among bloodstream CPK isolates, followed by NDM-1 (*n* = 71 [26%]), NDM-5 (*n* = 45 [16.5%]), OXA-204 (*n* = 11 [4%]), VIM-1 (*n* = 1), and VIM4 (*n* = 1). In addition, 52 (24%) isolates harbored >1 carbapenemase, with 41 carrying *bla*_OXA-48_ and *bla*_NDM-5_, and 11 carrying *bla*_OXA-48_ and *bla*_NDM-1_.

**Fig 2:**
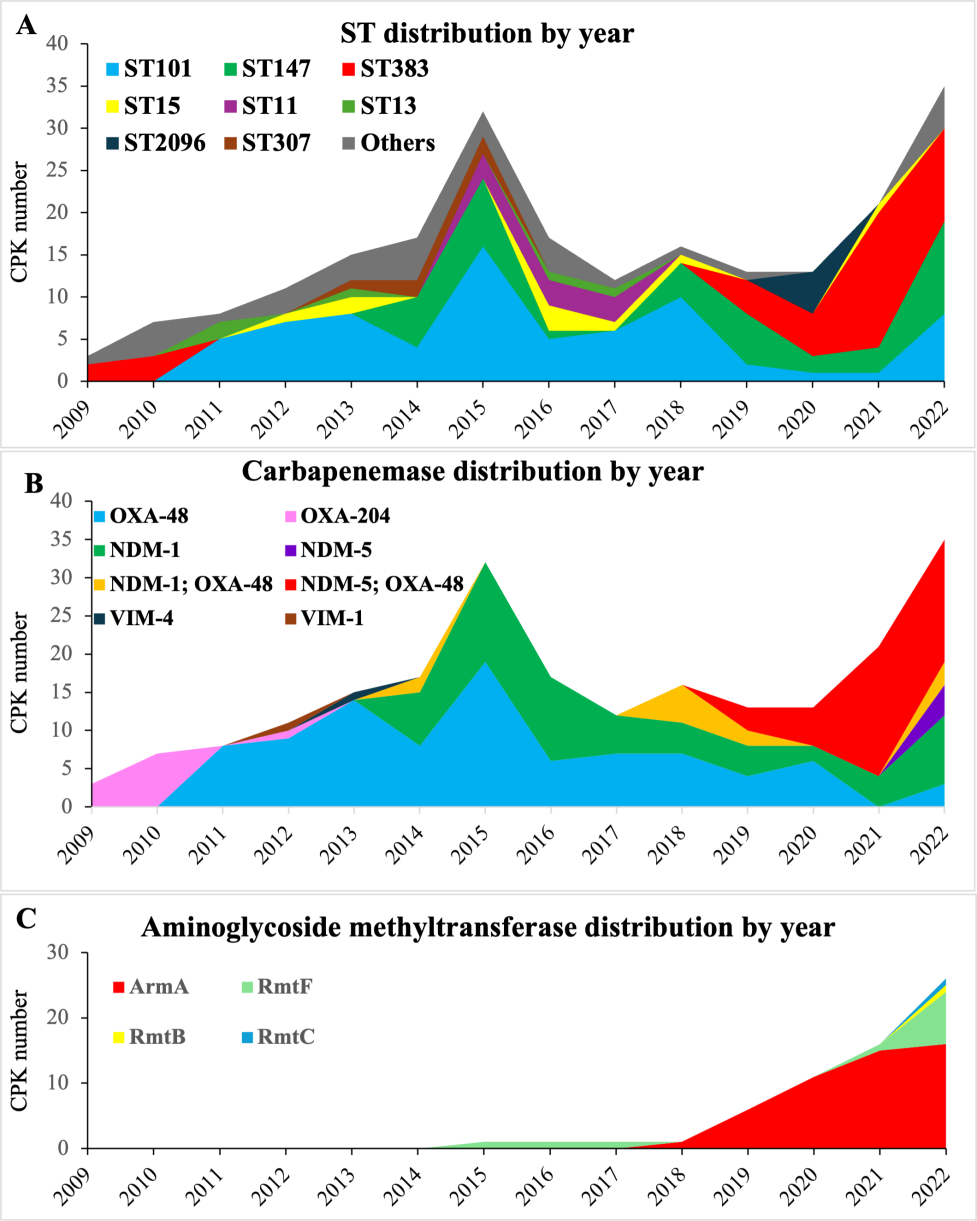
Temporal distribution of HBH’s bloodstream CPK isolates from 2009 to 2022. The X-axis shows years; the Y-axis shows the number of CPK isolated by year. Sample key shows the carbapenemase types. (**A**) Temporal distribution of clonal types; (**B**) temporal distribution of carbapenemase types; (**C**) temporal distribution of methylase types associated with aminoglycoside resistance.

Analyses identified shifting dynamics and evolving CPK isolates over time, with new STs and carbapenemase genes replacing prior isolates within HBH (Fig. 2; Fig. S2). ST101-OXA-48 predominated over 2011–2018. In contrast, ST383, which caused the first OXA-204 CPK epidemic in 2009–2010, reemerged in 2019 and became the predominant MLST while now encoding the OXA-48, NDM-5, and ArmA enzymes. ST147 has driven the dissemination of NDM-1 since 2014 and was prevalent through the end of the study period.

Among the bloodstream CPKs, we identified 61 (27.7%) convergent isolates among ST383 (*n* = 33), ST147 (*n* = 14), ST2096 (*n* = 5), and ST101 (*n* = 3). From 2019, the prevalence of *rmpA/iuc*-positive convergent isolates rose in ST383 (33/35; 94.3%) compared to other STs (24/47; 51.1%). Nevertheless, CPK bloodstream mortality over 2015–2022 did not differ relative to the presence or absence of these *rmpA/iuc* genes (34/51, 67%) (51/93, 55%) (*P* = 0.229).

### Carbapenemase-transmitting plasmids

Genomic analyses identified 22 plasmid replicons among the 95 bloodstream CPKs. Isolates carried a mean of 4.6 replicons per strain (median = 4; range of 1–8 replicons). Among the dominant STs (Fig. S3), ST147, associated with FIB/pKPHS1 replicons, commonly carried NDM-1 on FIB/pQil-FII_K_ replicons, while ST101 isolates, associated with repB1701, FIA(HI1), Col440I replicons, carried OXA-48 on IncL plasmids. In contrast, ST383 isolates most commonly harbored NDM-5 on plasmids with IncFIB(pNDM-Mar)-IncHI1B(pNDM-MAR) multireplicons and OXA-48 on IncL plasmids. Characterization of carbapenemase-transmitting plasmids was confirmed by functional conjugation assays with PCR-based confirmation of enzyme carriage in 62 (55.9 %) of the 111 carbapenemases detected among the 95 CPKs. For the remaining 44 of 49 cases (39.6%), MOB-suite reconstructions using draft short-read assemblies evaluated putative plasmids mobilizing spread.

Conjugation studies from these 95 bloodstream CPK isolates into the naive recipient strain J53 demonstrated transmissibility of the carbapenemase-transmitting plasmids, including 37 *bla*_OXA-48_-IncL and 1 *bla*_OXA-48_-IncM1 conjugative plasmid (38/53 OXA-48 CPKs, 71.7%), 5 *bla*_OXA-204_-IncC conjugative plasmids (5/8 OXA-204 plasmids, 62.5%), 18 *bla*_NDM-1_-IncFII_k_ conjugative plasmids, and 1 *bla*_NDM-1_-IncC conjugative plasmid (19/35 NDM-1 CPKs, 54.2%). Only one *bla*_NDM-5_-IncFIB-IncHI1B plasmid was transferred from an ST383 isolate (Tables S4 to S7).

Bioinformatic reconstructions of the carbapenemase-carrying plasmids (Table S4) identified two major IncL plasmids among bloodstream isolates, referred to as pHB1 (mob cluster AA018/AH562, OXA-48, CP018717.1), and pHB2 (AA002/AH529, OXA-48 + CTX-M-14, CP019078.1), with >96% coverage and >99% identity (Fig. S4 and S5; Tables S4 and S5; Tables S8 and S9), that disseminated *bla_OXA-48_* genes among bloodstream CPK STs in HBH and worldwide.

We identified four replicons associated with *bla*_NDM-1_: IncFII_K2_+FIB _(pQil)_ (23), IncFIB_(pQil)_ (5), IncFIB_K_+FII_K1_ (2), and IncC (2) (Fig. S6 to S9; Table S6). The *bla*_NDM-1_ pHB3 (FII_K2_+FIB_(pQil)_, AA018/AH562) plasmid had a backbone showing >90% coverage and >99% identity with p1203/15 (FII_K2_+FIB_(pQil)_, MW363916), a plasmid reported in a Polish ST147 outbreak that originated from Tunisia (23) (Fig. S6). This pHB3 plasmid occurred in six ST147 isolates from HBH, as well as in six other STs seen in HBH since 2014, highlighting its high transmissibility among STs and patient populations (Table S7). In contrast, the non-mobilizable *bla*_NDM-1_ pHB5 (IncFIB_(pQil)_, AA019/AH565), homologous to CP014757, occurred in only five ST147 (Fig. S7; Table S7).

The *bla*_NDM-5_ plasmid pHB4 (IncFIB_(pNDM-Mar)_-IncHI1B_(pNDM-MAR)_, AA405/AI436) showed close homology to plasmids CP091814 (Qatar, ST383), CP034201 (UK, ST383), and CP137386 (UK, ST147) (>87% coverage and >98% identity). These conjugative plasmids harbor multiple resistance (*bla*_NDM-5_, *bla*_CTX-M-15_, *armA*, *qnrS*, *aac(6’*)) and virulence genes (*iucABCD-iutA, rmpA*, *rmpA2*). In 2022, pHB4 was identified from CPK bloodstream isolates in ST147, ST29, and ST101 (Fig. S10).

**Fig 3:**
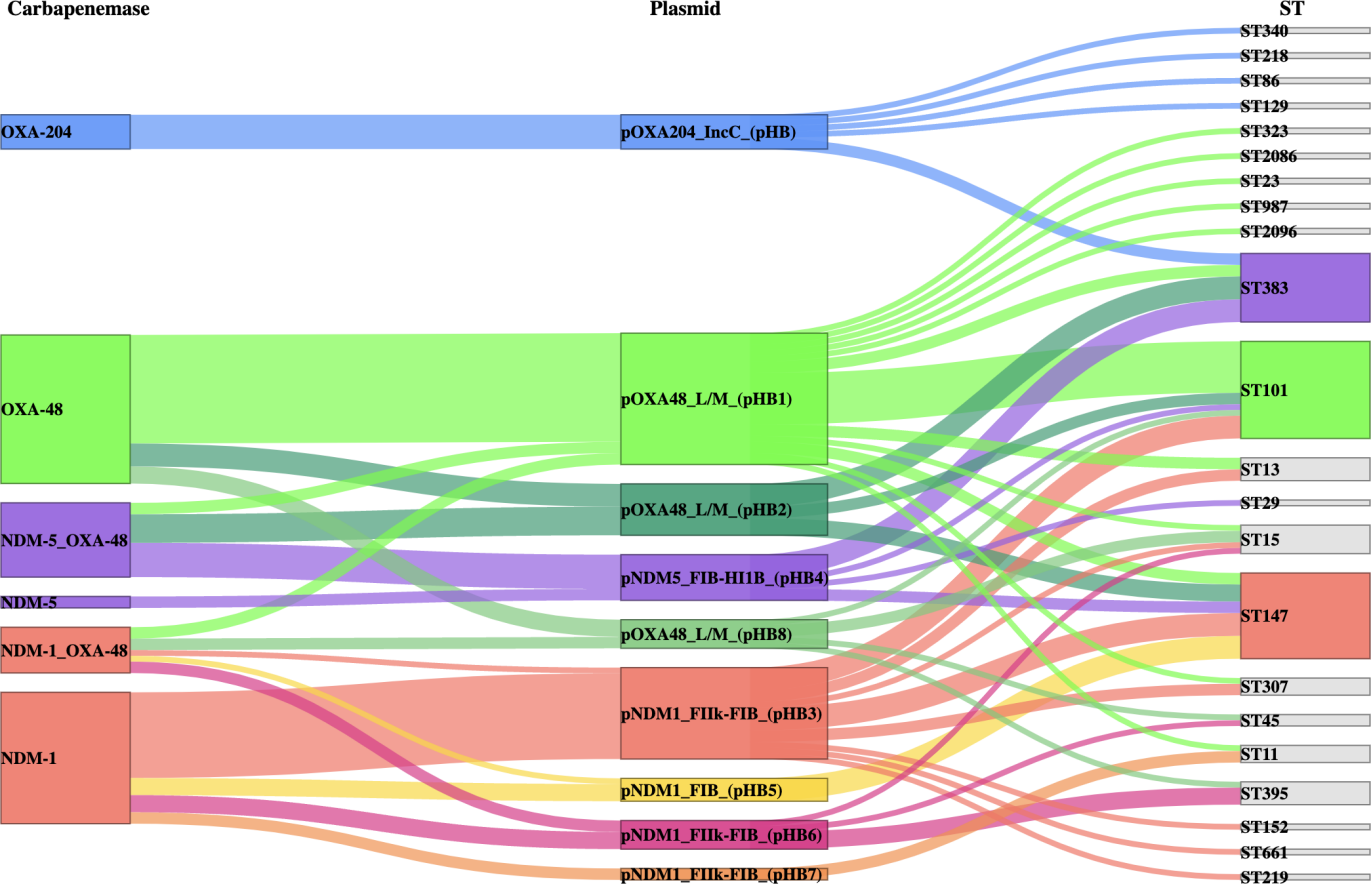
Sankey diagram illustrating relationships among the carbapenemase genes on the left, plasmid incompatibility and group types in the middle, and sequence types (ST) on the right. Line widths are proportional to the number of isolates and are colored based on carbapenemase-carrying plasmids noted in the middle section.

In aggregate, *bla*_OXA-48_ IncL plasmid pHB1, *bla*_NDM-1_ IncFII_K2_+FIB_(pQil)_ pHB3, and *bla*_NDM-5_ IncHI1B+FIB_(pNDM-Mar)_ pHB4 were the most prevalent and promiscuous plasmids within the HBH CPK population (Fig. 3).

### Genomic clustering identifies local to global strain relationships

Analysis using NCBI’s Pathogen Detection Resource showed that 68% (65 of 95 isolates) of HBH’s CPKs grouped with 26 international genomic NCBI single-nucleotide polymorphism (SNP) clusters. Three of these SNP clusters had been previously reported in Tunisia: ST101 and ST11 seen clinically, and ST147 identified in seafood. Five new clusters contained only isolates from this study, and 30 CPK isolates from HBH (31.6%) did not cluster with other NCBI-deposited *K. pneumoniae*, indicating that they lacked a close genomic match within the clustering thresholds used by the NCBI Pathogen Detection pipeline.

**Fig 4:**
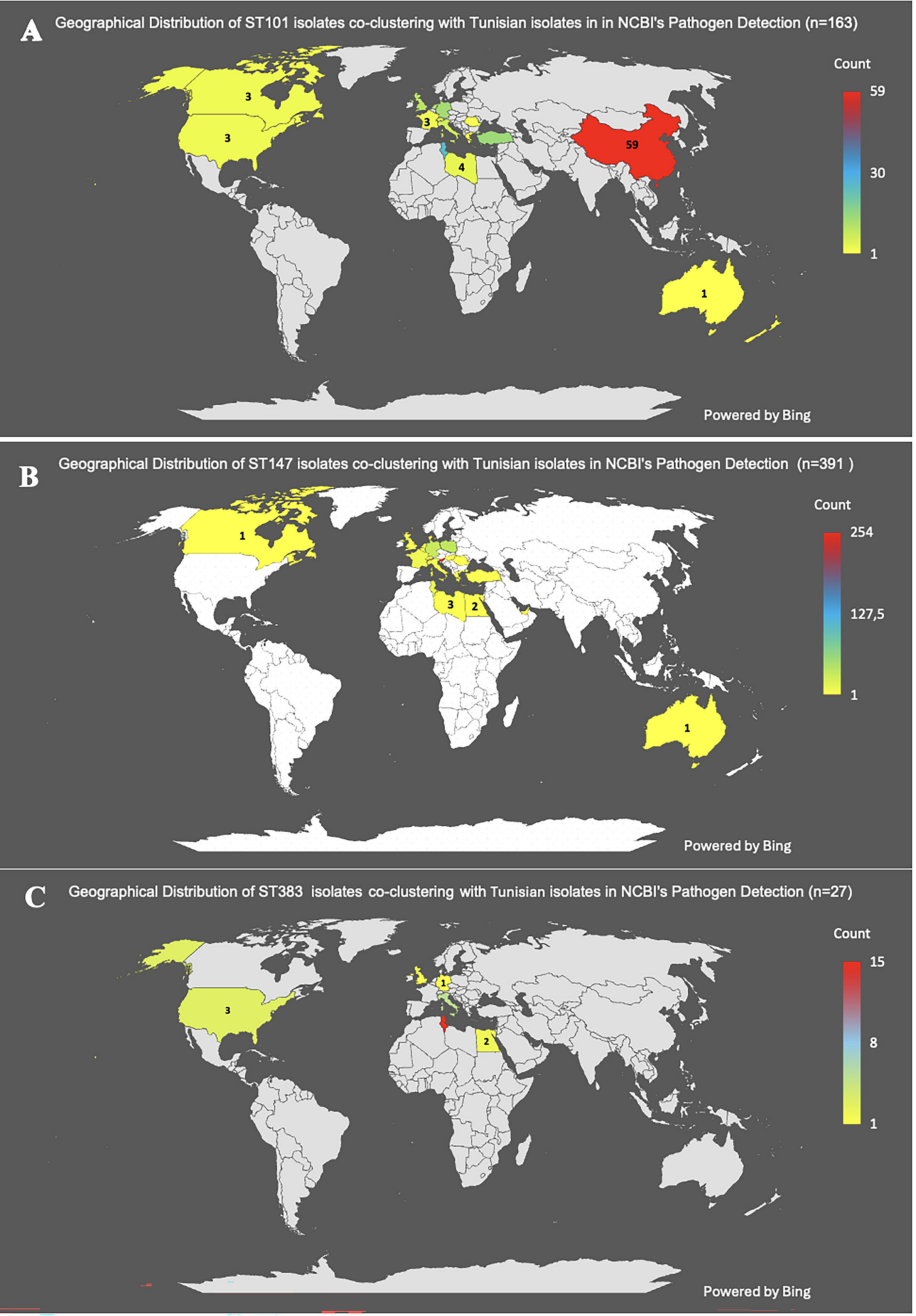
Geographic distribution of dominant STs seen in HBH. (**A**) ST101, (**B**) ST147, and (**C**) ST383. The color key and the number displayed in shaded countries indicate the number of isolates per country, retrieved from NCBI Pathogen Detection, that co-cluster with corresponding Tunisian clones. Genomic data accessed on 18 June 2025. Maps were generated using Bing Maps for Excel (Microsoft Office add-in). © Microsoft Bing.

Within HBH, 15 clusters involved 2–8 patients, suggesting potential internal transmission chains for at least 58% (*n* = 55) of sequenced isolates. Among the 55 isolates in ST101, ST147, and ST383, 45 (81%) clustered with isolates from 22 countries, particularly Mediterranean and other European countries, as well as the US, China, India, the Middle East, and Australia (Fig. 4).

To validate the relationships shown by NCBI’s clustering, whole-genome MLST (wgMLST) on 80,252 of the available 86,290 assemblies was used to identify the 100 most closely related genome assemblies. Phylogenetic estimation of the reference gene SNPs validated NCBI’s SNP-based clustering and phylogenetic relationships revealed among SNP clusters (Data S1A through D; interactive results at https://itol.embl.de/shared/pUTb8g8Zw3eX).

### Comparative genomics of HBH’s dominant ST383, ST147, and ST101 clusters

GalaxyTrakr analyses identified considerable diversity among ST101 (range 2–293 SNPs, mean 150 [SD 72]), ST147 (range 9–274, mean 166 [SD 76]), and ST383 isolates (range 2–251, mean 119 [SD 78]).

**Fig 5:**
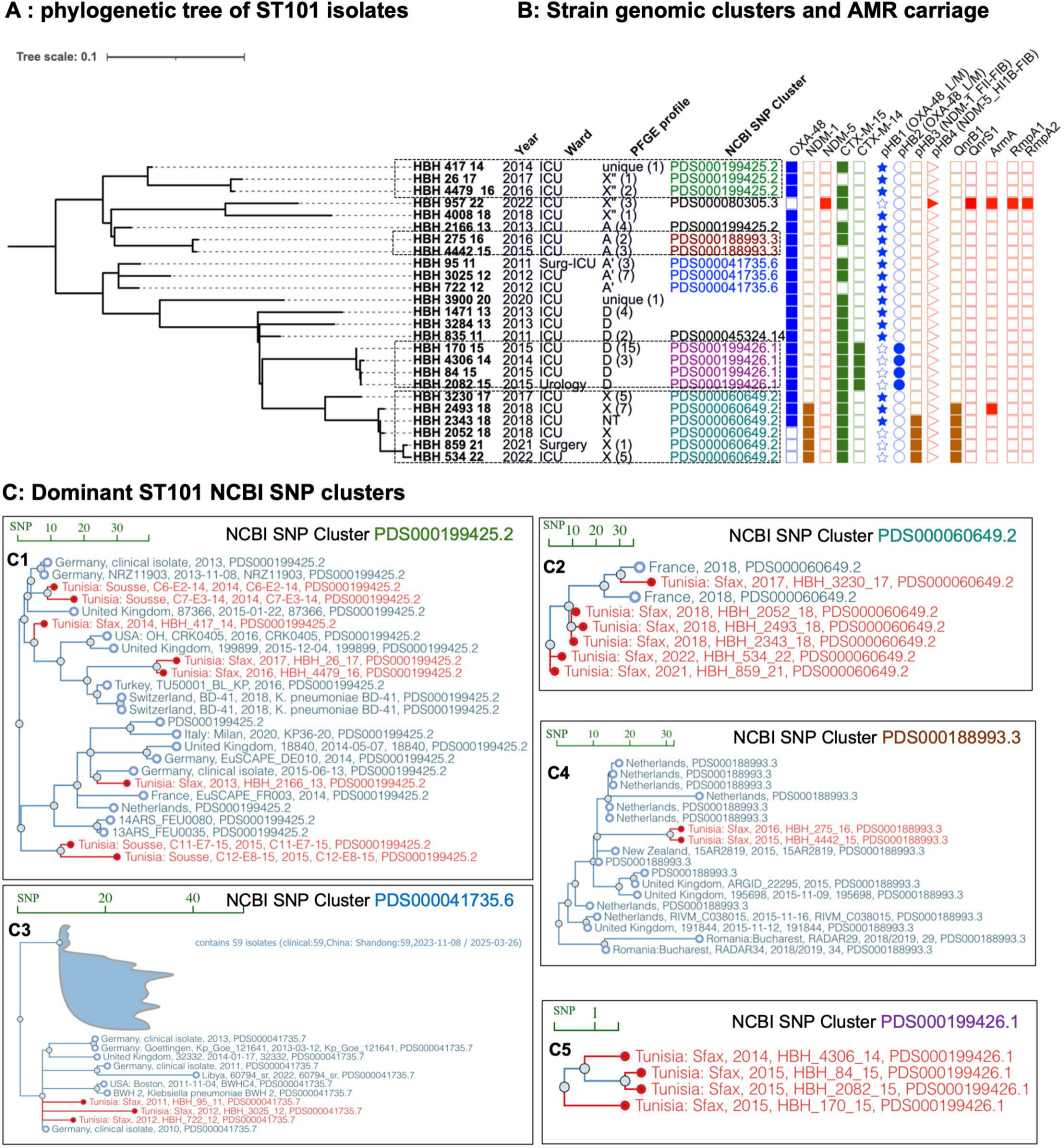
International context of ST101 genomic clusters seen in HBH. (**A**) Phylogenetic tree of ST101 isolates seen at HBH over 2009–2022. (**B**) Isolate metadata: isolation year and ward, NCBI SNP cluster ID, PFGE profile (parentheses show annual isolate counts for each profile), resistance genes, carbapenemase-producing plasmids. (**C**) NCBI SNP cluster analyses reveal that closely related isolates from different countries occur within the same NCBI SNP cluster, suggesting sporadic introduction into HBH (**C1, C2**), whereas other isolates are specific to the setting (**C3–C5**). Bar in each graph indicates the SNP distance, and the branches highlighted in red indicate the study isolates or the Tunisian strains from other settings. The NCBI SNP clusters are indicated in the same colors as in the phylogeny tree.

HBH’s first ST101 isolate in 2011 belonged to NCBI SNP cluster PDS000041735.6, which includes contemporaneous isolates from Germany, the UK, China, and the US, suggesting pre-existing global prevalence of this lineage. Within HBH, we detected four putative transmission events among ST101 isolates with SNP distance ≤21 (dotted outlined boxes, Fig. 5A). Transmitted isolates shared the same AMR profile and carbapenemase-transmitting plasmids (Fig. 5B), further indicating persistence of these clones within HBH over 2011–2022. However, some isolates within three ST101 NCBI SNP clusters showed closer SNP distances and were more closely related to isolates recovered outside HBH rather than to other HBH ST101 isolates, including Sousse-Tunisia (2014) (24), Germany (2013–2015) (25, 26), and the UK (2015) (27) for PDS000199425.2, Germany (2010) (25) for PDS000041735.6, and France (2017) (28) for PDS000060649.2 (Fig. 5C), supporting the hypothesis of reintroduction of these three ST101 sub-lineages into HBH at least one time during this period.

**Fig 6:**
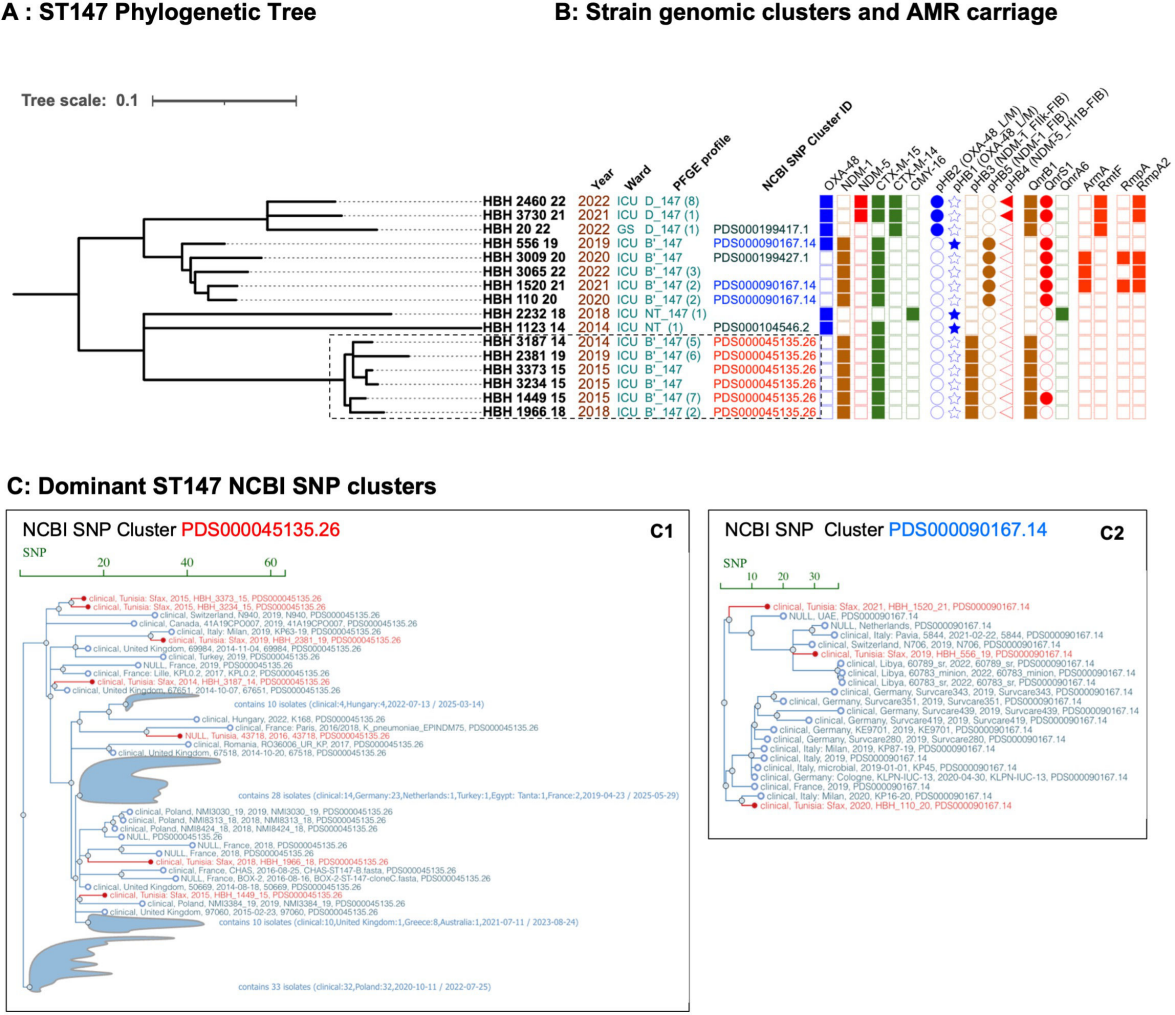
International context of ST147 genomic clusters seen in HBH. (**A**) Phylogenetic tree of ST147 isolates seen at HBH over 2009–2022. (**B**) Isolate features including ward, PFGE profile (parentheses show annual isolate counts for each profile), NCBI genomic cluster ID (if present), resistance genes, and mobilizing plasmids. (**C**) NCBI SNP cluster analyses reveal that many other closely related isolates from different countries fell within the same SNP cluster, supporting their repetitive introduction to the setting (**C1, C2**). Bar in each graph indicates the SNP distance, and the branches highlighted in red indicate the study isolates. The NCBI SNP clusters are indicated in the same colors as in the phylogeny tree.

Similar patterns occurred among the ST147 isolates, where *bla*_NDM-1_-containing isolates in NCBI cluster PDS000045135.26 appeared in HBH in 2014. Genomic analyses of 6 isolates from this cluster, representative of 22 ST147 with identical PFGE and AMR profiles isolated between 2014 and 2019, showed close SNP distances (9–25) and carriage of *bla*_NDM-1_ on pHB3 (pQIL, IncFII-FIB), suggesting persistence and transmission of this strain-plasmid complex within HBH (transparent boxes, Fig. 6A and B). However, NCBI Pathogen Detection’s analysis indicated that these isolates had closer SNP distances and were more closely related to isolates recovered in other countries, including Greece (2013–2018) (29), the UK (2014–2016) (27), Poland (2015–2019) (23), France (2018) (28), and Italy (2019, PRJNA1026096), as part of regional or global cryptic outbreaks in the late 2010s, within the large NCBI cluster PDS000045135.26 (100 isolates from 15 countries, Fig. 4B and 6C). These findings are consistent with repeated and sporadic introductions into HBH, at least four times between 2014 and 2019, of globally circulating ST147 lineages sharing a common ancestry and the same pHB3 *bla*_NDM-1_ plasmid, rather than a cross transmission of a single ST147 NDM-1 endemic clone (Fig. 4B and 6C; Data S1A through C, Trees 10–29). After 2019, we identified phylogenetically distinct ST147 harboring *bla*_NDM-1_ in the FIB pHB5 plasmid, as well as carriage of additional virulence plasmids.

**Fig 7:**
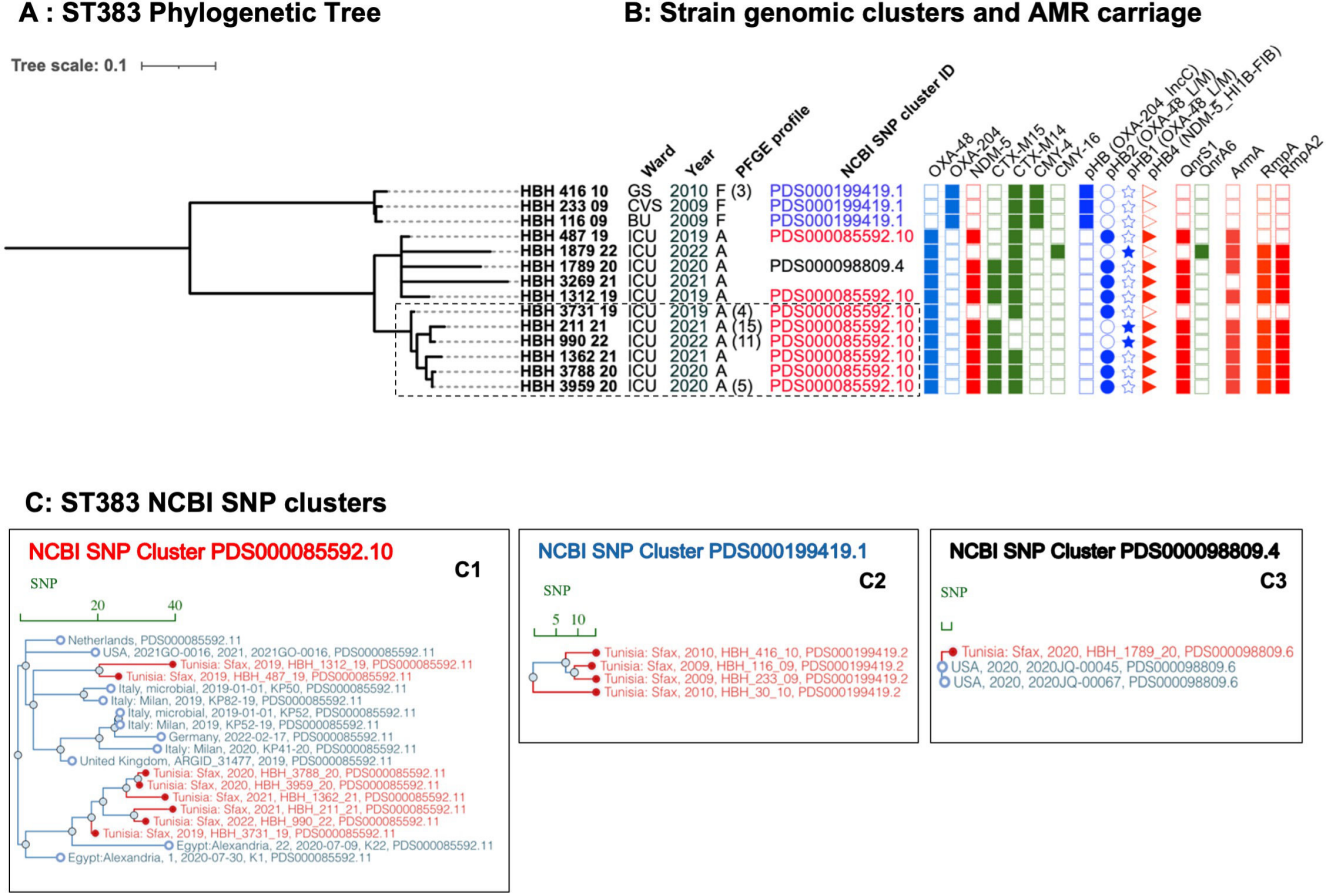
International context of ST383 genomic clusters seen in HBH. (**A**) Phylogenetic tree of ST383 isolates seen at HBH over 2009–2022. (**B**) Isolate features including ward, PFGE profile (parentheses show annual isolate counts for each profile), NCBI genomic cluster ID (if present), resistance genes, and mobilizing plasmids. (**C**) SNP cluster analyses reveal that many other closely related isolates from different countries fell within the same SNP cluster, supporting their repetitive introduction to the setting (**C1, C3**). Some clusters are specific to the setting (**C2**). Bar in each graph indicates the SNP distance, and the branches highlighted in red indicate the study isolates. The NCBI SNP clusters are indicated in the same colors as in the phylogeny tree.

Analyses also identified new and recent clusters of ST383 in HBH that co-clustered with isolates seen globally. Within these clusters, genomic analyses identified carriage of *bla*_NDM-5_ in the hybrid plasmid [pHB4] IncFIB(pNDM-Mar)-IncHI1B(pNDM-MAR). The ST383 isolates from 2009 to 2010 were separated by 8–13 SNPs from each other and were responsible for HBH’s first *bla*_OXA-204_ outbreak in 2009–2010 (10). These clonal isolates likely originated from a common ancestral strain carrying different replicons and resistance genes, and they formed a unique HBH-specific NCBI Pathogen Detection cluster (PDS000199419.1, Fig. 7C). In contrast, the isolates from 2019 to 2022 formed a separate SNP-defined group (corresponding to NCBI clusters PDS000085592.11 and PDS000098809.6) with 2–125 SNPs. These later isolates shared the same PFGE and AMR profiles, and *bla*_NDM-5_ hybrid plasmid: IncFIB(pNDM-Mar)-IncHI1B(pNDM-MAR), consistent with the multiple cross-transmission opportunities observed in HBH, as illustrated by the hospitalization-overlap timeline (Fig. S11). However, isolates belonging to these SNP clusters also originated from the Netherlands (2018) (30), the UK (2019) (31), Italy (2019, PRJNA1026096), Egypt (2020) (32), the US (2021, CDC PRJNA288601), and Germany (2022) (33), indicating the global dissemination of this lineage (Fig. 7C). Within HBH, genomic-epidemiologic analyses identified HBH_3269-21 and HBH_1879-22 as being part of patient transmission chains (Fig. 7A; Data S1B through D, Tree 27, 42, 43). The combined hospital-based epidemiologic data and placement of HBH isolates within an international context resolved nosocomial cross-transmission, namely within the ICU (Fig. 7A, dotted line boxes; Fig. S11), from putative sporadic reintroduction from outside reservoirs (Fig. 6C). Two distinct introduction events were detected, each corresponding to isolates showing stronger genomic relatedness to international strains than to local HBH isolates (Fig. 7C; Fig. S11).

### *K. pneumoniae* ST383’s phylogeny reveals recent origins contributing to four global outbreak events

**Fig 8:**
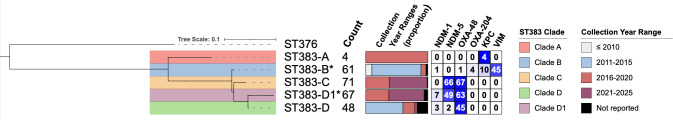
Global phylogenetic analysis of ST383/CG383 *K. pneumoniae*. Details about the clades, collection year, and the content of carbapenemases are shown. *: clades encompassing the HBH isolates.

*K. pneumoniae* ST383’s dominance at HBH (Fig. 2A) occurred in the context of limited global reports of these strains (Fig. 4C; Table S10). Nonetheless, we leveraged the combined HBH and international ST383 in NCBI Pathogen Detection to evaluate relationships among our recent patient isolates. The refined phylogeny of ST383 strains revealed a single monophyletic population of 258 members, composed of carbapenemase producers identified after 2010 [monophyletic clonal group 383 (CG383)]. Analyses identified four well-defined clades (Fig. 8, labeled A to D), which include a major descendant subclade D1, of clade D (Fig. 8; Data S1D, Tree 43). CG383 extends beyond ST383, including three ST6188 in subclade D1, two ST4853 isolates in clade D, and one ST5410 isolate in clade C (Data S1D, Tree 43).

While the earliest OXA-204 Tunisian ST383 (2009–2010) belonged to clade B (61 isolates), this clade mostly contained 2011–2018 VIM-producing outbreak isolates reported in Greece (29), the UK (27), the Netherlands (34), and Romania (35). In contrast, strains in more recent clades C (71 isolates) and D (115 isolates) acquired OXA-48 and NDM-5-hybrid plasmids (Fig. 8; Data S1D, Tree 43). These latter Tunisian strains clustered within clade D1, with 67 isolates first isolated in 2015–2016 in Egypt and Qatar (36).

Our longitudinal analyses of HBH’s ST383 CPK provide key bookends for the emergence of this concerning lineage, indicating origins around 2007, followed by repeated global dissemination (29). In contrast, we observed that NDM-5/OXA-48 ST383 lineages are associated with recently sequenced strains across internationally distributed clades, including those described longitudinally at HBH (Table S3).

## DISCUSSION

Genomic-epidemiologic analyses of patient-infecting CPK enable the understanding of their entry, dynamics, and mechanisms of spread within nosocomial environments. This information is critical to implement programs in infection prevention and control. We provide a high-resolution understanding of the dynamics of CPK over a 14-year period within a large Tunisian medical center. Given limited resources for genomic analyses, we leveraged clinical microbiologic phenotypic information with PFGE to select representative isolates for genomic-epidemiologic investigations. Our data revealed sustained increases in CPK incidence, consistent with national reports from Tunisia, other resource-limited countries, and with global alerts from WHO (1, 2, 16). This rise underlines the need to develop tailored infection prevention and control (IPC) programs for these settings.

While carriage of OXA-48-like carbapenemases occurred most frequently, we identified unexpected increases in NDM carriage, particularly of NDM-5 (37). Surprisingly, we did not detect KPC enzymes by genomics or clinical PCR testing, nor the globally dominant ST258 clone, suggesting that our setting diverges from global and regional Mediterranean epidemiological trends where ST258-KPC continues to predominate, and highlights the need for regional programs (4, 38).

Analyses of bloodstream CPK isolates revealed dynamic shifts in CPK populations, particularly among three lineages, ST101, ST147, and ST383 that carried OXA-48, NDM-1, and NDM-5+OXA-48, respectively. Although PFGE analysis suggested substantial intra-hospital transmission of these clones, the integration of whole-genome sequencing (WGS) and global contextualization within NCBI Pathogen Detection showed that some of these lineages had been reintroduced at least once and up to four times during the study period, raising suspicion for sporadic reintroduction from external sources. These findings inform the need for IPC programs to consider entry and transmission by colonized patients who may have acquired strains prior to admission, such as in neighboring healthcare institutions or travel to other countries.

While PFGE demonstrated less discriminatory power than genomic approaches, in a resource-constrained setting, it still provides useful information in selecting potentially related strains for genomic analyses. We also verified PFGE’s capacity to predict clonal associations among outbreak clusters of ST147 and ST383, particularly in settings where resources to conduct genomic analyses are limited, allowing use of this information to inform infection control and prevention strategies. However, continued analysis and vigilance are required to ensure the predictive capacity of PFGE to identify putative transmission chains.

Our longitudinal study confirms the endemicity of high-risk clones ST101 and ST147 among CPK in Tunisia and corroborates previous Tunisian reports describing mainly punctual outbreaks or studies limited in time or genomic scope (15). These findings align partly with European data, where a multicentric survey showed that although CG258-KPC remained dominant (30%), ST101-OXA-48 and ST147-NDM-1/KPC still accounted for 15% and 6% of CPK isolates, respectively, and circulated mainly in Serbia-Romania and Spain-Greece, reflecting restricted regional spread (38).

Both high-risk clones ST101 and ST147 have global distributions (4, 23). ST147 is endemic in India (*bla*_NDM_), Italy and Greece (*bla*_VIM_, *bla*_KPC_), and North African countries (*bla*_OXA-48_) (39). ST101 is mainly endemic in Mediterranean countries, where multiple OXA-48-producing outbreaks have been reported in Spain (40), Algeria (41), the Czech Republic (42), and Greece (43), with KPC-producing ST101 outbreaks in Italy (44).

Although previous reports described travel-related importation of ST147 and ST101 CPK from Tunisia into Poland and France (23, 28), our data show that many of our isolates were detected simultaneously, or earlier, in several other countries, supporting the hypothesis of repeated introductions or possible inter-country exchanges of these lineages. As admission screening was not performed at HBH, we cannot confirm the direction of these occurrences; however, our findings highlight the importance of such screening even in a highly endemic setting. Moreover, cases classified as “non-hospital-acquired,” with a first positive culture <48 h after admission, should be interpreted cautiously, as this cutoff does not distinguish true community versus nosocomial acquisition from prior healthcare exposures, in the absence of systematic CPK screening at admission.

We further identified emerging global, high-risk clusters of ST383 carrying *bla*_NDM-5_, *bla*_OXA-48_, on known virulence hybrid plasmids. These latter isolates accounted for 43% of HBH’s CPK bloodstream isolates from 2019 to 2022. The *bla*_NDM-5_, *bla*_OXA-48_ MDR ST383 strains were first detected in 2016 in Qatar (36), followed by isolated reports without evidence of wider expansion in the UK and Lebanon in 2018 (45, 46), then Italy, Germany, France, Tunisia, Libya, Egypt, Saudi Arabia, Singapore, and the US over 2019–2023 (32, 47). Genomic analyses of these isolates suggest that *bla*_NDM-5_-carriage occurs in emerging and highly drug-resistant epidemic clones (clades C and D1). In addition, the recent increases in ST383 global prevalence correlate with carriage of OXA-48 and NDM-5, while the earlier clade D strains, which lack NDM, have decreased in prevalence. Importantly, this emerging lineage is distinct from earlier clade B ST383 strains reported around 2009, including the VIM/KPC producers largely confined to Greece and the earlier OXA-204 ST383 isolates from HBH (Table S10; Fig. 8) (29). In contrast, our study is the first to demonstrate that the NDM-5/OXA-48 ST383 clone can sustain a large, 4-year epidemic. These ST383 provide a strong working example where international collaboration among low-resource African and Mediterranean countries has defined an emerging new health threat.

MDR plasmids transmitted the CPK carbapenemases. pBH3 IncFII_K2_+FIB_(pQil)_ carried *bla*_NDM-1_ among 30 isolates in 6 strain clusters, while pHB4 IncFIB_HI1B hybrid plasmid transmitted *bla*_NDM-5_ among 13 isolates in 4 clonal clusters. Two IncL plasmids, pHB1 and pHB2, transmitted *bla*_OXA-48_ across all lineages, suggesting chronic persistence of these replicons within HBH. Global contextualization of HBH isolates and AMR-transmitting plasmids further identified Mediterranean-focused transmission of *bla*_NDM-1_ by FII_K2_+FIB_(pQil)_, pKpQIL-like plasmids (23), while *bla*_OXA-48_ transmission occurred globally via IncL replicons (Data S1A through D) (48). In contrast, among HBH’s genomically analyzed CPKs, we did not detect IncX, and rarely IncC, which facilitate *bla*_NDM_ transmission in other regions (37).

Although 26% of our Tunisian CPK carried plasmid-associated virulence markers (*rmpA*/*rmpA2* and/or *iuc*), only four were string-test positive, and none were associated with classical hypervirulent *K. pneumoniae* syndromes*,* such as liver abscesses, endophthalmitis, or metastatic infections. These findings suggest that the presence of selected virulence factors alone does not translate into a hypervirulent phenotype and are consistent with the results of Kochan et al. (49), demonstrating that convergent strains do not always behave as classical hypervirulent *K. pneumoniae*, likely reflecting incomplete virulence repertoires, differences in capsule types, or other genetic effects. Notably, most of our convergent strains carried truncated or incomplete *rmpA2* genes, suggesting reduced functionality. Although truncated *rmpA2* variants have been commonly described in convergent strains and their functional contribution remains uncertain (50), these results underscore the importance of considering gene integrity and genomic context when interpreting hypervirulence potential.

Our findings identified a complex landscape of endemic strains with repeated sporadic introduction of CPK into HBH, with the capacity for entering strains to become established in the hospital. Although the hospital does not directly admit patients from foreign countries, it receives many indirect transfers from the private sector, particularly from ICUs that manage patients from neighboring countries. These findings highlight the need to screen high-risk patients for CPK carriage to inform local IPC strategies. In particular, ICU admission provides an established focal point to conduct CPK surveillance for this at-risk population. In addition, the clinical microbiology laboratory’s PCR-based testing for carbapenemases, combined with targeted PCR assays designed to rapidly identify the major high-risk lineages (ST101, ST147, and ST383), can inform timely cohorting of colonized patients and provide adaptable IPC strategies in hospital environments seen in resource-limited countries.

Limitations of our studies include the focus on bloodstream isolates, which represented 25% of all CPK isolates seen at HBH. While phenotypic and PFGE analyses indicated the bloodstream isolates to be reflective of our CPK population, this approach could have detected additional circulating clones. Although focused on one medical center, HBH is a major referral hospital contributing >20% of all *K. pneumoniae* reported to the LART network, which enhances the representativeness of the data. Our study was limited to clinical isolates and, in the absence of systematic screening, could not capture asymptomatic colonization and its contributions to CPK transmission. The absence of long-read sequencing also limited full plasmid resolution, although conjugation assays supported several replicon-carbapenemase associations. Nevertheless, our study provides a working example of how meaningful-resolution genomic analyses can be achieved in resource-limited countries by combining a PFGE-WGS strategy with publicly available resources and validated bioinformatic tools to achieve robust epidemiological surveillance. This approach underscores the need for global genomic surveillance to guide IPC strategies at hospitals on an international level.

## MATERIALS AND METHODS

### Clinical setting and study design

HBH is a tertiary 500-bed hospital with 22,000 admissions per year, serving as the main referral center for the entire southern region of the country. HBH includes 10 surgical and 3 medical wards, 1 medical ICU (22 beds), and 1 surgical ICU (5 beds). The SUD Committee of Persons’ Protection approved the study (reference number 0192/2019) with a waiver of consent. Patient cultures with the demographic information, including age, gender, and hospital admission, were retrieved from hospital records. We confirmed carbapenemase carriage clinically in all *K. pneumoniae* isolates with ertapenem MICs ≥ 0.5 mg/L and PCR. Clonal relationships among blood CPK isolates were further evaluated by PFGE, as previously described in our prior studies (9, 10). PFGE profiles were interpreted according to Tenover’s criteria, isolates differing by <3 bands were considered clonal (10). Isolates were selected by year from each pulsotype, phenotypic, and PCR profile for WGS.

### Antimicrobial susceptibility testing, PCR screening, and string test

Antimicrobial susceptibility testing was performed according to the European Committee on Antimicrobial Susceptibility by disk diffusion or broth microdilution for colistin, tigecycline, and aztreonam-avibactam. PCR-based analyses evaluated carriage of carbapenem resistance genes (*bla*_NDM,_ *bla*_IMP_, *bla*_KPC,_ *bla*_VIM_, and *bla*_OXA-48-like_), aminoglycoside methylase genes (*armA*, *rmtA*, *rmtB*, *rmtC*, *rmtD*, *rmtE*, *rmtF*), and virulence genes (*rmpA*, *rmpA2*, *iutA*, *ybtS*, *mrkD*) (51, 52). String test for the hypermucoviscous phenotype used colonies cultured on MacConkey agar plates as described (53).

### Conjugation assays

*In vitro* assays for transfer of carbapenemase genes from blood CPK isolates used rifampicin-resistant *Escherichia coli* J53-2 recipients. Transconjugants were selected on imipenem (2 μg/mL) and rifampicin (250 μg/mL) to counter-select donor strains and confirmed by PCR (10). We classified plasmids according to their incompatibility group by using the PCR-based replicon typing method as described previously (54).

### Genomic analyses

Genomic DNA was extracted using QIAamp DNA Mini Kit (Qiagen) and sequenced by 150 bp paired-end Illumina NovaSeq. Raw data were quality-checked with FastQC (55) and *de novo* assembled with SPAdes (v.3.14.0) (56). Kleborate v2.1, using default settings, identified strain multilocus sequence and capsule locus types, AMR, and virulence genes, and assessed *rmpA*/*rmpA2* gene truncation and coverage (57).

For cluster and outbreak analyses, we used the FDA Center for Food Safety and Nutrition’s SNP Pipeline in GalaxyTrakr (18, 19). Using GalaxyTrakr, SNP alignments were mapped to the highest-N50 isolate, and clonality was defined by the ≤21-SNP threshold (35). Phylogenetic trees were constructed using FastTree 2.0, visualized and annotated using iTOL (v6) (58). Sequencing reads were deposited to NCBI’s Sequence Read Archive (Bioproject PRJNA1160997) for rapid SNP clustering in NCBI Pathogen Detection to place strains within an international context, and call resistance genes (20, 21). International SNP clusters were defined according to the NCBI Pathogen Detection, which groups isolates based on core-genome SNP distances. Cluster identity and relatedness were checked using core genome SNP analysis as described below.

We considered a clone to be “reintroduced” to the setting when an identical PFGE profile persisted across different years, but whole-genome analyses (SNP distances and NCBI Pathogen Detection clusters) demonstrated that these isolates were genetically closer to isolates circulating in other countries than to earlier HBH isolates, suggesting repeated introductions rather than sustained transmission.

To validate the findings of the GalaxyTrakr SNP Pipeline and the NCBI Pathogen Detection tools, we conducted wgMLST and SNP analyses of the HBH isolates alongside the 100 closest genomes from the NCBI SRA (Table S11). From NCBI’s nucleotide database, 86,290 putative *Klebsiella pneumoniae* isolate genomes were downloaded as identified in the Pathogen Isolates browser on 13 February 2025 (Table S11) (20). From these, 84,393 were designated as *Klebsiella* by the program MLST, which was used to assign MLST types according to the Pasteur Institute typing scheme (59–61). For assembly quality control, two criteria were used: (i) 80 contigs or fewer contained more than 90% of the sequence information; (ii) total sequence length between 5.0378 and 6.2526 MB, approximating three standard deviations from the median sequence length plus and minus of available assemblies. Using these cutoffs, 80,252 sequence assemblies were used for phylogenetic analyses (Table S11).

Gene coding sequences (*n* = 5,316) from the reference genome CP003200.1 were used to create a wgMLST scheme (62). BLAST was used to identify gene sequence alignments that had an e-value under 0.001 and were all gapless, unique, and full-length (63). Distinct gene sequences were given a sequence type identity for wgMLST analyses (64). For phylogenetic estimation, SNP matrices were composed based on these sequences, replacing missing sequences with “N” characters. Trees were reduced to only sequence positions containing SNPs. Trees were calculated using RAxML-ng and its GTR+G substitution model with automatic bootstrap analysis to 1,000 bootstraps, and transfer bootstrap expectation as the branch confidence metric (65).

Closely related isolates were identified with ranking by perfect-match wgMLST allele counts relative to a selected seed strain. For SNP clustering analysis, the list of ranked isolates included was expanded from the seeded strain until 100 isolates submitted outside this study were included. For ST383 analysis, a seed was chosen, and the list was expanded until the total number of genomes was 400, which was confirmed to include all 248 ST383-typed assemblies. This set of 400 was pruned to just those identified by outgroup analysis using a cluster of six ST376 assemblies (Data S1D, Tree 42). Clonal group 383 (CG383) was defined as ST383 together with a few closely related sequence types that form a single monophyletic clade in core-genome SNP analysis. Phylogenetic tree visualizations were created using the Interactive Tree of Life with minor cosmetic alterations (58). Trees without known outgroups were analyzed using IToL midpoint rooting. For ST383 outgroup identification, several ML trees produced by RAxML without bootstrapping analysis were analyzed, which all indicated that a cluster of ST376 genomes was an appropriate outgroup. Global comparative genomic analysis of ST383 isolates was performed using RAxML-ng.

### Plasmid analyses

Plasmid replicon types were identified with PlasmidFinder 2.1 (66) and MOB-suite using the MOB-recon and MOB-typer modules (67) to identify carbapenemase-carrying plasmids from the draft assemblies per homology to plasmid markers (replicons, relaxases) and plasmids in the reference NCBI plasmid database (68). To aid in the visualization of plasmid alignments to a reference plasmid, we developed a program named PlasMap, available at https://github.com/worleyjn/PlasMap. This program uses the Genome Diagram package implemented in BioPython to visualize where contigs have aligned to reference plasmids, visualize the reference plasmids, visualize contigs with sequence not represented on the reference plasmid, and simultaneously observe plasmid gene content (69, 70). Reference plasmids and plasmid feature calls were downloaded from the NCBI Nucleotide database (Table S8). BLASTn was used to align draft genome contigs to reference plasmid sequences with cutoffs of an e-value <0.001 and length >500 (63). Plasmid-associated draft assembly contigs with any features <60% represented in sequence alignments were analyzed for unique gene content. Files produced in PDF format were edited when necessary for clarity.

### Statistics and visualization

Significant differences in counts-based categorical variables were evaluated in R (v4.4.2) by Chi-squared or Fisher’s exact test with the use of tidyverse and ggsankey for visualization (71). Phylogenetic trees were visualized in ITOL v6 (58). NCBI’s Pathogen Detection Isolates Browser evaluated the global context of dominant clones seen in HBH. PlasMap generated visual representations of the strain contigs aligned to homologous reference plasmids (20, 68).

## Data Availability

WGS data can be found under NCBI BioProject PRJNA1160997.

## SUPPLEMENTAL MATERIAL

Data S1A aac.00142-26-s0001.pdf Phylogenetic trees (1–12) generated in this study.

Data S1B aac.00142-26-s0002.pdf Phylogenetic trees (14–17) generated in this study.

Data S1C aac.00142-26-s0003.pdf Phylogenetic trees (28–41) generated in this study.

Data S1D aac.00142-26-s0004.pdf Phylogenetic trees (42–43) generated in this study.

Supplemental material aac.00142-26-s0005.pdf Fig. S1 to S12; Tables S1 and S2, S4 to S7, and S10.

Supplemental tables aac.00142-26-s0006.xlsx Tables S3, S8, S9, and S11.

ASM does not own the copyrights to Supplemental Material that may be linked to, or accessed through, an article. The authors have granted ASM a non-exclusive, world-wide license to publish the Supplemental Material files. Please contact the corresponding author directly for reuse.
