# Supplementary material for: Leveraging the global genomic epidemiology of carbapenemase-producing Klebsiella pneumoniae to inform infection prevention in Tunisian hospitals
Source: Antimicrob Agents Chemother. 2026 May 6;70(6):e00142-26. doi: 10.1128/aac.00142-26 (PMC13231914; doi:10.1128/aac.00142-26)

Supplementary Data 1: Additional phylogenetic dendrograms

Dendrograms in this appendix were created using RAxML-ng on a wgMLST derived SNP matrix containing positions with substitutions. Dendrogram members were selected by those with the most perfect wgMLST matches. The initial starting pool of genomes numbered 80,252.

Isolates submitted as part of this study have their Isolate and MLST colored dark red, and the isolate font is enlarged.

Countries are color coded by continent.

Gene content is blue when present, white when absent. KPC and VIM include all alleles, and the identities of all genes are available through the interactive trees at the link below.

Plasmid presence is a gradient from white (0% present) to blue (100% present). These correspond to the amount of the plasmid covered by unique blast hits longer than 500 b and with an e-value < 0.001.

Nodes and branches are colored based on their transfer bootstrap expectation, with red = 0 and green = 1.

Interactive versions of these trees and SNP difference matrices are available at:
https://itol.embl.de/shared/pUTb8g8Zw3eX

NCBI Pathogen Isolates database access date: February 13th, 2025.

The table of contents lists the MLSTs, SNP clusters, and important stats for each of the dendrograms.

Table of contents

#	File	Tree	Seed	# HBH Isolates	# Non-HBH Isolates	MLSTs	SNP Clusters	Matrix length (b)
1	1A	GCA_043858345	GCA_043858345	4	100	15, 2147	PDS000036279.2, PDS000041726.21, PDS000045329.17, PDS000046916.2, PDS000060613.1, PDS000066127.3, PDS000075194.4, PDS000100772.1, PDS000104501.4, PDS000106381.4, PDS000156654.1, PDS000179049.7, PDS000184430.3, PDS000199428.1, PDS000214445.1	3379
2	1A	GCA_043859295	GCA_043859295	1	100	323	PDS000007156.3, PDS000017083.21, PDS000041776.5, PDS000041777.3, PDS000064191.2, PDS000079407.1, PDS000093251.1, PDS000096681.1, PDS000103183.2, PDS000106604.3, PDS000107667.1, PDS000121548.1, PDS000170482.1, PDS000216815.1, PDS000216816.1	2931
3	1A	GCA_043859305	GCA_043859305	1	100	14	PDS000048890.1, PDS000063451.2, PDS000073429.1, PDS000074446.1, PDS000085075.2, PDS000098764.2, PDS000102332.1, PDS000104978.1, PDS000139927.2, PDS000146621.4, PDS000149382.2, PDS000164521.1, PDS000169108.4, PDS000172308.1, PDS000192187.1, PDS000194032.1, PDS000198392.1, PDS000198406.1, PDS000213373.1, PDS000215014.1, PDS000215017.1	7842
4	1A	GCA_043859365	GCA_043859365	4	100	129, 395, 1082, 1625, 1823	PDS000036210.15, PDS000040454.8, PDS000041774.4, PDS000044310.6, PDS000045400.2, PDS000053786.7, PDS000072337.1, PDS000076869.2, PDS000088522.1, PDS000095854.2, PDS000101907.1, PDS000114341.1, PDS000122140.2, PDS000149535.1, PDS000171747.1, PDS000171750.1, PDS000185317.1, PDS000190144.1, PDS000197597.1, PDS000201458.1	45842
5	1A	GCA_043859495	GCA_043859495	2	100	13	PDS000041826.3, PDS000041857.2, PDS000053017.24, PDS000056122.9, PDS000098778.2, PDS000104514.1, PDS000110898.1, PDS000121641.1, PDS000140054.5, PDS000155185.4, PDS000185322.1, PDS000194059.1, PDS000197052.1, PDS000217867.1	5125
6	1A	GCA_043859615	GCA_043859615	13	100	101, 2502	PDS000045320.1, PDS000045324.14, PDS000045328.1, PDS000054005.14, PDS000060649.2, PDS000080193.2, PDS000104479.25, PDS000166495.5, PDS000199426.1	1135
7	1A	GCA_043859815	GCA_043859815	1	100	23, 57	PDS000045398.1, PDS000060603.1, PDS000095240.1, PDS000100774.1, PDS000111295.2, PDS000111758.3, PDS000144603.1, PDS000160830.3, PDS000164559.2, PDS000168933.1, PDS000168937.2, PDS000183478.1, PDS000183493.1, PDS000183496.1, PDS000187849.1, PDS000192486.2, PDS000199499.1	3930
8	1A	GCA_043859855	GCA_043859855	1	101	44, 107, 219, 305, 514, 2449, 3636, 5756	PDS000036289.4, PDS000074438.1, PDS000074448.1, PDS000074449.1, PDS000112610.2, PDS000112630.1, PDS000114410.2, PDS000121675.1, PDS000148766.7, PDS000150182.3, PDS000150194.3, PDS000157999.11, PDS000170612.1, PDS000176509.1, PDS000181291.1, PDS000198446.1, PDS000215654.1	54969
9	1A	GCA_043859995	GCA_043859995	1	101	5, 504, 540, 1013, 1087, 2086, 3319, 4510, 6245	PDS000036226.2, PDS000083576.2, PDS000090692.1, PDS000108803.2, PDS000112629.2, PDS000123677.1, PDS000130868.1, PDS000140723.2, PDS000145396.1, PDS000165203.1, PDS000170846.1, PDS000170904.1, PDS000175697.1	36809
10	1A	GCA_043860015	GCA_043860015	1	100	147	PDS000006578.119, PDS000006642.4, PDS000009779.14, PDS000036185.10, PDS000040466.3, PDS000041784.1, PDS000052089.184, PDS000052092.1, PDS000060612.1, PDS000067059.3, PDS000076259.2, PDS000077015.34, PDS000083378.2, PDS000084451.3, PDS000096678.1, PDS000099727.3, PDS000104546.2, PDS000111778.2, PDS000129321.8, PDS000130855.1, PDS000132251.1, PDS000136742.4, PDS000156049.11, PDS000165990.1, PDS000187661.2	4189
11	1A	GCA_043860075	GCA_043860075	1	103	86, 3509, 5559	PDS000042808.1, PDS000074639.1, PDS000097499.1, PDS000100761.1, PDS000124950.1, PDS000168656.1, PDS000182304.3, PDS000183471.1, PDS000183505.1, PDS000197439.1, PDS000214453.1	5774
12	1A	GCA_043860195	GCA_043860195	1	103	163, 485, 496, 815, 914, 987, 2719, 3649, 4064, 6114	PDS000038919.5, PDS000053037.8, PDS000054678.2, PDS000059976.3, PDS000071977.4, PDS000080493.2, PDS000085062.4, PDS000105717.1, PDS000107774.2, PDS000112639.1, PDS000160856.1, PDS000161308.3, PDS000175698.1, PDS000178659.1, PDS000183523.1, PDS000183592.1, PDS000184186.2, PDS000197593.1, PDS000199202.1	40282

Table of contents, contniued

#	File	Tree	Seed	# HBH Isolates	# Non-HBH Isolates	MLSTs	SNP Clusters	Matrix length (b)
13	1A	GCA_043861645	GCA_043861645	3	100	11, 1640	PDS000045343.1, PDS000045344.10, PDS000045350.2, PDS000046910.86, PDS000046967.1, PDS000060651.29, PDS000079304.4, PDS000102670.3, PDS000103169.12, PDS000112591.1, PDS000138389.1, PDS000146165.3, PDS000148349.1, PDS000149983.1, PDS000165202.2, PDS000173787.1, PDS000185312.1, PDS000216219.1	3372
14	1B	GCA_043863025	GCA_043863025	4	101	307	PDS000038607.20, PDS000045377.17, PDS000045391.1, PDS000045393.5, PDS000051246.2, PDS000054607.22, PDS000056158.28, PDS000072661.1, PDS000073377.3, PDS000074878.9, PDS000075200.1, PDS000082283.3, PDS000104496.1, PDS000176502.1, PDS000189621.16, PDS000199421.1, PDS000199423.1, PDS000201306.1, PDS000201459.1, PDS000213377.1	2897
15	1B	GCA_043863145	GCA_043863145	2	100	45, 3031	PDS000006905.10, PDS000007035.4, PDS000008541.10, PDS000052944.18, PDS000067092.3, PDS000070441.1, PDS000074716.5, PDS000074859.1, PDS000078891.2, PDS000100089.1, PDS000100594.2, PDS000104481.1, PDS000104562.1, PDS000105716.1, PDS000106393.3, PDS000106675.2, PDS000109086.2, PDS000146617.5, PDS000162236.1, PDS000175704.1, PDS000178660.3, PDS000179055.2, PDS000192605.1, PDS000198705.1, PDS000206449.1, PDS000212921.2, PDS000214277.1, PDS000215653.1	5431
16	1B	GCA_043863165	GCA_043863165	3	100	395	PDS000036210.15, PDS000040454.8, PDS000041774.4, PDS000044310.6, PDS000045400.2, PDS000053740.1, PDS000053786.7, PDS000072337.1, PDS000077831.2, PDS000095854.2, PDS000112540.1, PDS000114341.1, PDS000156671.1, PDS000190144.1	3892
17	1B	GCA_043863205	GCA_043863205	2	101	218, 5939, 5943	PDS000074977.2, PDS000076457.1, PDS000079882.2, PDS000097290.2, PDS000100586.2, PDS000117560.1, PDS000183511.1, PDS000183580.1, PDS000190124.1, PDS000199420.1	5471
18	1B	GCA_043863245	GCA_043863245	1	101	152, 4513	PDS000036263.5, PDS000044881.3, PDS000071072.5, PDS000074978.1, PDS000075204.9, PDS000076193.10, PDS000088478.25, PDS000096689.1, PDS000100167.3, PDS000102786.1, PDS000106014.1, PDS000110342.1, PDS000170201.1, PDS000185318.1, PDS000186854.1, PDS000197056.1, PDS000197588.1, PDS000198509.1, PDS000201460.1, PDS000213526.2	11945
19	1B	GCA_043863305	GCA_043863305	1	100	661, 3409, 3453, 3655	PDS000041757.1, PDS000052192.6, PDS000053783.5, PDS000090686.2, PDS000093436.1, PDS000108371.1, PDS000142414.5, PDS000148773.2, PDS000160820.1, PDS000169090.1, PDS000170208.1, PDS000172294.1, PDS000185324.1, PDS000197609.1, PDS000198414.1, PDS000198827.1, PDS000203214.1	22027
20	1B	GCA_043863325	GCA_043863325	3	100	15	PDS000036279.2, PDS000041726.21, PDS000045329.17, PDS000045331.1, PDS000046916.2, PDS000053782.4, PDS000062513.1, PDS000066127.3, PDS000072336.1, PDS000075194.4, PDS000083760.2, PDS000104501.4, PDS000106761.1, PDS000115079.3, PDS000124952.2, PDS000170844.3, PDS000179049.7, PDS000186880.1, PDS000199428.1, PDS000214445.1	4301
21	1B	GCA_043863345	GCA_043863345	1	100	11, 3666	PDS000005579.7, PDS000013916.2, PDS000036336.53, PDS000045420.7, PDS000045421.52, PDS000065468.2, PDS000072335.6, PDS000077811.10, PDS000084821.3, PDS000092775.2, PDS000103182.2, PDS000113930.22, PDS000140731.18, PDS000156641.12	3532
22	1B	GCA_043863385	GCA_043863385	2	100	11	PDS000046910.86, PDS000060651.29, PDS000154385.11, PDS000165202.2, PDS000185312.1	2249
23	1B	GCA_043863405	GCA_043863405	3	100	13	PDS000046986.1, PDS000053017.24, PDS000056122.9, PDS000098778.2, PDS000157997.9, PDS000161927.6, PDS000173925.11, PDS000194059.1, PDS000197052.1, PDS000199430.1, PDS000211070.1	4337
24	1B	GCA_043863445	GCA_043863445	1	101	219	PDS000012112.164, PDS000179977.1, PDS000217221.1	3691
25	1B	GCA_043863535	GCA_043863535	1	100	147	PDS000006578.119, PDS000006642.4, PDS000009779.14, PDS000036182.26, PDS000036186.4, PDS000036295.1, PDS000040359.4, PDS000045262.1, PDS000052092.1, PDS000053519.1, PDS000065404.6, PDS000074889.1, PDS000075223.4, PDS000080300.2, PDS000083378.2, PDS000084451.3, PDS000097792.5, PDS000099727.3, PDS000100083.5, PDS000100389.1, PDS000104526.1, PDS000106769.6, PDS000130855.1, PDS000132251.1, PDS000156049.11, PDS000157007.2, PDS000164093.1, PDS000165990.1, PDS000197072.1	4594
26	1B	GCA_043904805	GCA_043904805	4	100	15, 2147	PDS000041726.21, PDS000045329.17, PDS000046916.2, PDS000060613.1, PDS000097194.4, PDS000100772.1, PDS000106381.4, PDS000124938.1, PDS000156654.1, PDS000179049.7, PDS000184430.3, PDS000188634.2, PDS000199428.1, PDS000214445.1	3822

Table of contents, continued

#	File	Tree	Seed	# HBH Isolates	# Non-HBH Isolates	MLSTs	SNP Clusters	Matrix length (b)
27	1B	GCA_043904845	GCA_043904845	11	100	383, 6118	PDS000060640.1, PDS000061038.3, PDS000070357.5, PDS000085592.11, PDS000098809.4, PDS000106324.3, PDS000106730.2, PDS000108392.5, PDS000133910.8, PDS000171211.1, PDS000175841.1, PDS000179530.2, PDS000180184.1, PDS000192050.8, PDS000202475.1, PDS000217883.1	2177
28	1C	GCA_043904865	GCA_043904865	1	100	340	PDS000017086.10, PDS000036321.13, PDS000083373.14, PDS000130841.1, PDS000130869.1	2174
29	1C	GCA_043904885	GCA_043904885	6	101	147	PDS000009779.14, PDS000026421.12, PDS000045135.24, PDS000045262.1, PDS000071979.8, PDS000080114.2, PDS000092779.3, PDS000104516.1	1031
30	1C	GCA_043905265	GCA_043905265	1	100	2096	PDS000060581.66	667
31	1C	GCA_043905305	GCA_043905305	6	101	383	PDS000007455.4, PDS000060640.1, PDS000070357.5, PDS000085592.11, PDS000106324.3, PDS000108392.5, PDS000133910.8, PDS000176495.1, PDS000199419.2, PDS000202475.1	1735
32	1C	GCA_043905425	GCA_043905425	1	100	147	PDS000091501.176, PDS000201182.1	226
33	1C	GCA_043905485	GCA_043905485	12	100	101, 2502, 3367	PDS000041735.6, PDS000045311.30, PDS000045312.1, PDS000045320.1, PDS000055633.53, PDS000080305.6, PDS000088521.1, PDS000188993.3, PDS000199424.2, PDS000199425.1	2177
34	1C	GCA_043905525	GCA_043905525	1	101	13	PDS000053017.24, PDS000056122.9, PDS000098778.2, PDS000173925.11, PDS000194059.1, PDS000197052.1, PDS000211070.1	2316
35	1C	GCA_043905545	GCA_043905545	1	101	29, 714, 5832	PDS000070366.1, PDS000101853.1, PDS000108354.1, PDS000122861.1, PDS000122913.1, PDS000133597.1, PDS000143149.1, PDS000143192.1, PDS000156350.1, PDS000169967.2, PDS000173086.1, PDS000182318.2, PDS000183535.1, PDS000183538.1, PDS000183548.1, PDS000188010.2, PDS000188027.1, PDS000198821.1, PDS000201454.1, PDS000214440.1	10581
36	1C	GCA_043905565	GCA_043905565	1	100	45, 1418, 2954, 3098	PDS000006905.10, PDS000052944.18, PDS000054827.4, PDS000060037.37, PDS000074703.6, PDS000074716.5, PDS000075208.5, PDS000093208.1, PDS000100594.2, PDS000101897.1, PDS000105716.1, PDS000106393.3, PDS000106675.2, PDS000127598.3, PDS000178660.3, PDS000179055.2, PDS000190165.1, PDS000198705.1, PDS000208562.1, PDS000212921.2, PDS000213518.2, PDS000214277.1	12155
37	1C	GCA_043905585	GCA_043905585	12	100	101, 2502	PDS000045324.14, PDS000045328.1, PDS000054005.14, PDS000060649.2, PDS000080193.2, PDS000104479.25, PDS000166495.5, PDS000199426.1	1107
38	1C	GCA_043905605	GCA_043905605	11	101	383, 6118	PDS000060640.1, PDS000061038.3, PDS000070357.5, PDS000085592.11, PDS000098809.4, PDS000106324.3, PDS000106730.2, PDS000108392.5, PDS000125001.14, PDS000133910.8, PDS000171211.1, PDS000175841.1, PDS000179530.2, PDS000180184.1, PDS000192050.8, PDS000202475.1, PDS000217883.1	2222
39	1C	GCA_043905625	GCA_043905625	1	100	147	PDS000091501.176, PDS000199417.1, PDS000210545.1	252
40	1C	GCA_043905645	GCA_043905645	5	100	147, 4843	PDS000052089.184, PDS000056180.7, PDS000077015.34, PDS000090167.14, PDS000103185.4, PDS000104520.1, PDS000106697.6, PDS000129332.6, PDS000136743.8, PDS000156049.11, PDS000199427.1, PDS000201444.2, PDS000205531.1	1758
41	1C	GCA_043905665	GCA_043905665	1	100	147	PDS000091501.176	252
42	1D	MLST383_top400.raxml.support	GCA_043905605	15	385	42, 110, 231, 376, 383, 413, 750, 1190, 1588, 2947, 4295, 4853, 5079, 5410, 5875, 6118, 6125	PDS000007455.4, PDS000041770.3, PDS000050643.3, PDS000052267.2, PDS000056155.1, PDS000059234.2, PDS000060640.1, PDS000061038.3, PDS000065279.1, PDS000065282.1, PDS000070357.5, PDS000071539.1, PDS000072925.3, PDS000074875.1, PDS000074905.1, PDS000074991.1, PDS000077168.1, PDS000077820.1, PDS000078846.2, PDS000083580.1, PDS000085591.7, PDS000085592.11, PDS000093234.4, PDS000098809.4, PDS000101835.1, PDS000104969.1, PDS000106030.1, PDS000106324.3, PDS000106730.2, PDS000106886.14, PDS000107419.2, PDS000108392.5, PDS000125001.14, PDS000133910.8, PDS000141863.2, PDS000143218.1, PDS000149358.4, PDS000161102.14, PDS000166706.2, PDS000171211.1, PDS000171212.1, PDS000172292.1, PDS000172301.1, PDS000175841.1, PDS000176495.1, PDS000179530.2, PDS000180184.1, PDS000186585.1, PDS000188960.4, PDS000192050.8, PDS000192602.1, PDS000194043.1, PDS000194063.1, PDS000198706.1, PDS000198822.1, PDS000199419.2, PDS000202475.1, PDS000205525.1, PDS000210726.1, PDS000217883.1	89712
43	1D	ST383_ST376	GCA_043905605	15	249	376, 383, 4853, 5410, 6118	PDS000007455.4, PDS000050643.3, PDS000060640.1, PDS000061038.3, PDS000070357.5, PDS000077168.1, PDS000085591.7, PDS000085592.11, PDS000098809.4, PDS000106324.3, PDS000106730.2, PDS000108392.5, PDS000125001.14, PDS000133910.8, PDS000141863.2, PDS000161102.14, PDS000171211.1, PDS000171212.1, PDS000175841.1, PDS000176495.1, PDS000179530.2, PDS000180184.1, PDS000188960.4, PDS000192050.8, PDS000192602.1, PDS000199419.2, PDS000202475.1, PDS000205525.1, PDS000217883.1	9974

Tree 1: GCA_043858345

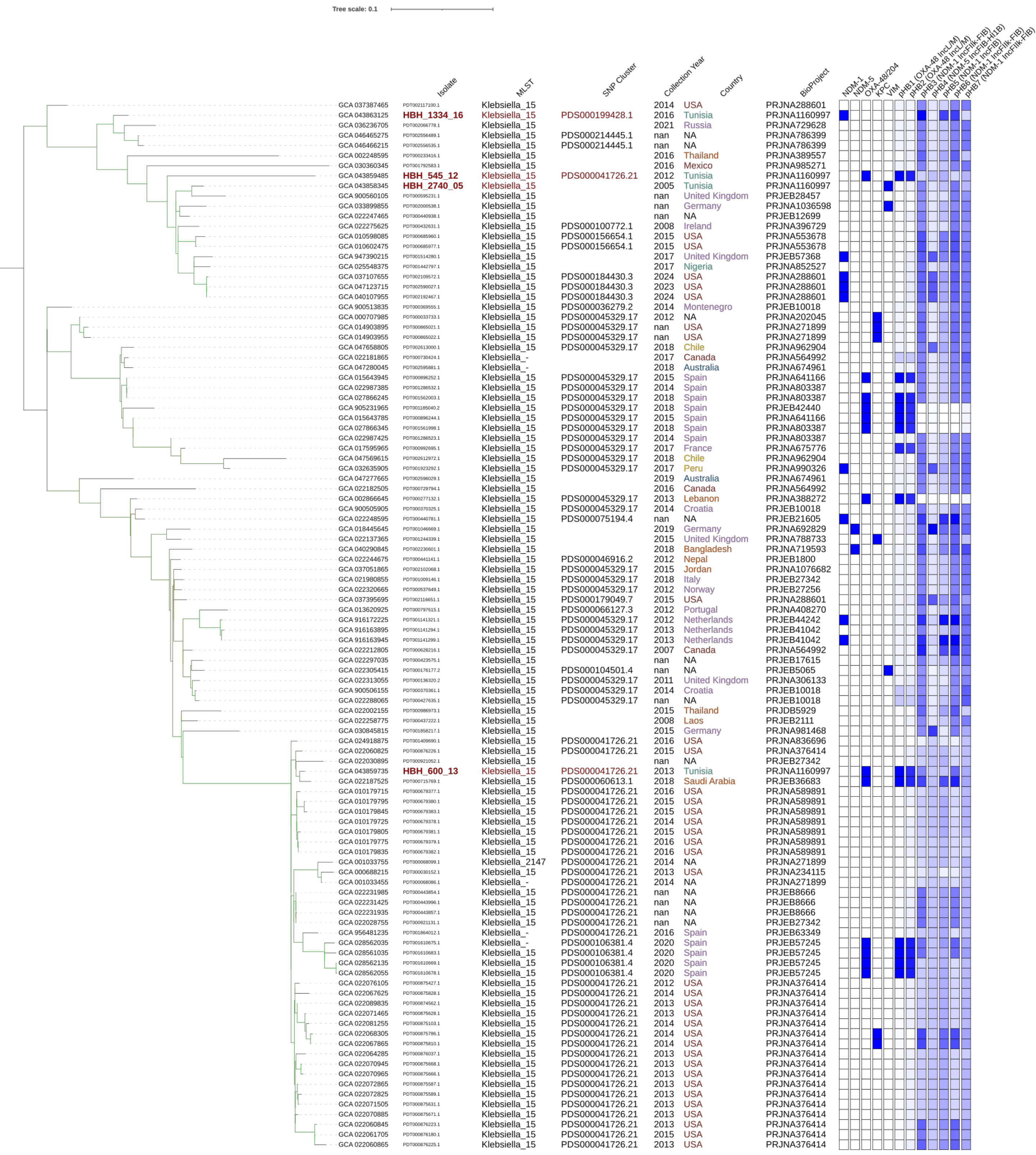

Tree 2: GCA_043859295

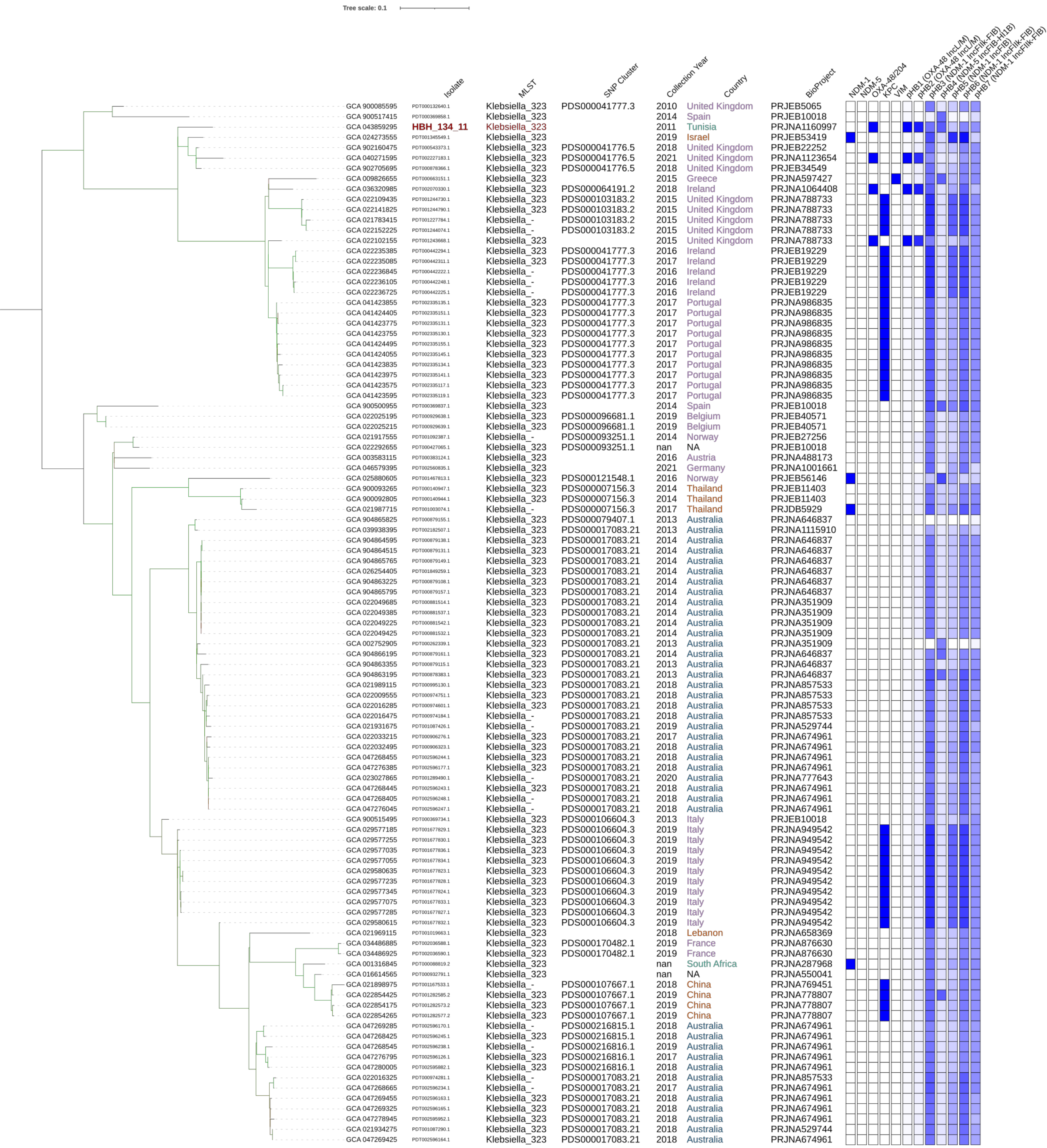

Tree 3: GCA_043859305

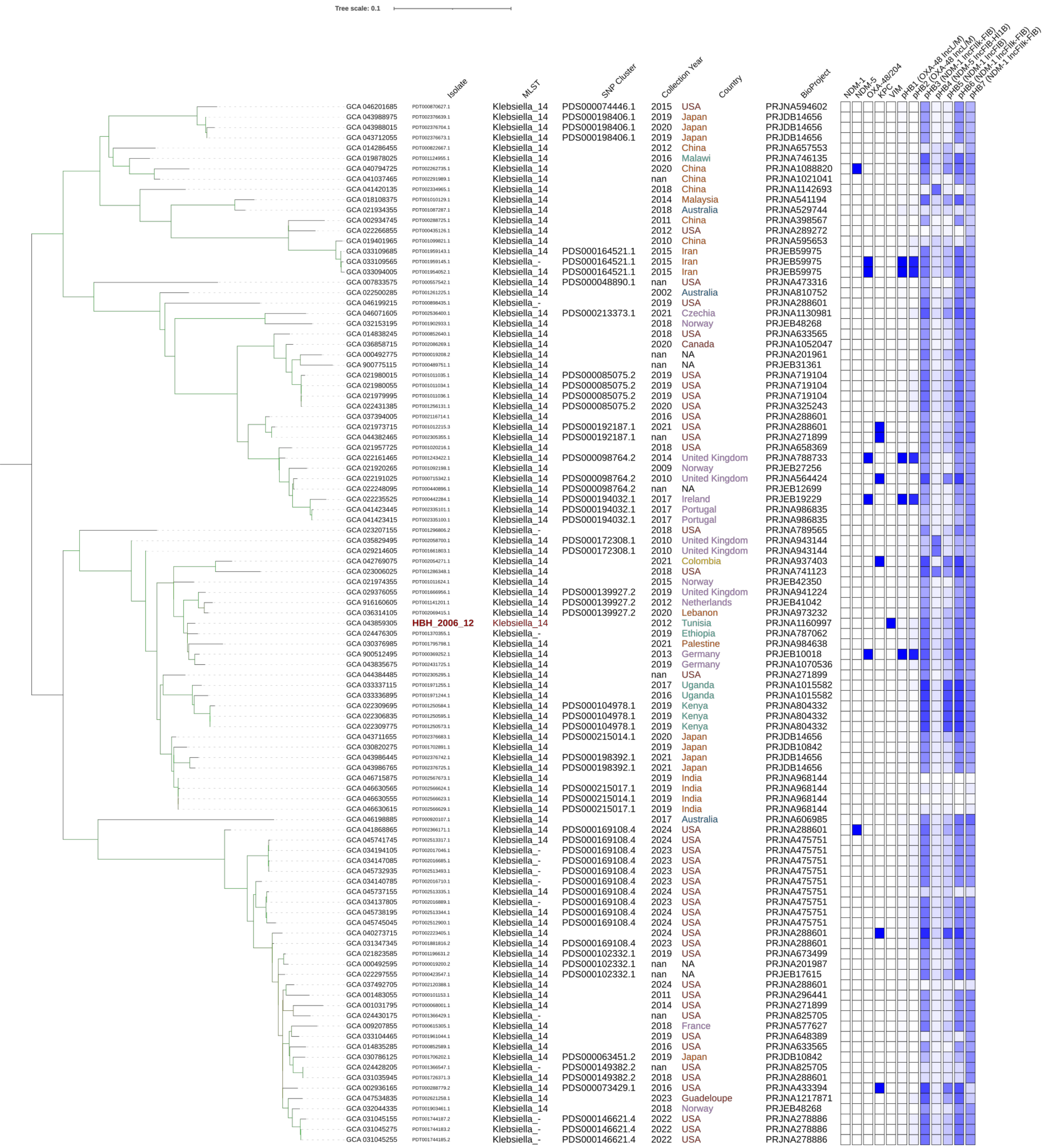

Tree 4: GCA_043859365

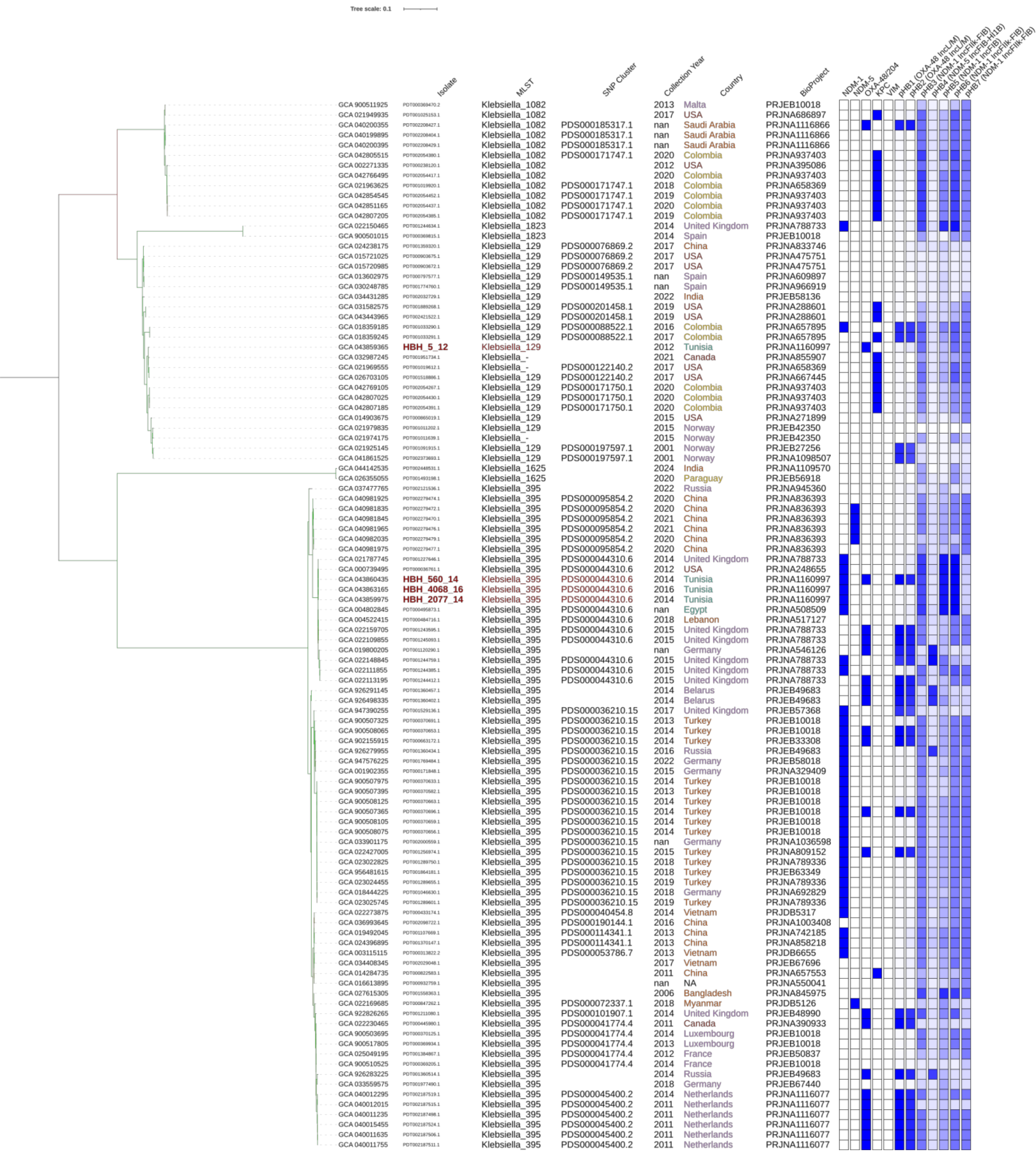

Tree 5: GCA_043859495

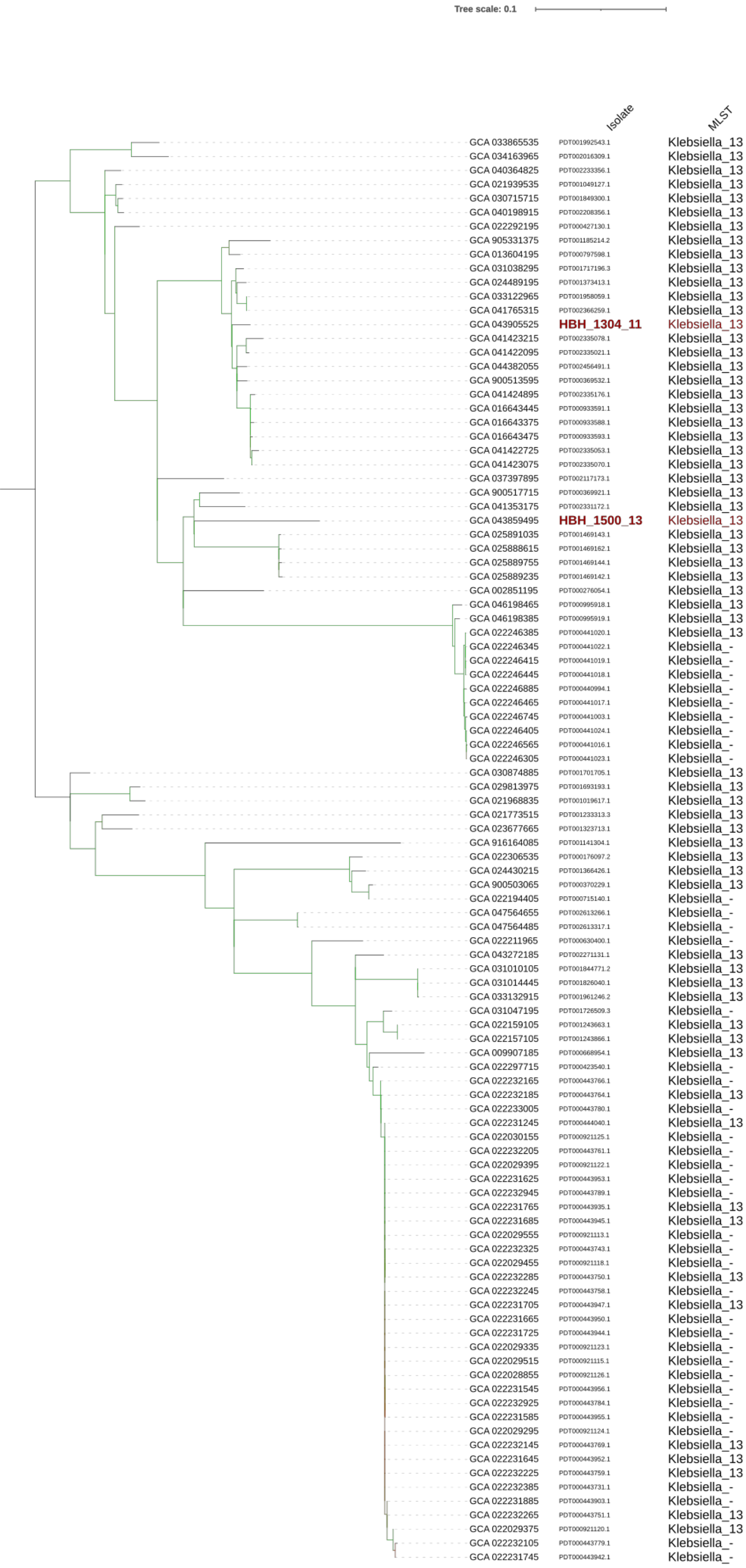

Isolate (GCA)	Isolate (PDT/HBH)	MLST	SNP Cluster	Collection Year	Country	BioProject
GCA 033865535	PDT001992543.1	Klebsiella_13		2016	Canada	PRJNA532924
GCA 034163965	PDT002016309.1	Klebsiella_13		2023	USA	PRJNA475751
GCA 040364825	PDT002233356.1	Klebsiella_13		2015	Japan	PRJDB17849
GCA 021939535	PDT001049127.1	Klebsiella_13		2018	Australia	PRJNA857533
GCA 030715715	PDT001849300.1	Klebsiella_13	PDS000185322.1	2019	Oman	PRJNA999478
GCA 040198915	PDT002208356.1	Klebsiella_13	PDS000185322.1	nan	Saudi Arabia	PRJNA1116866
GCA 022292195	PDT000427130.1	Klebsiella_13		nan	NA	PRJEB10018
GCA 905331375	PDT001185214.2	Klebsiella_13		2018	Spain	PRJEB42440
GCA 013604195	PDT000797598.1	Klebsiella_13		nan	Spain	PRJNA609897
GCA 031038295	PDT001717196.3	Klebsiella_13		2023	USA	PRJNA288601
GCA 024489195	PDT001373413.1	Klebsiella_13		2019	Spain	PRJEB50822
GCA 033122965	PDT001958059.1	Klebsiella_13	PDS000197052.1	2015	Norway	PRJEB27256
GCA 041765315	PDT002366259.1	Klebsiella_13	PDS000197052.1	2015	Norway	PRJNA1098507
GCA 043905525	**HBH_1304_11**	Klebsiella_13		2011	Tunisia	PRJNA1160997
GCA 041423215	PDT002335078.1	Klebsiella_13	PDS000194059.1	2017	Portugal	PRJNA986835
GCA 041422095	PDT002335021.1	Klebsiella_13	PDS000194059.1	2017	Portugal	PRJNA986835
GCA 044382055	PDT002456491.1	Klebsiella_13		nan	Saudi Arabia	PRJNA1116866
GCA 900513595	PDT000369532.1	Klebsiella_13		2014	Israel	PRJEB10018
GCA 041424895	PDT002335176.1	Klebsiella_13	PDS000056122.9	2017	Portugal	PRJNA986835
GCA 016643445	PDT000933591.1	Klebsiella_13	PDS000056122.9	nan	Portugal	PRJNA602991
GCA 016643375	PDT000933588.1	Klebsiella_13	PDS000056122.9	nan	Portugal	PRJNA602991
GCA 016643475	PDT000933593.1	Klebsiella_13	PDS000056122.9	nan	Portugal	PRJNA602991
GCA 041422725	PDT002335053.1	Klebsiella_13	PDS000056122.9	2017	Portugal	PRJNA986835
GCA 041423075	PDT002335070.1	Klebsiella_13	PDS000056122.9	2017	Portugal	PRJNA986835
GCA 037397895	PDT002117173.1	Klebsiella_13		2014	USA	PRJNA288601
GCA 900517715	PDT000369921.1	Klebsiella_13		2014	Spain	PRJEB10018
GCA 041353175	PDT002331172.1	Klebsiella_13		nan	Spain	PRJNA985214
GCA 043859495	**HBH_1500_13**	Klebsiella_13		2013	Tunisia	PRJNA1160997
GCA 025891035	PDT001469143.1	Klebsiella_13	PDS000121641.1	2016	Malaysia	PRJEB51966
GCA 025888615	PDT001469162.1	Klebsiella_13	PDS000121641.1	2016	Malaysia	PRJEB51966
GCA 025889755	PDT001469144.1	Klebsiella_13	PDS000121641.1	2016	Malaysia	PRJEB51966
GCA 025889235	PDT001469142.1	Klebsiella_13	PDS000121641.1	2016	Malaysia	PRJEB51966
GCA 002851195	PDT000276054.1	Klebsiella_13		2004	Colombia	PRJNA378654
GCA 046198465	PDT000995918.1	Klebsiella_13	PDS000041857.2	2006	Germany	PRJNA716285
GCA 046198385	PDT000995919.1	Klebsiella_13	PDS000041857.2	2006	Germany	PRJNA716285
GCA 022246385	PDT000441020.1	Klebsiella_13	PDS000041857.2	nan	NA	PRJEB12699
GCA 022246345	PDT000441022.1	Klebsiella_-	PDS000041857.2	nan	NA	PRJEB12699
GCA 022246415	PDT000441019.1	Klebsiella_-	PDS000041857.2	nan	NA	PRJEB12699
GCA 022246445	PDT000441018.1	Klebsiella_-	PDS000041857.2	nan	NA	PRJEB12699
GCA 022246885	PDT000440994.1	Klebsiella_-	PDS000041857.2	nan	NA	PRJEB12699
GCA 022246465	PDT000441017.1	Klebsiella_-	PDS000041857.2	nan	NA	PRJEB12699
GCA 022246745	PDT000441003.1	Klebsiella_-	PDS000041857.2	nan	NA	PRJEB12699
GCA 022246405	PDT000441024.1	Klebsiella_-	PDS000041857.2	nan	NA	PRJEB12699
GCA 022246565	PDT000441016.1	Klebsiella_-	PDS000041857.2	nan	NA	PRJEB12699
GCA 022246305	PDT000441023.1	Klebsiella_-	PDS000041857.2	nan	NA	PRJEB12699
GCA 030874885	PDT001701705.1	Klebsiella_13		2019	Japan	PRJDB10842
GCA 029813975	PDT001693193.1	Klebsiella_13		2017	Australia	PRJNA916637
GCA 021968835	PDT001019617.1	Klebsiella_13		2018	Lebanon	PRJNA658369
GCA 021773515	PDT001233313.3	Klebsiella_13	PDS000140054.5	2021	USA	PRJNA288601
GCA 023677665	PDT001323713.1	Klebsiella_13	PDS000110898.1	2018	Australia	PRJNA797179
GCA 916164085	PDT001141304.1	Klebsiella_13		2014	Netherlands	PRJEB41042
GCA 022306535	PDT000176097.2	Klebsiella_13		nan	NA	PRJEB5065
GCA 024430215	PDT001366426.1	Klebsiella_13	PDS000053017.24	nan	USA	PRJNA825705
GCA 900503065	PDT000370229.1	Klebsiella_13	PDS000098778.2	2014	United Kingdom	PRJEB10018
GCA 022194405	PDT000715140.1	Klebsiella_-	PDS000098778.2	2014	United Kingdom	PRJNA564424
GCA 047564655	PDT002613266.1	Klebsiella_-	PDS000217867.1	2019	Chile	PRJNA962904
GCA 047564485	PDT002613317.1	Klebsiella_-	PDS000217867.1	2019	Chile	PRJNA962904
GCA 022211965	PDT000630400.1	Klebsiella_-		2011	Canada	PRJNA564992
GCA 043272185	PDT002271131.1	Klebsiella_13		2018	Netherlands	PRJEB45369
GCA 031010105	PDT001844771.2	Klebsiella_13	PDS000155185.4	2023	USA	PRJNA288601
GCA 031014445	PDT001826040.1	Klebsiella_13	PDS000155185.4	2023	USA	PRJNA288601
GCA 033132915	PDT001961246.2	Klebsiella_13	PDS000155185.4	2023	USA	PRJNA288601
GCA 031047195	PDT001726509.3	Klebsiella_-		2018	USA	PRJNA288601
GCA 022159105	PDT001243663.1	Klebsiella_13	PDS000104514.1	2015	United Kingdom	PRJNA788733
GCA 022157105	PDT001243866.1	Klebsiella_13	PDS000104514.1	2015	United Kingdom	PRJNA788733
GCA 009907185	PDT000668954.1	Klebsiella_13		2015	Nigeria	PRJNA600702
GCA 022297715	PDT000423540.1	Klebsiella_-		nan	NA	PRJEB17615
GCA 022232165	PDT000443766.1	Klebsiella_-	PDS000041826.3	nan	NA	PRJEB8666
GCA 022232185	PDT000443764.1	Klebsiella_13	PDS000041826.3	nan	NA	PRJEB8666
GCA 022233005	PDT000443780.1	Klebsiella_-	PDS000041826.3	nan	NA	PRJEB8666
GCA 022231245	PDT000444040.1	Klebsiella_13	PDS000041826.3	nan	NA	PRJEB8666
GCA 022030155	PDT000921125.1	Klebsiella_-	PDS000041826.3	nan	NA	PRJEB27342
GCA 022232205	PDT000443761.1	Klebsiella_-	PDS000041826.3	nan	NA	PRJEB8666
GCA 022029395	PDT000921122.1	Klebsiella_-	PDS000041826.3	nan	NA	PRJEB27342
GCA 022231625	PDT000443953.1	Klebsiella_-	PDS000041826.3	nan	NA	PRJEB8666
GCA 022232945	PDT000443789.1	Klebsiella_-	PDS000041826.3	nan	NA	PRJEB8666
GCA 022231765	PDT000443935.1	Klebsiella_13	PDS000041826.3	nan	NA	PRJEB8666
GCA 022231685	PDT000443945.1	Klebsiella_13	PDS000041826.3	nan	NA	PRJEB8666
GCA 022029555	PDT000921113.1	Klebsiella_-	PDS000041826.3	nan	NA	PRJEB27342
GCA 022232325	PDT000443743.1	Klebsiella_-	PDS000041826.3	nan	NA	PRJEB8666
GCA 022029455	PDT000921118.1	Klebsiella_-	PDS000041826.3	nan	NA	PRJEB27342
GCA 022232285	PDT000443750.1	Klebsiella_13	PDS000041826.3	nan	NA	PRJEB8666
GCA 022232245	PDT000443758.1	Klebsiella_-	PDS000041826.3	nan	NA	PRJEB8666
GCA 022231705	PDT000443947.1	Klebsiella_13	PDS000041826.3	nan	NA	PRJEB8666
GCA 022231665	PDT000443950.1	Klebsiella_-	PDS000041826.3	nan	NA	PRJEB8666
GCA 022231725	PDT000443944.1	Klebsiella_-	PDS000041826.3	nan	NA	PRJEB8666
GCA 022029335	PDT000921123.1	Klebsiella_-	PDS000041826.3	nan	NA	PRJEB27342
GCA 022029515	PDT000921115.1	Klebsiella_-	PDS000041826.3	nan	NA	PRJEB27342
GCA 022028855	PDT000921126.1	Klebsiella_-	PDS000041826.3	nan	NA	PRJEB27342
GCA 022231545	PDT000443956.1	Klebsiella_-	PDS000041826.3	nan	NA	PRJEB8666
GCA 022232925	PDT000443784.1	Klebsiella_-	PDS000041826.3	nan	NA	PRJEB8666
GCA 022231585	PDT000443955.1	Klebsiella_-	PDS000041826.3	nan	NA	PRJEB8666
GCA 022029295	PDT000921124.1	Klebsiella_-	PDS000041826.3	nan	NA	PRJEB27342
GCA 022232145	PDT000443769.1	Klebsiella_13	PDS000041826.3	nan	NA	PRJEB8666
GCA 022231645	PDT000443952.1	Klebsiella_13	PDS000041826.3	nan	NA	PRJEB8666
GCA 022232225	PDT000443759.1	Klebsiella_13	PDS000041826.3	nan	NA	PRJEB8666
GCA 022232385	PDT000443731.1	Klebsiella_-	PDS000041826.3	nan	NA	PRJEB8666
GCA 022231885	PDT000443903.1	Klebsiella_-	PDS000041826.3	nan	NA	PRJEB8666
GCA 022232265	PDT000443751.1	Klebsiella_13	PDS000041826.3	nan	NA	PRJEB8666
GCA 022029375	PDT000921120.1	Klebsiella_13	PDS000041826.3	nan	NA	PRJEB27342
GCA 022232105	PDT000443779.1	Klebsiella_-	PDS000041826.3	nan	NA	PRJEB8666
GCA 022231745	PDT000443942.1	Klebsiella_-	PDS000041826.3	nan	NA	PRJEB8666

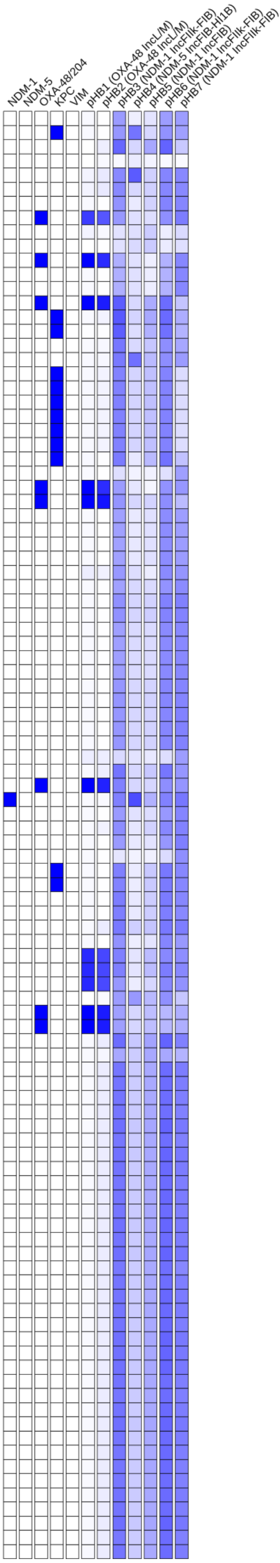

Tree 6: GCA_043859615

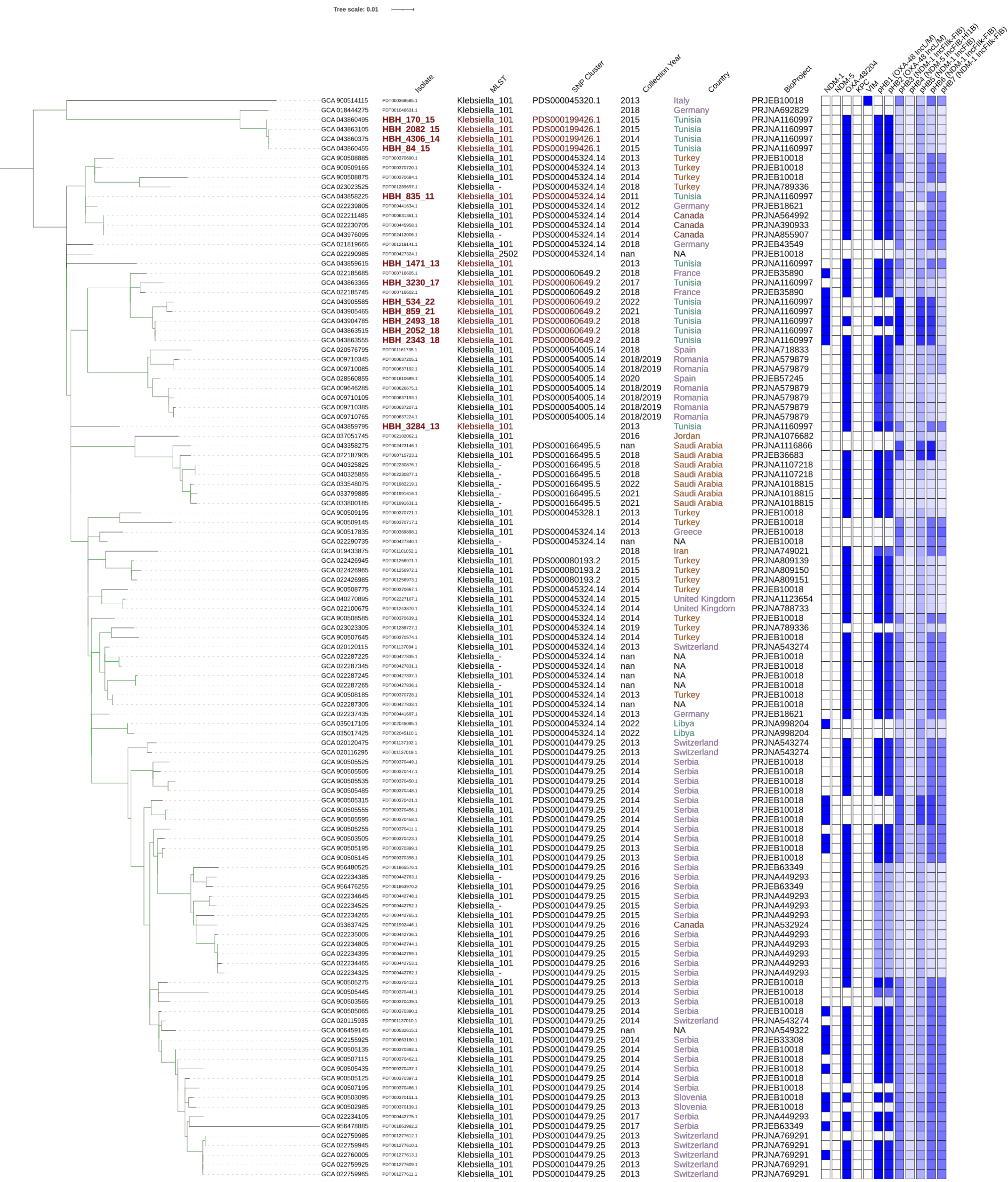

Tree 7: GCA_043859815

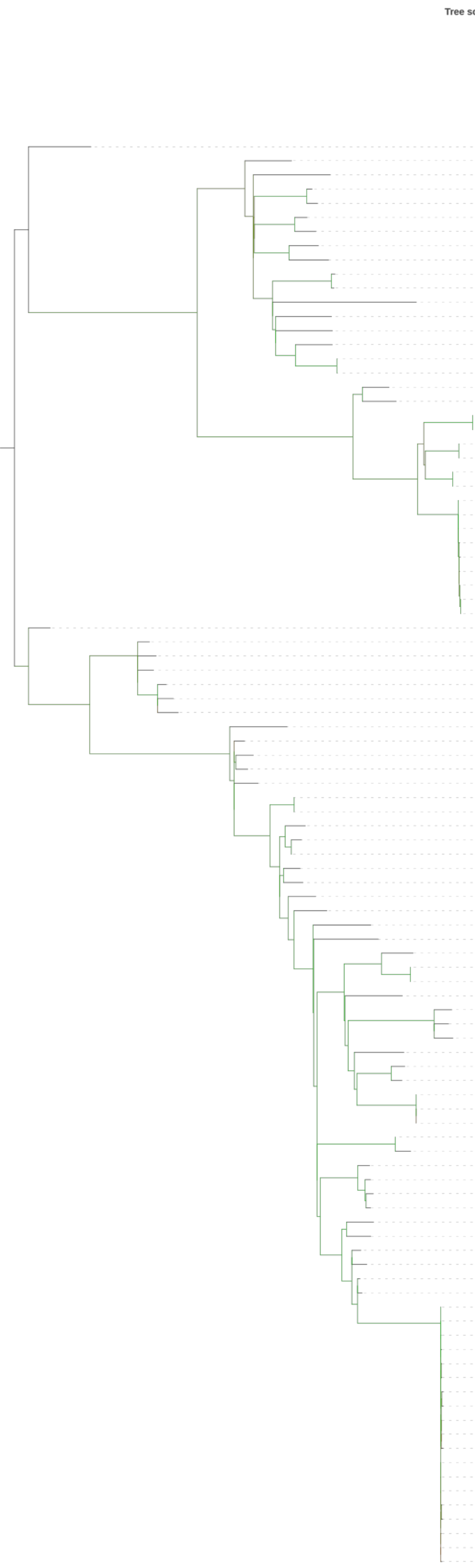

	Isolate	MLST	SNP Cluster	Collection Year	Country	BioProject
GCA 023743015	PDT001336675.1	Klebsiella_23		2018	China	PRJNA833902
GCA 040201615	PDT002208490.1	Klebsiella_23		nan	Saudi Arabia	PRJNA1116866
GCA 022305435	PDT000176176.2	Klebsiella_23		nan	NA	PRJEB5065
GCA 029913565	PDT001716423.1	Klebsiella_23	PDS000144603.1	nan	Singapore	PRJNA956314
GCA 029913845	PDT001716437.1	Klebsiella_23	PDS000144603.1	nan	Singapore	PRJNA956314
GCA 017743095	PDT001000586.1	Klebsiella_23		2020	China	PRJNA716566
GCA 034734155	PDT002042129.1	Klebsiella_23		2020	China	PRJNA1050895
GCA 022232625	PDT000443009.1	Klebsiella_23		2013	Vietnam	PRJEB26814
GCA 903815575	PDT000789034.1	Klebsiella_23		2011	France	PRJEB38788
GCA 021873475	PDT001198300.1	Klebsiella_23	PDS000100774.1	2017	Japan	PRJDB12075
GCA 021873455	PDT001198302.1	Klebsiella_23	PDS000100774.1	2017	Japan	PRJDB12075
GCA 900511245	PDT000369107.1	Klebsiella_23		2014	Austria	PRJEB10018
GCA 041204105	PDT002317893.1	Klebsiella_23		2019	France	PRJNA1132810
GCA 900502205	PDT000370110.1	Klebsiella_57		2014	Portugal	PRJEB10018
GCA 021876255	PDT001197704.1	Klebsiella_23		2017	South Africa	PRJEB46655
GCA 042208505	PDT002381737.1	Klebsiella_23	PDS000199499.1	2024	USA	PRJNA288601
GCA 042325345	PDT002393296.1	Klebsiella_23	PDS000199499.1	2024	USA	PRJNA288601
GCA 022401995	PDT001256417.1	Klebsiella_23		2019	China	PRJNA807910
GCA 019401465	PDT001099796.1	Klebsiella_23		2015	China	PRJNA595653
GCA 039847535	PDT002178478.1	Klebsiella_23	PDS000183496.1	2022	NA	PRJNA1113824
GCA 039608495	PDT002160565.1	Klebsiella_23	PDS000183496.1	2022	NA	PRJNA1100710
GCA 039609055	PDT002160593.1	Klebsiella_23	PDS000183493.1	2023	NA	PRJNA1100710
GCA 039847595	PDT002178481.1	Klebsiella_23	PDS000183493.1	2022	NA	PRJNA1113824
GCA 039610895	PDT002160685.1	Klebsiella_23	PDS000183478.1	2022	NA	PRJNA1100710
GCA 039847885	PDT002178494.1	Klebsiella_23	PDS000183478.1	2023	NA	PRJNA1113824
GCA 026162915	PDT001485882.1	Klebsiella_23	PDS000111758.3	2018	China	PRJNA885955
GCA 026163065	PDT001485890.1	Klebsiella_23	PDS000111758.3	2018	China	PRJNA885955
GCA 026162845	PDT001485879.1	Klebsiella_23	PDS000111758.3	2018	China	PRJNA885955
GCA 026163015	PDT001485888.1	Klebsiella_23	PDS000111758.3	2018	China	PRJNA885955
GCA 026163245	PDT001485899.1	Klebsiella_23	PDS000111758.3	2018	China	PRJNA885955
GCA 025920085	PDT001472184.1	Klebsiella_23	PDS000111758.3	2017	China	PRJNA438392
GCA 026163125	PDT001485893.1	Klebsiella_23	PDS000111758.3	2018	China	PRJNA885955
GCA 025920065	PDT001472183.1	Klebsiella_23	PDS000111758.3	2017	China	PRJNA438398
GCA 026163185	PDT001485896.1	Klebsiella_23	PDS000111758.3	2018	China	PRJNA885955
GCA 046464915	PDT002556470.1	Klebsiella_23		nan	NA	PRJNA786399
GCA 023062815	PDT001292621.1	Klebsiella_23	PDS000168937.2	2019	Belgium	PRJNA817214
GCA 046467675	PDT002556608.1	Klebsiella_23		nan	NA	PRJNA786399
GCA 046468375	PDT002556643.1	Klebsiella_23		nan	NA	PRJNA786399
GCA 046464975	PDT002556474.1	Klebsiella_23	PDS000168937.2	nan	NA	PRJNA786399
GCA 046467455	PDT002556597.1	Klebsiella_23	PDS000168937.2	nan	NA	PRJNA786399
GCA 034085985	PDT002017511.1	Klebsiella_23	PDS000168937.2	2012	China	PRJNA1015184
GCA 044740725	PDT002462499.1	Klebsiella_23		2019	China	PRJNA1165149
GCA 022402145	PDT001256426.1	Klebsiella_23		2019	China	PRJNA807890
GCA 026623545	PDT001512501.1	Klebsiella_23		2019	China	PRJNA905661
GCA 025891335	PDT001469207.1	Klebsiella_23	PDS000168933.1	2017	China	PRJNA791169
GCA 030020785	PDT001737463.1	Klebsiella_23		2022	United Kingdom	PRJNA957593
GCA 019841845	PDT001121604.1	Klebsiella_23	PDS000095240.1	2016	China	PRJNA757543
GCA 014283685	PDT000820996.1	Klebsiella_23	PDS000095240.1	2016	China	PRJNA657553
GCA 034682545	PDT001704498.1	Klebsiella_23		2020	Japan	PRJDB10842
GCA 023742675	PDT001336658.1	Klebsiella_23	PDS000111758.3	2019	China	PRJNA833902
GCA 014287045	PDT000822697.1	Klebsiella_23	PDS000111758.3	2007	China	PRJNA657553
GCA 034724775	PDT002041719.1	Klebsiella_23		2021	China	PRJNA1050895
GCA 046464695	PDT002556460.1	Klebsiella_23		nan	NA	PRJNA786399
GCA 030685615	PDT001846035.1	Klebsiella_23		2019	China	PRJNA1000265
GCA 902506975	PDT000662836.1	Klebsiella_23		2011	China	PRJEB34922
GCA 032285945	PDT001911319.1	Klebsiella_-		2022	USA	PRJNA978102
GCA 024429555	PDT001366428.1	Klebsiella_23		nan	USA	PRJNA825705
GCA 030587765	PDT001837966.1	Klebsiella_23		2017	South Korea	PRJNA521445
GCA 040539895	PDT002241116.1	Klebsiella_23	PDS000187849.1	2014	USA	PRJNA288601
GCA 030918245	PDT001865309.1	Klebsiella_23	PDS000187849.1	nan	USA	PRJNA994967
GCA 022231185	PDT000444146.1	Klebsiella_23		nan	NA	PRJEB8666
GCA 034408505	PDT002029039.1	Klebsiella_23		2017	Vietnam	PRJEB67696
GCA 029914525	PDT001716470.1	Klebsiella_23		nan	Singapore	PRJNA956314
GCA 022233885	PDT000442953.1	Klebsiella_23	PDS000164559.2	2012	Vietnam	PRJEB26814
GCA 043859815	**HBH_3084_13**	Klebsiella_23		2013	Tunisia	PRJNA1160997
GCA 040200575	PDT002208438.1	Klebsiella_23		nan	Saudi Arabia	PRJNA1116866
GCA 040190295	PDT002208157.1	Klebsiella_23		nan	Saudi Arabia	PRJNA1116866
GCA 044321445	PDT002311013.1	Klebsiella_23	PDS000192486.2	2021	Egypt	PRJEB76311
GCA 044321425	PDT002311012.1	Klebsiella_23	PDS000192486.2	2021	Egypt	PRJEB76311
GCA 044049895	PDT002327654.1	Klebsiella_23	PDS000192486.2	2021	Egypt	PRJEB76311
GCA 032075745	PDT001904972.1	Klebsiella_23	PDS000160830.3	2017	Norway	PRJEB48268
GCA 041902785	PDT002381572.1	Klebsiella_23	PDS000160830.3	2017	Norway	PRJNA1098507
GCA 022249935	PDT000440693.1	Klebsiella_23		nan	NA	PRJEB21605
GCA 022249275	PDT000440741.1	Klebsiella_23	PDS000045398.1	nan	NA	PRJEB21605
GCA 022249915	PDT000440694.1	Klebsiella_23	PDS000045398.1	nan	NA	PRJEB21605
GCA 022249315	PDT000440736.1	Klebsiella_23	PDS000045398.1	nan	NA	PRJEB21605
GCA 032076785	PDT001904941.1	Klebsiella_23		2018	Norway	PRJEB48268
GCA 022186865	PDT000715850.1	Klebsiella_23	PDS000060603.1	2018	Saudi Arabia	PRJEB36683
GCA 029911805	PDT001716354.1	Klebsiella_23	PDS000111295.2	nan	Singapore	PRJNA956314
GCA 036995585	PDT002098819.1	Klebsiella_23		2017	China	PRJNA1003408
GCA 029913185	PDT001716404.1	Klebsiella_23	PDS000111295.2	nan	Singapore	PRJNA956314
GCA 029913385	PDT001716414.1	Klebsiella_23	PDS000111295.2	nan	Singapore	PRJNA956314
GCA 023780405	PDT001325481.1	Klebsiella_23	PDS000111295.2	nan	Singapore	PRJNA801425
GCA 023781735	PDT001325485.1	Klebsiella_23	PDS000111295.2	nan	Singapore	PRJNA801425
GCA 023777335	PDT001325487.1	Klebsiella_23	PDS000111295.2	nan	Singapore	PRJNA801425
GCA 023825055	PDT001325489.1	Klebsiella_23	PDS000111295.2	nan	Singapore	PRJNA801425
GCA 023825415	PDT001325486.1	Klebsiella_23	PDS000111295.2	nan	Singapore	PRJNA801425
GCA 023779685	PDT001325484.1	Klebsiella_23	PDS000111295.2	nan	Singapore	PRJNA801425
GCA 023874345	PDT001325482.1	Klebsiella_23	PDS000111295.2	nan	Singapore	PRJNA801425
GCA 023874325	PDT001325501.1	Klebsiella_23	PDS000111295.2	nan	Singapore	PRJNA801425
GCA 023824995	PDT001325499.1	Klebsiella_23	PDS000111295.2	nan	Singapore	PRJNA801425
GCA 023825015	PDT001325494.1	Klebsiella_23	PDS000111295.2	nan	Singapore	PRJNA801425
GCA 023781755	PDT001325495.1	Klebsiella_23	PDS000111295.2	nan	Singapore	PRJNA801425
GCA 023781715	PDT001325491.1	Klebsiella_23	PDS000111295.2	nan	Singapore	PRJNA801425
GCA 002813595	PDT000270620.1	Klebsiella_23	PDS000111295.2	nan	Singapore	PRJNA391004
GCA 023776515	PDT001325498.1	Klebsiella_23	PDS000111295.2	nan	Singapore	PRJNA801425
GCA 023825375	PDT001325492.1	Klebsiella_23	PDS000111295.2	nan	Singapore	PRJNA801425
GCA 023775075	PDT001325493.1	Klebsiella_23	PDS000111295.2	nan	Singapore	PRJNA801425
GCA 023826515	PDT001325500.1	Klebsiella_23	PDS000111295.2	nan	Singapore	PRJNA801425
GCA 023826075	PDT001325496.1	Klebsiella_23	PDS000111295.2	nan	Singapore	PRJNA801425
GCA 023874285	PDT001325497.1	Klebsiella_23	PDS000111295.2	nan	Singapore	PRJNA801425

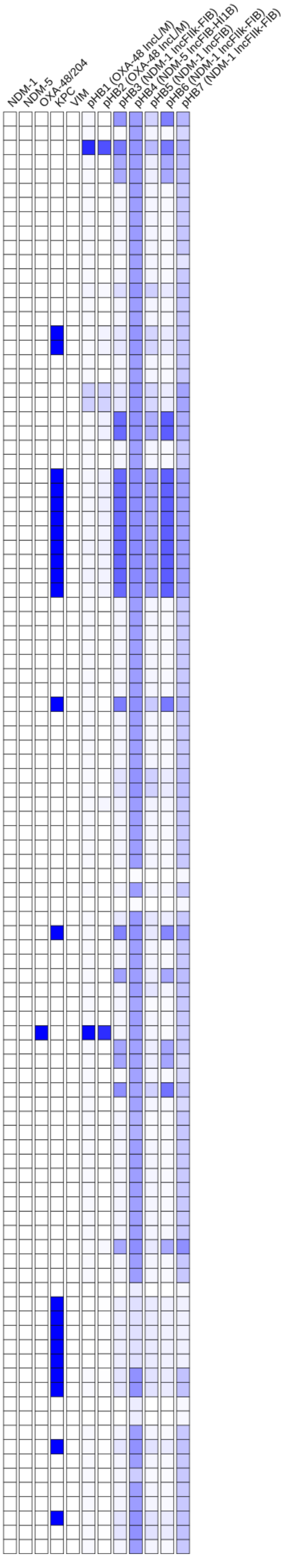

Tree 8: GCA_043859855

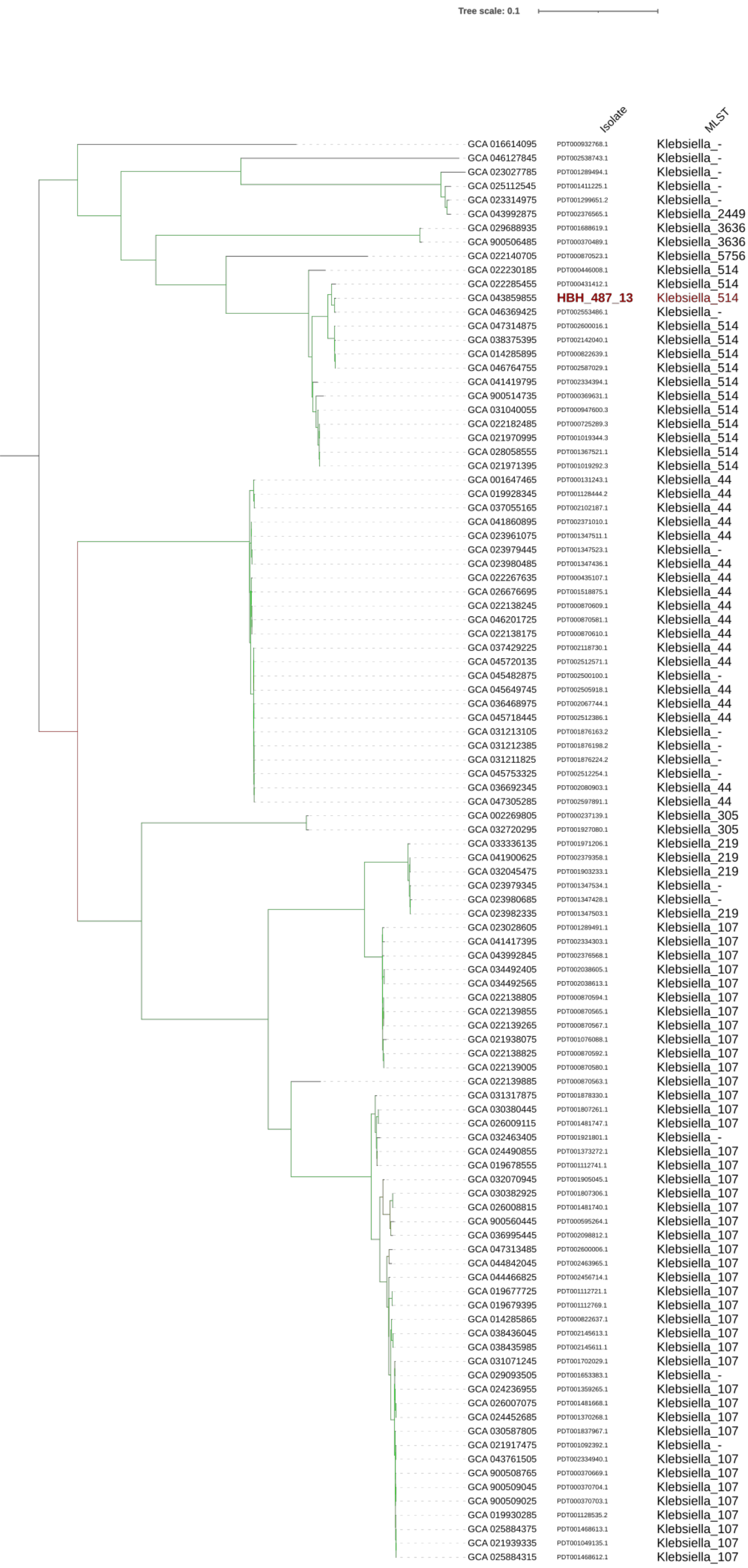

	Isolate	MLST	SNP Cluster	Collection Year	Country	BioProject
GCA 016614095	PDT000932768.1	Klebsiella_-		nan	NA	PRJNA550041
GCA 046127845	PDT002538743.1	Klebsiella_-		2019	New Zealand	PRJNA1102395
GCA 023027785	PDT001289494.1	Klebsiella_-		2020	Australia	PRJNA777643
GCA 025112545	PDT001411225.1	Klebsiella_-		2019	Singapore	PRJNA577535
GCA 023314975	PDT001299651.2	Klebsiella_-		2018	USA	PRJNA789565
GCA 043992875	PDT002376565.1	Klebsiella_2449		2016	Japan	PRJDB14656
GCA 029688935	PDT001688619.1	Klebsiella_3636		2018	USA	PRJNA684628
GCA 900506485	PDT000370489.1	Klebsiella_3636		2013	Belgium	PRJEB10018
GCA 022140705	PDT000870523.1	Klebsiella_5756		2017	USA	PRJNA594602
GCA 022230185	PDT000446008.1	Klebsiella_514		2012	Canada	PRJNA390933
GCA 022285455	PDT000431412.1	Klebsiella_514		2017	USA	PRJNA396774
GCA 043859855	**HBH_487_13**	Klebsiella_514		2013	Tunisia	PRJNA1160997
GCA 046369425	PDT002553486.1	Klebsiella_-		2024	China	PRJNA1101749
GCA 047314875	PDT002600016.1	Klebsiella_514		2023	China	PRJNA1213349
GCA 038375395	PDT002142040.1	Klebsiella_514		2022	China	PRJNA1081623
GCA 014285895	PDT000822639.1	Klebsiella_514	PDS000215654.1	2014	China	PRJNA657553
GCA 046764755	PDT002587029.1	Klebsiella_514	PDS000215654.1	2014	China	PRJNA1210003
GCA 041419795	PDT002334394.1	Klebsiella_514		2018	China	PRJNA1142693
GCA 900514735	PDT000369631.1	Klebsiella_514		2013	Italy	PRJEB10018
GCA 031040055	PDT000947600.3	Klebsiella_514	PDS000148766.7	2020	USA	PRJNA288601
GCA 022182485	PDT000725289.3	Klebsiella_514	PDS000148766.7	2019	USA	PRJNA288601
GCA 021970995	PDT001019344.3	Klebsiella_514	PDS000148766.7	2021	USA	PRJNA288601
GCA 028058555	PDT001367521.1	Klebsiella_514	PDS000148766.7	2022	USA	PRJNA288601
GCA 021971395	PDT001019292.3	Klebsiella_514	PDS000148766.7	2021	USA	PRJNA288601
GCA 001647465	PDT000131243.1	Klebsiella_44		2008	USA	PRJNA261239
GCA 019928345	PDT001128444.2	Klebsiella_44	PDS000176509.1	2003	Germany	PRJNA717739
GCA 037055165	PDT002102187.1	Klebsiella_44	PDS000176509.1	2012	Jordan	PRJNA1076682
GCA 041860895	PDT002371010.1	Klebsiella_44	PDS000112610.2	2019	Norway	PRJNA835677
GCA 023961075	PDT001347511.1	Klebsiella_44	PDS000112610.2	2019	Norway	PRJNA835677
GCA 023979445	PDT001347523.1	Klebsiella_-	PDS000112610.2	2019	Norway	PRJNA835677
GCA 023980485	PDT001347436.1	Klebsiella_44	PDS000112610.2	2019	Norway	PRJNA835677
GCA 022267635	PDT000435107.1	Klebsiella_44		2012	USA	PRJNA289272
GCA 026676695	PDT001518875.1	Klebsiella_44		2022	Ghana	PRJNA906096
GCA 022138245	PDT000870609.1	Klebsiella_44	PDS000074438.1	2015	USA	PRJNA594602
GCA 046201725	PDT000870581.1	Klebsiella_44	PDS000074438.1	2015	USA	PRJNA594602
GCA 022138175	PDT000870610.1	Klebsiella_44	PDS000074438.1	2015	USA	PRJNA594602
GCA 037429225	PDT002118730.1	Klebsiella_44	PDS000157999.11	2024	USA	PRJNA288601
GCA 045720135	PDT002512571.1	Klebsiella_44	PDS000157999.11	2024	USA	PRJNA288601
GCA 045482875	PDT002500100.1	Klebsiella_-	PDS000157999.11	2024	USA	PRJNA288601
GCA 045649745	PDT002505918.1	Klebsiella_44	PDS000157999.11	2024	USA	PRJNA288601
GCA 036468975	PDT002067744.1	Klebsiella_44	PDS000157999.11	2023	USA	PRJNA288601
GCA 045718445	PDT002512386.1	Klebsiella_44	PDS000157999.11	2024	USA	PRJNA288601
GCA 031213105	PDT001876163.2	Klebsiella_-	PDS000157999.11	2023	USA	PRJNA288601
GCA 031212385	PDT001876198.2	Klebsiella_-	PDS000157999.11	2023	USA	PRJNA288601
GCA 031211825	PDT001876224.2	Klebsiella_-	PDS000157999.11	2023	USA	PRJNA288601
GCA 045753325	PDT002512254.1	Klebsiella_-	PDS000157999.11	2024	USA	PRJNA288601
GCA 036692345	PDT002080903.1	Klebsiella_44	PDS000157999.11	2023	USA	PRJNA288601
GCA 047305285	PDT002597891.1	Klebsiella_44	PDS000157999.11	2024	USA	PRJNA288601
GCA 002269805	PDT000237139.1	Klebsiella_305		2015	China	PRJNA388518
GCA 032720295	PDT001927080.1	Klebsiella_305		2020	Italy	PRJNA1026096
GCA 033336135	PDT001971206.1	Klebsiella_219		2021	Uganda	PRJNA1015582
GCA 041900625	PDT002379358.1	Klebsiella_219	PDS000198446.1	2018	Norway	PRJNA1098507
GCA 032045475	PDT001903233.1	Klebsiella_219	PDS000198446.1	2018	Norway	PRJEB48268
GCA 023979345	PDT001347534.1	Klebsiella_-		2019	Norway	PRJNA835677
GCA 023980685	PDT001347428.1	Klebsiella_-		2019	Norway	PRJNA835677
GCA 023982335	PDT001347503.1	Klebsiella_219	PDS000112630.1	2019	Norway	PRJNA835677
GCA 023028605	PDT001289491.1	Klebsiella_107		2020	Australia	PRJNA777643
GCA 041417395	PDT002334303.1	Klebsiella_107		2019	China	PRJNA1142693
GCA 043992845	PDT002376568.1	Klebsiella_107		2016	Japan	PRJDB14656
GCA 034492405	PDT002038605.1	Klebsiella_107	PDS000170612.1	2019	France	PRJNA876630
GCA 034492565	PDT002038613.1	Klebsiella_107	PDS000170612.1	2019	France	PRJNA876630
GCA 022138805	PDT000870594.1	Klebsiella_107	PDS000074448.1	2015	USA	PRJNA594602
GCA 022139855	PDT000870565.1	Klebsiella_107	PDS000074448.1	2016	USA	PRJNA594602
GCA 022139265	PDT000870567.1	Klebsiella_107	PDS000074448.1	2016	USA	PRJNA594602
GCA 021938075	PDT001076088.1	Klebsiella_107		2015	Netherlands	PRJEB44175
GCA 022138825	PDT000870592.1	Klebsiella_107	PDS000074449.1	2015	USA	PRJNA594602
GCA 022139005	PDT000870580.1	Klebsiella_107	PDS000074449.1	2015	USA	PRJNA594602
GCA 022139885	PDT000870563.1	Klebsiella_107		2015	USA	PRJNA594602
GCA 031317875	PDT001878330.1	Klebsiella_107		2018	China	PRJNA914894
GCA 030380445	PDT001807261.1	Klebsiella_107	PDS000150194.3	2009	Japan	PRJNA984451
GCA 026009115	PDT001481747.1	Klebsiella_107	PDS000150194.3	2009	Japan	PRJDB13833
GCA 032463405	PDT001921801.1	Klebsiella_-		2021	USA	PRJNA800017
GCA 024490855	PDT001373272.1	Klebsiella_107		2019	Spain	PRJEB50822
GCA 019678555	PDT001112741.1	Klebsiella_107		2016	Germany	PRJNA427504
GCA 032070945	PDT001905045.1	Klebsiella_107		2018	Norway	PRJEB48268
GCA 030382925	PDT001807306.1	Klebsiella_107	PDS000150182.3	2005	Japan	PRJNA984451
GCA 026008815	PDT001481740.1	Klebsiella_107	PDS000150182.3	2005	Japan	PRJDB13833
GCA 900560445	PDT000595264.1	Klebsiella_107		nan	United Kingdom	PRJEB28457
GCA 036995445	PDT002098812.1	Klebsiella_107		2015	China	PRJNA1003408
GCA 047313485	PDT002600006.1	Klebsiella_107		2021	Spain	PRJNA1141252
GCA 044842045	PDT002463965.1	Klebsiella_107		2022	USA	PRJNA1127607
GCA 044466825	PDT002456714.1	Klebsiella_107		2022	Armenia	PRJNA1141898
GCA 019677725	PDT001112721.1	Klebsiella_107		2016	Germany	PRJNA395964
GCA 019679395	PDT001112769.1	Klebsiella_107		2016	Germany	PRJNA488925
GCA 014285865	PDT000822637.1	Klebsiella_107		2012	China	PRJNA657553
GCA 038436045	PDT002145613.1	Klebsiella_107	PDS000181291.1	2021	China	PRJNA945814
GCA 038435985	PDT002145611.1	Klebsiella_107	PDS000181291.1	2021	China	PRJNA945814
GCA 031071245	PDT001702029.1	Klebsiella_107		2019	Japan	PRJDB10842
GCA 029093505	PDT001653383.1	Klebsiella_-		2019	China	PRJNA796569
GCA 024236955	PDT001359265.1	Klebsiella_107	PDS000114410.2	2017	China	PRJNA833746
GCA 026007075	PDT001481668.1	Klebsiella_107	PDS000114410.2	2013	Japan	PRJDB12116
GCA 024452685	PDT001370268.1	Klebsiella_107	PDS000114410.2	2013	Japan	PRJDB12116
GCA 030587805	PDT001837967.1	Klebsiella_107		2016	South Korea	PRJNA521445
GCA 021917475	PDT001092392.1	Klebsiella_-	PDS000036289.4	2014	Norway	PRJEB27256
GCA 043761505	PDT002334940.1	Klebsiella_107	PDS000036289.4	2013	Netherlands	PRJEB78530
GCA 900508765	PDT000370669.1	Klebsiella_107	PDS000036289.4	2013	Turkey	PRJEB10018
GCA 900509045	PDT000370704.1	Klebsiella_107	PDS000036289.4	2013	Turkey	PRJEB10018
GCA 900509025	PDT000370703.1	Klebsiella_107	PDS000036289.4	2013	Turkey	PRJEB10018
GCA 019930285	PDT001128535.2	Klebsiella_107	PDS000036289.4	2011	Turkey	PRJNA717739
GCA 025884375	PDT001468613.1	Klebsiella_107	PDS000121675.1	2017	Australia	PRJNA857533
GCA 021939335	PDT001049135.1	Klebsiella_107	PDS000121675.1	2017	Australia	PRJNA857533
GCA 025884315	PDT001468612.1	Klebsiella_107	PDS000121675.1	2017	Australia	PRJNA857533

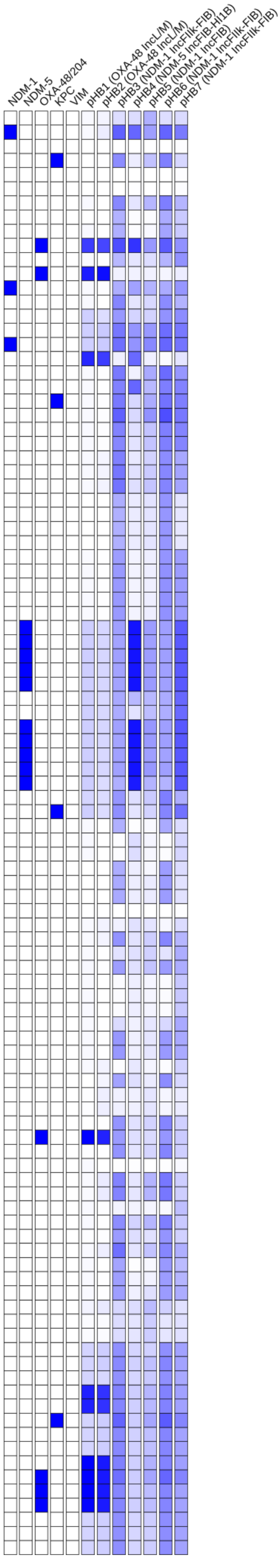

Tree 9: GCA_043859995

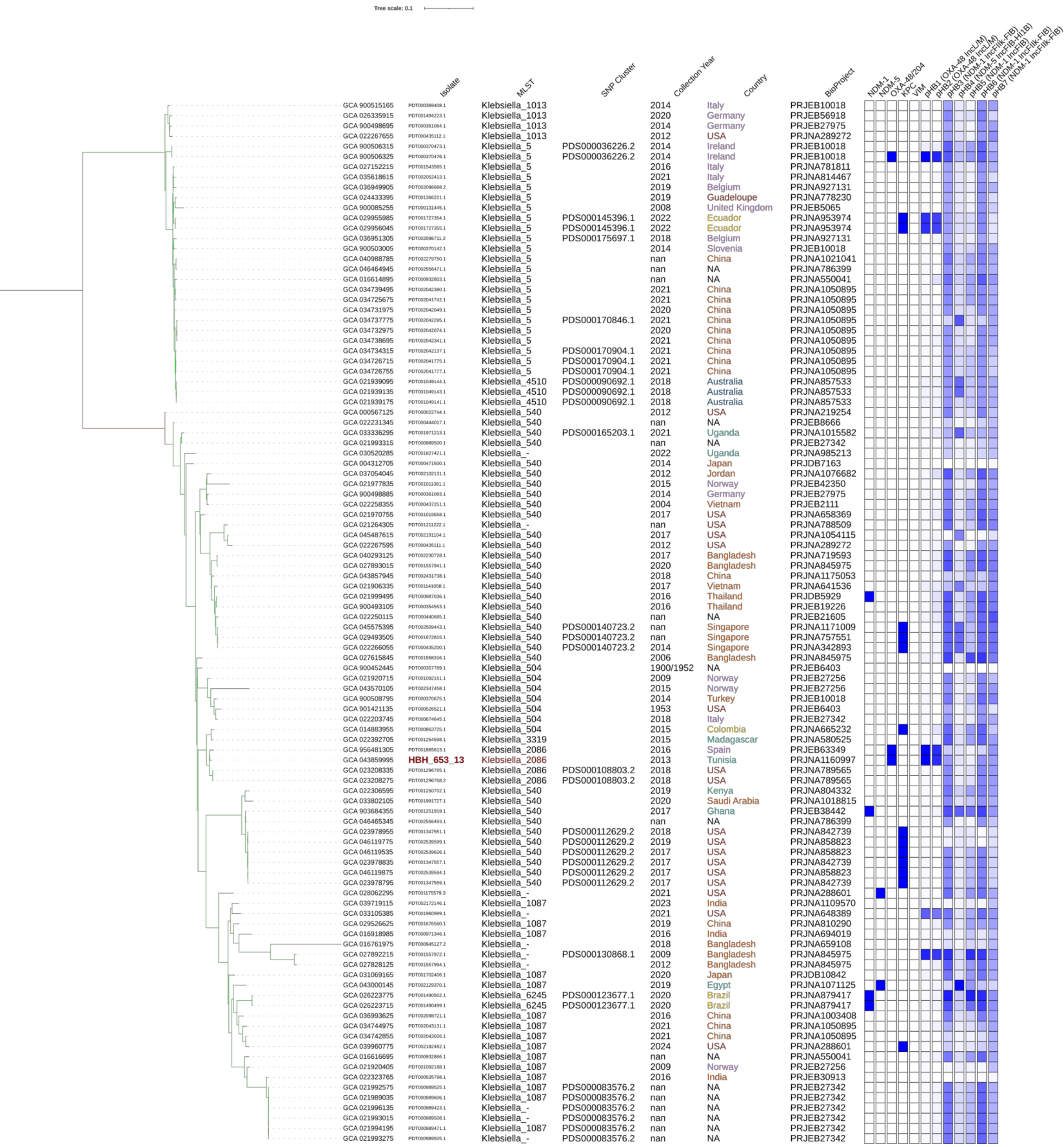

Tree 10: GCA_043860015

Tree scale: 0.1

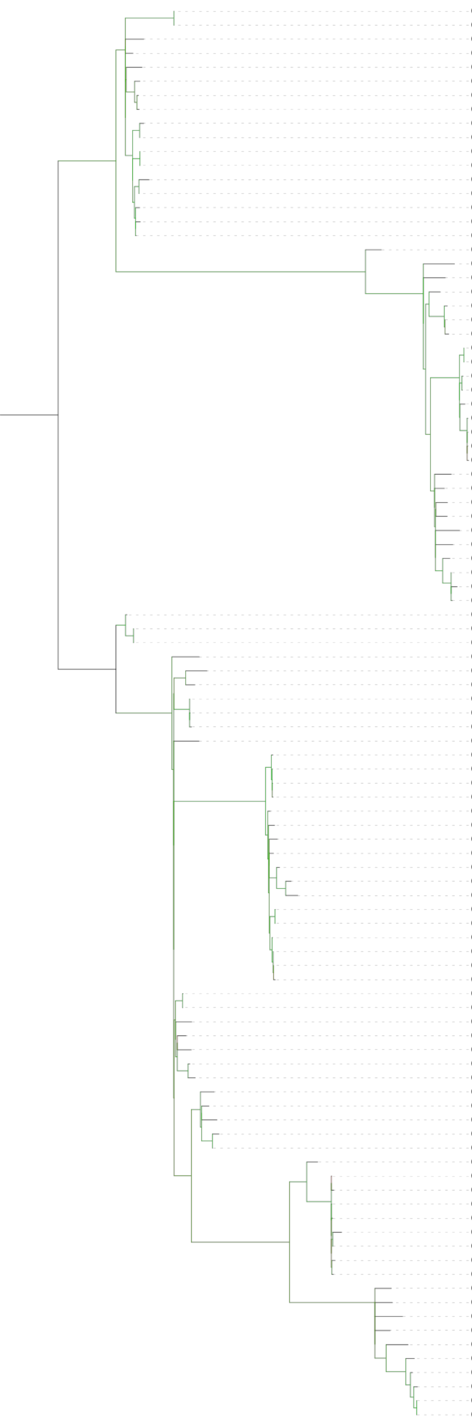

	Isolate	MLST	SNP Cluster	Collection Year	Country	BioProject
GCA 022170145	PDT000814187.1	Klebsiella_147	PDS000067059.3	2020	USA	PRJNA288601
GCA 022170185	PDT000814186.3	Klebsiella_147	PDS000067059.3	2020	USA	PRJNA288601
GCA 016136185	PDT000920642.1	Klebsiella_147	PDS000040466.3	2015	Russia	PRJNA525009
GCA 028883395	PDT001647978.1	Klebsiella_147		nan	Algeria	PRJNA915079
GCA 022107435	PDT001244777.1	Klebsiella_147		2014	United Kingdom	PRJNA788733
GCA 024609295	PDT001385475.1	Klebsiella_147		2016	Tunisia	PRJNA841857
GCA 900507565	PDT000370563.1	Klebsiella_147	PDS000104546.2	2014	Belgium	PRJEB10018
GCA 043860015	**HBH_1123_14**	Klebsiella_147	PDS000104546.2	2014	Tunisia	PRJNA1160997
GCA 916162535	PDT001141267.1	Klebsiella_147	PDS000096678.1	2015	Netherlands	PRJEB41042
GCA 916164145	PDT001141310.1	Klebsiella_147	PDS000096678.1	2010	Netherlands	PRJEB41042
GCA 008693405	PDT000594165.1	Klebsiella_147	PDS000076259.2	nan	USA	PRJNA231221
GCA 022042835	PDT000892792.3	Klebsiella_147	PDS000076259.2	2015	USA	PRJNA288601
GCA 900504755	PDT000370189.1	Klebsiella_147	PDS000036185.10	2014	Romania	PRJEB10018
GCA 900504855	PDT000370176.1	Klebsiella_147	PDS000036185.10	2014	Romania	PRJEB10018
GCA 900504225	PDT000370263.1	Klebsiella_147	PDS000036185.10	2014	Romania	PRJEB10018
GCA 900504685	PDT000370199.1	Klebsiella_147	PDS000036185.10	2014	Romania	PRJEB10018
GCA 900504585	PDT000370192.1	Klebsiella_147	PDS000036185.10	2014	Romania	PRJEB10018
GCA 900512155	PDT000369490.2	Klebsiella_147		2014	Israel	PRJEB10018
GCA 012971605	PDT000730631.1	Klebsiella_147		2016	South Korea	PRJNA625837
GCA 033550765	PDT001979154.1	Klebsiella_147	PDS000165990.1	2017	Germany	PRJEB67440
GCA 022306355	PDT000176139.2	Klebsiella_147	PDS000006642.4	nan	NA	PRJEB5065
GCA 043625685	PDT002332973.1	Klebsiella_147	PDS000099727.3	nan	Spain	PRJEB55414
GCA 043625465	PDT002332979.1	Klebsiella_147	PDS000099727.3	nan	Spain	PRJEB55414
GCA 900517095	PDT000369887.1	Klebsiella_147	PDS000099727.3	2014	Spain	PRJEB10018
GCA 022001215	PDT000987012.1	Klebsiella_147	PDS000083378.2	2016	Thailand	PRJDB5929
GCA 022001185	PDT000987015.1	Klebsiella_147	PDS000083378.2	2016	Thailand	PRJDB5929
GCA 021983395	PDT001003375.1	Klebsiella_147	PDS000083378.2	2016	Thailand	PRJDB5929
GCA 022282305	PDT000431960.1	Klebsiella_147	PDS000083378.2	2016	Thailand	PRJDB5929
GCA 022005405	PDT000986943.1	Klebsiella_147	PDS000083378.2	2015	Thailand	PRJDB5929
GCA 021982475	PDT001008034.1	Klebsiella_147	PDS000084451.3	2015	Thailand	PRJDB5929
GCA 021987055	PDT001003115.1	Klebsiella_147	PDS000084451.3	2015	Thailand	PRJDB5929
GCA 021985595	PDT001003215.1	Klebsiella_147	PDS000084451.3	2015	Thailand	PRJDB5929
GCA 021985635	PDT001003212.1	Klebsiella_147	PDS000084451.3	2015	Thailand	PRJDB5929
GCA 900517045	PDT000369761.1	Klebsiella_147		2013	Hungary	PRJEB10018
GCA 900510435	PDT000369189.1	Klebsiella_147		2014	France	PRJEB10018
GCA 022246125	PDT000441036.1	Klebsiella_-		nan	NA	PRJEB12699
GCA 022246065	PDT000441038.1	Klebsiella_147		nan	NA	PRJEB12699
GCA 022293415	PDT000423788.1	Klebsiella_147	PDS000052092.1	nan	NA	PRJEB17615
GCA 022212825	PDT000628215.1	Klebsiella_147		2008	Canada	PRJNA564992
GCA 041003655	PDT002281177.1	Klebsiella_147		2021	China	PRJNA1135755
GCA 021815755	PDT001219543.1	Klebsiella_147	PDS000006578.119	2011	Canada	PRJNA725227
GCA 022230785	PDT000445955.1	Klebsiella_147	PDS000006578.119	2013	Canada	PRJNA390933
GCA 025116845	PDT001411104.1	Klebsiella_147	PDS000006578.119	2011	Singapore	PRJNA577535
GCA 040588405	PDT002245963.1	Klebsiella_147	PDS000187661.2	2024	USA	PRJNA288601
GCA 039648715	PDT002165226.1	Klebsiella_147	PDS000187661.2	2024	USA	PRJNA288601
GCA 040500635	PDT002239188.1	Klebsiella_147	PDS000187661.2	2024	USA	PRJNA288601
GCA 022030675	PDT000921062.1	Klebsiella_-		nan	NA	PRJEB27342
GCA 002189175	PDT000217885.1	Klebsiella_147		2016	Tunisia	PRJNA360256
GCA 022121525	PDT001244814.1	Klebsiella_147	PDS000104546.2	2015	United Kingdom	PRJNA788733
GCA 028645895	PDT001616162.1	Klebsiella_147	PDS000136742.4	2022	USA	PRJNA288601
GCA 027102235	PDT001537712.3	Klebsiella_147	PDS000136742.4	2022	USA	PRJNA288601
GCA 039665215	PDT002168329.1	Klebsiella_147	PDS000136742.4	2024	USA	PRJNA288601
GCA 033799285	PDT001991586.1	Klebsiella_147		2021	Saudi Arabia	PRJNA1018815
GCA 031036015	PDT001726355.3	Klebsiella_147	PDS000052089.184	2018	USA	PRJNA288601
GCA 043448245	PDT002421413.1	Klebsiella_147	PDS000052089.184	2019	USA	PRJNA288601
GCA 043442185	PDT002421417.1	Klebsiella_147	PDS000052089.184	2019	USA	PRJNA288601
GCA 043458055	PDT002421633.1	Klebsiella_147	PDS000052089.184	2019	USA	PRJNA288601
GCA 022282545	PDT000431951.1	Klebsiella_147	PDS000156049.11	2016	Thailand	PRJDB5929
GCA 900502585	PDT000370025.1	Klebsiella_147	PDS000156049.11	2014	Denmark	PRJEB10018
GCA 022276245	PDT000432066.1	Klebsiella_147	PDS000156049.11	2015	Myanmar	PRJDB5126
GCA 021875215	PDT001197766.1	Klebsiella_147	PDS000156049.11	2013	Singapore	PRJNA577535
GCA 004803235	PDT000495887.1	Klebsiella_147	PDS000077015.34	nan	Egypt	PRJNA508509
GCA 022353505	PDT001118281.2	Klebsiella_147	PDS000077015.34	2019	Italy	PRJNA643814
GCA 021944635	PDT001042146.1	Klebsiella_147	PDS000077015.34	2019	Italy	PRJNA725484
GCA 002202215	PDT000219969.1	Klebsiella_147	PDS000156049.11	nan	NA	PRJNA292902
GCA 002202235	PDT000219970.1	Klebsiella_147	PDS000156049.11	nan	NA	PRJNA292902
GCA 021944495	PDT001042155.1	Klebsiella_147	PDS000156049.11	nan	USA	PRJNA725484
GCA 021943235	PDT001042219.1	Klebsiella_147	PDS000156049.11	nan	USA	PRJNA725484
GCA 021943995	PDT001042177.1	Klebsiella_147	PDS000156049.11	nan	USA	PRJNA725484
GCA 021943595	PDT001042197.1	Klebsiella_147	PDS000156049.11	nan	USA	PRJNA725484
GCA 022237855	PDT000441689.1	Klebsiella_147	PDS000041784.1	2010	Germany	PRJEB18621
GCA 022237945	PDT000441688.1	Klebsiella_147	PDS000041784.1	2009	Germany	PRJEB18621
GCA 022985765	PDT001286509.1	Klebsiella_147		2017	Germany	PRJNA789142
GCA 022275685	PDT000432630.1	Klebsiella_147		2008	Ireland	PRJNA396729
GCA 022211765	PDT000630651.1	Klebsiella_147		2012	Canada	PRJNA564992
GCA 022188405	PDT000715641.1	Klebsiella_147	PDS000060612.1	2018	Saudi Arabia	PRJEB36683
GCA 022187725	PDT000715781.1	Klebsiella_147	PDS000060612.1	2018	Saudi Arabia	PRJEB36683
GCA 022274495	PDT000433141.1	Klebsiella_147	PDS000040466.3	2015	Russia	PRJNA429779
GCA 002263345	PDT000236085.1	Klebsiella_147		2016	NA	PRJNA269675
GCA 002263375	PDT000236086.1	Klebsiella_147		2016	NA	PRJNA269675
GCA 024023475	PDT001336816.1	Klebsiella_147	PDS000111778.2	2018	Israel	PRJEB53419
GCA 023924585	PDT001345509.1	Klebsiella_147	PDS000111778.2	2018	Israel	PRJEB53419
GCA 043831025	PDT002430489.1	Klebsiella_147		2019	Germany	PRJNA1070536
GCA 045999065	PDT002521531.1	Klebsiella_147	PDS000129321.8	2022	Ukraine	PRJNA1102281
GCA 031018605	PDT001779621.1	Klebsiella_147	PDS000129321.8	2023	USA	PRJNA288601
GCA 043906565	PDT002391370.1	Klebsiella_147	PDS000129321.8	2023	Netherlands	PRJNA1143178
GCA 027131555	PDT001538049.1	Klebsiella_147	PDS000129321.8	2022	Netherlands	PRJNA903550
GCA 947577115	PDT001769437.1	Klebsiella_147	PDS000129321.8	2022	Germany	PRJEB58018
GCA 947576795	PDT001769460.1	Klebsiella_147	PDS000129321.8	2022	Germany	PRJEB58018
GCA 042917305	PDT002097668.1	Klebsiella_147	PDS000129321.8	2023	Netherlands	PRJNA1076808
GCA 033585375	PDT001981974.1	Klebsiella_147	PDS000129321.8	2022	Netherlands	PRJNA903550
GCA 016635355	PDT000932944.1	Klebsiella_147		2015	Australia	PRJNA480723
GCA 900507055	PDT000370555.1	Klebsiella_147		2014	Belgium	PRJEB10018
GCA 029224285	PDT001663092.1	Klebsiella_147		2019	China	PRJNA838419
GCA 022182885	PDT000724039.1	Klebsiella_147		2018	Italy	PRJEB27342
GCA 027904235	PDT001570537.1	Klebsiella_147	PDS000132251.1	nan	Spain	PRJNA918858
GCA 027610945	PDT001558347.1	Klebsiella_147	PDS000130855.1	2007	Bangladesh	PRJNA845975
GCA 027615665	PDT001558328.1	Klebsiella_147	PDS000009779.14	2006	Bangladesh	PRJNA845975
GCA 900511365	PDT000369134.1	Klebsiella_147	PDS000009779.14	2014	Poland	PRJEB10018
GCA 022320785	PDT001251452.1	Klebsiella_147	PDS000009779.14	nan	United Kingdom	PRJNA787440
GCA 034651715	PDT002040257.1	Klebsiella_147	PDS000009779.14	nan	NA	PRJNA1017778

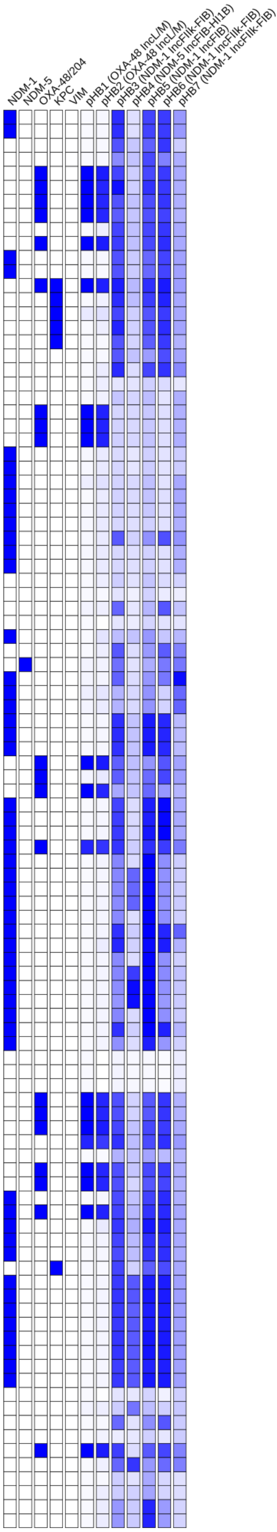

Tree 11: GCA_043860075

Tree scale: 0.01

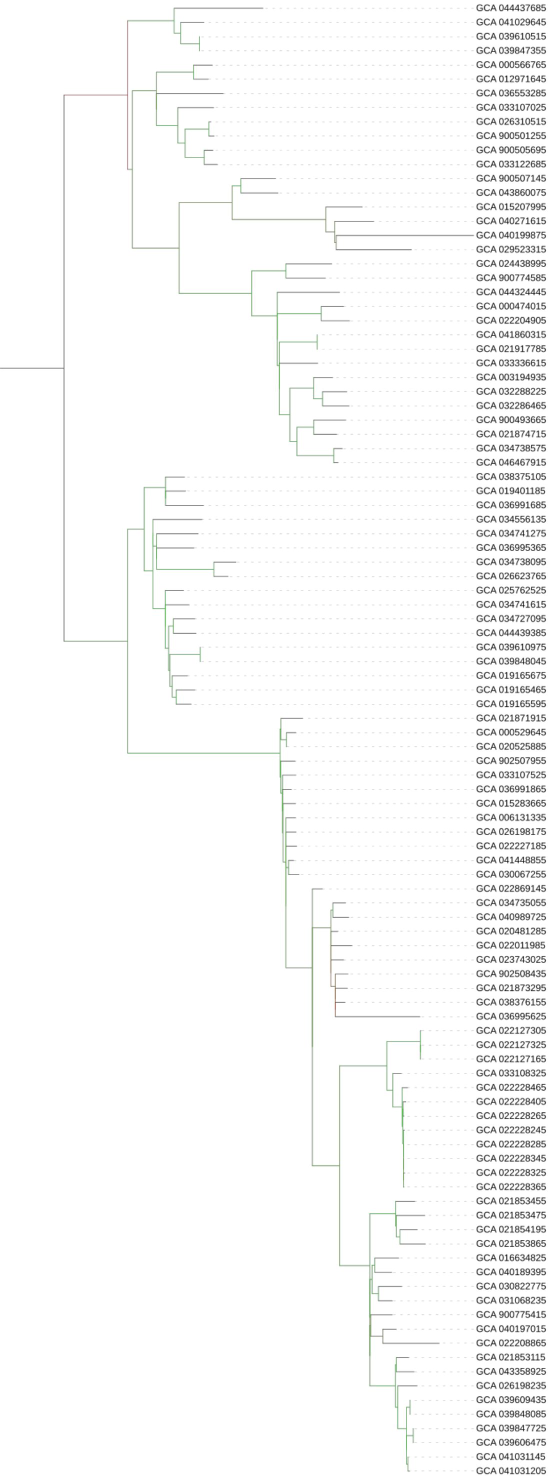

	Isolate	MLST	SNP Cluster	Collection Year	Country	BioProject
GCA 044437685	PDT002456599.1	Klebsiella_86		2022	China	PRJNA1056658
GCA 041029645	PDT002290937.1	Klebsiella_86		nan	China	PRJNA1021041
GCA 039610515	PDT002160666.1	Klebsiella_86	PDS000183505.1	2023	NA	PRJNA1100710
GCA 039847355	PDT002178469.1	Klebsiella_86	PDS000183505.1	2022	NA	PRJNA1113824
GCA 000566765	PDT000022727.1	Klebsiella_86		nan	USA	PRJNA219294
GCA 012971645	PDT000730633.1	Klebsiella_86		2016	South Korea	PRJNA625837
GCA 036553285	PDT002077843.1	Klebsiella_86		2021	Russia	PRJNA269675
GCA 033107025	PDT001960880.1	Klebsiella_86		2020	Vietnam	PRJEB64408
GCA 026310515	PDT001494466.1	Klebsiella_86	PDS000124950.1	2020	Hungary	PRJEB56918
GCA 900501255	PDT000369940.1	Klebsiella_86	PDS000124950.1	2014	Greece	PRJEB10018
GCA 900505695	PDT000370301.1	Klebsiella_86		2013	Romania	PRJEB10018
GCA 033122685	PDT001958062.1	Klebsiella_86		2016	Norway	PRJEB48268
GCA 900507145	PDT000370463.1	Klebsiella_86		2014	Serbia	PRJEB10018
GCA 043860075	**HBH_965_10**	Klebsiella_86		2010	Tunisia	PRJNA1160997
GCA 015207995	PDT000878327.1	Klebsiella_86	PDS000168656.1	2014	Russia	PRJNA269675
GCA 040271615	PDT002227184.1	Klebsiella_86		2010	United Kingdom	PRJNA1123654
GCA 040199875	PDT002208403.1	Klebsiella_86		nan	Saudi Arabia	PRJNA1116866
GCA 029523315	PDT001676473.1	Klebsiella_86		2018	China	PRJNA810290
GCA 024438995	PDT001366060.1	Klebsiella_86		2019	Guadeloupe	PRJNA778230
GCA 900774585	PDT000489698.1	Klebsiella_86		nan	NA	PRJEB31361
GCA 044324445	PDT002305484.1	Klebsiella_-		nan	USA	PRJNA271899
GCA 000474015	PDT000018268.2	Klebsiella_86		nan	NA	PRJNA177782
GCA 022204905	PDT000672164.1	Klebsiella_86		2014	Guadeloupe	PRJEB9692
GCA 041860315	PDT002369859.2	Klebsiella_86	PDS000197439.1	2009	Norway	PRJNA1098507
GCA 021917785	PDT001092341.1	Klebsiella_86	PDS000197439.1	2009	Norway	PRJEB27256
GCA 033336615	PDT001971230.1	Klebsiella_86		2020	Uganda	PRJNA1015582
GCA 003194935	PDT000326702.1	Klebsiella_86		2014	NA	PRJNA269675
GCA 032288225	PDT001911291.1	Klebsiella_86		2021	USA	PRJNA978102
GCA 032286465	PDT001911298.1	Klebsiella_86		2021	USA	PRJNA978102
GCA 900493665	PDT000354583.1	Klebsiella_86		2016	Thailand	PRJEB19226
GCA 021874715	PDT001198231.1	Klebsiella_86		2016	Japan	PRJDB12075
GCA 034738575	PDT002042335.1	Klebsiella_86	PDS000214453.1	2021	China	PRJNA1050895
GCA 046467915	PDT002556620.1	Klebsiella_86	PDS000214453.1	nan	NA	PRJNA786399
GCA 038375105	PDT002142025.1	Klebsiella_86		2023	China	PRJNA1081623
GCA 019401185	PDT001099782.1	Klebsiella_86		2016	China	PRJNA595653
GCA 036991685	PDT002098624.1	Klebsiella_86		2018	China	PRJNA1003408
GCA 034556135	PDT002039654.1	Klebsiella_86		2019	Russia	PRJNA1052416
GCA 034741275	PDT002042468.1	Klebsiella_86		2021	China	PRJNA1050895
GCA 036995365	PDT002098808.1	Klebsiella_86		2016	China	PRJNA1003408
GCA 034738095	PDT002042311.1	Klebsiella_86		2021	China	PRJNA1050895
GCA 026623765	PDT001512510.1	Klebsiella_-		2019	China	PRJNA905661
GCA 025762525	PDT001462495.1	Klebsiella_86		2017	China	PRJNA844536
GCA 034741615	PDT002042485.1	Klebsiella_86		2021	China	PRJNA1050895
GCA 034727095	PDT002041793.1	Klebsiella_86		2021	China	PRJNA1050895
GCA 044439385	PDT002456644.1	Klebsiella_86		2020	China	PRJNA1056658
GCA 039610975	PDT002160688.1	Klebsiella_86	PDS000183471.1	2022	NA	PRJNA1100710
GCA 039848045	PDT002178502.1	Klebsiella_86	PDS000183471.1	2023	NA	PRJNA1113824
GCA 019165675	PDT001085910.1	Klebsiella_86		2014	China	PRJNA736556
GCA 019165465	PDT001085900.1	Klebsiella_86		2016	China	PRJNA736556
GCA 019165595	PDT001085906.1	Klebsiella_86		2015	China	PRJNA736556
GCA 021871915	PDT001198379.1	Klebsiella_86	PDS000100761.1	2015	Japan	PRJDB12075
GCA 000529645	PDT000020956.1	Klebsiella_86	PDS000097499.1	nan	NA	PRJEB4361
GCA 020525885	PDT001157375.1	Klebsiella_86	PDS000097499.1	2008	France	PRJNA768294
GCA 902507955	PDT000662914.1	Klebsiella_86		2017	China	PRJEB34922
GCA 033107525	PDT001960852.1	Klebsiella_86		2017	Vietnam	PRJEB64408
GCA 036991865	PDT002098634.1	Klebsiella_86		2014	China	PRJNA1003408
GCA 015283665	PDT000884767.1	Klebsiella_86		2019	China	PRJNA672205
GCA 006131335	PDT000518718.1	Klebsiella_86		2015	China	PRJNA510171
GCA 026198175	PDT001488447.1	Klebsiella_86		2020	China	PRJNA838135
GCA 022227185	PDT000440760.1	Klebsiella_86		nan	NA	PRJEB21605
GCA 041448855	PDT002340822.1	Klebsiella_86		2021	China	PRJNA1148939
GCA 030067255	PDT001758446.1	Klebsiella_86		2022	China	PRJNA971333
GCA 022869145	PDT001285153.1	Klebsiella_86		2017	China	PRJNA822025
GCA 034735055	PDT002042160.1	Klebsiella_86		2021	China	PRJNA1050895
GCA 040989725	PDT002279960.1	Klebsiella_86		nan	China	PRJNA1021041
GCA 020481285	PDT001151369.1	Klebsiella_86		2014	China	PRJNA612328
GCA 022011985	PDT001110150.1	Klebsiella_5559		2020	China	PRJNA745055
GCA 023743025	PDT001336676.1	Klebsiella_86		2019	China	PRJNA833902
GCA 902508435	PDT000662965.1	Klebsiella_86		2017	China	PRJEB34922
GCA 021873295	PDT001198309.1	Klebsiella_86		2017	Japan	PRJDB12075
GCA 038376155	PDT002142112.1	Klebsiella_86		2021	China	PRJNA1081623
GCA 036995625	PDT002098821.1	Klebsiella_86		2015	China	PRJNA1003408
GCA 022127305	PDT000871148.1	Klebsiella_86	PDS000074639.1	2014	Vietnam	PRJNA646358
GCA 022127325	PDT000871147.1	Klebsiella_86	PDS000074639.1	2014	Vietnam	PRJNA646358
GCA 022127165	PDT000871205.1	Klebsiella_86	PDS000074639.1	2014	Vietnam	PRJNA646358
GCA 033108325	PDT001960808.1	Klebsiella_86		2012	Vietnam	PRJEB64408
GCA 022228465	PDT000458529.1	Klebsiella_86	PDS000042808.1	2015	Vietnam	PRJNA413040
GCA 022228405	PDT000458532.1	Klebsiella_86	PDS000042808.1	2015	Vietnam	PRJNA413040
GCA 022228265	PDT000458538.1	Klebsiella_86	PDS000042808.1	2015	Vietnam	PRJNA413040
GCA 022228245	PDT000458540.1	Klebsiella_86	PDS000042808.1	2015	Vietnam	PRJNA413040
GCA 022228285	PDT000458537.1	Klebsiella_86	PDS000042808.1	2015	Vietnam	PRJNA413040
GCA 022228345	PDT000458535.1	Klebsiella_86	PDS000042808.1	2015	Vietnam	PRJNA413040
GCA 022228325	PDT000458539.1	Klebsiella_86	PDS000042808.1	2015	Vietnam	PRJNA413040
GCA 022228365	PDT000458533.1	Klebsiella_86	PDS000042808.1	2015	Vietnam	PRJNA413040
GCA 021853455	PDT001214083.1	Klebsiella_86		2018	Japan	PRJDB11378
GCA 021853475	PDT001214082.1	Klebsiella_86		2018	Japan	PRJDB11378
GCA 021854195	PDT001214050.1	Klebsiella_86		2017	Japan	PRJDB11378
GCA 021853865	PDT001214064.1	Klebsiella_86		2017	Japan	PRJDB11378
GCA 016634825	PDT000932920.1	Klebsiella_86		2014	Australia	PRJNA480723
GCA 040189395	PDT002208112.1	Klebsiella_86		nan	Saudi Arabia	PRJNA1116866
GCA 030822775	PDT001702214.1	Klebsiella_86		2019	Japan	PRJDB10842
GCA 031068235	PDT001702693.1	Klebsiella_86		2019	Japan	PRJDB10842
GCA 900775415	PDT000489781.1	Klebsiella_86		nan	NA	PRJEB31361
GCA 040197015	PDT002208261.1	Klebsiella_86		nan	Saudi Arabia	PRJNA1116866
GCA 022208865	PDT000636354.1	Klebsiella_86		2015	France	PRJNA551371
GCA 021853115	PDT001214102.1	Klebsiella_86		2018	Japan	PRJDB11378
GCA 043358925	PDT002423178.1	Klebsiella_86		nan	Saudi Arabia	PRJNA1116866
GCA 026198235	PDT001488450.1	Klebsiella_86		2020	China	PRJNA838135
GCA 039609435	PDT002160612.1	Klebsiella_3509	PDS000182304.3	2023	NA	PRJNA1100710
GCA 039848085	PDT002178504.1	Klebsiella_3509	PDS000182304.3	2023	NA	PRJNA1113824
GCA 039847725	PDT002178487.1	Klebsiella_3509	PDS000182304.3	2023	NA	PRJNA1113824
GCA 039606475	PDT002160476.1	Klebsiella_3509	PDS000182304.3	2023	NA	PRJNA1100710
GCA 041031145	PDT002291013.1	Klebsiella_3509	PDS000182304.3	nan	China	PRJNA1021041
GCA 041031205	PDT002291016.1	Klebsiella_3509	PDS000182304.3	nan	China	PRJNA1021041

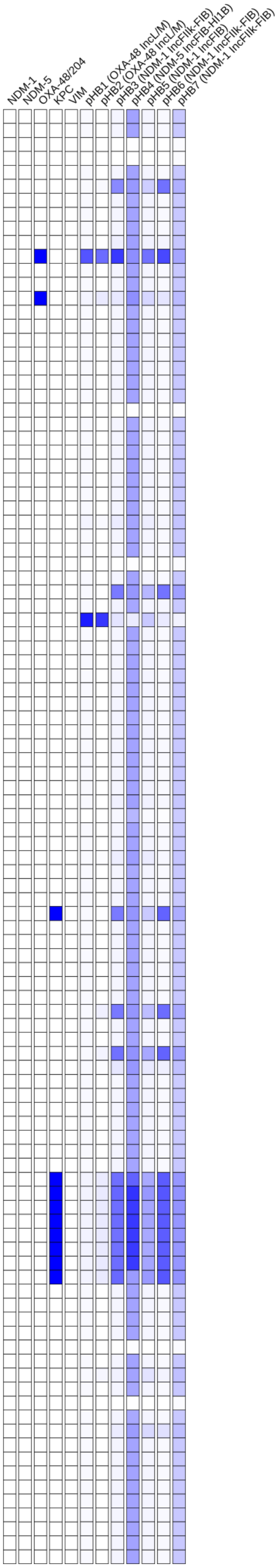

Tree 12: GCA_043860195

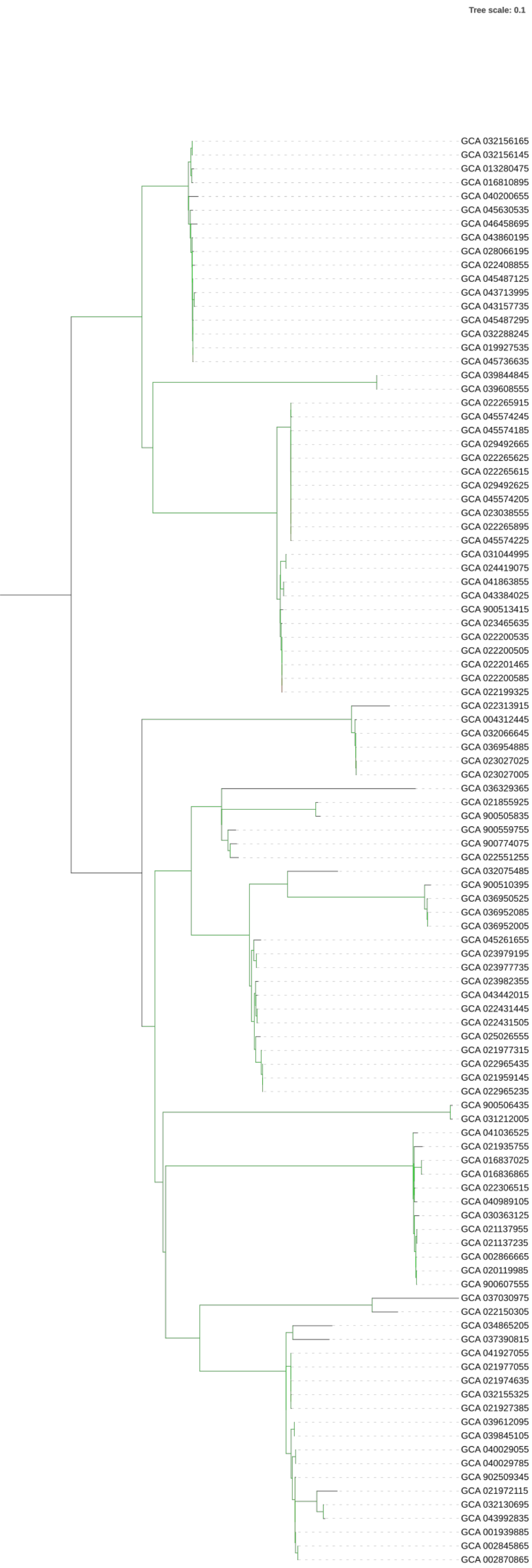

	Isolate	MLST	SNP Cluster	Collection Year	Country	BioProject
GCA 032156165	PDT001902803.1	Klebsiella_987	PDS000160856.1	2018	Norway	PRJEB48268
GCA 032156145	PDT001902804.1	Klebsiella_987	PDS000160856.1	2018	Norway	PRJEB48268
GCA 013280475	PDT000761304.1	Klebsiella_987		2019	Lebanon	PRJNA634137
GCA 016810895	PDT000950670.1	Klebsiella_987		2017	Qatar	PRJNA690895
GCA 040200655	PDT002208442.1	Klebsiella_987		nan	Saudi Arabia	PRJNA1116866
GCA 045630535	PDT002500098.1	Klebsiella_987		2024	USA	PRJNA288601
GCA 046458695	PDT002554583.1	Klebsiella_987		2019	Qatar	PRJNA1005941
GCA 043860195	**HBH_2668_14**	Klebsiella_987		2014	Tunisia	PRJNA1160997
GCA 028066195	PDT000878052.3	Klebsiella_987		2018	USA	PRJNA288601
GCA 022408855	PDT001256452.1	Klebsiella_987		nan	USA	PRJNA786742
GCA 045487125	PDT002191129.1	Klebsiella_987	PDS000161308.3	2018	USA	PRJNA1054115
GCA 043713995	PDT002387637.1	Klebsiella_987	PDS000199202.1	2024	USA	PRJNA1127607
GCA 043157735	PDT002230378.1	Klebsiella_987	PDS000199202.1	2024	USA	PRJNA1127607
GCA 045487295	PDT002191123.1	Klebsiella_987	PDS000161308.3	2019	USA	PRJNA1054115
GCA 032288245	PDT001911290.1	Klebsiella_987	PDS000161308.3	2021	USA	PRJNA978102
GCA 019927535	PDT001128404.2	Klebsiella_987	PDS000161308.3	2019	USA	PRJNA717739
GCA 045736635	PDT002513471.1	Klebsiella_987	PDS000161308.3	2024	USA	PRJNA475751
GCA 039844845	PDT002178359.1	Klebsiella_-	PDS000183523.1	2023	NA	PRJNA1113824
GCA 039608555	PDT002160568.1	Klebsiella_-	PDS000183523.1	2022	NA	PRJNA1100710
GCA 022265915	PDT000435268.1	Klebsiella_496	PDS000071977.4	2014	Singapore	PRJNA342893
GCA 045574245	PDT002509415.1	Klebsiella_496	PDS000071977.4	nan	Singapore	PRJNA1171009
GCA 045574185	PDT002509412.1	Klebsiella_496	PDS000071977.4	nan	Singapore	PRJNA1171009
GCA 029492665	PDT001672886.1	Klebsiella_496	PDS000071977.4	nan	Singapore	PRJNA765801
GCA 022265625	PDT000435320.1	Klebsiella_496	PDS000071977.4	2014	Singapore	PRJNA342893
GCA 022265615	PDT000435317.1	Klebsiella_496	PDS000071977.4	2014	Singapore	PRJNA342893
GCA 029492625	PDT001672888.1	Klebsiella_496	PDS000071977.4	nan	Singapore	PRJNA765801
GCA 045574205	PDT002509413.1	Klebsiella_496	PDS000071977.4	nan	Singapore	PRJNA1171009
GCA 023038555	PDT001288449.1	Klebsiella_496	PDS000071977.4	nan	Singapore	PRJNA757551
GCA 022265895	PDT000435269.1	Klebsiella_-	PDS000071977.4	2014	Singapore	PRJNA342893
GCA 045574225	PDT002509414.1	Klebsiella_496	PDS000071977.4	nan	Singapore	PRJNA1171009
GCA 031044995	PDT001744196.2	Klebsiella_496	PDS000178659.1	2022	USA	PRJNA278886
GCA 024419075	PDT001367717.3	Klebsiella_496	PDS000178659.1	2022	USA	PRJNA288601
GCA 041863855	PDT002373757.1	Klebsiella_496	PDS000197593.1	2009	Norway	PRJNA1098507
GCA 043384025	PDT002281232.1	Klebsiella_496	PDS000197593.1	2009	Norway	PRJEB27256
GCA 900513415	PDT000369506.1	Klebsiella_4064		2013	Israel	PRJEB10018
GCA 023465635	PDT001289381.1	Klebsiella_-		2020	Australia	PRJNA777643
GCA 022200535	PDT000706783.1	Klebsiella_-	PDS000059976.3	2018	Italy	PRJEB27342
GCA 022200505	PDT000706785.1	Klebsiella_496	PDS000059976.3	2018	Italy	PRJEB27342
GCA 022201465	PDT000706611.1	Klebsiella_-	PDS000059976.3	2018	Italy	PRJEB27342
GCA 022200585	PDT000706777.1	Klebsiella_496	PDS000059976.3	2018	Italy	PRJEB27342
GCA 022199325	PDT000708315.1	Klebsiella_-	PDS000059976.3	2018	Italy	PRJEB27342
GCA 022313915	PDT000136264.2	Klebsiella_485		2012	United Kingdom	PRJNA306133
GCA 004312445	PDT000471488.1	Klebsiella_485		2014	Japan	PRJDB7163
GCA 032066645	PDT001905133.1	Klebsiella_485	PDS000053037.8	2018	Norway	PRJEB48268
GCA 036954885	PDT002096791.2	Klebsiella_485	PDS000053037.8	2018	Netherlands	PRJNA927131
GCA 023027025	PDT001289537.1	Klebsiella_-	PDS000053037.8	2020	Australia	PRJNA777643
GCA 023027005	PDT001289538.1	Klebsiella_485	PDS000053037.8	2020	Australia	PRJNA777643
GCA 036329365	PDT002071127.1	Klebsiella_485		2020	China	PRJNA930978
GCA 021855925	PDT001209403.1	Klebsiella_163		2018	Italy	PRJEB27342
GCA 900505835	PDT000370309.1	Klebsiella_163		2013	Croatia	PRJEB10018
GCA 900559755	PDT000595196.1	Klebsiella_485		nan	United Kingdom	PRJEB28457
GCA 900774075	PDT000489647.1	Klebsiella_485		nan	NA	PRJEB31361
GCA 022551255	PDT001263629.1	Klebsiella_485		2017	USA	PRJNA726883
GCA 032075485	PDT001904986.1	Klebsiella_485		2017	Norway	PRJEB48268
GCA 900510395	PDT000369184.1	Klebsiella_3649		2013	France	PRJEB10018
GCA 036950525	PDT002096696.2	Klebsiella_3649		2019	Belgium	PRJNA927131
GCA 036952085	PDT002096735.2	Klebsiella_3649	PDS000175698.1	2017	Belgium	PRJNA927131
GCA 036952005	PDT002096734.2	Klebsiella_3649	PDS000175698.1	2017	Belgium	PRJNA927131
GCA 045261655	PDT002495236.1	Klebsiella_485		nan	NA	PRJNA1187231
GCA 023979195	PDT001347538.1	Klebsiella_485	PDS000112639.1	2012	USA	PRJNA842739
GCA 023977735	PDT001347566.1	Klebsiella_485	PDS000112639.1	2012	USA	PRJNA842739
GCA 023982355	PDT001347537.1	Klebsiella_485		2019	Norway	PRJNA835677
GCA 043442015	PDT002421651.1	Klebsiella_485		2019	USA	PRJNA288601
GCA 022431445	PDT001256106.1	Klebsiella_485	PDS000105717.1	2019	USA	PRJNA325243
GCA 022431505	PDT001256103.1	Klebsiella_485	PDS000105717.1	2019	USA	PRJNA325243
GCA 025026555	PDT001412323.1	Klebsiella_485		2019	Norway	PRJNA835677
GCA 021977315	PDT001011401.1	Klebsiella_-		2015	Norway	PRJEB42350
GCA 022965435	PDT001284042.2	Klebsiella_-	PDS000107774.2	2017	USA	PRJNA789565
GCA 021959145	PDT001020144.1	Klebsiella_-	PDS000107774.2	2017	USA	PRJNA658369
GCA 022965235	PDT001284041.1	Klebsiella_-	PDS000107774.2	2017	USA	PRJNA789565
GCA 900506435	PDT000370486.1	Klebsiella_485		2014	Ireland	PRJEB10018
GCA 031212005	PDT001876217.2	Klebsiella_-		2023	USA	PRJNA288601
GCA 041036525	PDT002291942.1	Klebsiella_914		nan	China	PRJNA1021041
GCA 021935755	PDT001087216.1	Klebsiella_914		2018	Australia	PRJNA529744
GCA 016837025	PDT000952438.1	Klebsiella_914	PDS000080493.2	2012	Paraguay	PRJNA698801
GCA 016836865	PDT000952431.1	Klebsiella_914	PDS000080493.2	2012	Paraguay	PRJNA698801
GCA 022306515	PDT000176099.2	Klebsiella_914		nan	NA	PRJEB5065
GCA 040989105	PDT002279766.1	Klebsiella_914		nan	China	PRJNA1021041
GCA 030363125	PDT001792608.1	Klebsiella_6114		2020	China	PRJNA925808
GCA 021137955	PDT001201758.1	Klebsiella_2719	PDS000038919.5	2020	Switzerland	PRJNA759850
GCA 021137235	PDT001201735.1	Klebsiella_2719	PDS000038919.5	2020	Switzerland	PRJNA758223
GCA 002866665	PDT000277133.1	Klebsiella_2719	PDS000038919.5	2013	Lebanon	PRJNA388273
GCA 020119985	PDT001137078.1	Klebsiella_2719	PDS000038919.5	2013	Switzerland	PRJNA543274
GCA 900607555	PDT000411390.1	Klebsiella_2719	PDS000038919.5	2013	Switzerland	PRJEB28660
GCA 037030975	PDT002100111.1	Klebsiella_485		2023	Mexico	PRJNA1051187
GCA 022150305	PDT001244886.1	Klebsiella_485		2016	United Kingdom	PRJNA788733
GCA 034865205	PDT002044322.1	Klebsiella_485		2014	France	PRJNA1054041
GCA 037390815	PDT002117060.1	Klebsiella_815		2014	USA	PRJNA288601
GCA 041927055	PDT002383492.1	Klebsiella_485	PDS000085062.4	2015	Norway	PRJNA1098507
GCA 021977055	PDT001011424.1	Klebsiella_485	PDS000085062.4	2015	Norway	PRJEB42350
GCA 021974635	PDT001011609.1	Klebsiella_485	PDS000085062.4	2015	Norway	PRJEB42350
GCA 032155325	PDT001902847.1	Klebsiella_485	PDS000085062.4	2017	Norway	PRJEB48268
GCA 021927385	PDT001091768.1	Klebsiella_485	PDS000085062.4	2015	Norway	PRJEB27256
GCA 039612095	PDT002160744.1	Klebsiella_485	PDS000183592.1	2022	NA	PRJNA1100710
GCA 039845105	PDT002178372.1	Klebsiella_485	PDS000183592.1	2023	NA	PRJNA1113824
GCA 040029055	PDT002187530.1	Klebsiella_485	PDS000184186.2	2019	China	PRJNA1116330
GCA 040029785	PDT002187531.2	Klebsiella_485	PDS000184186.2	2019	China	PRJNA1116332
GCA 902509345	PDT000663058.1	Klebsiella_485		2017	China	PRJEB34922
GCA 021972115	PDT001013483.1	Klebsiella_485		2018	Denmark	PRJEB37711
GCA 032130695	PDT001905385.1	Klebsiella_485		2020	Japan	PRJDB16654
GCA 043992835	PDT002376569.1	Klebsiella_485		2016	Japan	PRJDB14656
GCA 001939885	PDT000177253.2	Klebsiella_485	PDS000054678.2	2015	China	PRJNA349219
GCA 002845865	PDT000274017.1	Klebsiella_485	PDS000054678.2	2009	China	PRJNA420654
GCA 002870865	PDT000277856.1	Klebsiella_485	PDS000054678.2	2011	China	PRJNA428155

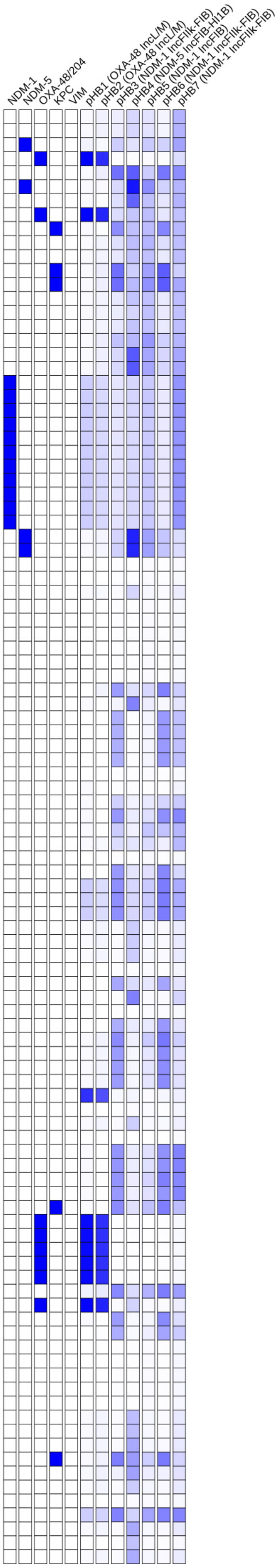

Tree 13: GCA_043861645

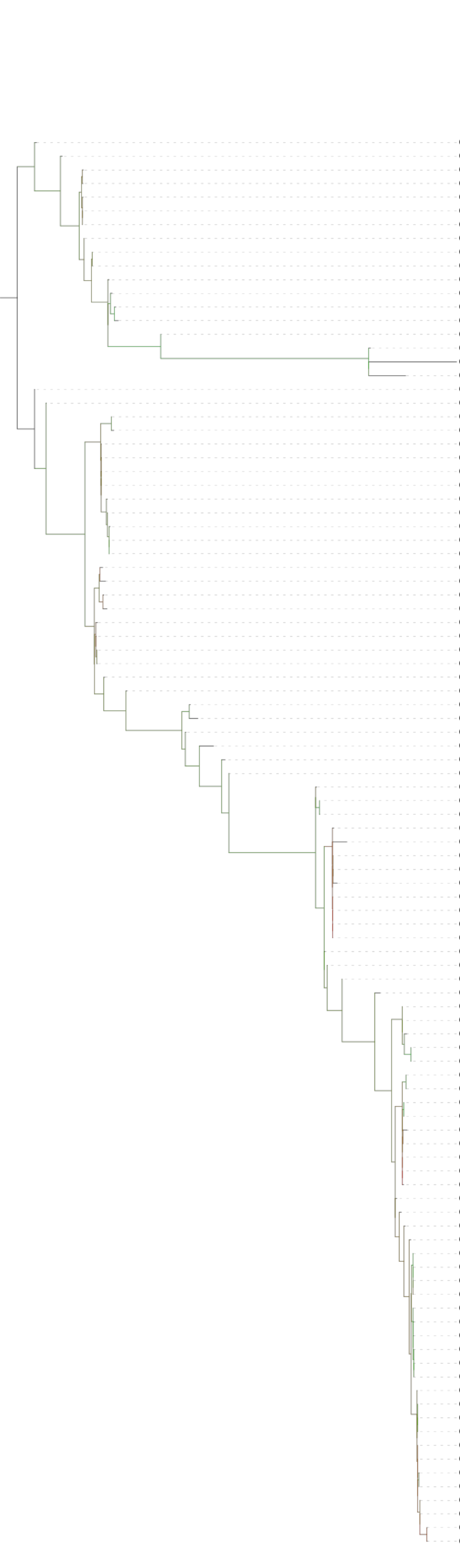

	Isolate	MLST	SNP Cluster	Collection Year	Country	BioProject
GCA 003977205	PDT000429864.1	Klebsiella_11		nan	Egypt	PRJNA508509
GCA 003977125	PDT000429861.1	Klebsiella_11	PDS000045344.10	nan	Egypt	PRJNA508509
GCA 023930245	PDT001345441.1	Klebsiella_11	PDS000112591.1	2018	Israel	PRJEB53419
GCA 023929625	PDT001345469.1	Klebsiella_11	PDS000112591.1	2018	Israel	PRJEB53419
GCA 023929685	PDT001345462.1	Klebsiella_11	PDS000112591.1	2018	Israel	PRJEB53419
GCA 023927865	PDT001345470.1	Klebsiella_11	PDS000112591.1	2019	Israel	PRJEB53419
GCA 023929885	PDT001345461.1	Klebsiella_11	PDS000112591.1	2018	Israel	PRJEB53419
GCA 021798705	PDT001227457.1	Klebsiella_11	PDS000045344.10	2014	United Kingdom	PRJNA788733
GCA 900512285	PDT000369263.1	Klebsiella_11	PDS000045344.10	2014	Germany	PRJEB10018
GCA 902156005	PDT000663173.1	Klebsiella_11	PDS000045344.10	2014	Germany	PRJEB33308
GCA 022151685	PDT000858357.1	Klebsiella_1640		2016	USA	PRJNA658369
GCA 044366505	PDT002455612.1	Klebsiella_11		nan	Saudi Arabia	PRJNA1116866
GCA 041806955	PDT002369363.1	Klebsiella_11		2022	Ukraine	PRJNA1152760
GCA 947575555	PDT001769515.1	Klebsiella_11		2022	Germany	PRJEB58018
GCA 006130345	PDT000518667.1	Klebsiella_11	PDS000102670.3	nan	China	PRJNA510171
GCA 022116175	PDT001244870.1	Klebsiella_11	PDS000103169.12	2016	United Kingdom	PRJNA788733
GCA 030587745	PDT001837965.1	Klebsiella_11	PDS000146165.3	2016	South Korea	PRJNA521445
GCA 003583235	PDT000383130.1	Klebsiella_11	PDS000045343.1	2016	Austria	PRJNA488173
GCA 023457815	PDT001307905.1	Klebsiella_11	PDS000045344.10	nan	Singapore	PRJNA765801
GCA 045572155	PDT002509377.1	Klebsiella_11	PDS000045344.10	nan	Singapore	PRJNA1171009
GCA 026354855	PDT001493207.1	Klebsiella_11	PDS000046910.86	2020	Paraguay	PRJEB56918
GCA 023826215	PDT001325858.1	Klebsiella_11	PDS000046910.86	2017	Thailand	PRJNA814829
GCA 032636025	PDT001923286.1	Klebsiella_11	PDS000046910.86	2016	Peru	PRJNA990326
GCA 047565655	PDT002613153.1	Klebsiella_11	PDS000046910.86	2019	Chile	PRJNA962904
GCA 033336475	PDT001971223.1	Klebsiella_11	PDS000165202.2	2021	Uganda	PRJNA1015582
GCA 033336235	PDT001971211.1	Klebsiella_11	PDS000165202.2	2021	Uganda	PRJNA1015582
GCA 002248545	PDT000233413.1	Klebsiella_11	PDS000046910.86	2016	Thailand	PRJNA389557
GCA 022282825	PDT000431935.1	Klebsiella_11	PDS000046910.86	2015	Thailand	PRJDB5929
GCA 021984515	PDT001003297.1	Klebsiella_11	PDS000046910.86	2017	Thailand	PRJDB5929
GCA 900092735	PDT000139244.1	Klebsiella_11	PDS000046910.86	2015	Thailand	PRJEB11403
GCA 900092785	PDT000140928.1	Klebsiella_11	PDS000046910.86	2014	Thailand	PRJEB11403
GCA 041806695	PDT002369355.1	Klebsiella_11		2022	Ukraine	PRJNA1152760
GCA 030177035	PDT001771583.1	Klebsiella_11		2022	Germany	PRJNA972998
GCA 043976115	PDT002412018.1	Klebsiella_11		2017	Canada	PRJNA855907
GCA 023926025	PDT001345467.1	Klebsiella_11	PDS000112591.1	2018	Israel	PRJEB53419
GCA 045967875	PDT002526876.1	Klebsiella_11		2024	USA	PRJNA288601
GCA 016651515	PDT000937791.1	Klebsiella_11	PDS000079304.4	nan	Egypt	PRJNA689413
GCA 022027815	PDT000925033.1	Klebsiella_11	PDS000173787.1	2016	Singapore	PRJNA674942
GCA 036576685	PDT002077871.1	Klebsiella_11	PDS000173787.1	2014	Egypt	PRJNA844012
GCA 900503115	PDT000370230.1	Klebsiella_11	PDS000045344.10	2014	United Kingdom	PRJEB10018
GCA 043861645	**HBH_6_15**	Klebsiella_11		2015	Tunisia	PRJNA1160997
GCA 947576325	PDT001769443.1	Klebsiella_11	PDS000148349.1	2022	Germany	PRJEB58018
GCA 947576835	PDT001769471.1	Klebsiella_11	PDS000148349.1	2022	Germany	PRJEB58018
GCA 916162515	PDT001141266.1	Klebsiella_11	PDS000045344.10	2013	Netherlands	PRJEB41042
GCA 003977085	PDT000429860.1	Klebsiella_11	PDS000045344.10	nan	Egypt	PRJNA508509
GCA 045037455	PDT002479293.1	Klebsiella_11		2020/2023	Egypt	PRJNA1180919
GCA 022186265	PDT000716565.1	Klebsiella_11	PDS000079304.4	2018	Germany	PRJEB35890
GCA 022244385	PDT000441155.1	Klebsiella_11	PDS000045344.10	2012	Nepal	PRJEB1800
GCA 022293675	PDT000423776.1	Klebsiella_11	PDS000045350.2	nan	NA	PRJEB17615
GCA 022293655	PDT000423775.1	Klebsiella_11	PDS000045350.2	nan	NA	PRJEB17615
GCA 022328365	PDT000535638.1	Klebsiella_11	PDS000046967.1	2011	Spain	PRJEB30771
GCA 044973085	PDT002090357.1	Klebsiella_11	PDS000046910.86	2015	Vietnam	PRJDB6407
GCA 020477735	PDT001151240.1	Klebsiella_11	PDS000046910.86	2019	China	PRJNA612328
GCA 022053265	PDT000881187.1	Klebsiella_11	PDS000046910.86	2019	China	PRJNA575579
GCA 020478355	PDT001151265.1	Klebsiella_11	PDS000046910.86	2019	China	PRJNA612328
GCA 021981475	PDT001008113.1	Klebsiella_11	PDS000046910.86	2015	Thailand	PRJDB5929
GCA 022327845	PDT000535683.1	Klebsiella_11	PDS000046910.86	2016	Spain	PRJEB30771
GCA 022327945	PDT000535679.1	Klebsiella_11	PDS000046910.86	2016	Spain	PRJEB30771
GCA 022327985	PDT000535677.1	Klebsiella_11	PDS000046910.86	2016	Spain	PRJEB30771
GCA 021983835	PDT001003357.1	Klebsiella_11	PDS000046910.86	2016	Thailand	PRJDB5929
GCA 022053225	PDT000881188.1	Klebsiella_11	PDS000046910.86	2019	China	PRJNA575579
GCA 024652545	PDT001388548.1	Klebsiella_11	PDS000046910.86	2020	Brazil	PRJNA830894
GCA 013138485	PDT000744695.1	Klebsiella_11	PDS000046910.86	2015	Brazil	PRJNA580478
GCA 023027725	PDT001289502.1	Klebsiella_11	PDS000149983.1	2020	Australia	PRJNA777643
GCA 030283745	PDT001782321.2	Klebsiella_11	PDS000149983.1	2020	Australia	PRJNA967113
GCA 045037375	PDT002479292.1	Klebsiella_11		2020/2023	Egypt	PRJNA1180919
GCA 047123215	PDT002590040.1	Klebsiella_11	PDS000216219.1	2023	USA	PRJNA288601
GCA 035557175	PDT002452039.1	Klebsiella_11	PDS000216219.1	2023	USA	PRJNA812595
GCA 034361505	PDT002029843.1	Klebsiella_11	PDS000185312.1	2018	China	PRJNA832474
GCA 040195615	PDT002208208.1	Klebsiella_11	PDS000185312.1	2018	China	PRJNA353728
GCA 047564535	PDT002613322.1	Klebsiella_11	PDS000046910.86	2018	Chile	PRJNA962904
GCA 047540185	PDT002613311.1	Klebsiella_11	PDS000046910.86	2018	Chile	PRJNA962904
GCA 047564335	PDT002613332.1	Klebsiella_11	PDS000046910.86	2019	Chile	PRJNA962904
GCA 047564395	PDT002613325.1	Klebsiella_11	PDS000046910.86	2019	Chile	PRJNA962904
GCA 024742255	PDT001398900.1	Klebsiella_11	PDS000046910.86	2019	Argentina	PRJNA870418
GCA 044010465	PDT002245738.1	Klebsiella_11	PDS000046910.86	2023	Spain	PRJNA1133624
GCA 021020875	PDT001195411.1	Klebsiella_11		2020	Brazil	PRJNA755436
GCA 028060715	PDT001275804.3	Klebsiella_11	PDS000079304.4	2022	USA	PRJNA288601
GCA 044362655	PDT002455515.1	Klebsiella_11		nan	Saudi Arabia	PRJNA1116866
GCA 023925995	PDT001345471.1	Klebsiella_11	PDS000112591.1	2018	Israel	PRJEB53419
GCA 029078385	PDT001655822.1	Klebsiella_11	PDS000138389.1	2015	United Kingdom	PRJNA917129
GCA 022001095	PDT000987010.1	Klebsiella_11	PDS000046910.86	2016	Thailand	PRJDB5929
GCA 021999345	PDT000987042.1	Klebsiella_11	PDS000046910.86	2016	Thailand	PRJDB5929
GCA 021996475	PDT000987051.1	Klebsiella_11	PDS000046910.86	2016	Thailand	PRJDB5929
GCA 021996535	PDT000987050.1	Klebsiella_11	PDS000046910.86	2016	Thailand	PRJDB5929
GCA 037052585	PDT002102104.1	Klebsiella_11	PDS000060651.29	2016	Jordan	PRJNA1076682
GCA 964273615	PDT002406610.1	Klebsiella_11		2024	Slovakia	PRJEB72521
GCA 964238725	PDT002346905.1	Klebsiella_11	PDS000060651.29	2024	Slovakia	PRJEB72521
GCA 022235485	PDT000442286.1	Klebsiella_11	PDS000060651.29	2017	Ireland	PRJEB19229
GCA 043863385	**HBH_40_17**	Klebsiella_11	PDS000060651.29	2017	Tunisia	PRJNA1160997
GCA 043863185	**HBH_4427_16**	Klebsiella_11	PDS000060651.29	2016	Tunisia	PRJNA1160997
GCA 034388275	PDT002031405.1	Klebsiella_11	PDS000046910.86	2018	China	PRJNA832474
GCA 046039295	PDT002531290.1	Klebsiella_11	PDS000046910.86	2019	China	PRJNA1194345
GCA 046039105	PDT002531281.1	Klebsiella_11	PDS000046910.86	2019	China	PRJNA1194345
GCA 046039015	PDT002531277.1	Klebsiella_11	PDS000046910.86	2019	China	PRJNA1194345
GCA 004832985	PDT000496026.1	Klebsiella_11	PDS000046910.86	2018	Thailand	PRJNA532291
GCA 022305175	PDT000181850.2	Klebsiella_11	PDS000046910.86	nan	NA	PRJEB11403
GCA 021983275	PDT001003381.1	Klebsiella_11	PDS000046910.86	2016	Thailand	PRJDB5929
GCA 022282285	PDT000431962.1	Klebsiella_11	PDS000046910.86	2016	Thailand	PRJDB5929
GCA 022282365	PDT000431959.1	Klebsiella_11	PDS000046910.86	2016	Thailand	PRJDB5929
GCA 004832575	PDT000496005.1	Klebsiella_11	PDS000046910.86	2018	Thailand	PRJNA532291
GCA 022282775	PDT000431938.1	Klebsiella_11	PDS000046910.86	2015	Thailand	PRJDB5929
GCA 019660105	PDT001112707.1	Klebsiella_11	PDS000046910.86	2015	China	PRJNA752009

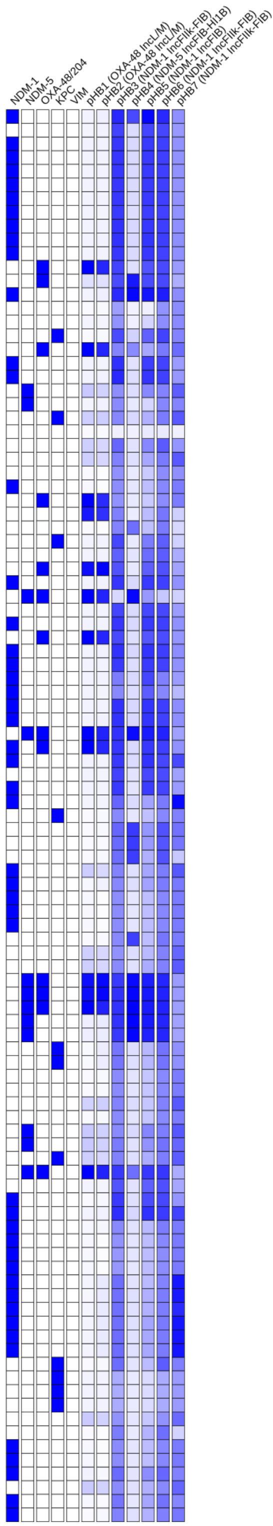

Supplement: Data S1A — Phylogenetic trees (1–12) generated in this study. [file aac.00142-26-s0001.pdf]
