# Supplementary material for: Leveraging the global genomic epidemiology of carbapenemase-producing Klebsiella pneumoniae to inform infection prevention in Tunisian hospitals
Source: Antimicrob Agents Chemother. 2026 May 6;70(6):e00142-26. doi: 10.1128/aac.00142-26 (PMC13231914; doi:10.1128/aac.00142-26)

Tree 14: GCA_043863025

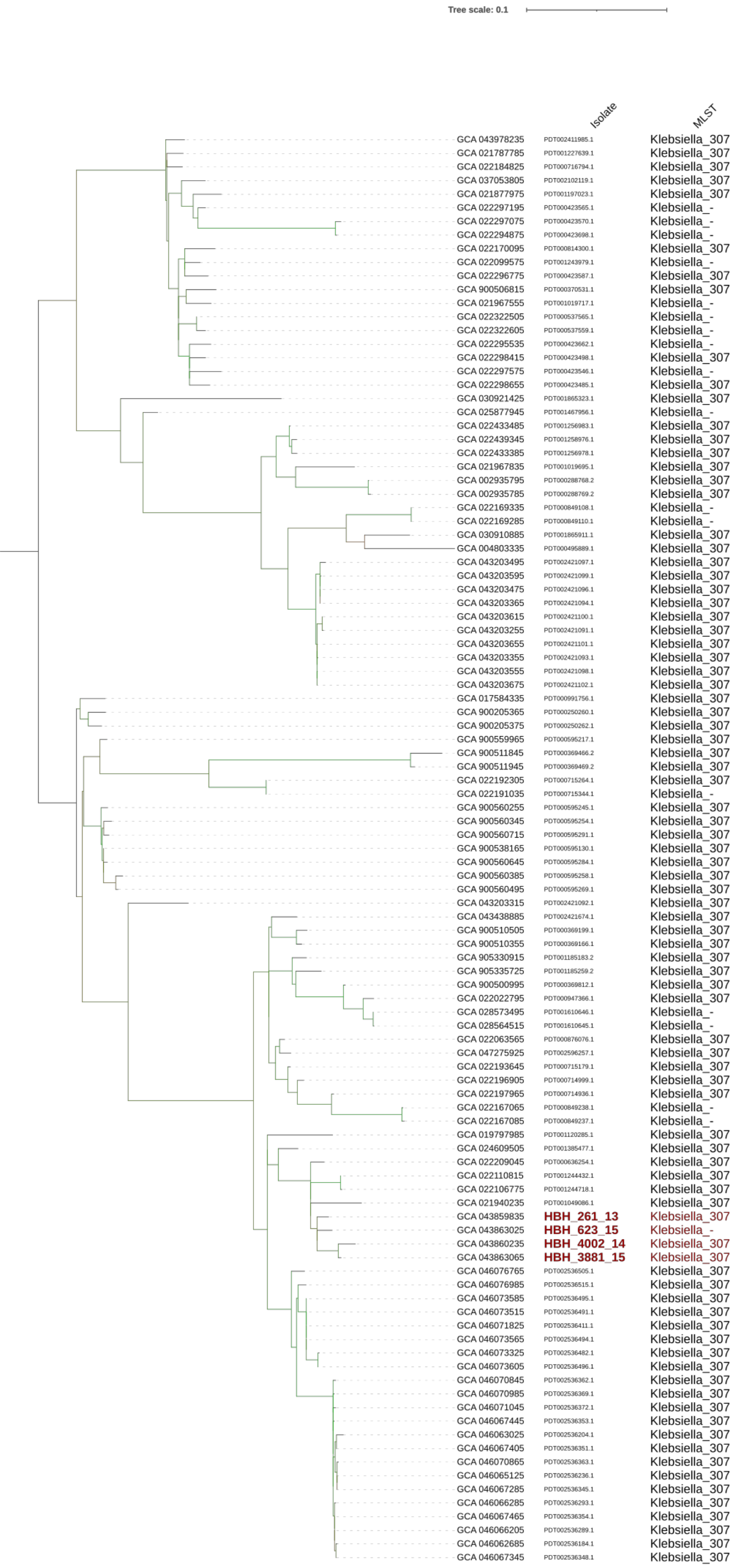

	Isolate	MLST	SNP Cluster	Collection Year	Country	BioProject
GCA 043978235	PDT002411985.1	Klebsiella_307		2017	Canada	PRJNA855907
GCA 021787785	PDT001227639.1	Klebsiella_307	PDS000045377.17	2014	United Kingdom	PRJNA788733
GCA 022184825	PDT000716794.1	Klebsiella_307	PDS000045377.17	2014	Spain	PRJNA575837
GCA 037053805	PDT002102119.1	Klebsiella_307	PDS000176502.1	2015	Jordan	PRJNA1076682
GCA 021877975	PDT001197023.1	Klebsiella_307		nan	Australia	PRJNA751993
GCA 022297195	PDT000423565.1	Klebsiella_-	PDS000051246.2	nan	NA	PRJEB17615
GCA 022297075	PDT000423570.1	Klebsiella_-	PDS000051246.2	nan	NA	PRJEB17615
GCA 022294875	PDT000423698.1	Klebsiella_-	PDS000051246.2	nan	NA	PRJEB17615
GCA 022170095	PDT000814300.1	Klebsiella_307		nan	Indonesia	PRJDB7330
GCA 022099575	PDT001243979.1	Klebsiella_-	PDS000074878.9	2016	United Kingdom	PRJNA788733
GCA 022296775	PDT000423587.1	Klebsiella_307		nan	NA	PRJEB17615
GCA 900506815	PDT000370531.1	Klebsiella_307		2013	Belgium	PRJEB10018
GCA 021967555	PDT001019717.1	Klebsiella_-	PDS000038607.20	2018	USA	PRJNA658369
GCA 022322505	PDT000537565.1	Klebsiella_-	PDS000189621.16	2014	Norway	PRJEB27256
GCA 022322605	PDT000537559.1	Klebsiella_-	PDS000189621.16	2014	Norway	PRJEB27256
GCA 022295535	PDT000423662.1	Klebsiella_-		nan	NA	PRJEB17615
GCA 022298415	PDT000423498.1	Klebsiella_307		nan	NA	PRJEB17615
GCA 022297575	PDT000423546.1	Klebsiella_-		nan	NA	PRJEB17615
GCA 022298655	PDT000423485.1	Klebsiella_307	PDS000082283.3	nan	NA	PRJEB17615
GCA 030921425	PDT001865323.1	Klebsiella_307	PDS000045377.17	nan	USA	PRJNA994967
GCA 025877945	PDT001467956.1	Klebsiella_-		2019	Norway	PRJEB56146
GCA 022433485	PDT001256983.1	Klebsiella_307	PDS000073377.3	2016	USA	PRJNA658369
GCA 022439345	PDT001258976.1	Klebsiella_307	PDS000073377.3	2016	USA	PRJNA658369
GCA 022433385	PDT001256978.1	Klebsiella_307	PDS000073377.3	2016	USA	PRJNA658369
GCA 021967835	PDT001019695.1	Klebsiella_307		2018	USA	PRJNA658369
GCA 002935795	PDT000288768.2	Klebsiella_307	PDS000045391.1	2015	USA	PRJNA433394
GCA 002935785	PDT000288769.2	Klebsiella_307	PDS000045391.1	2014	USA	PRJNA433394
GCA 022169335	PDT000849108.1	Klebsiella_-	PDS000056158.28	2014	Spain	PRJNA626430
GCA 022169285	PDT000849110.1	Klebsiella_-	PDS000056158.28	2014	Spain	PRJNA626430
GCA 030910885	PDT001865911.1	Klebsiella_307		2018	Poland	PRJNA1005055
GCA 004803335	PDT000495889.1	Klebsiella_307		nan	Egypt	PRJNA508509
GCA 043203495	PDT002421097.1	Klebsiella_307	PDS000201306.1	2021	Israel	PRJNA1170061
GCA 043203595	PDT002421099.1	Klebsiella_307	PDS000201306.1	2021	Israel	PRJNA1170061
GCA 043203475	PDT002421096.1	Klebsiella_307	PDS000201306.1	2021	Israel	PRJNA1170061
GCA 043203365	PDT002421094.1	Klebsiella_307	PDS000201306.1	2021	Israel	PRJNA1170061
GCA 043203615	PDT002421100.1	Klebsiella_307	PDS000201306.1	2021	Israel	PRJNA1170061
GCA 043203255	PDT002421091.1	Klebsiella_307	PDS000201306.1	2021	Israel	PRJNA1170061
GCA 043203655	PDT002421101.1	Klebsiella_307	PDS000201306.1	2021	Israel	PRJNA1170061
GCA 043203355	PDT002421093.1	Klebsiella_307	PDS000201306.1	2021	Israel	PRJNA1170061
GCA 043203555	PDT002421098.1	Klebsiella_307	PDS000201306.1	2021	Israel	PRJNA1170061
GCA 043203675	PDT002421102.1	Klebsiella_307	PDS000201306.1	2021	Israel	PRJNA1170061
GCA 017584335	PDT000991756.1	Klebsiella_307		2019	Germany	PRJNA644257
GCA 900205365	PDT000250260.1	Klebsiella_307	PDS000075200.1	nan	NA	PRJEB22081
GCA 900205375	PDT000250262.1	Klebsiella_307	PDS000075200.1	nan	NA	PRJEB22081
GCA 900559965	PDT000595217.1	Klebsiella_307	PDS000045377.17	nan	United Kingdom	PRJEB28457
GCA 900511845	PDT000369466.2	Klebsiella_307	PDS000045377.17	2013	Malta	PRJEB10018
GCA 900511945	PDT000369469.2	Klebsiella_307	PDS000045377.17	2013	Malta	PRJEB10018
GCA 022192305	PDT000715264.1	Klebsiella_307	PDS000189621.16	2011	United Kingdom	PRJNA564424
GCA 022191035	PDT000715344.1	Klebsiella_-	PDS000189621.16	2011	United Kingdom	PRJNA564424
GCA 900560255	PDT000595245.1	Klebsiella_307	PDS000045377.17	nan	United Kingdom	PRJEB28457
GCA 900560345	PDT000595254.1	Klebsiella_307	PDS000045377.17	nan	United Kingdom	PRJEB28457
GCA 900560715	PDT000595291.1	Klebsiella_307	PDS000045377.17	nan	United Kingdom	PRJEB28457
GCA 900538165	PDT000595130.1	Klebsiella_307	PDS000045377.17	nan	United Kingdom	PRJEB28457
GCA 900560645	PDT000595284.1	Klebsiella_307	PDS000045377.17	nan	United Kingdom	PRJEB28457
GCA 900560385	PDT000595258.1	Klebsiella_307	PDS000045377.17	nan	United Kingdom	PRJEB28457
GCA 900560495	PDT000595269.1	Klebsiella_307	PDS000045377.17	nan	United Kingdom	PRJEB28457
GCA 043203315	PDT002421092.1	Klebsiella_307		2021	Israel	PRJNA1170061
GCA 043438885	PDT002421674.1	Klebsiella_307	PDS000201459.1	2019	USA	PRJNA288601
GCA 900510505	PDT000369199.1	Klebsiella_307	PDS000045393.5	2014	France	PRJEB10018
GCA 900510355	PDT000369166.1	Klebsiella_307	PDS000045393.5	2013	France	PRJEB10018
GCA 905330915	PDT001185183.2	Klebsiella_307	PDS000054607.22	2018	Spain	PRJEB42440
GCA 905335725	PDT001185259.2	Klebsiella_307	PDS000054607.22	2018	Spain	PRJEB42440
GCA 900500995	PDT000369812.1	Klebsiella_307	PDS000054607.22	2013	Spain	PRJEB10018
GCA 022022795	PDT000947366.1	Klebsiella_307	PDS000054607.22	nan	Spain	PRJEB37504
GCA 028573495	PDT001610646.1	Klebsiella_-	PDS000054607.22	2020	Spain	PRJEB57245
GCA 028564515	PDT001610645.1	Klebsiella_-	PDS000054607.22	2020	Spain	PRJEB57245
GCA 022063565	PDT000876076.1	Klebsiella_307	PDS000074878.9	2013	USA	PRJNA376414
GCA 047275925	PDT002596257.1	Klebsiella_307	PDS000074878.9	2018	Australia	PRJNA674961
GCA 022193645	PDT000715179.1	Klebsiella_307	PDS000074878.9	2012	United Kingdom	PRJNA564424
GCA 022196905	PDT000714999.1	Klebsiella_307	PDS000074878.9	2012	United Kingdom	PRJNA564424
GCA 022197965	PDT000714936.1	Klebsiella_307	PDS000074878.9	2012	United Kingdom	PRJNA564424
GCA 022167065	PDT000849238.1	Klebsiella_-	PDS000072661.1	2013	Spain	PRJNA626430
GCA 022167085	PDT000849237.1	Klebsiella_-	PDS000072661.1	2013	Spain	PRJNA626430
GCA 019797985	PDT001120285.1	Klebsiella_307		2012	Norway	PRJNA723772
GCA 024609505	PDT001385477.1	Klebsiella_307	PDS000199423.1	2016	Tunisia	PRJNA841857
GCA 022209045	PDT000636254.1	Klebsiella_307	PDS000045377.17	2015	France	PRJNA551371
GCA 022110815	PDT001244432.1	Klebsiella_307	PDS000104496.1	2015	United Kingdom	PRJNA788733
GCA 022106775	PDT001244718.1	Klebsiella_307	PDS000104496.1	2016	United Kingdom	PRJNA788733
GCA 021940235	PDT001049086.1	Klebsiella_307		2018	Australia	PRJNA857533
GCA 043859835	**HBH_261_13**	Klebsiella_307		2013	Tunisia	PRJNA1160997
GCA 043863025	**HBH_623_15**	Klebsiella_-	PDS000199423.1	2015	Tunisia	PRJNA1160997
GCA 043860235	**HBH_4002_14**	Klebsiella_307	PDS000199421.1	2014	Tunisia	PRJNA1160997
GCA 043863065	**HBH_3881_15**	Klebsiella_307	PDS000199421.1	2015	Tunisia	PRJNA1160997
GCA 046076765	PDT002536505.1	Klebsiella_307	PDS000213377.1	2020	Czechia	PRJNA1130981
GCA 046076985	PDT002536515.1	Klebsiella_307	PDS000213377.1	2020	Czechia	PRJNA1130981
GCA 046073585	PDT002536495.1	Klebsiella_307	PDS000213377.1	2020	Czechia	PRJNA1130981
GCA 046073515	PDT002536491.1	Klebsiella_307	PDS000213377.1	2020	Czechia	PRJNA1130981
GCA 046071825	PDT002536411.1	Klebsiella_307	PDS000213377.1	2020	Czechia	PRJNA1130981
GCA 046073565	PDT002536494.1	Klebsiella_307	PDS000213377.1	2020	Czechia	PRJNA1130981
GCA 046073325	PDT002536482.1	Klebsiella_307	PDS000213377.1	2020	Czechia	PRJNA1130981
GCA 046073605	PDT002536496.1	Klebsiella_307	PDS000213377.1	2020	Czechia	PRJNA1130981
GCA 046070845	PDT002536362.1	Klebsiella_307	PDS000213377.1	2021	Czechia	PRJNA1130981
GCA 046070985	PDT002536369.1	Klebsiella_307	PDS000213377.1	2021	Czechia	PRJNA1130981
GCA 046071045	PDT002536372.1	Klebsiella_307	PDS000213377.1	2021	Czechia	PRJNA1130981
GCA 046067445	PDT002536353.1	Klebsiella_307	PDS000213377.1	2021	Czechia	PRJNA1130981
GCA 046063025	PDT002536204.1	Klebsiella_307	PDS000213377.1	2021	Czechia	PRJNA1130981
GCA 046067405	PDT002536351.1	Klebsiella_307	PDS000213377.1	2021	Czechia	PRJNA1130981
GCA 046070865	PDT002536363.1	Klebsiella_307	PDS000213377.1	2021	Czechia	PRJNA1130981
GCA 046065125	PDT002536236.1	Klebsiella_307	PDS000213377.1	2021	Czechia	PRJNA1130981
GCA 046067285	PDT002536345.1	Klebsiella_307	PDS000213377.1	2021	Czechia	PRJNA1130981
GCA 046066285	PDT002536293.1	Klebsiella_307	PDS000213377.1	2021	Czechia	PRJNA1130981
GCA 046067465	PDT002536354.1	Klebsiella_307	PDS000213377.1	2021	Czechia	PRJNA1130981
GCA 046066205	PDT002536289.1	Klebsiella_307	PDS000213377.1	2021	Czechia	PRJNA1130981
GCA 046062685	PDT002536184.1	Klebsiella_307	PDS000213377.1	2021	Czechia	PRJNA1130981
GCA 046067345	PDT002536348.1	Klebsiella_307	PDS000213377.1	2021	Czechia	PRJNA1130981

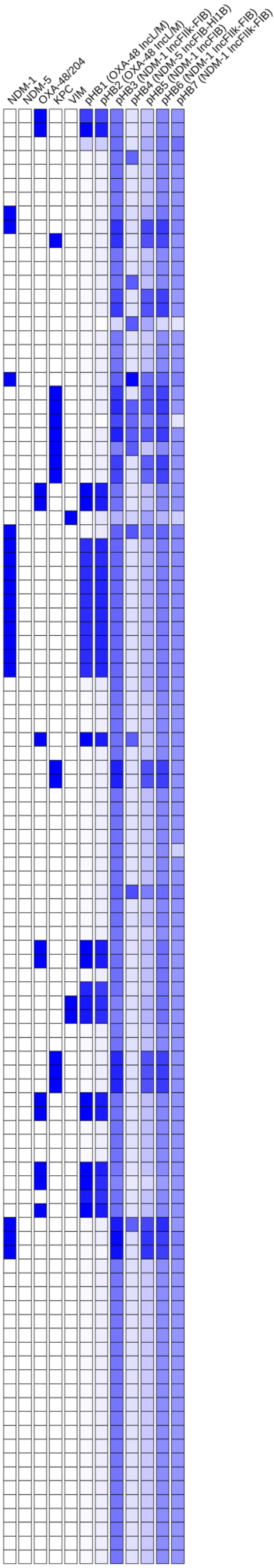

Tree 15: GCA_043863145

Tree scale: 0.1

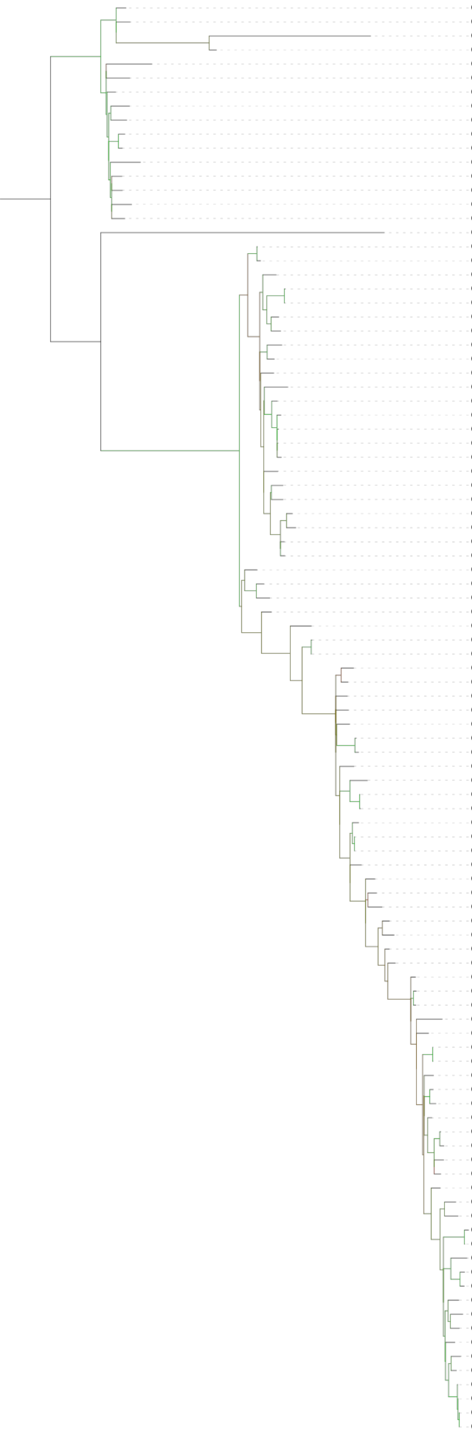

	Isolate	MLST	SNP Cluster	Collection Year	Country	BioProject
GCA 045488675	PDT002191062.1	Klebsiella_45		2017	USA	PRJNA1054115
GCA 044876025	PDT002465878.1	Klebsiella_45		nan	United Kingdom	PRJNA1181535
GCA 033106265	PDT001960955.1	Klebsiella_-		2017	USA	PRJNA648389
GCA 017975385	PDT001007098.1	Klebsiella_45		2017	Italy	PRJNA687927
GCA 043863145	**HBH_3610_16**	Klebsiella_45		2016	Tunisia	PRJNA1160997
GCA 900774385	PDT000489678.1	Klebsiella_45		nan	NA	PRJEB31361
GCA 033864515	PDT001992530.1	Klebsiella_45		2016	Canada	PRJNA532924
GCA 045431075	PDT002498126.1	Klebsiella_45		2024	USA	PRJNA288601
GCA 016904315	PDT000967858.1	Klebsiella_45		nan	USA	PRJNA231221
GCA 036904995	PDT002094487.1	Klebsiella_45		2024	USA	PRJNA1004324
GCA 030360325	PDT001792582.1	Klebsiella_45		2016	Mexico	PRJNA985271
GCA 021264245	PDT001211219.1	Klebsiella_45		nan	USA	PRJNA788509
GCA 900774825	PDT000489722.1	Klebsiella_45		nan	NA	PRJEB31361
GCA 032286605	PDT001911293.1	Klebsiella_45		2021	USA	PRJNA978102
GCA 030435275	PDT001815462.1	Klebsiella_45		2020	Chile	PRJNA991619
GCA 001033745	PDT000068098.1	Klebsiella_45		2014	NA	PRJNA271899
GCA 002192315	PDT000218722.1	Klebsiella_45	PDS000006905.10	nan	NA	PRJNA292902
GCA 043384065	PDT002281230.1	Klebsiella_45	PDS000198705.1	2007	Norway	PRJEB27256
GCA 041920645	PDT002382459.2	Klebsiella_45	PDS000198705.1	2007	Norway	PRJNA1098507
GCA 044390755	PDT002305105.1	Klebsiella_45		nan	USA	PRJNA271899
GCA 023315955	PDT001299601.1	Klebsiella_45	PDS000109086.2	2019	USA	PRJNA789565
GCA 023462775	PDT001296751.2	Klebsiella_-	PDS000109086.2	2018	USA	PRJNA789565
GCA 031044555	PDT001744219.2	Klebsiella_-	PDS000146617.5	2022	USA	PRJNA278886
GCA 033870595	PDT001992432.1	Klebsiella_45	PDS000008541.10	2015	Canada	PRJNA532924
GCA 900508325	PDT000370739.1	Klebsiella_45	PDS000007035.4	2014	Poland	PRJEB10018
GCA 032136875	PDT001903047.1	Klebsiella_45		2017	Norway	PRJEB48268
GCA 022312935	PDT000136333.2	Klebsiella_45		2011	United Kingdom	PRJNA306133
GCA 015719165	PDT000903580.1	Klebsiella_45		2018	USA	PRJNA475751
GCA 037385665	PDT002117108.1	Klebsiella_45	PDS000179055.2	2016	USA	PRJNA288601
GCA 022073085	PDT000875575.1	Klebsiella_45	PDS000052944.18	2013	USA	PRJNA376414
GCA 022211745	PDT000630652.1	Klebsiella_45	PDS000052944.18	2013	Canada	PRJNA564992
GCA 030019695	PDT001737461.1	Klebsiella_45	PDS000052944.18	2013	USA	PRJNA395086
GCA 030795985	PDT001705768.1	Klebsiella_45	PDS000052944.18	2019	Japan	PRJDB10842
GCA 032070025	PDT001905081.1	Klebsiella_45		2017	Norway	PRJEB48268
GCA 032134355	PDT001903162.1	Klebsiella_45		2017	Norway	PRJEB48268
GCA 003401365	PDT000364547.1	Klebsiella_45	PDS000104562.1	2014	USA	PRJNA436373
GCA 900516505	PDT000369790.1	Klebsiella_45		2013	Spain	PRJEB10018
GCA 031034435	PDT001726552.3	Klebsiella_45		2018	USA	PRJNA288601
GCA 043860055	**HBH_2308_14**	Klebsiella_45		2014	Tunisia	PRJNA1160997
GCA 021980835	PDT001009148.1	Klebsiella_-		2018	Italy	PRJEB27342
GCA 033103885	PDT001961101.1	Klebsiella_-	PDS000106393.3	2007	Spain	PRJEB41835
GCA 000826795	PDT000049482.1	Klebsiella_45		2012	USA	PRJNA261775
GCA 004322955	PDT000473852.1	Klebsiella_45		2014	USA	PRJNA279669
GCA 016527075	PDT000929082.1	Klebsiella_45		nan	Portugal	PRJNA602991
GCA 024430915	PDT001366287.1	Klebsiella_45		2019	Guadeloupe	PRJNA778230
GCA 046126435	PDT002538861.1	Klebsiella_45	PDS000212921.2	2018	New Zealand	PRJNA1102395
GCA 046126295	PDT002538872.1	Klebsiella_45	PDS000212921.2	2018	New Zealand	PRJNA1102395
GCA 044387895	PDT002305181.1	Klebsiella_45		nan	USA	PRJNA271899
GCA 043332935	PDT002278352.1	Klebsiella_45		2018	USA	PRJNA1106484
GCA 021928855	PDT001091678.1	Klebsiella_45		2015	Norway	PRJEB27256
GCA 036890025	PDT002094458.1	Klebsiella_45		2018	France	PRJNA1033693
GCA 033104365	PDT001961051.1	Klebsiella_45		2019	USA	PRJNA648389
GCA 016643595	PDT000933596.1	Klebsiella_3031	PDS000078891.2	nan	Portugal	PRJNA602991
GCA 016643105	PDT000933575.1	Klebsiella_3031	PDS000078891.2	nan	Portugal	PRJNA602991
GCA 900515915	PDT000369662.1	Klebsiella_45		2013	Italy	PRJEB10018
GCA 016797645	PDT000948126.1	Klebsiella_45		2013	USA	PRJNA395086
GCA 022079815	PDT000875223.1	Klebsiella_-	PDS000074859.1	2014	USA	PRJNA376414
GCA 022079735	PDT000875226.1	Klebsiella_-	PDS000074859.1	2014	USA	PRJNA376414
GCA 040191135	PDT002208199.1	Klebsiella_45		nan	Saudi Arabia	PRJNA1116866
GCA 046448265	PDT002554279.1	Klebsiella_45	PDS000214277.1	2014	Russia	PRJNA1196851
GCA 046449325	PDT002554332.1	Klebsiella_45	PDS000214277.1	2018	Russia	PRJNA1196851
GCA 046764395	PDT002587012.1	Klebsiella_45	PDS000215653.1	2018	China	PRJNA1210003
GCA 036995725	PDT002098826.1	Klebsiella_45		2016	China	PRJNA1003408
GCA 019677585	PDT001112719.1	Klebsiella_45	PDS000106675.2	2016	Germany	PRJNA393144
GCA 900514325	PDT000369443.1	Klebsiella_45	PDS000162236.1	2014	Italy	PRJEB10018
GCA 022285865	PDT000431391.1	Klebsiella_45	PDS000067092.3	2017	USA	PRJNA396774
GCA 014901295	PDT000864971.1	Klebsiella_45	PDS000067092.3	nan	USA	PRJNA271899
GCA 022501305	PDT001262315.1	Klebsiella_45		2019	China	PRJNA804657
GCA 023032025	PDT001289296.1	Klebsiella_-		2020	Australia	PRJNA777643
GCA 023739135	PDT001336493.1	Klebsiella_45		2021	China	PRJNA833902
GCA 032045175	PDT001903326.1	Klebsiella_45	PDS000175704.1	2018	Norway	PRJEB48268
GCA 036949225	PDT002096667.2	Klebsiella_45	PDS000175704.1	2018	Belgium	PRJNA927131
GCA 022098395	PDT001096792.3	Klebsiella_45		2021	USA	PRJNA288601
GCA 900775615	PDT000489801.1	Klebsiella_45	PDS000100089.1	nan	NA	PRJEB31361
GCA 036890125	PDT002094463.1	Klebsiella_45	PDS000192605.1	2018	France	PRJNA1033693
GCA 041203925	PDT002317887.1	Klebsiella_45	PDS000192605.1	2019	France	PRJNA1132810
GCA 044322325	PDT002306240.1	Klebsiella_45		nan	USA	PRJNA271899
GCA 956480335	PDT001864109.1	Klebsiella_45		2018	Spain	PRJEB63349
GCA 021975415	PDT001011543.1	Klebsiella_45		2015	Norway	PRJEB42350
GCA 964300705	PDT002500299.1	Klebsiella_45	PDS000106675.2	2019	Germany	PRJEB81699
GCA 022114195	PDT001245323.1	Klebsiella_45	PDS000104481.1	2019	Netherlands	PRJNA754858
GCA 022114235	PDT001245311.1	Klebsiella_45	PDS000104481.1	2019	Netherlands	PRJNA754858
GCA 022184005	PDT000723800.1	Klebsiella_45		2018	Italy	PRJEB27342
GCA 022200125	PDT000706832.1	Klebsiella_-	PDS000070441.1	2018	Italy	PRJEB27342
GCA 022987545	PDT001286508.1	Klebsiella_45		2012	Germany	PRJNA789142
GCA 022430345	PDT001256202.1	Klebsiella_45	PDS000105716.1	2020	USA	PRJNA325243
GCA 016903895	PDT000967411.1	Klebsiella_45		nan	USA	PRJNA231221
GCA 044895205	PDT002451634.2	Klebsiella_45	PDS000206449.1	2021	USA	PRJNA1106484
GCA 043961635	PDT002428766.1	Klebsiella_45	PDS000206449.1	2021	USA	PRJNA1106484
GCA 043709025	PDT002376631.1	Klebsiella_45		2018	Japan	PRJDB14656
GCA 026256265	PDT001849267.1	Klebsiella_45	PDS000074716.5	2013	Australia	PRJNA646837
GCA 003432285	PDT000371002.1	Klebsiella_45	PDS000074716.5	2013	Australia	PRJNA486877
GCA 043569885	PDT002347492.1	Klebsiella_45	PDS000100594.2	2009	Norway	PRJEB27256
GCA 032042675	PDT001903028.1	Klebsiella_45		2018	Norway	PRJEB48268
GCA 900511045	PDT000369071.1	Klebsiella_45		2013	Czechia	PRJEB10018
GCA 022109315	PDT001245091.1	Klebsiella_45		2014	United Kingdom	PRJNA788733
GCA 022285805	PDT000431395.1	Klebsiella_45		2017	USA	PRJNA396774
GCA 022286615	PDT000431357.1	Klebsiella_45		2017	USA	PRJNA396774
GCA 043331735	PDT002278350.1	Klebsiella_45	PDS000178660.3	2019	USA	PRJNA1106484
GCA 022033555	PDT000901854.3	Klebsiella_45	PDS000178660.3	2019	USA	PRJNA288601
GCA 046015795	PDT002530113.1	Klebsiella_45	PDS000178660.3	2018	USA	PRJNA288601
GCA 022033615	PDT000901821.3	Klebsiella_45	PDS000178660.3	2018	USA	PRJNA288601

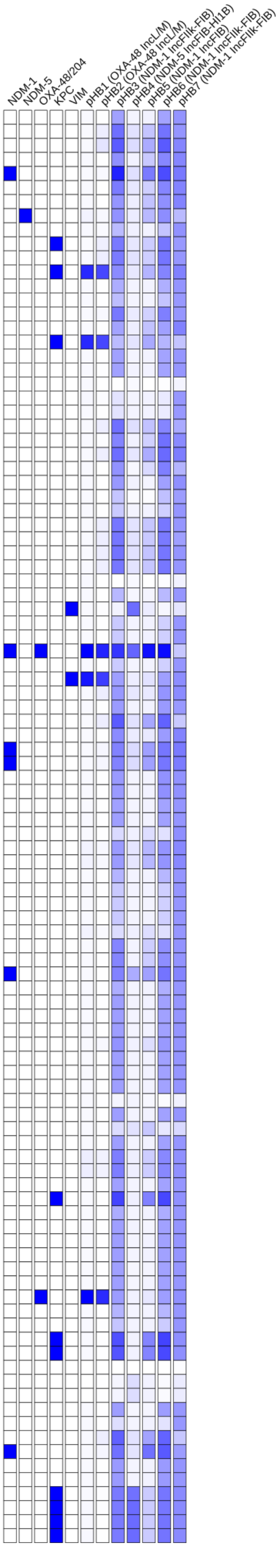

Tree 16: GCA_043863165

Tree scale: 0.1

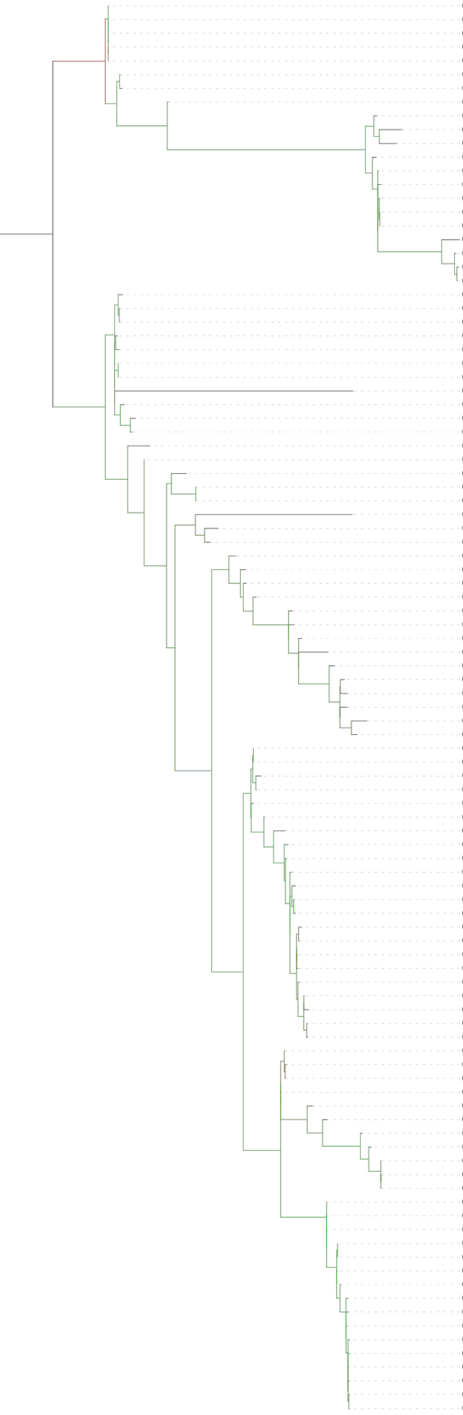

	Isolate	MLST	SNP Cluster	Collection Year	Country	BioProject
GCA 000787995	PDT000045029.1	Klebsiella_395	PDS000112540.1	2006	USA	PRJNA261239
GCA 023894035	PDT001342107.1	Klebsiella_395	PDS000112540.1	2006	USA	PRJNA849970
GCA 023894045	PDT001342108.1	Klebsiella_395	PDS000112540.1	2006	USA	PRJNA849970
GCA 023894015	PDT001342106.1	Klebsiella_395	PDS000112540.1	2006	Germany	PRJNA849970
GCA 023894115	PDT001342111.1	Klebsiella_395	PDS000112540.1	2006	USA	PRJNA849970
GCA 022211805	PDT000630914.1	Klebsiella_395	PDS000053740.1	2013	Canada	PRJNA564992
GCA 022211265	PDT000632287.1	Klebsiella_395	PDS000053740.1	2016	Canada	PRJNA564992
GCA 022147125	PDT001244080.1	Klebsiella_395		2015	United Kingdom	PRJNA788733
GCA 004522415	PDT000484716.1	Klebsiella_395	PDS000044310.6	2018	Lebanon	PRJNA517127
GCA 022109855	PDT001245093.1	Klebsiella_395	PDS000044310.6	2015	United Kingdom	PRJNA788733
GCA 022159705	PDT001243595.1	Klebsiella_395	PDS000044310.6	2015	United Kingdom	PRJNA788733
GCA 004802845	PDT000495873.1	Klebsiella_395	PDS000044310.6	nan	Egypt	PRJNA508509
GCA 000739495	PDT000036761.1	Klebsiella_395	PDS000044310.6	2012	USA	PRJNA248655
GCA 021787745	PDT001227646.1	Klebsiella_395	PDS000044310.6	2014	United Kingdom	PRJNA788733
GCA 043860435	**HBH_560_14**	Klebsiella_395	PDS000044310.6	2014	Tunisia	PRJNA1160997
GCA 043863165	**HBH_4068_16**	Klebsiella_395	PDS000044310.6	2016	Tunisia	PRJNA1160997
GCA 043859975	**HBH_2077_14**	Klebsiella_395	PDS000044310.6	2014	Tunisia	PRJNA1160997
GCA 019800205	PDT001120290.1	Klebsiella_395		nan	Germany	PRJNA546126
GCA 022148845	PDT001244759.1	Klebsiella_395	PDS000044310.6	2015	United Kingdom	PRJNA788733
GCA 022113195	PDT001244412.1	Klebsiella_395	PDS000044310.6	2015	United Kingdom	PRJNA788733
GCA 022111855	PDT001244385.1	Klebsiella_395	PDS000044310.6	2015	United Kingdom	PRJNA788733
GCA 034408345	PDT002029048.1	Klebsiella_395		2017	Vietnam	PRJEB67696
GCA 003115075	PDT000313820.2	Klebsiella_395	PDS000053786.7	2015	Vietnam	PRJDB6655
GCA 003115115	PDT000313822.2	Klebsiella_395	PDS000053786.7	2013	Vietnam	PRJDB6655
GCA 014284735	PDT000822583.1	Klebsiella_395		2011	China	PRJNA657553
GCA 016613895	PDT000932759.1	Klebsiella_395		nan	NA	PRJNA550041
GCA 019492045	PDT001107669.1	Klebsiella_395	PDS000114341.1	2013	China	PRJNA742185
GCA 024396895	PDT001370147.1	Klebsiella_395	PDS000114341.1	2013	China	PRJNA858218
GCA 022170985	PDT000787915.1	Klebsiella_395		2015	China	PRJNA595047
GCA 022273875	PDT000433174.1	Klebsiella_395	PDS000040454.8	2014	Vietnam	PRJDB5317
GCA 036993645	PDT002098722.1	Klebsiella_395	PDS000190144.1	2016	China	PRJNA1003408
GCA 040990615	PDT002280004.1	Klebsiella_395	PDS000190144.1	nan	China	PRJNA1021041
GCA 045781975	PDT002474505.1	Klebsiella_395		2017	India	PRJNA939406
GCA 027615305	PDT001558363.1	Klebsiella_395		2006	Bangladesh	PRJNA845975
GCA 022181705	PDT000730841.1	Klebsiella_395		2017	Canada	PRJNA564992
GCA 022169685	PDT000847262.1	Klebsiella_395	PDS000072337.1	2018	Myanmar	PRJDB5126
GCA 022169705	PDT000847261.1	Klebsiella_395	PDS000072337.1	2018	Myanmar	PRJDB5126
GCA 037477765	PDT002121536.1	Klebsiella_395		2022	Russia	PRJNA945360
GCA 926498335	PDT001360402.1	Klebsiella_395		2014	Belarus	PRJEB49683
GCA 926291145	PDT001360457.1	Klebsiella_395		2014	Belarus	PRJEB49683
GCA 900511835	PDT000369119.1	Klebsiella_395	PDS000077831.2	2014	Estonia	PRJEB10018
GCA 021774805	PDT001229737.3	Klebsiella_395		2021	USA	PRJNA288601
GCA 956476815	PDT001861751.1	Klebsiella_395	PDS000156671.1	2017	Romania	PRJEB63349
GCA 009710125	PDT000637194.1	Klebsiella_395		2018/2019	Romania	PRJNA579879
GCA 900512835	PDT000369592.1	Klebsiella_395		2013	Italy	PRJEB10018
GCA 022331865	PDT000537925.1	Klebsiella_395		2017	Russia	PRJNA522420
GCA 046448825	PDT002554307.1	Klebsiella_395		2014	Russia	PRJNA1196851
GCA 032289265	PDT001911265.1	Klebsiella_395		2021	USA	PRJNA978102
GCA 033900425	PDT002000548.1	Klebsiella_395		nan	Germany	PRJNA1036598
GCA 036236835	PDT002066783.1	Klebsiella_395		2022	Russia	PRJNA729628
GCA 009661535	PDT000628437.2	Klebsiella_395		2017	Russia	PRJNA580263
GCA 033559575	PDT001977490.1	Klebsiella_395		2018	Germany	PRJEB67440
GCA 926283225	PDT001360514.1	Klebsiella_395		2014	Russia	PRJEB49683
GCA 015209465	PDT000878356.1	Klebsiella_395		2013	Russia	PRJNA269675
GCA 900503695	PDT000370125.1	Klebsiella_395	PDS000041774.4	2014	Luxembourg	PRJEB10018
GCA 900517805	PDT000369934.1	Klebsiella_395	PDS000041774.4	2013	Luxembourg	PRJEB10018
GCA 900510525	PDT000369205.1	Klebsiella_395	PDS000041774.4	2014	France	PRJEB10018
GCA 025049195	PDT001384867.1	Klebsiella_395	PDS000041774.4	2012	France	PRJEB50837
GCA 022230465	PDT000445980.1	Klebsiella_395	PDS000041774.4	2011	Canada	PRJNA390933
GCA 043980005	PDT002411906.1	Klebsiella_395	PDS000041774.4	2015	Canada	PRJNA855907
GCA 040011555	PDT002187503.1	Klebsiella_395	PDS000045400.2	2011	Netherlands	PRJNA1116077
GCA 040011675	PDT002187508.1	Klebsiella_395	PDS000045400.2	2011	Netherlands	PRJNA1116077
GCA 040012355	PDT002187521.1	Klebsiella_395	PDS000045400.2	2011	Netherlands	PRJNA1116077
GCA 040012015	PDT002187515.1	Klebsiella_395	PDS000045400.2	2011	Netherlands	PRJNA1116077
GCA 040012295	PDT002187519.1	Klebsiella_395	PDS000045400.2	2014	Netherlands	PRJNA1116077
GCA 040012335	PDT002187520.1	Klebsiella_395	PDS000045400.2	2011	Netherlands	PRJNA1116077
GCA 040011775	PDT002187512.1	Klebsiella_395	PDS000045400.2	2011	Netherlands	PRJNA1116077
GCA 040015455	PDT002187524.1	Klebsiella_395	PDS000045400.2	2011	Netherlands	PRJNA1116077
GCA 040011795	PDT002187513.1	Klebsiella_395	PDS000045400.2	2011	Netherlands	PRJNA1116077
GCA 040011455	PDT002187500.1	Klebsiella_395	PDS000045400.2	2011	Netherlands	PRJNA1116077
GCA 040011235	PDT002187498.1	Klebsiella_395	PDS000045400.2	2011	Netherlands	PRJNA1116077
GCA 040012075	PDT002187517.1	Klebsiella_395	PDS000045400.2	2011	Netherlands	PRJNA1116077
GCA 040011695	PDT002187509.1	Klebsiella_395	PDS000045400.2	2011	Netherlands	PRJNA1116077
GCA 040011735	PDT002187510.1	Klebsiella_395	PDS000045400.2	2011	Netherlands	PRJNA1116077
GCA 040011635	PDT002187506.1	Klebsiella_395	PDS000045400.2	2011	Netherlands	PRJNA1116077
GCA 040011755	PDT002187511.1	Klebsiella_395	PDS000045400.2	2011	Netherlands	PRJNA1116077
GCA 900508065	PDT000370653.1	Klebsiella_395	PDS000036210.15	2014	Turkey	PRJEB10018
GCA 001902355	PDT000171848.1	Klebsiella_395	PDS000036210.15	2015	Germany	PRJNA329409
GCA 902155915	PDT000663172.1	Klebsiella_395	PDS000036210.15	2014	Turkey	PRJEB33308
GCA 900507325	PDT000370691.1	Klebsiella_395	PDS000036210.15	2013	Turkey	PRJEB10018
GCA 926279955	PDT001360434.1	Klebsiella_395	PDS000036210.15	2016	Russia	PRJEB49683
GCA 947390255	PDT001529136.1	Klebsiella_395	PDS000036210.15	2017	United Kingdom	PRJEB57368
GCA 040981925	PDT002279474.1	Klebsiella_395	PDS000095854.2	2020	China	PRJNA836393
GCA 040981965	PDT002279476.1	Klebsiella_395	PDS000095854.2	2021	China	PRJNA836393
GCA 040982035	PDT002279479.1	Klebsiella_395	PDS000095854.2	2020	China	PRJNA836393
GCA 040981975	PDT002279477.1	Klebsiella_395	PDS000095854.2	2020	China	PRJNA836393
GCA 040981835	PDT002279472.1	Klebsiella_395	PDS000095854.2	2020	China	PRJNA836393
GCA 900508125	PDT000370663.1	Klebsiella_395	PDS000036210.15	2014	Turkey	PRJEB10018
GCA 900507975	PDT000370633.1	Klebsiella_395	PDS000036210.15	2014	Turkey	PRJEB10018
GCA 900507395	PDT000370582.1	Klebsiella_395	PDS000036210.15	2013	Turkey	PRJEB10018
GCA 900508105	PDT000370659.1	Klebsiella_395	PDS000036210.15	2014	Turkey	PRJEB10018
GCA 900508075	PDT000370656.1	Klebsiella_395	PDS000036210.15	2014	Turkey	PRJEB10018
GCA 900507365	PDT000370696.1	Klebsiella_395	PDS000036210.15	2014	Turkey	PRJEB10018
GCA 033901175	PDT002000559.1	Klebsiella_395	PDS000036210.15	nan	Germany	PRJNA1036598
GCA 022427005	PDT001256974.1	Klebsiella_395	PDS000036210.15	2015	Turkey	PRJNA809152
GCA 023022825	PDT001289750.1	Klebsiella_395	PDS000036210.15	2018	Turkey	PRJNA789336
GCA 956481615	PDT001864181.1	Klebsiella_395	PDS000036210.15	2018	Turkey	PRJEB63349
GCA 023021665	PDT001289808.1	Klebsiella_395	PDS000036210.15	2019	Turkey	PRJNA789336
GCA 023024455	PDT001289655.1	Klebsiella_395	PDS000036210.15	2019	Turkey	PRJNA789336
GCA 023025745	PDT001289601.1	Klebsiella_395	PDS000036210.15	2019	Turkey	PRJNA789336
GCA 956482755	PDT001864185.1	Klebsiella_395	PDS000036210.15	2018	Turkey	PRJEB63349
GCA 018447595	PDT001047583.1	Klebsiella_395	PDS000036210.15	2018	Germany	PRJNA692829
GCA 018444225	PDT001046630.1	Klebsiella_395	PDS000036210.15	2018	Germany	PRJNA692829

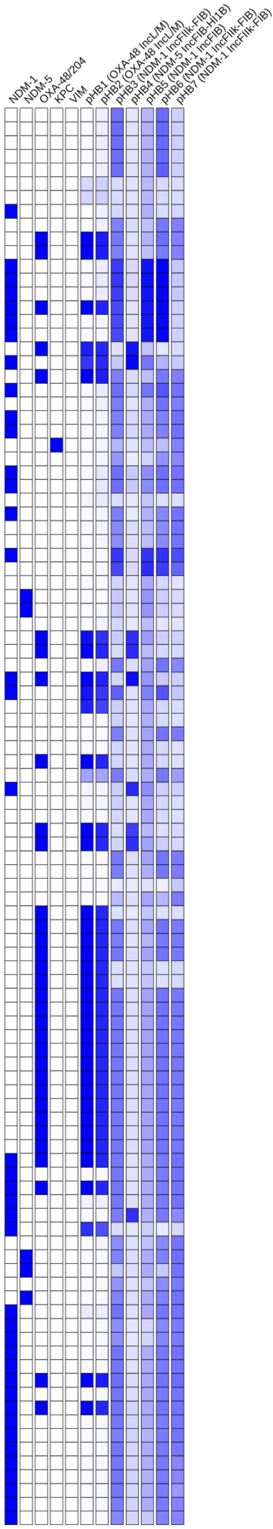

Tree 17: GCA_043863205

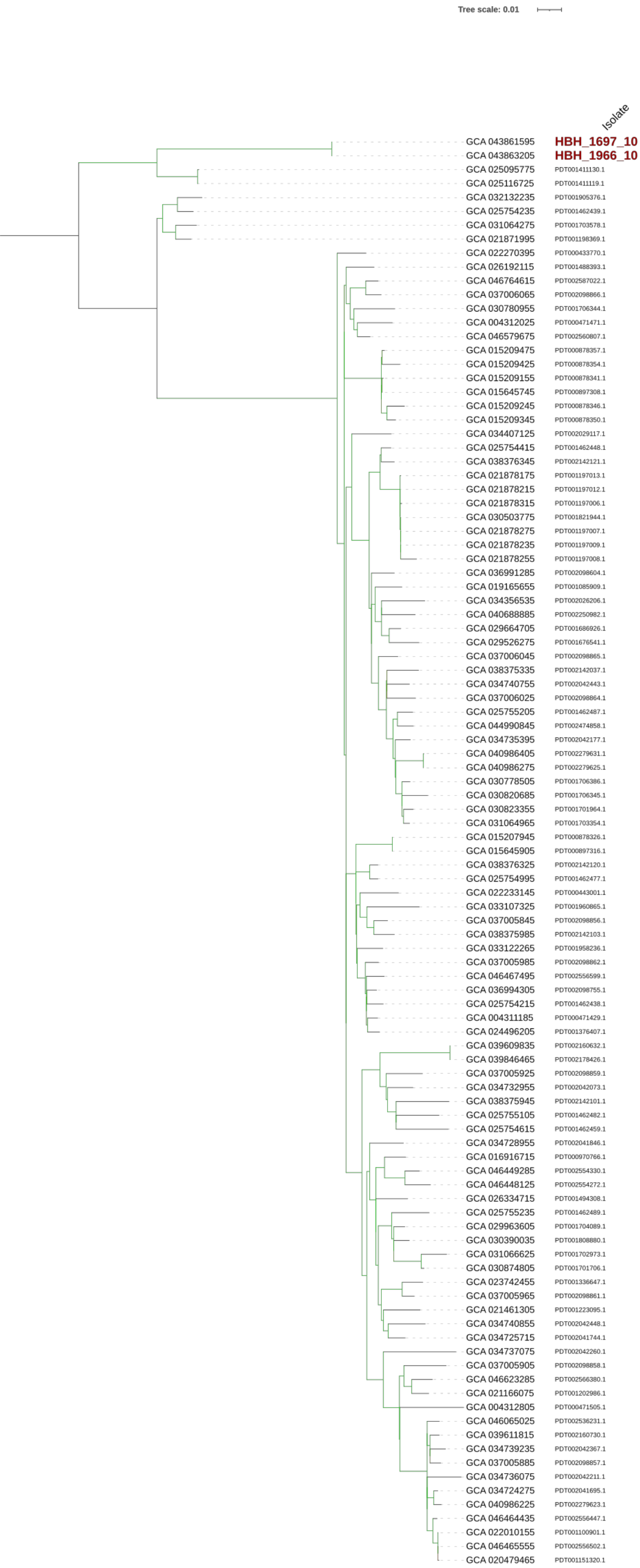

	Isolate	MLST	SNP Cluster	Collection Year	Country	BioProject
GCA 043861595	**HBH_1697_10**	Klebsiella_218	PDS000199420.1	2010	Tunisia	PRJNA1160997
GCA 043863205	**HBH_1966_10**	Klebsiella_218	PDS000199420.1	2010	Tunisia	PRJNA1160997
GCA 025095775	PDT001411130.1	Klebsiella_-	PDS000117560.1	2020	Singapore	PRJNA577535
GCA 025116725	PDT001411119.1	Klebsiella_-	PDS000117560.1	2020	Singapore	PRJNA577535
GCA 032132235	PDT001905376.1	Klebsiella_218		2020	Japan	PRJDB16654
GCA 025754235	PDT001462439.1	Klebsiella_218		2012	China	PRJNA742521
GCA 031064275	PDT001703578.1	Klebsiella_218		2019	Japan	PRJDB10842
GCA 021871995	PDT001198369.1	Klebsiella_218		2015	Japan	PRJDB12075
GCA 022270395	PDT000433770.1	Klebsiella_-		2015	Singapore	PRJNA398288
GCA 026192115	PDT001488393.1	Klebsiella_218		2020	India	PRJNA663753
GCA 046764615	PDT002587022.1	Klebsiella_218		2017	China	PRJNA1210003
GCA 037006065	PDT002098866.1	Klebsiella_218		2020	China	PRJNA957912
GCA 030780955	PDT001706344.1	Klebsiella_218		2020	Japan	PRJDB10842
GCA 004312025	PDT000471471.1	Klebsiella_218		2014	Japan	PRJDB7163
GCA 046579675	PDT002560807.1	Klebsiella_218		2022	Germany	PRJNA1001661
GCA 015209475	PDT000878357.1	Klebsiella_218	PDS000074977.2	2013	Russia	PRJNA269675
GCA 015209425	PDT000878354.1	Klebsiella_218		2013	Russia	PRJNA269675
GCA 015209155	PDT000878341.1	Klebsiella_218	PDS000074977.2	2013	Russia	PRJNA269675
GCA 015645745	PDT000897308.1	Klebsiella_218	PDS000074977.2	2013	Russia	PRJNA269675
GCA 015209245	PDT000878346.1	Klebsiella_218		2013	Russia	PRJNA269675
GCA 015209345	PDT000878350.1	Klebsiella_218	PDS000074977.2	2013	Russia	PRJNA269675
GCA 034407125	PDT002029117.1	Klebsiella_218		2019	India	PRJNA1045567
GCA 025754415	PDT001462448.1	Klebsiella_218		2010	China	PRJNA742521
GCA 038376345	PDT002142121.1	Klebsiella_218		2021	China	PRJNA1081623
GCA 021878175	PDT001197013.1	Klebsiella_218	PDS000100586.2	2019	China	PRJNA733872
GCA 021878215	PDT001197012.1	Klebsiella_218	PDS000100586.2	2019	China	PRJNA733872
GCA 021878315	PDT001197006.1	Klebsiella_218	PDS000100586.2	2019	China	PRJNA733872
GCA 030503775	PDT001821944.1	Klebsiella_218	PDS000100586.2	2019	China	PRJNA732824
GCA 021878275	PDT001197007.1	Klebsiella_218	PDS000100586.2	2019	China	PRJNA733872
GCA 021878235	PDT001197009.1	Klebsiella_218	PDS000100586.2	2019	China	PRJNA733872
GCA 021878255	PDT001197008.1	Klebsiella_218	PDS000100586.2	2019	China	PRJNA733872
GCA 036991285	PDT002098604.1	Klebsiella_218		2015	China	PRJNA1003408
GCA 019165655	PDT001085909.1	Klebsiella_218		2017	China	PRJNA736556
GCA 034356535	PDT002026206.1	Klebsiella_-		2011	China	PRJNA1050414
GCA 040688885	PDT002250982.1	Klebsiella_218		2019	China	PRJNA1131666
GCA 029664705	PDT001686926.1	Klebsiella_218		2022	China	PRJNA937035
GCA 029526275	PDT001676541.1	Klebsiella_218		2019	China	PRJNA810290
GCA 037006045	PDT002098865.1	Klebsiella_218		2021	China	PRJNA957912
GCA 038375335	PDT002142037.1	Klebsiella_218		2022	China	PRJNA1081623
GCA 034740755	PDT002042443.1	Klebsiella_218		2021	China	PRJNA1050895
GCA 037006025	PDT002098864.1	Klebsiella_218		2020	China	PRJNA957912
GCA 025755205	PDT001462487.1	Klebsiella_218		2015	China	PRJNA742521
GCA 044990845	PDT002474858.1	Klebsiella_218		2020	China	PRJNA1181777
GCA 034735395	PDT002042177.1	Klebsiella_218		2021	China	PRJNA1050895
GCA 040986405	PDT002279631.1	Klebsiella_218	PDS000190124.1	nan	China	PRJNA1021041
GCA 040986275	PDT002279625.1	Klebsiella_218	PDS000190124.1	nan	China	PRJNA1021041
GCA 030778505	PDT001706386.1	Klebsiella_218		2020	Japan	PRJDB10842
GCA 030820685	PDT001706345.1	Klebsiella_218		2020	Japan	PRJDB10842
GCA 030823355	PDT001701964.1	Klebsiella_-		2019	Japan	PRJDB10842
GCA 031064965	PDT001703354.1	Klebsiella_218		2019	Japan	PRJDB10842
GCA 015207945	PDT000878326.1	Klebsiella_218	PDS000076457.1	2014	Russia	PRJNA269675
GCA 015645905	PDT000897316.1	Klebsiella_218	PDS000076457.1	2014	Russia	PRJNA269675
GCA 038376325	PDT002142120.1	Klebsiella_218		2021	China	PRJNA1081623
GCA 025754995	PDT001462477.1	Klebsiella_218		2015	China	PRJNA742521
GCA 022233145	PDT000443001.1	Klebsiella_218		2011	Vietnam	PRJEB26814
GCA 033107325	PDT001960865.1	Klebsiella_218		2018	Vietnam	PRJEB64408
GCA 037005845	PDT002098856.1	Klebsiella_218		2022	China	PRJNA957912
GCA 038375985	PDT002142103.1	Klebsiella_218		2021	China	PRJNA1081623
GCA 033122265	PDT001958236.1	Klebsiella_218		2015	Norway	PRJEB27256
GCA 037005985	PDT002098862.1	Klebsiella_218		2020	China	PRJNA957912
GCA 046467495	PDT002556599.1	Klebsiella_5943		nan	NA	PRJNA786399
GCA 036994305	PDT002098755.1	Klebsiella_218		2009	China	PRJNA1003408
GCA 025754215	PDT001462438.1	Klebsiella_218		2012	China	PRJNA742521
GCA 004311185	PDT000471429.1	Klebsiella_218		2014	Japan	PRJDB7163
GCA 024496205	PDT001376407.1	Klebsiella_218		2022	China	PRJNA861119
GCA 039609835	PDT002160632.1	Klebsiella_218	PDS000183511.1	2023	NA	PRJNA1100710
GCA 039846465	PDT002178426.1	Klebsiella_218	PDS000183511.1	2022	NA	PRJNA1113824
GCA 037005925	PDT002098859.1	Klebsiella_218		2022	China	PRJNA957912
GCA 034732955	PDT002042073.1	Klebsiella_218		2020	China	PRJNA1050895
GCA 038375945	PDT002142101.1	Klebsiella_218		2021	China	PRJNA1081623
GCA 025755105	PDT001462482.1	Klebsiella_218		2015	China	PRJNA742521
GCA 025754615	PDT001462459.1	Klebsiella_218		2016	China	PRJNA742521
GCA 034728955	PDT002041846.1	Klebsiella_218		2021	China	PRJNA1050895
GCA 016916715	PDT000970766.1	Klebsiella_218		2018	Russia	PRJNA269675
GCA 046449285	PDT002554330.1	Klebsiella_218		2014	Russia	PRJNA1196851
GCA 046448125	PDT002554272.1	Klebsiella_218		2011	Russia	PRJNA1196851
GCA 026334715	PDT001494308.1	Klebsiella_218		2020	Ghana	PRJEB56918
GCA 025755235	PDT001462489.1	Klebsiella_218		2015	China	PRJNA742521
GCA 029963605	PDT001704089.1	Klebsiella_218		2019	Japan	PRJDB10842
GCA 030390035	PDT001808880.1	Klebsiella_218		nan	USA	PRJNA788509
GCA 031066625	PDT001702973.1	Klebsiella_218		2019	Japan	PRJDB10842
GCA 030874805	PDT001701706.1	Klebsiella_218		2019	Japan	PRJDB10842
GCA 023742455	PDT001336647.1	Klebsiella_5939		2020	China	PRJNA833902
GCA 037005965	PDT002098861.1	Klebsiella_5939		2021	China	PRJNA957912
GCA 021461305	PDT001223095.1	Klebsiella_218	PDS000079882.2	2019	Ethiopia	PRJNA769286
GCA 034740855	PDT002042448.1	Klebsiella_218		2021	China	PRJNA1050895
GCA 034725715	PDT002041744.1	Klebsiella_218		2021	China	PRJNA1050895
GCA 034737075	PDT002042260.1	Klebsiella_218		2021	China	PRJNA1050895
GCA 037005905	PDT002098858.1	Klebsiella_218		2022	China	PRJNA957912
GCA 046623285	PDT002566380.1	Klebsiella_218		2017	Taiwan	PRJNA1202880
GCA 021166075	PDT001202986.1	Klebsiella_218		nan	Taiwan	PRJNA549322
GCA 004312805	PDT000471505.1	Klebsiella_218		2014	Japan	PRJDB7163
GCA 046065025	PDT002536231.1	Klebsiella_218		2021	Czechia	PRJNA1130981
GCA 039611815	PDT002160730.1	Klebsiella_218	PDS000183580.1	2023	NA	PRJNA1100710
GCA 034739235	PDT002042367.1	Klebsiella_218		2021	China	PRJNA1050895
GCA 037005885	PDT002098857.1	Klebsiella_218		2022	China	PRJNA957912
GCA 034736075	PDT002042211.1	Klebsiella_218		2021	China	PRJNA1050895
GCA 034724275	PDT002041695.1	Klebsiella_218		2021	China	PRJNA1050895
GCA 040986225	PDT002279623.1	Klebsiella_218		nan	China	PRJNA1021041
GCA 046464435	PDT002556447.1	Klebsiella_218	PDS000097290.2	nan	NA	PRJNA786399
GCA 022010155	PDT001100901.1	Klebsiella_218	PDS000097290.2	2009	China	PRJNA323522
GCA 046465555	PDT002556502.1	Klebsiella_218	PDS000097290.2	nan	NA	PRJNA786399
GCA 020479465	PDT001151320.1	Klebsiella_218	PDS000097290.2	2014	China	PRJNA612328

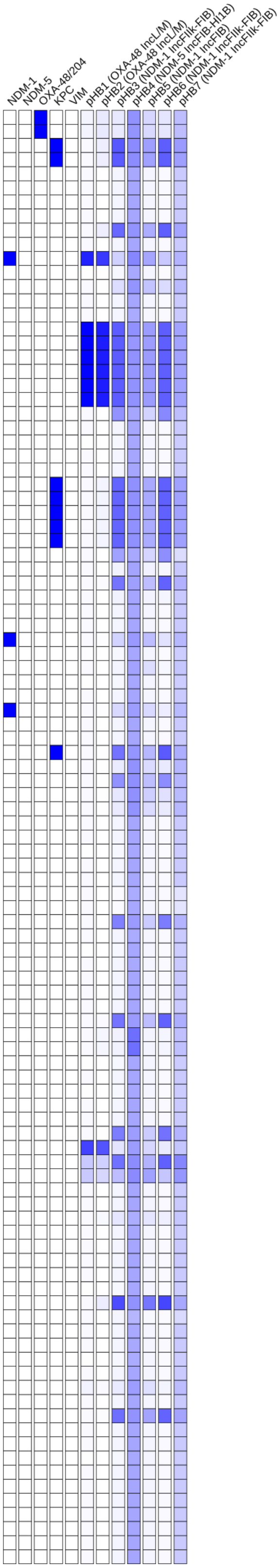

Tree 18: GCA_043863245

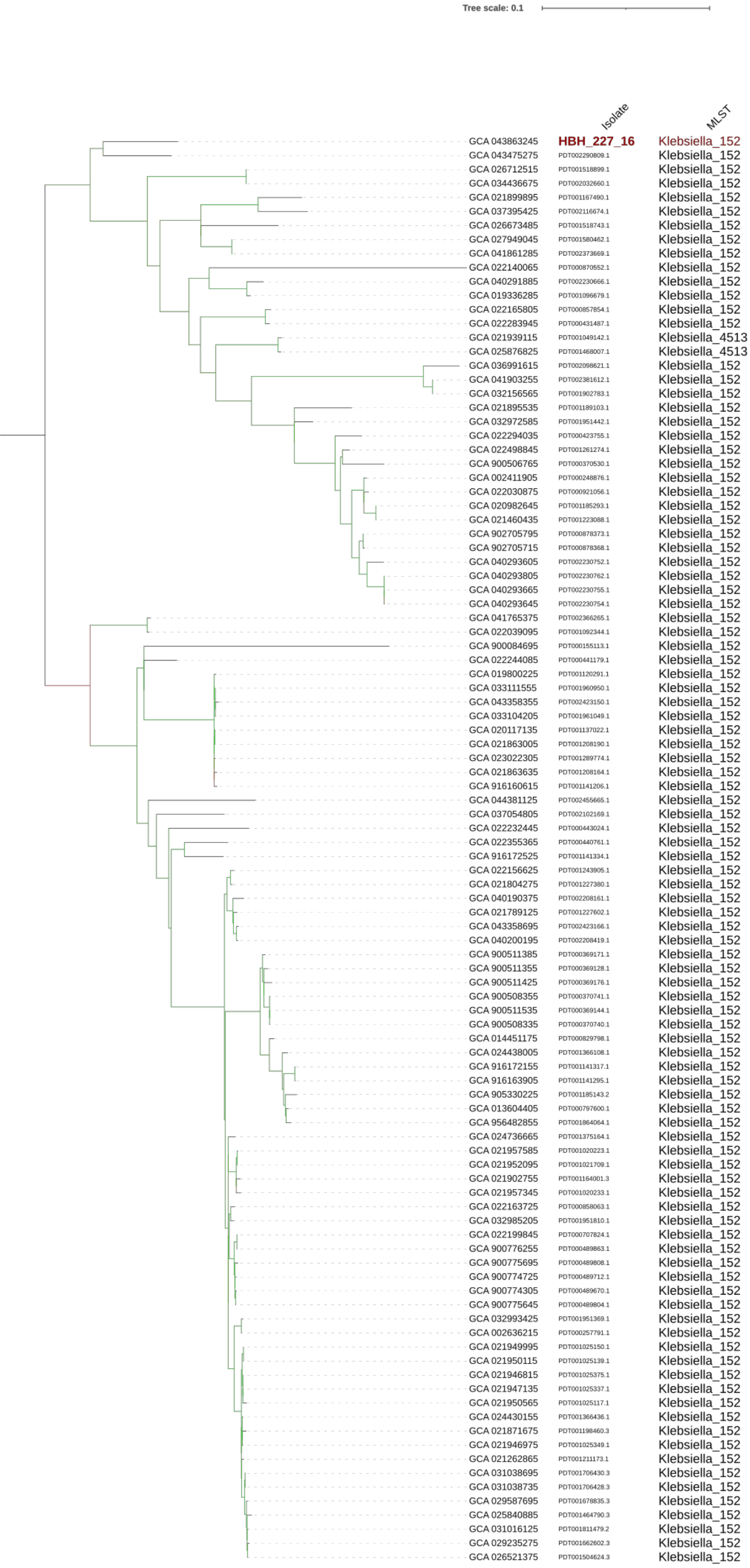

	Isolate	MLST	SNP Cluster	Collection Year	Country	BioProject
GCA 043863245	**HBH_227_16**	Klebsiella_152		2016	Tunisia	PRJNA1160997
GCA 043475275	PDT002290809.1	Klebsiella_152		2022	Bangladesh	PRJNA987754
GCA 026712515	PDT001518899.1	Klebsiella_152	PDS000170201.1	2021	India	PRJNA737586
GCA 034436675	PDT002032660.1	Klebsiella_152	PDS000170201.1	2021	India	PRJEB58136
GCA 021899895	PDT001167490.1	Klebsiella_152		2017	China	PRJNA769451
GCA 037395425	PDT002116674.1	Klebsiella_152		2014	USA	PRJNA288601
GCA 026673485	PDT001518743.1	Klebsiella_152		2022	Ghana	PRJNA906096
GCA 027949045	PDT001580462.1	Klebsiella_152	PDS000197588.1	2020	Norway	PRJNA922900
GCA 041861285	PDT002373669.1	Klebsiella_152	PDS000197588.1	2020	Norway	PRJNA922900
GCA 022140065	PDT000870552.1	Klebsiella_152		2015	USA	PRJNA594602
GCA 040291885	PDT002230666.1	Klebsiella_152		2017	Bangladesh	PRJNA719593
GCA 019336285	PDT001096679.1	Klebsiella_152		2019	India	PRJNA636233
GCA 022165805	PDT000857854.1	Klebsiella_152		2016	USA	PRJNA658369
GCA 022283945	PDT000431487.1	Klebsiella_152		2017	USA	PRJNA396774
GCA 021939115	PDT001049142.1	Klebsiella_4513	PDS000100167.3	2018	Australia	PRJNA857533
GCA 025876825	PDT001468007.1	Klebsiella_4513	PDS000100167.3	2019	Norway	PRJEB56146
GCA 036991615	PDT002098621.1	Klebsiella_152		2016	China	PRJNA1003408
GCA 041903255	PDT002381612.1	Klebsiella_152	PDS000198509.1	2017	Norway	PRJNA1098507
GCA 032156565	PDT001902783.1	Klebsiella_152	PDS000198509.1	2017	Norway	PRJEB48268
GCA 021895535	PDT001189103.1	Klebsiella_152		2016	Pakistan	PRJEB40861
GCA 032972585	PDT001951442.1	Klebsiella_152		2011	Canada	PRJNA855907
GCA 022294035	PDT000423755.1	Klebsiella_152		nan	NA	PRJEB17615
GCA 022498845	PDT001261274.1	Klebsiella_152	PDS000106014.1	2002	Australia	PRJNA810752
GCA 900506765	PDT000370530.1	Klebsiella_152		2013	Belgium	PRJEB10018
GCA 002411905	PDT000248876.1	Klebsiella_152		2015	Ghana	PRJNA411997
GCA 022030875	PDT000921056.1	Klebsiella_152		nan	NA	PRJEB27342
GCA 020982645	PDT001185293.1	Klebsiella_152	PDS000102786.1	2021	Ghana	PRJNA750808
GCA 021460435	PDT001223088.1	Klebsiella_152	PDS000102786.1	2021	Ghana	PRJNA769286
GCA 902705795	PDT000878373.1	Klebsiella_152	PDS000074978.1	2006	United Kingdom	PRJEB34549
GCA 902705715	PDT000878368.1	Klebsiella_152	PDS000074978.1	2007	United Kingdom	PRJEB34549
GCA 040293605	PDT002230752.1	Klebsiella_152		2016	Bangladesh	PRJNA719593
GCA 040293805	PDT002230762.1	Klebsiella_152	PDS000186854.1	2017	Bangladesh	PRJNA719593
GCA 040293665	PDT002230755.1	Klebsiella_152	PDS000186854.1	2017	Bangladesh	PRJNA719593
GCA 040293645	PDT002230754.1	Klebsiella_152	PDS000186854.1	2017	Bangladesh	PRJNA719593
GCA 041765375	PDT002366265.1	Klebsiella_152	PDS000197056.1	2009	Norway	PRJNA1098507
GCA 022039095	PDT001092344.1	Klebsiella_152	PDS000197056.1	2009	Norway	PRJEB27256
GCA 900084695	PDT000155113.1	Klebsiella_152		2001	United Kingdom	PRJEB5065
GCA 022244085	PDT000441179.1	Klebsiella_152		2012	Nepal	PRJEB1800
GCA 019800225	PDT001120291.1	Klebsiella_152	PDS000075204.9	nan	Germany	PRJNA546126
GCA 033111555	PDT001960950.1	Klebsiella_152	PDS000075204.9	2017	USA	PRJNA648389
GCA 043358355	PDT002423150.1	Klebsiella_152	PDS000075204.9	nan	Saudi Arabia	PRJNA1116866
GCA 033104205	PDT001961049.1	Klebsiella_152	PDS000075204.9	2019	USA	PRJNA648389
GCA 020117135	PDT001137022.1	Klebsiella_152	PDS000075204.9	2017	Switzerland	PRJNA543274
GCA 021863005	PDT001208190.1	Klebsiella_152	PDS000075204.9	2018	Switzerland	PRJEB49326
GCA 023022305	PDT001289774.1	Klebsiella_152	PDS000075204.9	2018	Turkey	PRJNA789336
GCA 021863635	PDT001208164.1	Klebsiella_152	PDS000075204.9	2018	Switzerland	PRJEB49326
GCA 916160615	PDT001141206.1	Klebsiella_152	PDS000075204.9	2008	Netherlands	PRJEB41042
GCA 044381125	PDT002455665.1	Klebsiella_152		nan	Saudi Arabia	PRJNA1116866
GCA 037054805	PDT002102169.1	Klebsiella_152		2011	Jordan	PRJNA1076682
GCA 022232445	PDT000443024.1	Klebsiella_152		2012	Vietnam	PRJEB26814
GCA 022355365	PDT000440761.1	Klebsiella_152		nan	NA	PRJEB21605
GCA 916172525	PDT001141334.1	Klebsiella_152		2020	Netherlands	PRJEB44242
GCA 022156625	PDT001243905.1	Klebsiella_152		2015	United Kingdom	PRJNA788733
GCA 021804275	PDT001227380.1	Klebsiella_152		2015	United Kingdom	PRJNA788733
GCA 040190375	PDT002208161.1	Klebsiella_152	PDS000185318.1	nan	Saudi Arabia	PRJNA1116866
GCA 021789125	PDT001227602.1	Klebsiella_152		2014	United Kingdom	PRJNA788733
GCA 043358695	PDT002423166.1	Klebsiella_152	PDS000201460.1	nan	Saudi Arabia	PRJNA1116866
GCA 040200195	PDT002208419.1	Klebsiella_152	PDS000201460.1	nan	Saudi Arabia	PRJNA1116866
GCA 900511385	PDT000369171.1	Klebsiella_152		2014	Poland	PRJEB10018
GCA 900511355	PDT000369128.1	Klebsiella_152		2014	Poland	PRJEB10018
GCA 900511425	PDT000369176.1	Klebsiella_152		2013	Poland	PRJEB10018
GCA 900508355	PDT000370741.1	Klebsiella_152	PDS000036263.5	2014	Poland	PRJEB10018
GCA 900511535	PDT000369144.1	Klebsiella_152	PDS000036263.5	2014	Poland	PRJEB10018
GCA 900508335	PDT000370740.1	Klebsiella_152	PDS000036263.5	2013	Poland	PRJEB10018
GCA 014451175	PDT000829798.1	Klebsiella_152	PDS000071072.5	2017	Brazil	PRJNA521546
GCA 024438005	PDT001366108.1	Klebsiella_152	PDS000213526.2	2018	Guadeloupe	PRJNA778230
GCA 916172155	PDT001141317.1	Klebsiella_152	PDS000096689.1	2019	Netherlands	PRJEB44242
GCA 916163905	PDT001141295.1	Klebsiella_152	PDS000096689.1	2010	Netherlands	PRJEB41042
GCA 905330225	PDT001185143.2	Klebsiella_152	PDS000110342.1	2018	Spain	PRJEB42440
GCA 013604405	PDT000797600.1	Klebsiella_152		nan	Spain	PRJNA609897
GCA 956482855	PDT001864064.1	Klebsiella_152		2016	Spain	PRJEB63349
GCA 024736665	PDT001375164.1	Klebsiella_152		2019	Spain	PRJEB50822
GCA 021957585	PDT001020223.1	Klebsiella_152	PDS000076193.10	2018	Colombia	PRJNA658369
GCA 021952095	PDT001021709.1	Klebsiella_152	PDS000076193.10	2018	Colombia	PRJNA658369
GCA 021902755	PDT001164001.3	Klebsiella_152	PDS000076193.10	2019	USA	PRJNA288601
GCA 021957345	PDT001020233.1	Klebsiella_152	PDS000076193.10	2018	Colombia	PRJNA658369
GCA 022163725	PDT000858063.1	Klebsiella_152		2017	USA	PRJNA658369
GCA 032985205	PDT001951810.1	Klebsiella_152		2017	Canada	PRJNA855907
GCA 022199845	PDT000707824.1	Klebsiella_152	PDS000044881.3	nan	NA	PRJEB22903
GCA 900776255	PDT000489863.1	Klebsiella_152	PDS000044881.3	nan	NA	PRJEB31361
GCA 900775695	PDT000489808.1	Klebsiella_152	PDS000044881.3	nan	NA	PRJEB31361
GCA 900774725	PDT000489712.1	Klebsiella_152	PDS000044881.3	nan	NA	PRJEB31361
GCA 900774305	PDT000489670.1	Klebsiella_152	PDS000044881.3	nan	NA	PRJEB31361
GCA 900775645	PDT000489804.1	Klebsiella_152	PDS000044881.3	nan	NA	PRJEB31361
GCA 032993425	PDT001951369.1	Klebsiella_152	PDS000213526.2	2014	Canada	PRJNA855907
GCA 002636215	PDT000257791.1	Klebsiella_152	PDS000213526.2	2014	Canada	PRJNA231221
GCA 021949995	PDT001025150.1	Klebsiella_152	PDS000213526.2	2016	USA	PRJNA686897
GCA 021950115	PDT001025139.1	Klebsiella_152	PDS000213526.2	2016	USA	PRJNA686897
GCA 021946815	PDT001025375.1	Klebsiella_152	PDS000213526.2	2016	USA	PRJNA686897
GCA 021947135	PDT001025337.1	Klebsiella_152	PDS000213526.2	2016	USA	PRJNA686897
GCA 021950565	PDT001025117.1	Klebsiella_152	PDS000213526.2	2018	USA	PRJNA686897
GCA 024430155	PDT001366436.1	Klebsiella_152	PDS000213526.2	nan	USA	PRJNA825705
GCA 021871675	PDT001198460.3	Klebsiella_152	PDS000213526.2	2021	USA	PRJNA288601
GCA 021946975	PDT001025349.1	Klebsiella_152	PDS000213526.2	2017	USA	PRJNA686897
GCA 021262865	PDT001211173.1	Klebsiella_152	PDS000213526.2	nan	USA	PRJNA788509
GCA 031038695	PDT001706430.3	Klebsiella_152	PDS000088478.25	2023	USA	PRJNA288601
GCA 031038735	PDT001706428.3	Klebsiella_152	PDS000088478.25	2023	USA	PRJNA288601
GCA 029587695	PDT001678835.3	Klebsiella_152	PDS000088478.25	2023	USA	PRJNA288601
GCA 025840885	PDT001464790.3	Klebsiella_152	PDS000088478.25	2022	USA	PRJNA288601
GCA 031016125	PDT001811479.2	Klebsiella_152	PDS000088478.25	2023	USA	PRJNA288601
GCA 029235275	PDT001662602.3	Klebsiella_152	PDS000088478.25	2023	USA	PRJNA288601
GCA 026521375	PDT001504624.3	Klebsiella_152	PDS000088478.25	2022	USA	PRJNA288601

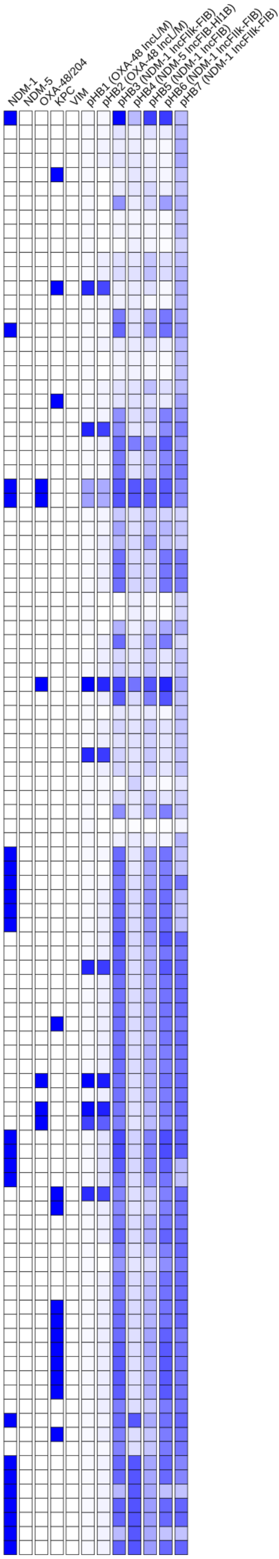

Tree 19: GCA_043863305

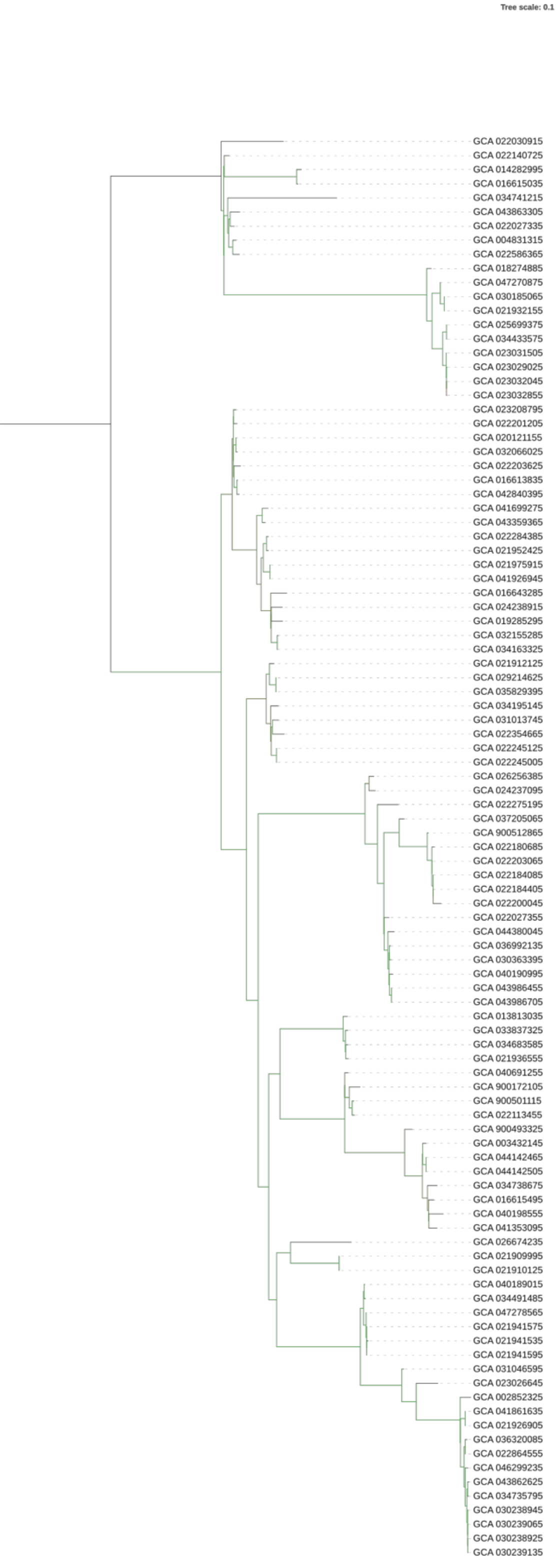

	Isolate	MLST	SNP Cluster	Collection Year	Country	BioProject
GCA 022030915	PDT000921045.1	Klebsiella_661		nan	NA	PRJEB27342
GCA 022140725	PDT000870522.1	Klebsiella_661		2017	USA	PRJNA594602
GCA 014282995	PDT000820963.1	Klebsiella_3409		2016	China	PRJNA657553
GCA 016615035	PDT000932809.1	Klebsiella_3409		nan	NA	PRJNA550041
GCA 034741215	PDT002042465.1	Klebsiella_3453		2021	China	PRJNA1050895
GCA 043863305	**HBH_2991_17**	Klebsiella_661		2017	Tunisia	PRJNA1160997
GCA 022027335	PDT000925080.1	Klebsiella_-		2016	Singapore	PRJNA674942
GCA 004831315	PDT000495941.1	Klebsiella_661		2018	Thailand	PRJNA532291
GCA 022586365	PDT001269982.1	Klebsiella_661		2019	China	PRJNA811738
GCA 018274885	PDT001020811.1	Klebsiella_661		2019	China	PRJNA725121
GCA 047270875	PDT002595997.1	Klebsiella_661	PDS000142414.5	2019	Australia	PRJNA674961
GCA 030185065	PDT001771669.2	Klebsiella_661	PDS000142414.5	2019	Australia	PRJNA924056
GCA 021932155	PDT001087404.1	Klebsiella_661	PDS000142414.5	2019	Australia	PRJNA529744
GCA 025699375	PDT001454971.1	Klebsiella_661	PDS000170208.1	2021	India	PRJNA737586
GCA 034433575	PDT002032681.1	Klebsiella_661	PDS000170208.1	2021	India	PRJEB58136
GCA 023031505	PDT001289337.1	Klebsiella_661	PDS000108371.1	2020	Australia	PRJNA777643
GCA 023029025	PDT001289415.1	Klebsiella_661	PDS000108371.1	2020	Australia	PRJNA777643
GCA 023032045	PDT001289295.1	Klebsiella_661	PDS000108371.1	2020	Australia	PRJNA777643
GCA 023032855	PDT001289233.1	Klebsiella_661	PDS000108371.1	2020	Australia	PRJNA777643
GCA 023208795	PDT001296737.2	Klebsiella_-		2018	USA	PRJNA789565
GCA 022201205	PDT000706644.1	Klebsiella_-		2018	Italy	PRJEB27342
GCA 020121155	PDT001137126.1	Klebsiella_661	PDS000160820.1	2017	Switzerland	PRJNA543274
GCA 032066025	PDT001905153.1	Klebsiella_661	PDS000160820.1	2017	Norway	PRJEB48268
GCA 022203625	PDT000674653.1	Klebsiella_-		2018	Italy	PRJEB27342
GCA 016613835	PDT000932757.1	Klebsiella_661		nan	NA	PRJNA550041
GCA 042840395	PDT002084472.1	Klebsiella_661		2019	Australia	PRJNA674961
GCA 041699275	PDT002351952.1	Klebsiella_661		2023	Ecuador	PRJNA1151051
GCA 043359365	PDT002423200.1	Klebsiella_661		nan	Saudi Arabia	PRJNA1116866
GCA 022284385	PDT000431463.1	Klebsiella_661		2017	USA	PRJNA396774
GCA 021952425	PDT001021726.1	Klebsiella_661		2017	Colombia	PRJNA658369
GCA 021975915	PDT001011509.1	Klebsiella_661	PDS000198827.1	2015	Norway	PRJEB42350
GCA 041926945	PDT002383481.1	Klebsiella_661	PDS000198827.1	2015	Norway	PRJNA1098507
GCA 016643285	PDT000933584.1	Klebsiella_661		nan	Portugal	PRJNA602991
GCA 024238915	PDT001359356.1	Klebsiella_661		2017	China	PRJNA833746
GCA 019285295	PDT001093762.1	Klebsiella_661		nan	Thailand	PRJNA668870
GCA 032155285	PDT001902850.1	Klebsiella_661	PDS000169090.1	2017	Norway	PRJEB48268
GCA 034163325	PDT002016340.1	Klebsiella_-	PDS000169090.1	2022	USA	PRJNA475751
GCA 021912125	PDT001094737.1	Klebsiella_661		2018	Thailand	PRJEB38540
GCA 029214625	PDT001661802.1	Klebsiella_-	PDS000172294.1	2019	United Kingdom	PRJNA943144
GCA 035829395	PDT002058695.1	Klebsiella_661	PDS000172294.1	2019	United Kingdom	PRJNA943144
GCA 034195145	PDT002017829.1	Klebsiella_661		2023	USA	PRJNA739636
GCA 031013745	PDT001830708.2	Klebsiella_661		2023	USA	PRJNA288601
GCA 022354665	PDT001253437.1	Klebsiella_661		2018	China	PRJNA603241
GCA 022245125	PDT000441113.1	Klebsiella_-	PDS000041757.1	2012	Nepal	PRJEB1800
GCA 022245005	PDT000441121.1	Klebsiella_661	PDS000041757.1	2012	Nepal	PRJEB1800
GCA 026256385	PDT001849241.1	Klebsiella_661		2013	Australia	PRJNA646837
GCA 024237095	PDT001359271.1	Klebsiella_661		2017	China	PRJNA833746
GCA 022275195	PDT000433097.1	Klebsiella_-		2010	Australia	PRJNA351911
GCA 037205065	PDT002115704.1	Klebsiella_661		2022	Saudi Arabia	PRJNA1018815
GCA 900512865	PDT000369596.1	Klebsiella_3655		2013	Italy	PRJEB10018
GCA 022180685	PDT000736015.1	Klebsiella_-	PDS000052192.6	2018	Italy	PRJEB27342
GCA 022203065	PDT000674690.1	Klebsiella_-	PDS000052192.6	2018	Italy	PRJEB27342
GCA 022184085	PDT000723763.1	Klebsiella_-	PDS000052192.6	2018	Italy	PRJEB27342
GCA 022184405	PDT000723762.1	Klebsiella_-	PDS000052192.6	2018	Italy	PRJEB27342
GCA 022200045	PDT000706887.1	Klebsiella_-	PDS000052192.6	2018	Italy	PRJEB27342
GCA 022027355	PDT000925074.1	Klebsiella_-		2016	Singapore	PRJNA674942
GCA 044380045	PDT002305404.1	Klebsiella_661		nan	USA	PRJNA271899
GCA 036992135	PDT002098647.1	Klebsiella_661		2015	China	PRJNA1003408
GCA 030363395	PDT001792617.1	Klebsiella_661		2020	China	PRJNA925808
GCA 040190995	PDT002208192.1	Klebsiella_661		nan	Saudi Arabia	PRJNA1116866
GCA 043986455	PDT002376743.1	Klebsiella_661	PDS000198414.1	2021	Japan	PRJDB14656
GCA 043986705	PDT002376731.1	Klebsiella_661	PDS000198414.1	2021	Japan	PRJDB14656
GCA 013813035	PDT000799019.1	Klebsiella_661		2017	United Kingdom	PRJNA605147
GCA 033837325	PDT001992462.1	Klebsiella_-		2016	Canada	PRJNA532924
GCA 034683585	PDT001704442.1	Klebsiella_661		2020	Japan	PRJDB10842
GCA 021936555	PDT001079457.1	Klebsiella_661		2021	USA	PRJNA739636
GCA 040691255	PDT002251066.1	Klebsiella_661		2020	China	PRJNA1131666
GCA 900172105	PDT000198763.1	Klebsiella_661		2014	United Kingdom	PRJEB8771
GCA 900501115	PDT000369824.1	Klebsiella_661		2014	Spain	PRJEB10018
GCA 022113455	PDT001245300.1	Klebsiella_-		2019	Netherlands	PRJNA754858
GCA 900493325	PDT000354664.1	Klebsiella_661		2016	Thailand	PRJEB19226
GCA 003432145	PDT000370995.1	Klebsiella_661		2014	Australia	PRJNA486877
GCA 044142465	PDT002448528.1	Klebsiella_661	PDS000203214.1	2024	India	PRJNA1109570
GCA 044142505	PDT002448530.1	Klebsiella_661	PDS000203214.1	2024	India	PRJNA1109570
GCA 034738675	PDT002042340.1	Klebsiella_661		2021	China	PRJNA1050895
GCA 016615495	PDT000932823.1	Klebsiella_661		nan	NA	PRJNA550041
GCA 040198555	PDT002208338.1	Klebsiella_661		nan	Saudi Arabia	PRJNA1116866
GCA 041353095	PDT002331169.1	Klebsiella_661		nan	Spain	PRJNA985214
GCA 026674235	PDT001518780.1	Klebsiella_661		2022	Ghana	PRJNA906096
GCA 021909995	PDT001094837.1	Klebsiella_-	PDS000093436.1	2018	Thailand	PRJEB38540
GCA 021910125	PDT001094831.1	Klebsiella_-	PDS000093436.1	2018	Thailand	PRJEB38540
GCA 044189015	PDT002208094.1	Klebsiella_661	PDS000185324.1	nan	Saudi Arabia	PRJNA1116866
GCA 034491485	PDT002038563.1	Klebsiella_661	PDS000185324.1	2020	France	PRJNA876630
GCA 047278565	PDT002595974.1	Klebsiella_-	PDS000090686.2	2017	Australia	PRJNA674961
GCA 021941575	PDT001049037.1	Klebsiella_661	PDS000090686.2	2017	Australia	PRJNA857533
GCA 021941535	PDT001049036.1	Klebsiella_661	PDS000090686.2	2018	Australia	PRJNA857533
GCA 021941595	PDT001049038.1	Klebsiella_661	PDS000090686.2	2017	Australia	PRJNA857533
GCA 031046595	PDT001726497.3	Klebsiella_661		2018	USA	PRJNA288601
GCA 023026645	PDT001289556.1	Klebsiella_661		2020	Australia	PRJNA777643
GCA 002852325	PDT000276101.2	Klebsiella_661		2017	China	PRJNA353728
GCA 041861635	PDT002373704.1	Klebsiella_661	PDS000197609.1	2015	Norway	PRJNA1098507
GCA 021926905	PDT001091800.1	Klebsiella_661	PDS000197609.1	2015	Norway	PRJEB27256
GCA 036320085	PDT002069421.1	Klebsiella_661	PDS000053783.5	2016	China	PRJNA1026749
GCA 022864555	PDT001283093.1	Klebsiella_661	PDS000053783.5	2017	China	PRJNA778807
GCA 046299235	PDT002552212.1	Klebsiella_661	PDS000053783.5	2023	China	PRJNA1200658
GCA 043862625	PDT002431766.1	Klebsiella_661		2017	China	PRJNA1175053
GCA 034735795	PDT002042197.1	Klebsiella_661		2021	China	PRJNA1050895
GCA 030238945	PDT001773009.1	Klebsiella_661	PDS000148773.2	2019	Australia	PRJNA949397
GCA 030239065	PDT001773015.1	Klebsiella_661	PDS000148773.2	2019	Australia	PRJNA949397
GCA 030238925	PDT001773008.1	Klebsiella_661	PDS000148773.2	2019	Australia	PRJNA949397
GCA 030239135	PDT001773019.1	Klebsiella_661	PDS000148773.2	2019	Australia	PRJNA949397

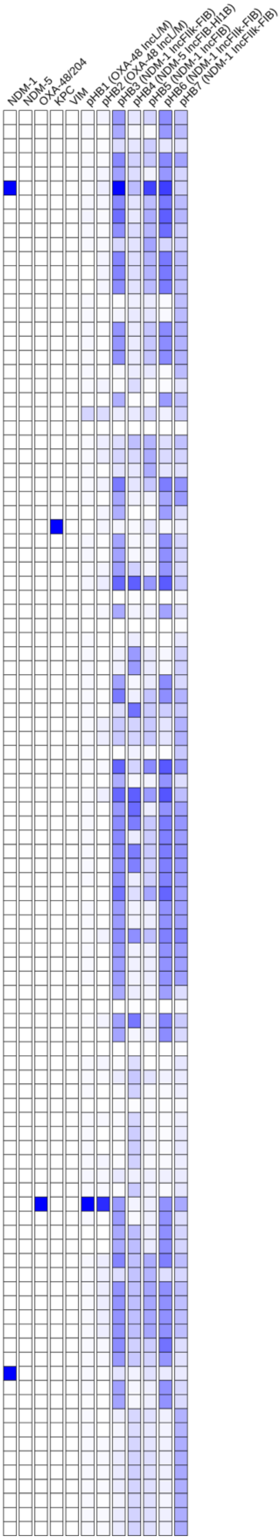

Tree 20: GCA_043863325

Tree scale: 0.1

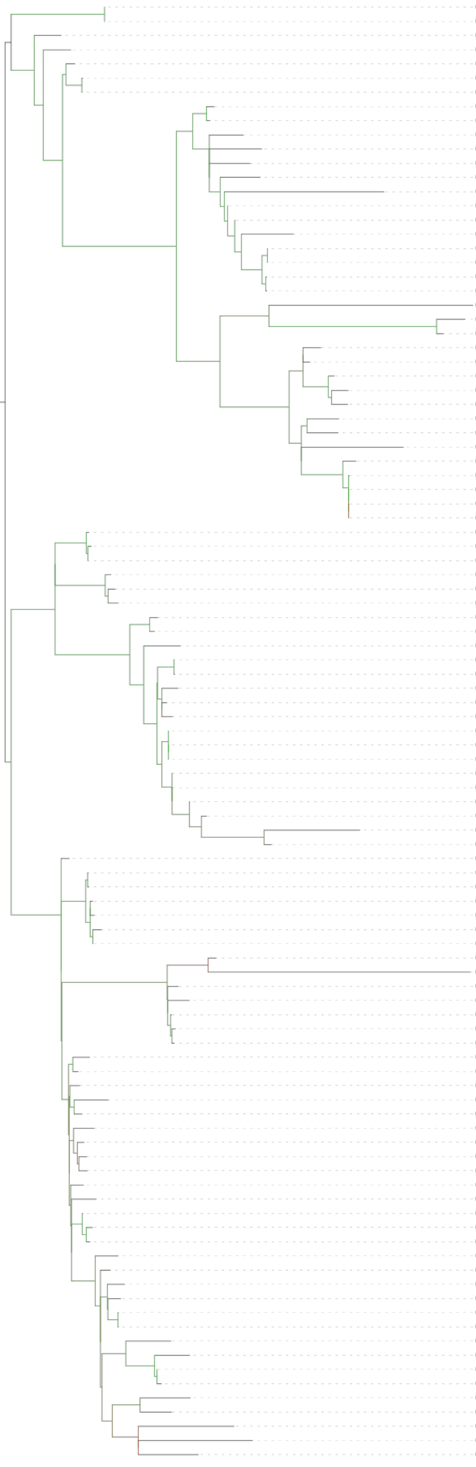

Genome	Isolate	MLST	SNP Cluster	Collection Year	Country	BioProject
GCA 022169805	PDT000847255.1	Klebsiella_15	PDS000072336.1	2018	Myanmar	PRJDB5126
GCA 022169725	PDT000847259.1	Klebsiella_15	PDS000072336.1	2018	Myanmar	PRJDB5126
GCA 041038315	PDT002292032.1	Klebsiella_15		nan	China	PRJNA1021041
GCA 022211465	PDT000631362.1	Klebsiella_15	PDS000062513.1	2014	Canada	PRJNA564992
GCA 037387465	PDT002117100.1	Klebsiella_15		2014	USA	PRJNA288601
GCA 024603435	PDT001379022.1	Klebsiella_15	PDS000115079.3	2018	USA	PRJNA836696
GCA 033104225	PDT001961052.1	Klebsiella_15	PDS000115079.3	2019	USA	PRJNA648389
GCA 043863325	**HBH_2648_17**	Klebsiella_15	PDS000199428.1	2017	Tunisia	PRJNA1160997
GCA 043863125	**HBH_1334_16**	Klebsiella_15	PDS000199428.1	2016	Tunisia	PRJNA1160997
GCA 900513835	PDT000369555.1	Klebsiella_15	PDS000036279.2	2014	Montenegro	PRJEB10018
GCA 012971665	PDT000730634.1	Klebsiella_15		2017	South Korea	PRJNA625837
GCA 036236705	PDT002066778.1	Klebsiella_15		2021	Russia	PRJNA729628
GCA 026197775	PDT001488427.1	Klebsiella_15		2021	China	PRJNA838135
GCA 024582535	PDT001381311.1	Klebsiella_15		2020	China	PRJNA774095
GCA 046465275	PDT002556489.1	Klebsiella_15	PDS000214445.1	nan	NA	PRJNA786399
GCA 046466215	PDT002556535.1	Klebsiella_15	PDS000214445.1	nan	NA	PRJNA786399
GCA 034735455	PDT002042180.1	Klebsiella_15		2021	China	PRJNA1050895
GCA 039611895	PDT002160734.1	Klebsiella_15	PDS000170844.3	2023	NA	PRJNA1100710
GCA 039844865	PDT002178360.1	Klebsiella_15	PDS000170844.3	2022	NA	PRJNA1113824
GCA 034740115	PDT002042411.1	Klebsiella_15	PDS000170844.3	2021	China	PRJNA1050895
GCA 034739755	PDT002042393.1	Klebsiella_15	PDS000170844.3	2021	China	PRJNA1050895
GCA 030360345	PDT001792583.1	Klebsiella_15		2016	Mexico	PRJNA985271
GCA 046531795	PDT002559559.1	Klebsiella_15		2023	China	PRJNA1095163
GCA 025109045	PDT001411010.1	Klebsiella_15	PDS000045329.17	2014	Singapore	PRJNA577535
GCA 900512275	PDT000369264.1	Klebsiella_15		2014	Germany	PRJEB10018
GCA 900505905	PDT000370325.1	Klebsiella_15	PDS000045329.17	2014	Croatia	PRJEB10018
GCA 022313055	PDT000136320.2	Klebsiella_15	PDS000045329.17	2011	United Kingdom	PRJNA306133
GCA 022229825	PDT000446046.1	Klebsiella_15		2015	Germany	PRJEB18059
GCA 022984665	PDT001286497.1	Klebsiella_15	PDS000045329.17	2011	Germany	PRJNA789142
GCA 041039825	PDT002292107.1	Klebsiella_15		nan	China	PRJNA1021041
GCA 022009575	PDT000974742.1	Klebsiella_15		2018	Australia	PRJNA857533
GCA 900493705	PDT000354582.1	Klebsiella_15	PDS000045331.1	2016	Thailand	PRJEB19226
GCA 043859485	**HBH_545_12**	Klebsiella_15	PDS000041726.21	2012	Tunisia	PRJNA1160997
GCA 022231935	PDT000443857.1	Klebsiella_15	PDS000041726.21	nan	NA	PRJEB8666
GCA 022028755	PDT000921131.1	Klebsiella_15	PDS000041726.21	nan	NA	PRJEB27342
GCA 022231425	PDT000443996.1	Klebsiella_15	PDS000041726.21	nan	NA	PRJEB8666
GCA 022231985	PDT000443854.1	Klebsiella_15	PDS000041726.21	nan	NA	PRJEB8666
GCA 916172225	PDT001141321.1	Klebsiella_15	PDS000045329.17	2012	Netherlands	PRJEB44242
GCA 916163895	PDT001141294.1	Klebsiella_15	PDS000045329.17	2013	Netherlands	PRJEB41042
GCA 916163945	PDT001141299.1	Klebsiella_15	PDS000045329.17	2013	Netherlands	PRJEB41042
GCA 013620925	PDT000797615.1	Klebsiella_15	PDS000066127.3	2012	Portugal	PRJNA408270
GCA 013521185	PDT000797569.1	Klebsiella_15	PDS000066127.3	2012	Portugal	PRJNA408270
GCA 013620905	PDT000797616.1	Klebsiella_15	PDS000066127.3	2013	Portugal	PRJNA408270
GCA 040493875	PDT002239669.1	Klebsiella_15	PDS000124952.2	2021	Brazil	PRJNA975155
GCA 012241595	PDT000721275.1	Klebsiella_15	PDS000124952.2	2019	Brazil	PRJNA622302
GCA 905231965	PDT001185040.2	Klebsiella_15	PDS000045329.17	2018	Spain	PRJEB42440
GCA 047658805	PDT002613000.1	Klebsiella_15	PDS000045329.17	2018	Chile	PRJNA962904
GCA 047569515	PDT002613001.1	Klebsiella_-	PDS000045329.17	2018	Chile	PRJNA962904
GCA 022171845	PDT000785613.1	Klebsiella_15		nan	Peru	PRJNA592157
GCA 022181865	PDT000730424.1	Klebsiella_-		2017	Canada	PRJNA564992
GCA 047280045	PDT002595881.1	Klebsiella_-		2018	Australia	PRJNA674961
GCA 015643945	PDT000896252.1	Klebsiella_15	PDS000045329.17	2015	Spain	PRJNA641166
GCA 022987385	PDT001286532.1	Klebsiella_15	PDS000045329.17	2014	Spain	PRJNA803387
GCA 027866245	PDT001562003.1	Klebsiella_15	PDS000045329.17	2018	Spain	PRJNA803387
GCA 015643785	PDT000896244.1	Klebsiella_15	PDS000045329.17	2015	Spain	PRJNA641166
GCA 027866345	PDT001561998.1	Klebsiella_15	PDS000045329.17	2018	Spain	PRJNA803387
GCA 022987425	PDT001286523.1	Klebsiella_15	PDS000045329.17	2014	Spain	PRJNA803387
GCA 017595965	PDT000992695.1	Klebsiella_15	PDS000045329.17	2017	France	PRJNA675776
GCA 047569615	PDT002612972.1	Klebsiella_15	PDS000045329.17	2018	Chile	PRJNA962904
GCA 032635905	PDT001923292.1	Klebsiella_15	PDS000045329.17	2017	Peru	PRJNA990326
GCA 022212805	PDT000628216.1	Klebsiella_15	PDS000045329.17	2007	Canada	PRJNA564992
GCA 017597315	PDT000992763.1	Klebsiella_15	PDS000083760.2	2013	France	PRJNA675776
GCA 017596835	PDT000992739.1	Klebsiella_15	PDS000083760.2	2015	France	PRJNA675776
GCA 017596705	PDT000992732.1	Klebsiella_15	PDS000083760.2	2015	France	PRJNA675776
GCA 017597125	PDT000992753.1	Klebsiella_15	PDS000083760.2	2014	France	PRJNA675776
GCA 025051015	PDT001384770.1	Klebsiella_15	PDS000083760.2	2014	France	PRJEB50837
GCA 017597025	PDT000992748.1	Klebsiella_15	PDS000083760.2	2015	France	PRJNA675776
GCA 022297035	PDT000423575.1	Klebsiella_15		nan	NA	PRJEB17615
GCA 014835065	PDT000852579.1	Klebsiella_15	PDS000179049.7	2017	USA	PRJNA633565
GCA 900513305	PDT000369344.1	Klebsiella_15		2013	Italy	PRJEB10018
GCA 900512715	PDT000369312.2	Klebsiella_15		2014	Germany	PRJEB10018
GCA 900506155	PDT000370361.1	Klebsiella_15	PDS000045329.17	2014	Croatia	PRJEB10018
GCA 022288065	PDT000427635.1	Klebsiella_15	PDS000045329.17	nan	NA	PRJEB10018
GCA 022288045	PDT000427641.1	Klebsiella_15	PDS000045329.17	nan	NA	PRJEB10018
GCA 022115555	PDT001244484.1	Klebsiella_15		2014	United Kingdom	PRJNA788733
GCA 037051865	PDT002102068.1	Klebsiella_15	PDS000045329.17	2015	Jordan	PRJNA1076682
GCA 022320665	PDT000537649.1	Klebsiella_15	PDS000045329.17	2012	Norway	PRJEB27256
GCA 022189205	PDT000715443.1	Klebsiella_15		2013	United Kingdom	PRJNA564424
GCA 021980855	PDT001009146.1	Klebsiella_15	PDS000045329.17	2018	Italy	PRJEB27342
GCA 006130925	PDT000518697.1	Klebsiella_15		2015	China	PRJNA510171
GCA 037395695	PDT002116651.1	Klebsiella_15	PDS000179049.7	2015	USA	PRJNA288601
GCA 043301705	PDT002265375.1	Klebsiella_15	PDS000179049.7	2018	USA	PRJNA1106484
GCA 040559185	PDT002244896.1	Klebsiella_15	PDS000179049.7	2016	USA	PRJNA1088550
GCA 022244675	PDT000441141.1	Klebsiella_15	PDS000046916.2	2012	Nepal	PRJEB1800
GCA 040290845	PDT002230601.1	Klebsiella_15		2018	Bangladesh	PRJNA719593
GCA 000707985	PDT000033733.1	Klebsiella_15	PDS000045329.17	2012	NA	PRJNA202045
GCA 014903955	PDT000865022.1	Klebsiella_15	PDS000045329.17	nan	USA	PRJNA271899
GCA 014903895	PDT000865021.1	Klebsiella_15	PDS000045329.17	nan	USA	PRJNA271899
GCA 012970325	PDT000730590.1	Klebsiella_15		2016	South Korea	PRJNA625837
GCA 002866645	PDT000277132.1	Klebsiella_15	PDS000045329.17	2013	Lebanon	PRJNA388272
GCA 022305415	PDT000176177.2	Klebsiella_15	PDS000104501.4	nan	NA	PRJEB5065
GCA 022239445	PDT000441644.1	Klebsiella_15	PDS000106761.1	2014	Germany	PRJEB18621
GCA 022248595	PDT000440781.1	Klebsiella_15	PDS000075194.4	nan	NA	PRJEB21605
GCA 025881125	PDT001467779.1	Klebsiella_15	PDS000075194.4	2015	Norway	PRJEB56146
GCA 041401685	PDT002333248.1	Klebsiella_15	PDS000053782.4	2021	Vietnam	PRJNA1043438
GCA 040306085	PDT002230833.1	Klebsiella_15	PDS000186880.1	2017	Bangladesh	PRJNA719593
GCA 040306005	PDT002230829.1	Klebsiella_15	PDS000186880.1	2017	Bangladesh	PRJNA719593
GCA 040306905	PDT002230864.1	Klebsiella_15	PDS000186880.1	2017	Bangladesh	PRJNA719593
GCA 043430835	PDT002421569.1	Klebsiella_15	PDS000045329.17	2019	USA	PRJNA288601
GCA 034295475	PDT002023820.1	Klebsiella_15		2023	USA	PRJNA288601
GCA 047277665	PDT002596029.1	Klebsiella_15		2019	Australia	PRJNA674961
GCA 022182505	PDT000729794.1	Klebsiella_15		2016	Canada	PRJNA564992
GCA 022247465	PDT000440938.1	Klebsiella_15		nan	NA	PRJEB12699

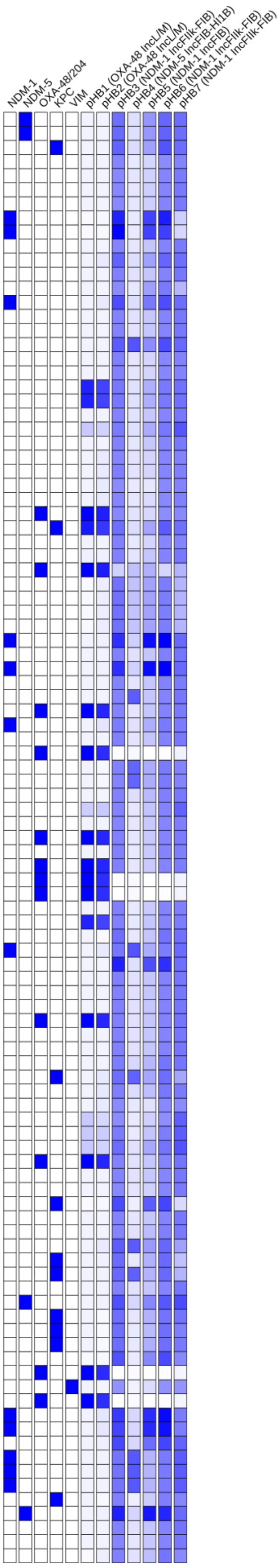

Tree 21: GCA_043863345

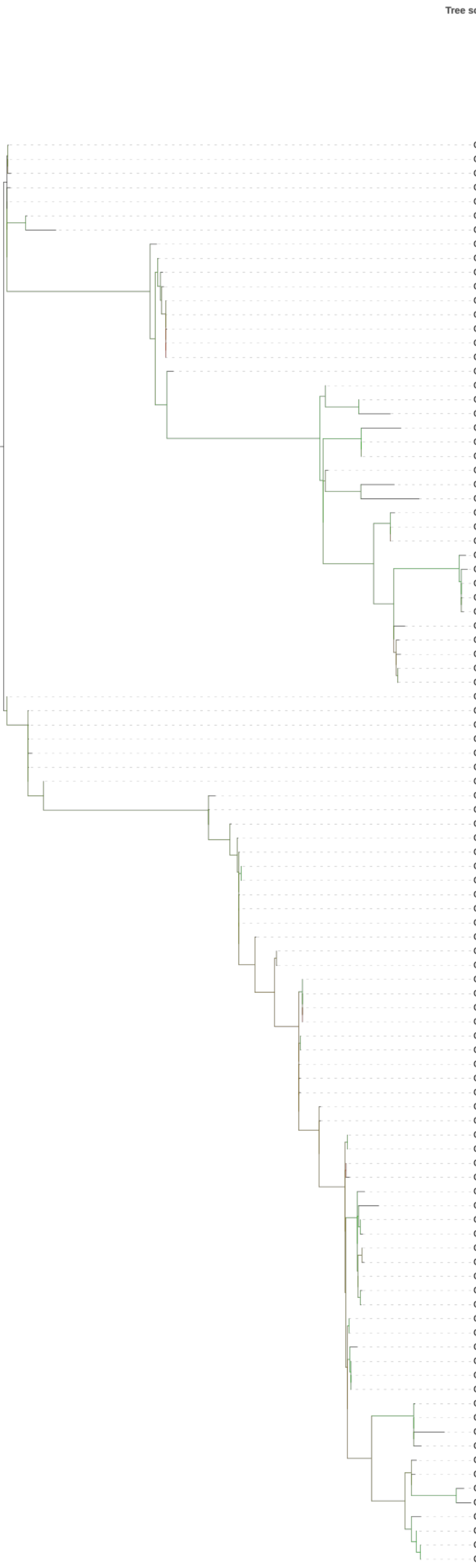

	Isolate	MLST	SNP Cluster	Collection Year	Country	BioProject
GCA 013134415	PDT000744649.1	Klebsiella_11	PDS000036336.53	2014	Poland	PRJNA610720
GCA 013134625	PDT000744658.1	Klebsiella_11	PDS000036336.53	2014	Poland	PRJNA610720
GCA 013134095	PDT000744632.1	Klebsiella_11	PDS000036336.53	2014	Poland	PRJNA610720
GCA 013135335	PDT000744694.1	Klebsiella_11	PDS000036336.53	2013	Poland	PRJNA610720
GCA 013134975	PDT000744676.1	Klebsiella_11	PDS000036336.53	2014	Poland	PRJNA610720
GCA 013135085	PDT000744682.1	Klebsiella_11	PDS000036336.53	2016	Poland	PRJNA610720
GCA 964215045	PDT002343415.1	Klebsiella_11	PDS000113930.22	2023	Slovakia	PRJEB72521
GCA 013134425	PDT000744648.1	Klebsiella_11	PDS000036336.53	2017	Poland	PRJNA610720
GCA 013135015	PDT000744678.1	Klebsiella_11	PDS000036336.53	2016	Poland	PRJNA610720
GCA 030912725	PDT001866003.1	Klebsiella_11	PDS000036336.53	2016	Poland	PRJNA1005055
GCA 013134715	PDT000744663.1	Klebsiella_11	PDS000036336.53	2017	Poland	PRJNA610720
GCA 013134315	PDT000744643.1	Klebsiella_11	PDS000036336.53	2017	Poland	PRJNA610720
GCA 013134195	PDT000744637.1	Klebsiella_11	PDS000036336.53	2018	Poland	PRJNA610720
GCA 013134635	PDT000744660.1	Klebsiella_11		2016	Poland	PRJNA610720
GCA 013134535	PDT000744655.1	Klebsiella_11	PDS000036336.53	2017	Poland	PRJNA610720
GCA 013134515	PDT000744653.1	Klebsiella_11	PDS000036336.53	2017	Poland	PRJNA610720
GCA 018107825	PDT001010112.1	Klebsiella_11	PDS000072335.6	2015	Malaysia	PRJNA541194
GCA 002202255	PDT000219971.1	Klebsiella_11	PDS000077811.10	nan	NA	PRJNA292902
GCA 021931595	PDT001087435.1	Klebsiella_11	PDS000077811.10	2019	Australia	PRJNA529744
GCA 003115035	PDT000313818.2	Klebsiella_11	PDS000077811.10	2016	Vietnam	PRJDB6655
GCA 003400795	PDT000364535.1	Klebsiella_11		2015	USA	PRJNA436373
GCA 011044805	PDT000701153.1	Klebsiella_11	PDS000077811.10	2019	Russia	PRJNA606163
GCA 011044715	PDT000701149.1	Klebsiella_11	PDS000077811.10	2019	Russia	PRJNA606163
GCA 021932555	PDT001087385.1	Klebsiella_11	PDS000077811.10	2019	Australia	PRJNA529744
GCA 016137005	PDT000920683.1	Klebsiella_11	PDS000077811.10	2015	Russia	PRJNA525009
GCA 016136765	PDT000920670.1	Klebsiella_11	PDS000077811.10	2015	Russia	PRJNA525009
GCA 021933295	PDT001087345.1	Klebsiella_11	PDS000092775.2	2017	Australia	PRJNA529744
GCA 047270615	PDT002596048.1	Klebsiella_11	PDS000092775.2	2017	Australia	PRJNA674961
GCA 021934475	PDT001087281.1	Klebsiella_11	PDS000092775.2	2017	Australia	PRJNA529744
GCA 022251515	PDT000440603.1	Klebsiella_11	PDS000045420.7	2012	Spain	PRJEB1272
GCA 021901255	PDT001166807.1	Klebsiella_11	PDS000045421.52	2013	Spain	PRJNA757198
GCA 021900415	PDT001166845.1	Klebsiella_11	PDS000045421.52	2014	Spain	PRJNA757198
GCA 905330575	PDT001185164.2	Klebsiella_11	PDS000045421.52	2018	Spain	PRJEB42440
GCA 905218905	PDT001184997.2	Klebsiella_11	PDS000045421.52	2018	Spain	PRJEB42440
GCA 022274375	PDT000433150.1	Klebsiella_11	PDS000077811.10	2016	Russia	PRJNA429779
GCA 042910375	PDT002091441.1	Klebsiella_11	PDS000077811.10	2014	Vietnam	PRJDB6407
GCA 003115015	PDT000313817.2	Klebsiella_11	PDS000077811.10	2015	Vietnam	PRJDB6655
GCA 021905235	PDT001141114.1	Klebsiella_11	PDS000077811.10	2017	Vietnam	PRJNA641536
GCA 021907035	PDT001110127.1	Klebsiella_11	PDS000077811.10	2017	Vietnam	PRJNA641536
GCA 900510265	PDT000369137.1	Klebsiella_3666	PDS000036336.53	2013	Poland	PRJEB10018
GCA 013134105	PDT000744634.1	Klebsiella_11	PDS000036336.53	2016	Poland	PRJNA610720
GCA 013134935	PDT000744674.1	Klebsiella_11	PDS000036336.53	2016	Poland	PRJNA610720
GCA 013135065	PDT000744681.1	Klebsiella_11	PDS000036336.53	2016	Poland	PRJNA610720
GCA 013133885	PDT000744624.1	Klebsiella_11		2016	Poland	PRJNA610720
GCA 013135225	PDT000744689.1	Klebsiella_11	PDS000036336.53	2015	Poland	PRJNA610720
GCA 013134965	PDT000744677.1	Klebsiella_11	PDS000036336.53	2016	Poland	PRJNA610720
GCA 956476295	PDT001861531.1	Klebsiella_11	PDS000036336.53	2018	Greece	PRJEB63349
GCA 022116275	PDT001244504.1	Klebsiella_11		2015	United Kingdom	PRJNA788733
GCA 900500715	PDT000369925.1	Klebsiella_11	PDS000036336.53	2014	Greece	PRJEB10018
GCA 900501515	PDT000369995.1	Klebsiella_11	PDS000036336.53	2014	Greece	PRJEB10018
GCA 900500745	PDT000369911.1	Klebsiella_11	PDS000036336.53	2013	Greece	PRJEB10018
GCA 947662495	PDT001594203.1	Klebsiella_11	PDS000036336.53	2015	Greece	PRJEB58216
GCA 028090835	PDT001585546.1	Klebsiella_11	PDS000036336.53	2015	Greece	PRJEB58216
GCA 900502385	PDT000369994.1	Klebsiella_11	PDS000036336.53	2014	Greece	PRJEB10018
GCA 900501265	PDT000369891.1	Klebsiella_11	PDS000036336.53	2013	Greece	PRJEB10018
GCA 900500895	PDT000369956.1	Klebsiella_11	PDS000036336.53	2014	Greece	PRJEB10018
GCA 956476705	PDT001861619.1	Klebsiella_11	PDS000036336.53	2018	Greece	PRJEB63349
GCA 028090975	PDT001585537.1	Klebsiella_11	PDS000036336.53	2014	Greece	PRJEB58216
GCA 947660525	PDT001594156.1	Klebsiella_11	PDS000036336.53	2014	Greece	PRJEB58216
GCA 947662905	PDT001592194.1	Klebsiella_11	PDS000036336.53	2016	Greece	PRJEB58216
GCA 947660195	PDT001592186.1	Klebsiella_11	PDS000036336.53	2016	Greece	PRJEB58216
GCA 028090395	PDT001585577.1	Klebsiella_11	PDS000036336.53	2016	Greece	PRJEB58216
GCA 028090015	PDT001585568.1	Klebsiella_11	PDS000036336.53	2016	Greece	PRJEB58216
GCA 947660675	PDT001592150.1	Klebsiella_11	PDS000036336.53	2015	Greece	PRJEB58216
GCA 028090695	PDT001585552.1	Klebsiella_11	PDS000036336.53	2015	Greece	PRJEB58216
GCA 900501545	PDT000369901.1	Klebsiella_11	PDS000036336.53	2013	Greece	PRJEB10018
GCA 900502325	PDT000369993.1	Klebsiella_11	PDS000036336.53	2014	Greece	PRJEB10018
GCA 022378115	PDT001243323.1	Klebsiella_11	PDS000036336.53	2015	United Kingdom	PRJNA788733
GCA 900500735	PDT000369922.1	Klebsiella_11	PDS000036336.53	2014	Greece	PRJEB10018
GCA 956480625	PDT001865593.1	Klebsiella_11	PDS000036336.53	2018	Serbia	PRJEB63349
GCA 947660585	PDT001594174.1	Klebsiella_11	PDS000036336.53	2013	Greece	PRJEB58216
GCA 028091255	PDT001585523.1	Klebsiella_11	PDS000036336.53	2013	Greece	PRJEB58216
GCA 902155945	PDT000663177.1	Klebsiella_3666	PDS000036336.53	2013	Poland	PRJEB33308
GCA 013134365	PDT000744646.1	Klebsiella_11	PDS000036336.53	2014	Poland	PRJNA610720
GCA 900516945	PDT000369689.1	Klebsiella_11	PDS000036336.53	2014	Bulgaria	PRJEB10018
GCA 028089515	PDT001585613.1	Klebsiella_11	PDS000036336.53	2018	Greece	PRJEB58216
GCA 028090035	PDT001585602.1	Klebsiella_11	PDS000036336.53	2017	Greece	PRJEB58216
GCA 956476755	PDT001861545.1	Klebsiella_11	PDS000036336.53	2017	Greece	PRJEB63349
GCA 947662925	PDT001592218.1	Klebsiella_11	PDS000156641.12	2017	Greece	PRJEB58216
GCA 956472565	PDT001861494.1	Klebsiella_11	PDS000156641.12	2017	Greece	PRJEB63349
GCA 028089615	PDT001585606.1	Klebsiella_11	PDS000156641.12	2017	Greece	PRJEB58216
GCA 956476685	PDT001861610.1	Klebsiella_11	PDS000156641.12	2018	Greece	PRJEB63349
GCA 956479255	PDT001861577.1	Klebsiella_11	PDS000156641.12	2018	Greece	PRJEB63349
GCA 900504905	PDT000370216.1	Klebsiella_11	PDS000065468.2	2014	United Kingdom	PRJEB10018
GCA 022159045	PDT001243673.1	Klebsiella_11	PDS000065468.2	2014	United Kingdom	PRJNA788733
GCA 900513195	PDT000369334.1	Klebsiella_11		2013	Italy	PRJEB10018
GCA 002180175	PDT000217000.1	Klebsiella_11	PDS000005579.7	nan	NA	PRJNA292902
GCA 022316285	PDT000111520.2	Klebsiella_11	PDS000005579.7	2014	USA	PRJNA312972
GCA 022316195	PDT000111521.2	Klebsiella_11	PDS000005579.7	2013	Germany	PRJNA312972
GCA 002189415	PDT000217894.1	Klebsiella_11	PDS000013916.2	2016	Tunisia	PRJNA360371
GCA 002189385	PDT000217893.1	Klebsiella_11	PDS000013916.2	2016	Tunisia	PRJNA360302
GCA 021965615	PDT001019819.1	Klebsiella_11		2018	Lebanon	PRJNA658369
GCA 043863345	**HBH_658_17**	Klebsiella_11	PDS000013916.2	2017	Tunisia	PRJNA1160997
GCA 900512615	PDT000369296.1	Klebsiella_11	PDS000077811.10	2013	Germany	PRJEB10018
GCA 004141975	PDT000460519.1	Klebsiella_11		2012	Canada	PRJNA481972
GCA 929618885	PDT001250889.1	Klebsiella_11	PDS000084821.3	2019	United Kingdom	PRJEB30134
GCA 042695245	PDT002404019.1	Klebsiella_11	PDS000140731.18	2024	USA	PRJNA288601
GCA 022186305	PDT000716558.1	Klebsiella_11		2016	Germany	PRJEB35890
GCA 021787715	PDT001227642.1	Klebsiella_11	PDS000103182.2	2014	United Kingdom	PRJNA788733
GCA 022139805	PDT001244322.1	Klebsiella_11	PDS000103182.2	2015	United Kingdom	PRJNA788733
GCA 022106595	PDT001244829.1	Klebsiella_11	PDS000103182.2	2015	United Kingdom	PRJNA788733

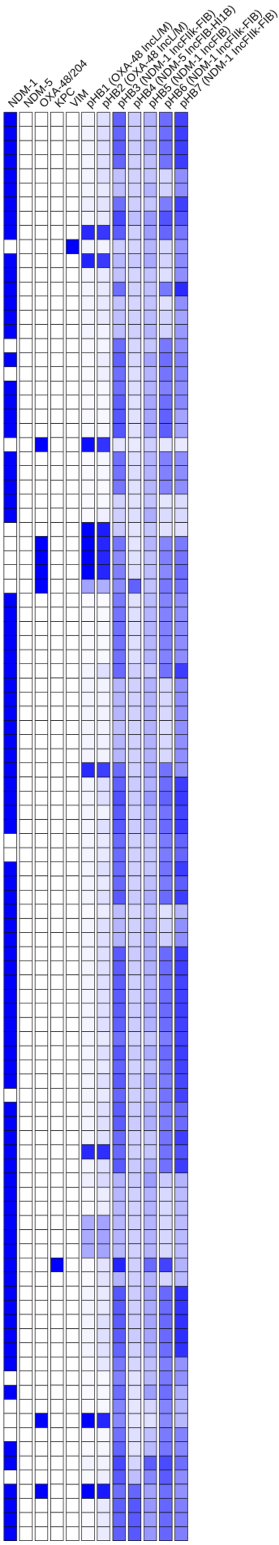

Tree 22: GCA_043863385

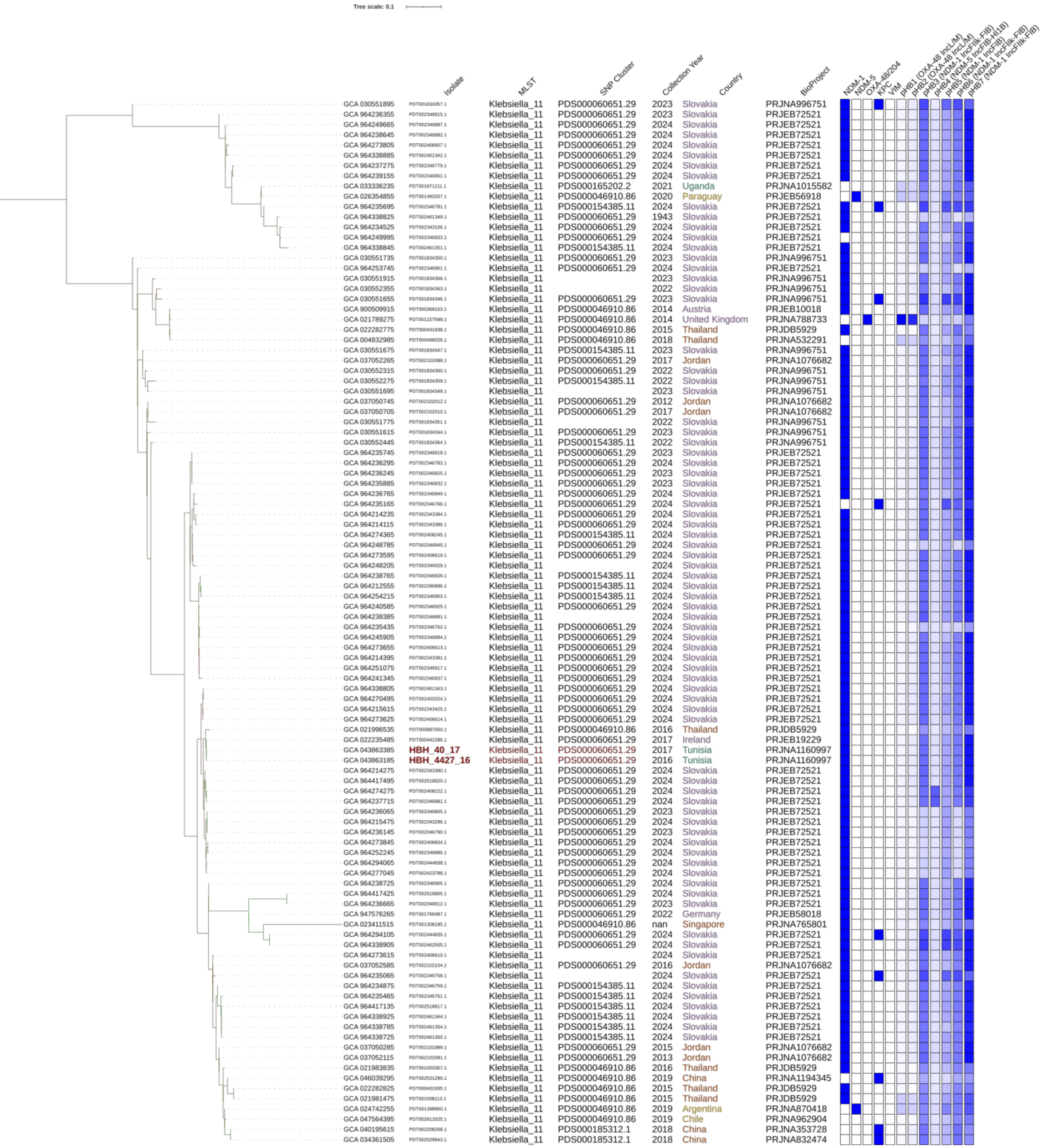

Tree 23: GCA_043863405

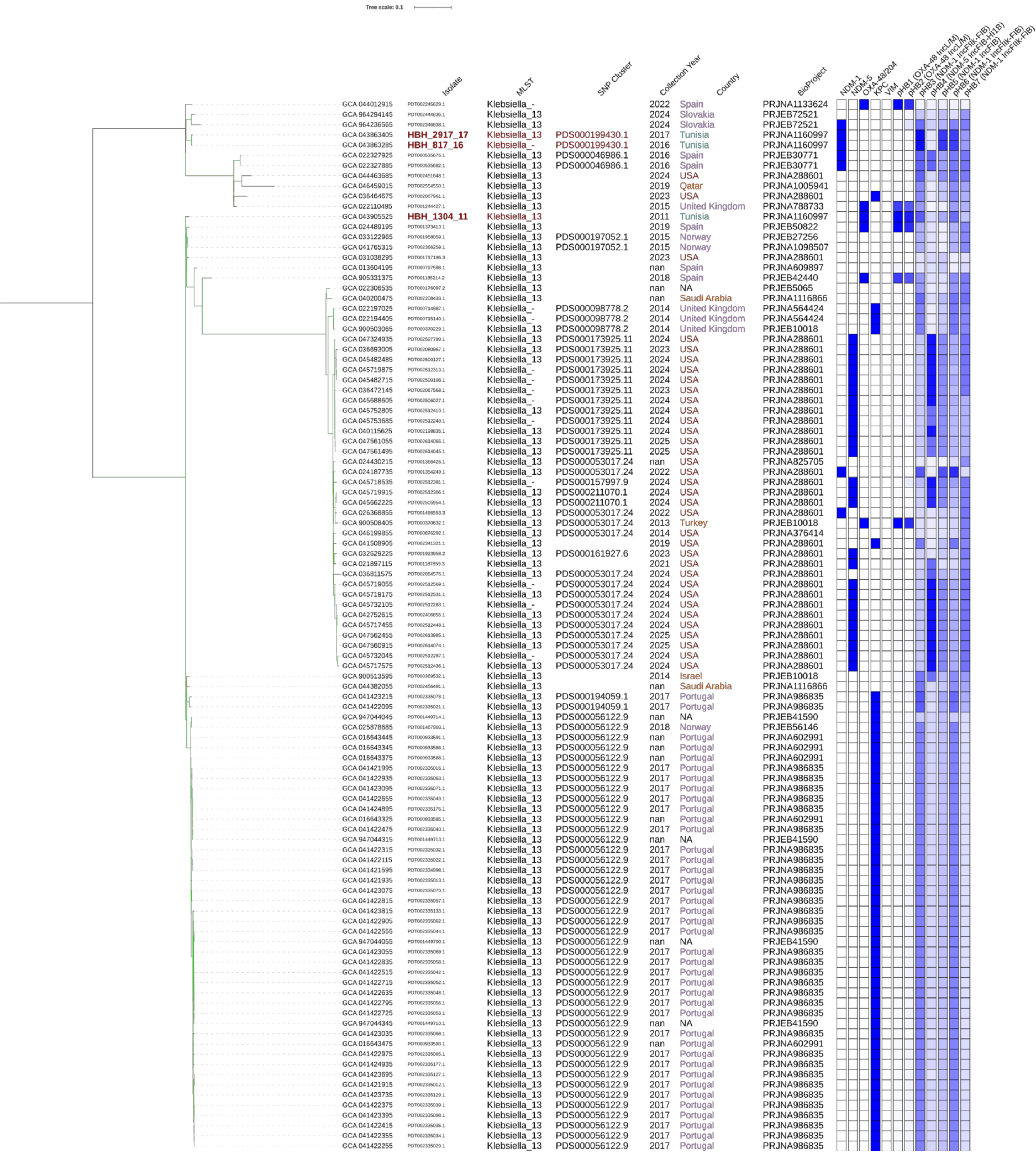

Tree 24: GCA_043863445

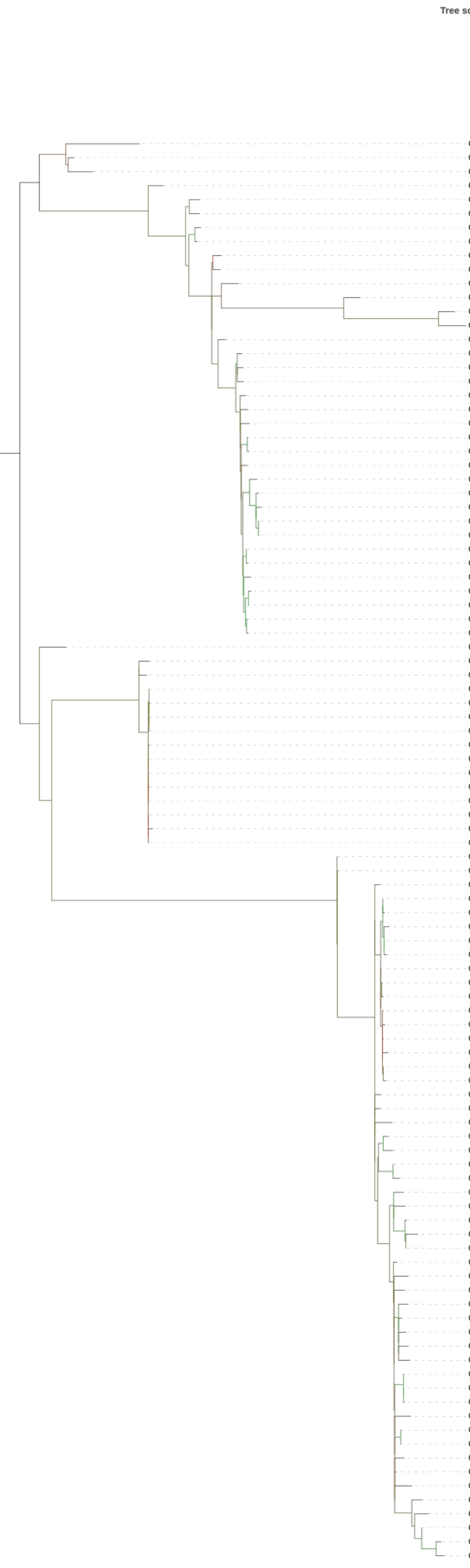

	Isolate	MLST	SNP Cluster	Collection Year	Country	BioProject
GCA 022054405	PDT000880924.1	Klebsiella_219	PDS000012112.164	2018	Qatar	PRJNA599387
GCA 900776315	PDT000489869.1	Klebsiella_219	PDS000012112.164	nan	NA	PRJEB31361
GCA 014282805	PDT000820954.1	Klebsiella_219	PDS000012112.164	2016	China	PRJNA657553
GCA 043328015	PDT002269108.1	Klebsiella_219		2018	Netherlands	PRJEB45369
GCA 024990555	PDT001410813.1	Klebsiella_219	PDS000179977.1	2020	Kenya	PRJNA777842
GCA 022198525	PDT000708422.1	Klebsiella_-	PDS000012112.164	2018	Italy	PRJEB27342
GCA 043975585	PDT002414006.1	Klebsiella_219	PDS000012112.164	2020	USA	PRJNA1106484
GCA 041509125	PDT002341307.1	Klebsiella_219	PDS000012112.164	2019	USA	PRJNA288601
GCA 021865095	PDT001208138.1	Klebsiella_219	PDS000012112.164	2019	Switzerland	PRJEB49326
GCA 031066135	PDT001703054.1	Klebsiella_-	PDS000012112.164	2020	Japan	PRJDB10842
GCA 021862845	PDT001208199.1	Klebsiella_219	PDS000012112.164	2017	Switzerland	PRJEB49326
GCA 047278345	PDT002595988.1	Klebsiella_219	PDS000012112.164	2019	Australia	PRJNA674961
GCA 022181965	PDT000730313.1	Klebsiella_219	PDS000012112.164	2017	Canada	PRJNA564992
GCA 027895535	PDT001557779.1	Klebsiella_219	PDS000012112.164	2020	India	PRJNA790720
GCA 041857965	PDT002366229.1	Klebsiella_219		2024	USA	PRJNA288601
GCA 034159145	PDT002016696.1	Klebsiella_219	PDS000012112.164	2023	USA	PRJNA475751
GCA 045733105	PDT002513468.1	Klebsiella_219	PDS000012112.164	2024	USA	PRJNA475751
GCA 045742925	PDT002513095.1	Klebsiella_219	PDS000012112.164	2024	USA	PRJNA475751
GCA 032122875	PDT001903553.2	Klebsiella_219	PDS000012112.164	2023	USA	PRJNA278886
GCA 047438955	PDT002608018.1	Klebsiella_219	PDS000217221.1	2024	USA	PRJNA288601
GCA 030675185	PDT001844513.1	Klebsiella_219	PDS000012112.164	2021	USA	PRJNA981469
GCA 045490455	PDT002188836.1	Klebsiella_219	PDS000012112.164	2023	Canada	PRJNA1070647
GCA 045490555	PDT002188828.1	Klebsiella_219	PDS000012112.164	2023	Canada	PRJNA1070647
GCA 031071365	PDT001701889.1	Klebsiella_219	PDS000012112.164	2019	Japan	PRJDB10842
GCA 044366365	PDT002455605.1	Klebsiella_219	PDS000012112.164	nan	Saudi Arabia	PRJNA1116866
GCA 036992945	PDT002098687.1	Klebsiella_219	PDS000012112.164	2016	China	PRJNA1003408
GCA 034389715	PDT002031463.1	Klebsiella_219	PDS000012112.164	2018	China	PRJNA832474
GCA 031318215	PDT001878347.1	Klebsiella_219	PDS000012112.164	2016	China	PRJNA914894
GCA 031318185	PDT001878346.1	Klebsiella_219	PDS000012112.164	2016	China	PRJNA914894
GCA 043327795	PDT002269116.1	Klebsiella_219	PDS000012112.164	2018	Netherlands	PRJEB45369
GCA 043302915	PDT002270523.1	Klebsiella_219	PDS000012112.164	2018	Netherlands	PRJEB45369
GCA 044467005	PDT002456719.1	Klebsiella_219		2022	Armenia	PRJNA1141898
GCA 040200615	PDT002208440.1	Klebsiella_219	PDS000012112.164	nan	Saudi Arabia	PRJNA1116866
GCA 043358755	PDT002423169.1	Klebsiella_219	PDS000012112.164	nan	Saudi Arabia	PRJNA1116866
GCA 044366105	PDT002455592.1	Klebsiella_219	PDS000012112.164	nan	Saudi Arabia	PRJNA1116866
GCA 044382305	PDT002456499.1	Klebsiella_219	PDS000012112.164	nan	Saudi Arabia	PRJNA1116866
GCA 044321605	PDT002311007.1	Klebsiella_219	PDS000012112.164	2021	Egypt	PRJEB76311
GCA 016527465	PDT000929088.1	Klebsiella_219	PDS000012112.164	nan	Portugal	PRJNA602991
GCA 033105465	PDT001960996.1	Klebsiella_219	PDS000012112.164	2022	USA	PRJNA648389
GCA 022638795	PDT001271018.1	Klebsiella_219	PDS000012112.164	2020	USA	PRJNA288601
GCA 022024715	PDT000932232.5	Klebsiella_219	PDS000012112.164	2020	USA	PRJNA288601
GCA 022024475	PDT000932240.5	Klebsiella_219	PDS000012112.164	2020	USA	PRJNA288601
GCA 021988195	PDT001002765.3	Klebsiella_219	PDS000012112.164	2020	USA	PRJNA288601
GCA 021988335	PDT001002759.3	Klebsiella_219	PDS000012112.164	2021	USA	PRJNA288601
GCA 022042935	PDT000892783.3	Klebsiella_219	PDS000012112.164	2020	USA	PRJNA288601
GCA 022042975	PDT000892782.5	Klebsiella_219	PDS000012112.164	2020	USA	PRJNA288601
GCA 021988255	PDT001002764.3	Klebsiella_219	PDS000012112.164	2020	USA	PRJNA288601
GCA 022687485	PDT001274660.3	Klebsiella_219	PDS000012112.164	2020	USA	PRJNA288601
GCA 022042995	PDT000892780.5	Klebsiella_219	PDS000012112.164	2020	USA	PRJNA288601
GCA 022024575	PDT000932242.5	Klebsiella_-	PDS000012112.164	2020	USA	PRJNA288601
GCA 022042895	PDT000892786.3	Klebsiella_219	PDS000012112.164	2020	USA	PRJNA288601
GCA 029214885	PDT001661785.1	Klebsiella_219	PDS000012112.164	2016	United Kingdom	PRJNA943144
GCA 035828975	PDT002058690.1	Klebsiella_219	PDS000012112.164	2016	United Kingdom	PRJNA943144
GCA 023690725	PDT001323899.1	Klebsiella_219	PDS000012112.164	2018	Australia	PRJNA797179
GCA 022043055	PDT000892776.3	Klebsiella_219	PDS000012112.164	2020	USA	PRJNA288601
GCA 022043075	PDT000892777.3	Klebsiella_219	PDS000012112.164	2020	USA	PRJNA288601
GCA 021809935	PDT001223237.3	Klebsiella_219	PDS000012112.164	2021	USA	PRJNA288601
GCA 022024695	PDT000932225.3	Klebsiella_219	PDS000012112.164	2020	USA	PRJNA288601
GCA 022024835	PDT000932224.5	Klebsiella_219	PDS000012112.164	2020	USA	PRJNA288601
GCA 022043255	PDT000892767.5	Klebsiella_219	PDS000012112.164	2019	USA	PRJNA288601
GCA 022024495	PDT000932238.3	Klebsiella_219	PDS000012112.164	2020	USA	PRJNA288601
GCA 021823725	PDT001221211.3	Klebsiella_219	PDS000012112.164	2021	USA	PRJNA288601
GCA 022042955	PDT000892781.5	Klebsiella_219	PDS000012112.164	2020	USA	PRJNA288601
GCA 022024795	PDT001302147.2	Klebsiella_219	PDS000012112.164	2020	USA	PRJNA288601
GCA 022024655	PDT000932228.3	Klebsiella_219	PDS000012112.164	2020	USA	PRJNA288601
GCA 022024675	PDT000932229.5	Klebsiella_219	PDS000012112.164	2020	USA	PRJNA288601
GCA 022043035	PDT000892779.5	Klebsiella_219	PDS000012112.164	2020	USA	PRJNA288601
GCA 021988295	PDT001002761.3	Klebsiella_219	PDS000012112.164	2021	USA	PRJNA288601
GCA 034159485	PDT002016704.1	Klebsiella_219	PDS000012112.164	2023	USA	PRJNA475751
GCA 037029855	PDT002100089.1	Klebsiella_219	PDS000012112.164	2018	Russia	PRJNA1052416
GCA 043863445	**HBH_7_18**	Klebsiella_219		2018	Tunisia	PRJNA1160997
GCA 023647395	PDT001322071.1	Klebsiella_219	PDS000012112.164	2021	USA	PRJNA823025
GCA 043457995	PDT002421695.1	Klebsiella_219	PDS000012112.164	2019	USA	PRJNA288601
GCA 043321095	PDT002268760.1	Klebsiella_219	PDS000012112.164	2018	Netherlands	PRJEB45369
GCA 043790545	PDT002255690.1	Klebsiella_219	PDS000012112.164	2019	Czechia	PRJNA1130712
GCA 042854715	PDT002054337.1	Klebsiella_219	PDS000012112.164	2019	Colombia	PRJNA937403
GCA 021959785	PDT001020108.1	Klebsiella_-	PDS000012112.164	2018	Colombia	PRJNA658369
GCA 047566005	PDT002613111.1	Klebsiella_219	PDS000012112.164	2018	Chile	PRJNA962904
GCA 047568975	PDT002613056.1	Klebsiella_219	PDS000012112.164	2018	Chile	PRJNA962904
GCA 047566415	PDT002613078.1	Klebsiella_219	PDS000012112.164	2018	Chile	PRJNA962904
GCA 022086715	PDT000874785.1	Klebsiella_219	PDS000012112.164	2013	USA	PRJNA376414
GCA 025113345	PDT001411424.1	Klebsiella_219	PDS000012112.164	2015	Singapore	PRJNA577535
GCA 905217845	PDT001184992.2	Klebsiella_219	PDS000012112.164	2018	Spain	PRJEB42440
GCA 019990825	PDT001132729.1	Klebsiella_219	PDS000012112.164	2018	Turkey	PRJNA761902
GCA 900508675	PDT000370654.1	Klebsiella_219	PDS000012112.164	2014	Turkey	PRJEB10018
GCA 022208985	PDT000636293.1	Klebsiella_219	PDS000012112.164	2016	France	PRJNA551371
GCA 029878605	PDT001704835.1	Klebsiella_219	PDS000012112.164	2019	Japan	PRJDB10842
GCA 044365125	PDT002455545.1	Klebsiella_219	PDS000012112.164	nan	Saudi Arabia	PRJNA1116866
GCA 040986885	PDT002279655.1	Klebsiella_219	PDS000012112.164	nan	China	PRJNA1021041
GCA 041039485	PDT002292090.1	Klebsiella_219	PDS000012112.164	nan	China	PRJNA1021041
GCA 041040145	PDT002292123.1	Klebsiella_219	PDS000012112.164	nan	China	PRJNA1021041
GCA 900492975	PDT000354540.1	Klebsiella_219	PDS000012112.164	2016	Thailand	PRJEB19226
GCA 026017005	PDT001474895.1	Klebsiella_219	PDS000012112.164	2019	Australia	PRJNA680857
GCA 026016935	PDT001474898.1	Klebsiella_219	PDS000012112.164	2018	Australia	PRJNA680857
GCA 044445085	PDT002456709.1	Klebsiella_219	PDS000012112.164	2015	China	PRJNA1056658
GCA 033106365	PDT001960951.1	Klebsiella_219	PDS000012112.164	2017	USA	PRJNA648389
GCA 028561655	PDT001610751.1	Klebsiella_219	PDS000012112.164	nan	NA	PRJNA919159
GCA 902508905	PDT000663016.1	Klebsiella_219	PDS000012112.164	2017	China	PRJEB34922
GCA 902509425	PDT000663055.1	Klebsiella_219	PDS000012112.164	2017	China	PRJEB34922
GCA 003346675	PDT000355817.1	Klebsiella_219	PDS000012112.164	2013	Algeria	PRJNA482637
GCA 003346685	PDT000355818.1	Klebsiella_219	PDS000012112.164	2013	Algeria	PRJNA482637
GCA 003346645	PDT000355816.1	Klebsiella_219	PDS000012112.164	2013	Algeria	PRJNA482637

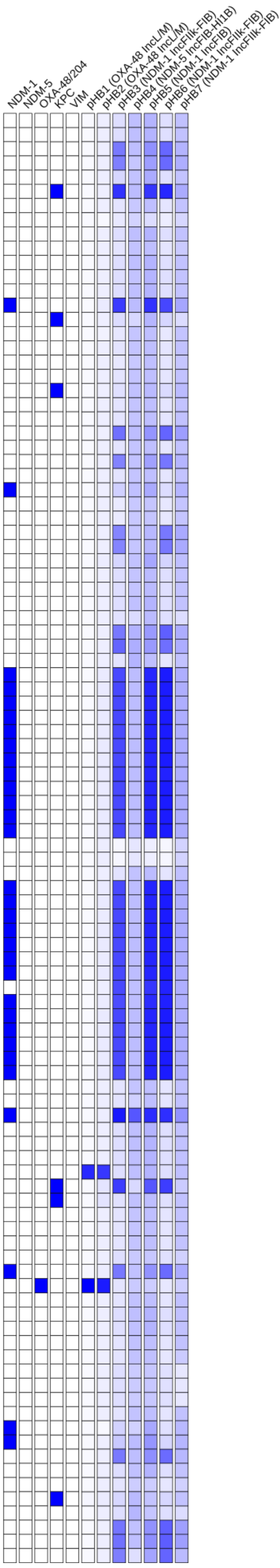

Tree 25: GCA_043863535

Tree scale: 0.1

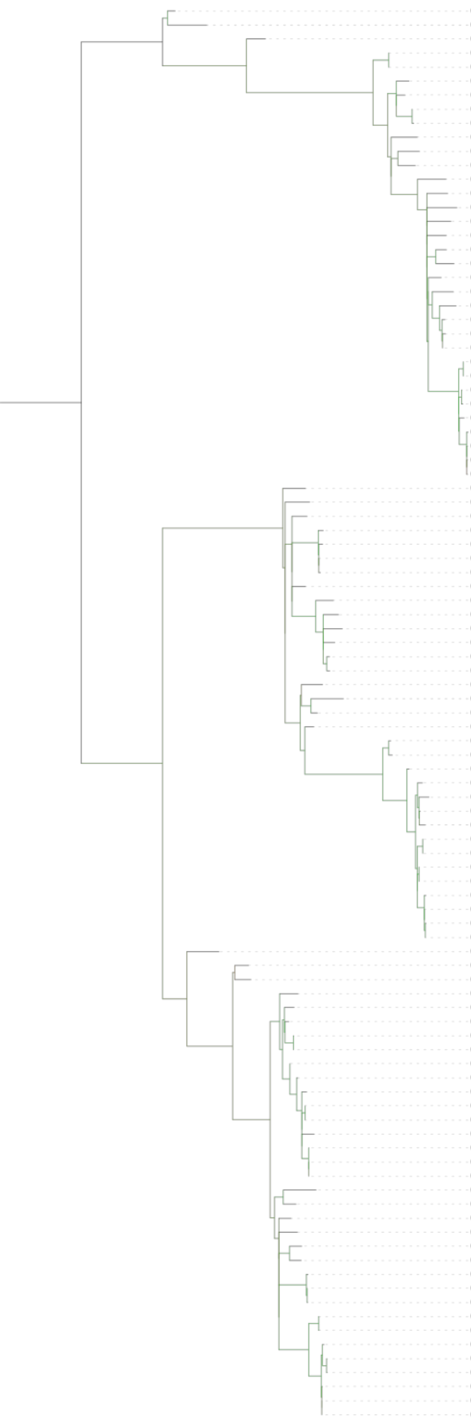

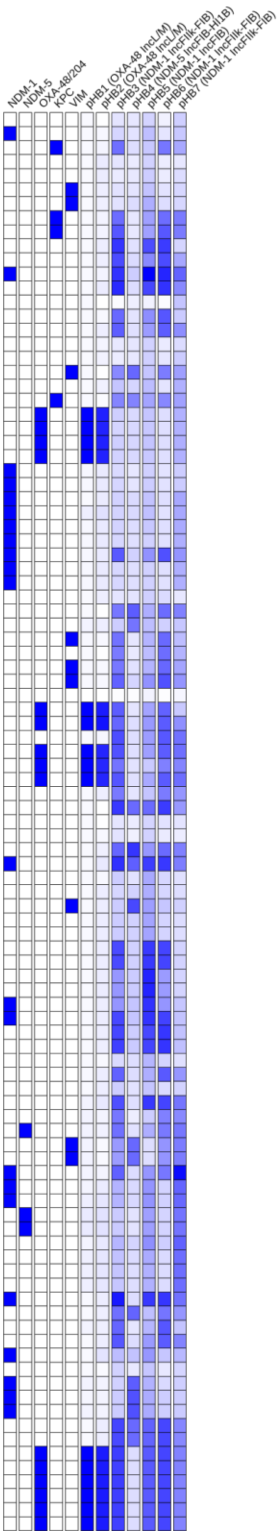

	Isolate	MLST	SNP Cluster	Collection Year	Country	BioProject
GCA 016635355	PDT000932944.1	Klebsiella_147		2015	Australia	PRJNA480723
GCA 025037385	PDT001386114.1	Klebsiella_147		2019	Canada	PRJNA855907
GCA 031628955	PDT001891056.1	Klebsiella_147		2021	Thailand	PRJNA525849
GCA 022212225	PDT000630103.1	Klebsiella_147	PDS000053519.1	2010	Canada	PRJNA564992
GCA 022212665	PDT000628739.1	Klebsiella_147	PDS000053519.1	2009	Canada	PRJNA564992
GCA 020120075	PDT001137082.1	Klebsiella_147	PDS000075223.4	2014	Switzerland	PRJNA543274
GCA 022104055	PDT001243382.1	Klebsiella_147	PDS000104526.1	2015	United Kingdom	PRJNA788733
GCA 902156305	PDT000663206.1	Klebsiella_147	PDS000036186.4	2014	Luxembourg	PRJEB33308
GCA 900503645	PDT000370117.1	Klebsiella_147	PDS000036186.4	2014	Luxembourg	PRJEB10018
GCA 900513535	PDT000369525.1	Klebsiella_147		2013	Israel	PRJEB10018
GCA 022276345	PDT000431990.1	Klebsiella_147	PDS000040359.4	2016	Vietnam	PRJDB6407
GCA 900502585	PDT000370025.1	Klebsiella_147	PDS000156049.11	2014	Denmark	PRJEB10018
GCA 012971605	PDT000730631.1	Klebsiella_147		2016	South Korea	PRJNA625837
GCA 040191035	PDT002208194.1	Klebsiella_147		nan	Saudi Arabia	PRJNA1116866
GCA 029224285	PDT001663092.1	Klebsiella_147		2019	China	PRJNA838419
GCA 022089675	PDT000874576.1	Klebsiella_147	PDS000074889.1	2012	USA	PRJNA376414
GCA 032134505	PDT001903152.1	Klebsiella_147		2018	Norway	PRJEB48268
GCA 022182885	PDT000724039.1	Klebsiella_147		2018	Italy	PRJEB27342
GCA 916161875	PDT001141251.1	Klebsiella_147		2013	Netherlands	PRJEB41042
GCA 022306355	PDT000176139.2	Klebsiella_147	PDS000006642.4	nan	NA	PRJEB5065
GCA 031624155	PDT001891027.1	Klebsiella_147		2020	China	PRJNA845066
GCA 020579485	PDT001161797.1	Klebsiella_147	PDS000097792.5	2014	Spain	PRJNA718833
GCA 043625685	PDT002332973.1	Klebsiella_147	PDS000099727.3	nan	Spain	PRJEB55414
GCA 900517095	PDT000369887.1	Klebsiella_147	PDS000099727.3	2014	Spain	PRJEB10018
GCA 043625465	PDT002332979.1	Klebsiella_147	PDS000099727.3	nan	Spain	PRJEB55414
GCA 022001215	PDT000987012.1	Klebsiella_147	PDS000083378.2	2016	Thailand	PRJDB5929
GCA 022001185	PDT000987015.1	Klebsiella_147	PDS000083378.2	2016	Thailand	PRJDB5929
GCA 022282305	PDT000431960.1	Klebsiella_147	PDS000083378.2	2016	Thailand	PRJDB5929
GCA 021983395	PDT001003375.1	Klebsiella_147	PDS000083378.2	2016	Thailand	PRJDB5929
GCA 022005405	PDT000986943.1	Klebsiella_147	PDS000083378.2	2015	Thailand	PRJDB5929
GCA 021985635	PDT001003212.1	Klebsiella_147	PDS000084451.3	2015	Thailand	PRJDB5929
GCA 021987055	PDT001003115.1	Klebsiella_147	PDS000084451.3	2015	Thailand	PRJDB5929
GCA 021985595	PDT001003215.1	Klebsiella_147	PDS000084451.3	2015	Thailand	PRJDB5929
GCA 021982475	PDT001008034.1	Klebsiella_147	PDS000084451.3	2015	Thailand	PRJDB5929
GCA 033550765	PDT001979154.1	Klebsiella_147	PDS000165990.1	2017	Germany	PRJEB67440
GCA 956464875	PDT001820175.1	Klebsiella_147		2019	Portugal	PRJEB62836
GCA 900507055	PDT000370555.1	Klebsiella_147		2014	Belgium	PRJEB10018
GCA 916161525	PDT001141234.1	Klebsiella_147	PDS000036182.26	2013	Netherlands	PRJEB41042
GCA 024527215	PDT001373245.1	Klebsiella_147	PDS000036182.26	2019	Spain	PRJEB50822
GCA 900516415	PDT000369783.1	Klebsiella_147	PDS000036182.26	2013	Spain	PRJEB10018
GCA 900501685	PDT000369810.1	Klebsiella_147	PDS000036182.26	2014	Spain	PRJEB10018
GCA 032042535	PDT001903163.1	Klebsiella_147		2017	Norway	PRJEB48268
GCA 022240035	PDT000441629.1	Klebsiella_147		2011	Germany	PRJEB18621
GCA 043863535	**HBH_2232_18**	Klebsiella_147		2018	Tunisia	PRJNA1160997
GCA 034513615	PDT002038633.1	Klebsiella_147		2019	France	PRJNA876630
GCA 022237725	PDT000441691.1	Klebsiella_147		2012	Germany	PRJEB18621
GCA 022241025	PDT000441623.1	Klebsiella_147	PDS000080300.2	2011	Germany	PRJEB18621
GCA 022987485	PDT001286510.1	Klebsiella_147	PDS000080300.2	2011	Germany	PRJNA789142
GCA 022181605	PDT000732046.1	Klebsiella_147		2017	Canada	PRJNA564992
GCA 900517655	PDT000369912.1	Klebsiella_147	PDS000036295.1	2013	Spain	PRJEB10018
GCA 022246065	PDT000441038.1	Klebsiella_147		nan	NA	PRJEB12699
GCA 900510435	PDT000369189.1	Klebsiella_147		2014	France	PRJEB10018
GCA 027610945	PDT001558347.1	Klebsiella_147	PDS000130855.1	2007	Bangladesh	PRJNA845975
GCA 027625855	PDT001558113.1	Klebsiella_147	PDS000130855.1	2013	Bangladesh	PRJNA845975
GCA 027615665	PDT001558328.1	Klebsiella_147	PDS000009779.14	2006	Bangladesh	PRJNA845975
GCA 900512445	PDT000369279.1	Klebsiella_147	PDS000009779.14	2014	Germany	PRJEB10018
GCA 030912625	PDT001865998.1	Klebsiella_147	PDS000157007.2	2019	Poland	PRJNA1005055
GCA 900511365	PDT000369134.1	Klebsiella_147	PDS000009779.14	2014	Poland	PRJEB10018
GCA 900511505	PDT000369153.1	Klebsiella_147	PDS000009779.14	2013	Poland	PRJEB10018
GCA 003856595	PDT000414016.1	Klebsiella_147	PDS000045262.1	2009	Sweden	PRJNA413669
GCA 003181055	PDT000323199.1	Klebsiella_147	PDS000045262.1	nan	Sweden	PRJNA472712
GCA 022320785	PDT001251452.1	Klebsiella_147	PDS000009779.14	nan	United Kingdom	PRJNA787440
GCA 034651715	PDT002040257.1	Klebsiella_147	PDS000009779.14	nan	NA	PRJNA1017778
GCA 022295635	PDT000423657.1	Klebsiella_147	PDS000009779.14	nan	NA	PRJEB17615
GCA 022295855	PDT000423646.1	Klebsiella_147	PDS000009779.14	nan	NA	PRJEB17615
GCA 022298095	PDT000423521.1	Klebsiella_147	PDS000009779.14	nan	NA	PRJEB17615
GCA 022298135	PDT000423515.1	Klebsiella_147	PDS000009779.14	nan	NA	PRJEB17615
GCA 022298835	PDT000423475.1	Klebsiella_147	PDS000100389.1	nan	NA	PRJEB17615
GCA 900084235	PDT000155118.1	Klebsiella_147		2006	United Kingdom	PRJEB5065
GCA 900517045	PDT000369761.1	Klebsiella_147		2013	Hungary	PRJEB10018
GCA 022211825	PDT000630648.1	Klebsiella_147	PDS000164093.1	2013	Canada	PRJNA564992
GCA 030821325	PDT001702632.1	Klebsiella_147		2019	Japan	PRJDB10842
GCA 041003655	PDT002281177.1	Klebsiella_147		2021	China	PRJNA1135755
GCA 956477775	PDT001861559.1	Klebsiella_147	PDS000106769.6	2018	Greece	PRJEB63349
GCA 028089455	PDT001585616.1	Klebsiella_147	PDS000106769.6	2018	Greece	PRJEB58216
GCA 021815755	PDT001219543.1	Klebsiella_147	PDS000006578.119	2011	Canada	PRJNA725227
GCA 025116845	PDT001411104.1	Klebsiella_147	PDS000006578.119	2011	Singapore	PRJNA577535
GCA 022230785	PDT000445955.1	Klebsiella_147	PDS000006578.119	2013	Canada	PRJNA390933
GCA 046202785	PDT000435261.1	Klebsiella_147	PDS000006578.119	2014	Singapore	PRJNA342893
GCA 029492505	PDT001672901.1	Klebsiella_147	PDS000006578.119	nan	Singapore	PRJNA765801
GCA 002192375	PDT000218724.1	Klebsiella_147	PDS000006578.119	nan	NA	PRJNA292902
GCA 022230605	PDT000445964.1	Klebsiella_147	PDS000006578.119	2014	Canada	PRJNA390933
GCA 043977955	PDT002412003.1	Klebsiella_147	PDS000006578.119	2014	Canada	PRJNA855907
GCA 022230585	PDT000445967.1	Klebsiella_147	PDS000006578.119	2014	Canada	PRJNA390933
GCA 027827355	PDT001558040.1	Klebsiella_147	PDS000100083.5	2020	Bangladesh	PRJNA845975
GCA 900086515	PDT000131554.1	Klebsiella_147		2009	United Kingdom	PRJEB5065
GCA 022246125	PDT000441036.1	Klebsiella_-		nan	NA	PRJEB12699
GCA 022212825	PDT000628215.1	Klebsiella_147		2008	Canada	PRJNA564992
GCA 022293415	PDT000423788.1	Klebsiella_147	PDS000052092.1	nan	NA	PRJEB17615
GCA 022298575	PDT000423490.1	Klebsiella_147		nan	NA	PRJEB17615
GCA 030372715	PDT001792663.1	Klebsiella_147	PDS000065404.6	2018	Peru	PRJNA865026
GCA 032635685	PDT001923307.1	Klebsiella_147	PDS000065404.6	2017	Peru	PRJNA990326
GCA 032636005	PDT001923285.1	Klebsiella_147	PDS000065404.6	2017	Peru	PRJNA990326
GCA 041765295	PDT002366257.1	Klebsiella_147	PDS000197072.1	2013	Norway	PRJNA1098507
GCA 021917705	PDT001092378.1	Klebsiella_147	PDS000197072.1	2013	Norway	PRJEB27256
GCA 027904215	PDT001570538.1	Klebsiella_147	PDS000132251.1	nan	Spain	PRJNA918858
GCA 027907115	PDT001570542.1	Klebsiella_147	PDS000132251.1	nan	Spain	PRJNA918858
GCA 027907135	PDT001570541.1	Klebsiella_147	PDS000132251.1	nan	Spain	PRJNA918858
GCA 027904195	PDT001570539.1	Klebsiella_147	PDS000132251.1	nan	Spain	PRJNA918858
GCA 027904235	PDT001570537.1	Klebsiella_147	PDS000132251.1	nan	Spain	PRJNA918858
GCA 027907155	PDT001570540.1	Klebsiella_147	PDS000132251.1	nan	Spain	PRJNA918858

Tree 26: GCA_043904805

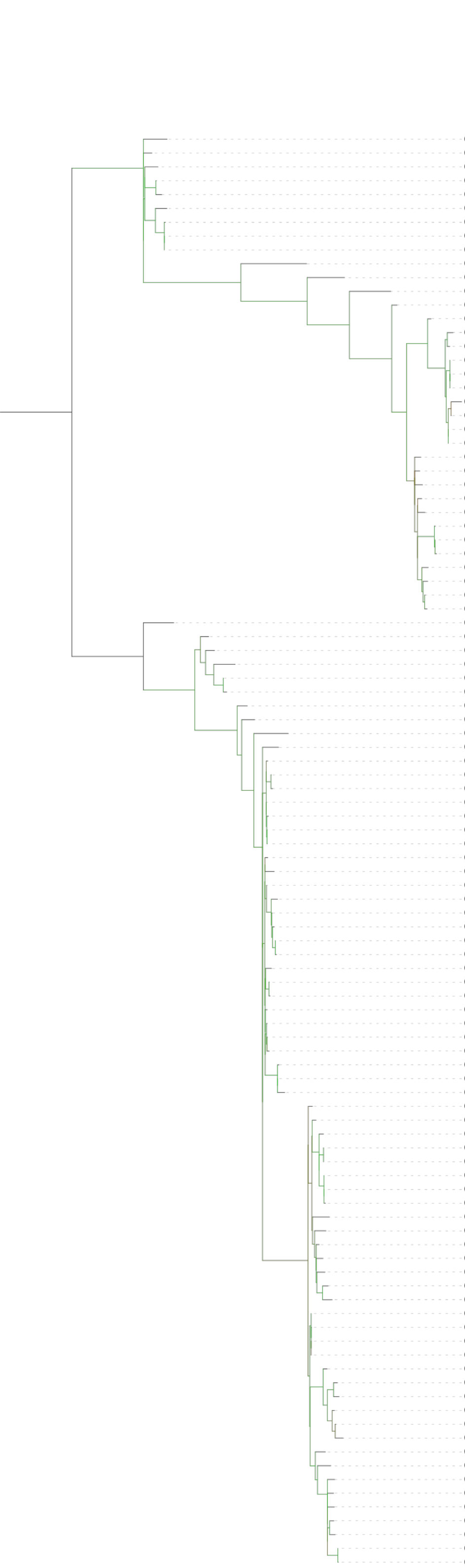

	Isolate	MLST	SNP Cluster	Collection Year	Country	BioProject
GCA 900560105	PDT000595231.1	Klebsiella_15		nan	United Kingdom	PRJEB28457
GCA 022247465	PDT000440938.1	Klebsiella_15		nan	NA	PRJEB12699
GCA 022275625	PDT000432631.1	Klebsiella_15	PDS000100772.1	2008	Ireland	PRJNA396729
GCA 010602475	PDT000685977.1	Klebsiella_15	PDS000156654.1	2015	USA	PRJNA553678
GCA 010598085	PDT000685960.1	Klebsiella_15	PDS000156654.1	2015	USA	PRJNA553678
GCA 947390215	PDT001514280.1	Klebsiella_15		2017	United Kingdom	PRJEB57368
GCA 037107655	PDT002109572.1	Klebsiella_15	PDS000184430.3	2024	USA	PRJNA288601
GCA 047123715	PDT002590027.1	Klebsiella_15	PDS000184430.3	2023	USA	PRJNA288601
GCA 040107955	PDT002192467.1	Klebsiella_15	PDS000184430.3	2024	USA	PRJNA288601
GCA 002248595	PDT000233416.1	Klebsiella_15		2016	Thailand	PRJNA389557
GCA 022182505	PDT000729794.1	Klebsiella_15		2016	Canada	PRJNA564992
GCA 047277665	PDT002596029.1	Klebsiella_15		2019	Australia	PRJNA674961
GCA 002866645	PDT000277132.1	Klebsiella_15	PDS000045329.17	2013	Lebanon	PRJNA388272
GCA 000707985	PDT000033733.1	Klebsiella_15	PDS000045329.17	2012	NA	PRJNA202045
GCA 047280045	PDT002595881.1	Klebsiella_-		2018	Australia	PRJNA674961
GCA 022181865	PDT000730424.1	Klebsiella_-		2017	Canada	PRJNA564992
GCA 015643945	PDT000896252.1	Klebsiella_15	PDS000045329.17	2015	Spain	PRJNA641166
GCA 022987385	PDT001286532.1	Klebsiella_15	PDS000045329.17	2014	Spain	PRJNA803387
GCA 027866245	PDT001562003.1	Klebsiella_15	PDS000045329.17	2018	Spain	PRJNA803387
GCA 047569615	PDT002612972.1	Klebsiella_15	PDS000045329.17	2018	Chile	PRJNA962904
GCA 022987425	PDT001286523.1	Klebsiella_15	PDS000045329.17	2014	Spain	PRJNA803387
GCA 015643785	PDT000896244.1	Klebsiella_15	PDS000045329.17	2015	Spain	PRJNA641166
GCA 027866345	PDT001561998.1	Klebsiella_15	PDS000045329.17	2018	Spain	PRJNA803387
GCA 021980855	PDT001009146.1	Klebsiella_15	PDS000045329.17	2018	Italy	PRJEB27342
GCA 037051865	PDT002102068.1	Klebsiella_15	PDS000045329.17	2015	Jordan	PRJNA1076682
GCA 037395695	PDT002116651.1	Klebsiella_15	PDS000179049.7	2015	USA	PRJNA288601
GCA 022212805	PDT000628216.1	Klebsiella_15	PDS000045329.17	2007	Canada	PRJNA564992
GCA 022244675	PDT000441141.1	Klebsiella_15	PDS000046916.2	2012	Nepal	PRJEB1800
GCA 916172225	PDT001141321.1	Klebsiella_15	PDS000045329.17	2012	Netherlands	PRJEB44242
GCA 916163945	PDT001141299.1	Klebsiella_15	PDS000045329.17	2013	Netherlands	PRJEB41042
GCA 916163895	PDT001141294.1	Klebsiella_15	PDS000045329.17	2013	Netherlands	PRJEB41042
GCA 022297035	PDT000423575.1	Klebsiella_15		nan	NA	PRJEB17615
GCA 022313055	PDT000136320.2	Klebsiella_15	PDS000045329.17	2011	United Kingdom	PRJNA306133
GCA 900506155	PDT000370361.1	Klebsiella_15	PDS000045329.17	2014	Croatia	PRJEB10018
GCA 022288065	PDT000427635.1	Klebsiella_15	PDS000045329.17	nan	NA	PRJEB10018
GCA 043859485	**HBH_545_12**	Klebsiella_15	PDS000041726.21	2012	Tunisia	PRJNA1160997
GCA 900505905	PDT000370325.1	Klebsiella_15	PDS000045329.17	2014	Croatia	PRJEB10018
GCA 043863125	**HBH_1334_16**	Klebsiella_15	PDS000199428.1	2016	Tunisia	PRJNA1160997
GCA 036236705	PDT002066778.1	Klebsiella_15		2021	Russia	PRJNA729628
GCA 046465275	PDT002556489.1	Klebsiella_15	PDS000214445.1	nan	NA	PRJNA786399
GCA 046466215	PDT002556535.1	Klebsiella_15	PDS000214445.1	nan	NA	PRJNA786399
GCA 037387465	PDT002117100.1	Klebsiella_15		2014	USA	PRJNA288601
GCA 022258775	PDT000437222.1	Klebsiella_15		2008	Laos	PRJEB2111
GCA 026369015	PDT001496549.3	Klebsiella_15		2022	USA	PRJNA288601
GCA 022237205	PDT000441700.1	Klebsiella_15	PDS000124938.1	2013	Germany	PRJEB18621
GCA 010179715	PDT000679377.1	Klebsiella_15	PDS000041726.21	2016	USA	PRJNA589891
GCA 010179795	PDT000679380.1	Klebsiella_15	PDS000041726.21	2015	USA	PRJNA589891
GCA 010179845	PDT000679383.1	Klebsiella_15	PDS000041726.21	2015	USA	PRJNA589891
GCA 010179725	PDT000679378.1	Klebsiella_15	PDS000041726.21	2014	USA	PRJNA589891
GCA 010179805	PDT000679381.1	Klebsiella_15	PDS000041726.21	2015	USA	PRJNA589891
GCA 010179775	PDT000679379.1	Klebsiella_15	PDS000041726.21	2016	USA	PRJNA589891
GCA 010179835	PDT000679382.1	Klebsiella_15	PDS000041726.21	2016	USA	PRJNA589891
GCA 022076105	PDT000875427.1	Klebsiella_15	PDS000041726.21	2012	USA	PRJNA376414
GCA 022067625	PDT000875828.1	Klebsiella_15	PDS000041726.21	2014	USA	PRJNA376414
GCA 022089835	PDT000874562.1	Klebsiella_15	PDS000041726.21	2013	USA	PRJNA376414
GCA 022063665	PDT000876071.1	Klebsiella_15	PDS000041726.21	2013	USA	PRJNA376414
GCA 022071465	PDT000875628.1	Klebsiella_15	PDS000041726.21	2013	USA	PRJNA376414
GCA 022081255	PDT000875103.1	Klebsiella_15	PDS000041726.21	2014	USA	PRJNA376414
GCA 022067865	PDT000875810.1	Klebsiella_15	PDS000041726.21	2014	USA	PRJNA376414
GCA 022068305	PDT000875786.1	Klebsiella_15	PDS000041726.21	2014	USA	PRJNA376414
GCA 022064285	PDT000876037.1	Klebsiella_15	PDS000041726.21	2013	USA	PRJNA376414
GCA 022070945	PDT000875668.1	Klebsiella_15	PDS000041726.21	2013	USA	PRJNA376414
GCA 022070965	PDT000875666.1	Klebsiella_15	PDS000041726.21	2013	USA	PRJNA376414
GCA 022070885	PDT000875671.1	Klebsiella_15	PDS000041726.21	2013	USA	PRJNA376414
GCA 022072865	PDT000875587.1	Klebsiella_15	PDS000041726.21	2013	USA	PRJNA376414
GCA 022071505	PDT000875631.1	Klebsiella_15	PDS000041726.21	2013	USA	PRJNA376414
GCA 022072825	PDT000875589.1	Klebsiella_15	PDS000041726.21	2013	USA	PRJNA376414
GCA 022060845	PDT000876223.1	Klebsiella_15	PDS000041726.21	2013	USA	PRJNA376414
GCA 022061705	PDT000876180.1	Klebsiella_15	PDS000041726.21	2015	USA	PRJNA376414
GCA 022060865	PDT000876225.1	Klebsiella_15	PDS000041726.21	2013	USA	PRJNA376414
GCA 024918875	PDT001409690.1	Klebsiella_15	PDS000041726.21	2016	USA	PRJNA836696
GCA 022060825	PDT000876226.1	Klebsiella_15	PDS000041726.21	2015	USA	PRJNA376414
GCA 031038415	PDT001716002.3	Klebsiella_15		2023	USA	PRJNA288601
GCA 021904015	PDT001149771.1	Klebsiella_15	PDS000097194.4	2021	USA	PRJNA288601
GCA 021904075	PDT001148880.3	Klebsiella_15	PDS000097194.4	2021	USA	PRJNA288601
GCA 040735645	PDT002255402.1	Klebsiella_15	PDS000188634.2	2024	USA	PRJNA288601
GCA 039549035	PDT002156713.1	Klebsiella_15	PDS000188634.2	2024	USA	PRJNA288601
GCA 041213875	PDT002310441.1	Klebsiella_15	PDS000188634.2	2024	USA	PRJNA288601
GCA 022030895	PDT000921052.1	Klebsiella_15		nan	NA	PRJEB27342
GCA 028883295	PDT001647973.1	Klebsiella_15		nan	NA	PRJNA915079
GCA 043859735	**HBH_600_13**	Klebsiella_15	PDS000041726.21	2013	Tunisia	PRJNA1160997
GCA 022187525	PDT000715769.1	Klebsiella_15	PDS000060613.1	2018	Saudi Arabia	PRJEB36683
GCA 022103415	PDT001244308.1	Klebsiella_15		2014	United Kingdom	PRJNA788733
GCA 002189165	PDT000217883.1	Klebsiella_15		2016	Tunisia	PRJNA360233
GCA 043904805	**HBH_363_18**	Klebsiella_15		2018	Tunisia	PRJNA1160997
GCA 022028755	PDT000921131.1	Klebsiella_15	PDS000041726.21	nan	NA	PRJEB27342
GCA 022231935	PDT000443857.1	Klebsiella_15	PDS000041726.21	nan	NA	PRJEB8666
GCA 022231425	PDT000443996.1	Klebsiella_15	PDS000041726.21	nan	NA	PRJEB8666
GCA 022231985	PDT000443854.1	Klebsiella_15	PDS000041726.21	nan	NA	PRJEB8666
GCA 002152405	PDT000210736.1	Klebsiella_2147	PDS000041726.21	2015	USA	PRJNA271899
GCA 001033755	PDT000068099.1	Klebsiella_2147	PDS000041726.21	2014	NA	PRJNA271899
GCA 001033885	PDT000068105.1	Klebsiella_15	PDS000041726.21	2014	NA	PRJNA271899
GCA 014903595	PDT000865015.1	Klebsiella_15	PDS000041726.21	nan	USA	PRJNA271899
GCA 001033455	PDT000068086.1	Klebsiella_-	PDS000041726.21	2014	NA	PRJNA271899
GCA 000688215	PDT000030152.1	Klebsiella_15	PDS000041726.21	2013	USA	PRJNA234115
GCA 956481235	PDT001864012.1	Klebsiella_-	PDS000041726.21	2016	Spain	PRJEB63349
GCA 024488485	PDT001373087.1	Klebsiella_15		2019	Spain	PRJEB50822
GCA 028561035	PDT001610683.1	Klebsiella_15	PDS000106381.4	2020	Spain	PRJEB57245
GCA 040931645	PDT002275246.1	Klebsiella_15	PDS000106381.4	2022	Spain	PRJNA1132269
GCA 905329805	PDT001185122.2	Klebsiella_15	PDS000106381.4	2018	Spain	PRJEB42440
GCA 028562035	PDT001610675.1	Klebsiella_-	PDS000106381.4	2020	Spain	PRJEB57245
GCA 024490915	PDT001373269.1	Klebsiella_15	PDS000106381.4	2019	Spain	PRJEB50822
GCA 028562135	PDT001610669.1	Klebsiella_15	PDS000106381.4	2020	Spain	PRJEB57245
GCA 028562055	PDT001610678.1	Klebsiella_15	PDS000106381.4	2020	Spain	PRJEB57245

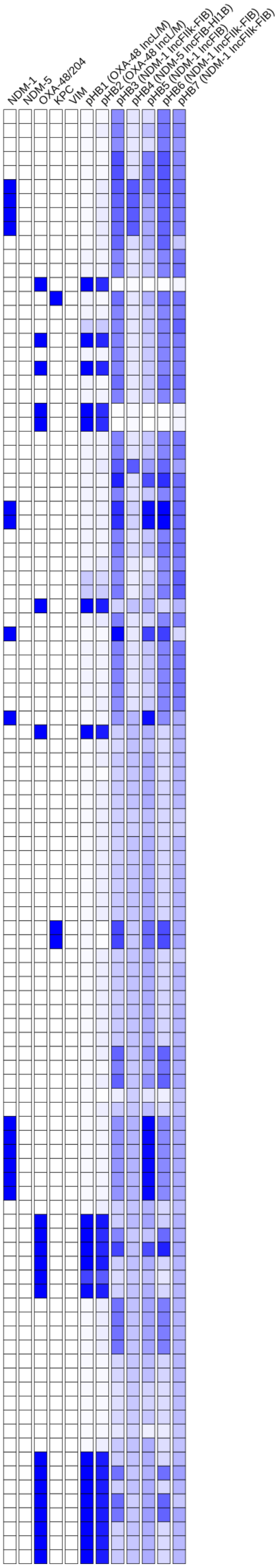

Tree 27: GCA_043904845

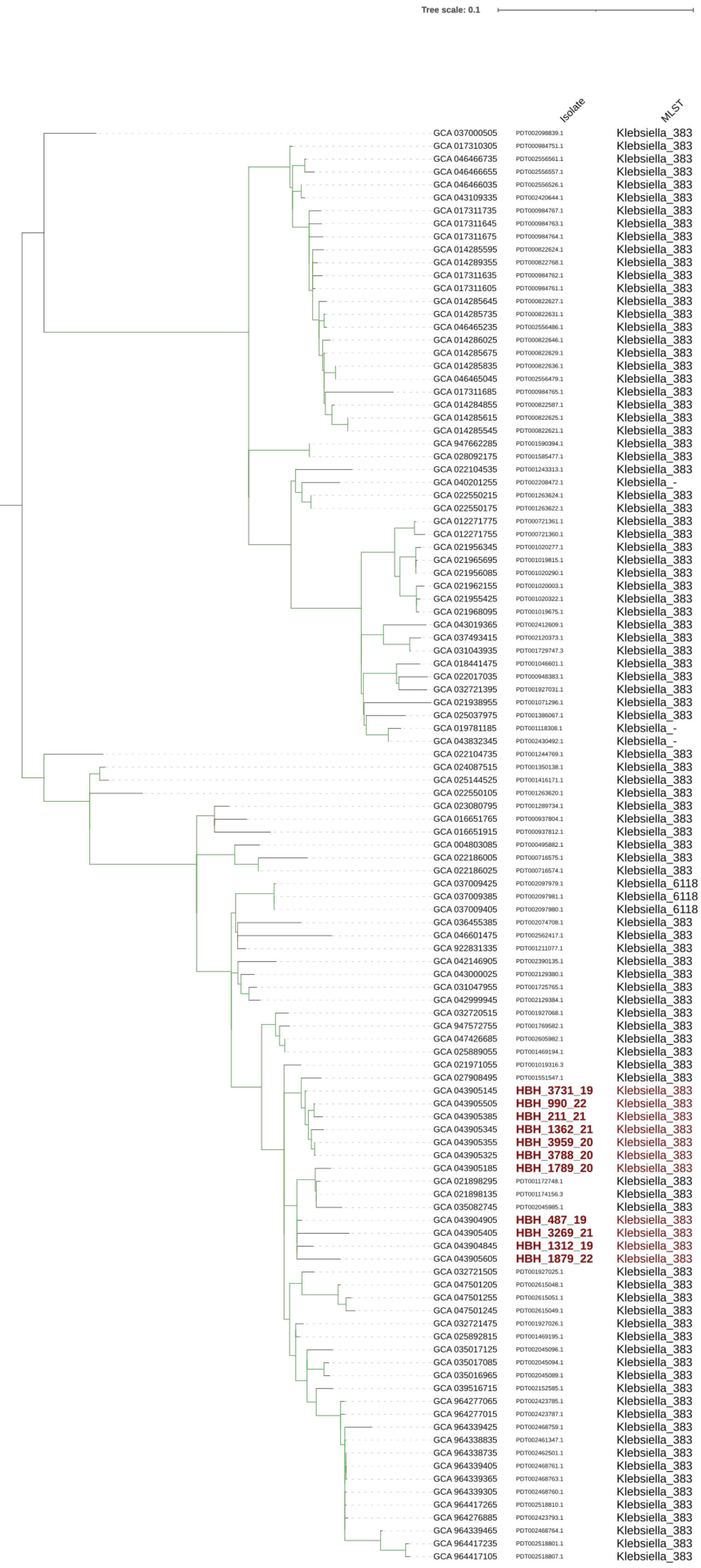

	Isolate	MLST	SNP Cluster	Collection Year	Country	BioProject
GCA 037000505	PDT002098839.1	Klebsiella_383		2015	Egypt	PRJNA1079765
GCA 017310305	PDT000984751.1	Klebsiella_383	PDS000070357.5	2013	China	PRJNA648010
GCA 046466735	PDT002556561.1	Klebsiella_383	PDS000070357.5	nan	NA	PRJNA786399
GCA 046466655	PDT002556557.1	Klebsiella_383	PDS000070357.5	nan	NA	PRJNA786399
GCA 046466035	PDT002556526.1	Klebsiella_383	PDS000070357.5	nan	NA	PRJNA786399
GCA 043109335	PDT002420644.1	Klebsiella_383	PDS000070357.5	2014	China	PRJNA1170063
GCA 017311735	PDT000984767.1	Klebsiella_383	PDS000070357.5	2013	China	PRJNA648010
GCA 017311645	PDT000984763.1	Klebsiella_383	PDS000070357.5	2014	China	PRJNA648010
GCA 017311675	PDT000984764.1	Klebsiella_383	PDS000070357.5	2017	China	PRJNA648010
GCA 014285595	PDT000822624.1	Klebsiella_383	PDS000070357.5	2015	China	PRJNA657553
GCA 014289355	PDT000822768.1	Klebsiella_383	PDS000070357.5	2013	China	PRJNA657553
GCA 017311635	PDT000984762.1	Klebsiella_383	PDS000070357.5	2014	China	PRJNA648010
GCA 017311605	PDT000984761.1	Klebsiella_383	PDS000070357.5	2013	China	PRJNA648010
GCA 014285645	PDT000822627.1	Klebsiella_383	PDS000070357.5	2015	China	PRJNA657553
GCA 014285735	PDT000822631.1	Klebsiella_383	PDS000070357.5	2014	China	PRJNA657553
GCA 046465235	PDT002556486.1	Klebsiella_383	PDS000070357.5	nan	NA	PRJNA786399
GCA 014286025	PDT000822646.1	Klebsiella_383	PDS000070357.5	2013	China	PRJNA657553
GCA 014285675	PDT000822629.1	Klebsiella_383	PDS000070357.5	2013	China	PRJNA657553
GCA 014285835	PDT000822636.1	Klebsiella_383	PDS000070357.5	2014	China	PRJNA657553
GCA 046465045	PDT002556479.1	Klebsiella_383	PDS000070357.5	nan	NA	PRJNA786399
GCA 017311685	PDT000984765.1	Klebsiella_383	PDS000070357.5	2017	China	PRJNA648010
GCA 014284855	PDT000822587.1	Klebsiella_383	PDS000070357.5	2015	China	PRJNA657553
GCA 014285615	PDT000822625.1	Klebsiella_383	PDS000070357.5	2015	China	PRJNA657553
GCA 014285545	PDT000822621.1	Klebsiella_383	PDS000070357.5	2015	China	PRJNA657553
GCA 947662285	PDT001590394.1	Klebsiella_383	PDS000133910.8	2007	Greece	PRJEB58216
GCA 028092175	PDT001585477.1	Klebsiella_383	PDS000133910.8	2007	Greece	PRJEB58216
GCA 022104535	PDT001243313.1	Klebsiella_383	PDS000106324.3	2016	United Kingdom	PRJNA788733
GCA 040201255	PDT002208472.1	Klebsiella_-		nan	Saudi Arabia	PRJNA1116866
GCA 022550215	PDT001263624.1	Klebsiella_383	PDS000106324.3	2019	Egypt	PRJNA720263
GCA 022550175	PDT001263622.1	Klebsiella_383	PDS000106324.3	2018	Egypt	PRJNA720263
GCA 012271775	PDT000721361.1	Klebsiella_383	PDS000061038.3	2018	Lebanon	PRJNA613441
GCA 012271755	PDT000721360.1	Klebsiella_383	PDS000061038.3	2018	Lebanon	PRJNA613441
GCA 021956345	PDT001020277.1	Klebsiella_383	PDS000061038.3	2018	Lebanon	PRJNA658369
GCA 021965695	PDT001019815.1	Klebsiella_383	PDS000061038.3	2018	Lebanon	PRJNA658369
GCA 021956085	PDT001020290.1	Klebsiella_383	PDS000061038.3	2018	Lebanon	PRJNA658369
GCA 021962155	PDT001020003.1	Klebsiella_383	PDS000061038.3	2018	Lebanon	PRJNA658369
GCA 021955425	PDT001020322.1	Klebsiella_383	PDS000061038.3	2018	Lebanon	PRJNA658369
GCA 021968095	PDT001019675.1	Klebsiella_383	PDS000061038.3	2018	Lebanon	PRJNA658369
GCA 043019365	PDT002412609.1	Klebsiella_383		2024	USA	PRJNA288601
GCA 037493415	PDT002120373.1	Klebsiella_383	PDS000179530.2	2024	USA	PRJNA288601
GCA 031043935	PDT001729747.3	Klebsiella_383	PDS000179530.2	2023	USA	PRJNA288601
GCA 018441475	PDT001046601.1	Klebsiella_383		2018	Germany	PRJNA692829
GCA 022017035	PDT000948383.1	Klebsiella_383		2019	Italy	PRJEB37610
GCA 032721395	PDT001927031.1	Klebsiella_383		2019	Italy	PRJNA1026096
GCA 021938955	PDT001071296.1	Klebsiella_383	PDS000106730.2	2019	Germany	PRJEB42370
GCA 025037975	PDT001386067.1	Klebsiella_383		2019	Canada	PRJNA855907
GCA 019781185	PDT001118308.1	Klebsiella_-	PDS000202475.1	2019	Germany	PRJNA634442
GCA 043832345	PDT002430492.1	Klebsiella_-	PDS000202475.1	2019	Germany	PRJNA1070536
GCA 022104735	PDT001244769.1	Klebsiella_383		2016	United Kingdom	PRJNA788733
GCA 024087515	PDT001350138.1	Klebsiella_383	PDS000108392.5	2016	Qatar	PRJNA656934
GCA 025144525	PDT001416171.1	Klebsiella_383	PDS000108392.5	2016	Qatar	PRJNA656934
GCA 022550105	PDT001263620.1	Klebsiella_383		2019	Egypt	PRJNA720263
GCA 023080795	PDT001289734.1	Klebsiella_383	PDS000108392.5	2018	Turkey	PRJNA789336
GCA 016651765	PDT000937804.1	Klebsiella_383	PDS000106324.3	nan	Egypt	PRJNA689413
GCA 016651915	PDT000937812.1	Klebsiella_383		nan	Egypt	PRJNA689413
GCA 004803085	PDT000495882.1	Klebsiella_383		nan	Egypt	PRJNA508509
GCA 022186005	PDT000716575.1	Klebsiella_383	PDS000060640.1	2019	Germany	PRJEB35890
GCA 022186025	PDT000716574.1	Klebsiella_383	PDS000060640.1	2019	Germany	PRJEB35890
GCA 037009425	PDT002097979.1	Klebsiella_6118	PDS000175841.1	2024	USA	PRJNA288601
GCA 037009385	PDT002097981.1	Klebsiella_6118	PDS000175841.1	2024	USA	PRJNA288601
GCA 037009405	PDT002097980.1	Klebsiella_6118	PDS000175841.1	2024	USA	PRJNA288601
GCA 036455385	PDT002074708.1	Klebsiella_383		2021	Germany	PRJNA1060526
GCA 046601475	PDT002562417.1	Klebsiella_383		2021	Singapore	PRJNA1206822
GCA 922831335	PDT001211077.1	Klebsiella_383	PDS000085592.11	2019	United Kingdom	PRJEB48990
GCA 042146905	PDT002390135.1	Klebsiella_383		2024	USA	PRJNA288601
GCA 043000025	PDT002129380.1	Klebsiella_383		2021	Egypt	PRJNA1071125
GCA 031047955	PDT001725765.1	Klebsiella_383	PDS000180184.1	2020	Egypt	PRJNA917066
GCA 042999945	PDT002129384.1	Klebsiella_383	PDS000180184.1	2021	Egypt	PRJNA1071125
GCA 032720515	PDT001927068.1	Klebsiella_383	PDS000085592.11	2020	Italy	PRJNA1026096
GCA 947572755	PDT001769582.1	Klebsiella_383	PDS000085592.11	2022	Germany	PRJEB58018
GCA 047426685	PDT002605982.1	Klebsiella_383	PDS000085592.11	2019	Italy	PRJNA1026096
GCA 025889055	PDT001469194.1	Klebsiella_383	PDS000085592.11	2019	Italy	PRJNA787449
GCA 021971055	PDT001019316.3	Klebsiella_383	PDS000085592.11	2021	USA	PRJNA288601
GCA 027908495	PDT001551547.1	Klebsiella_383	PDS000085592.11	2020	Egypt	PRJNA825651
GCA 043905145	**HBH_3731_19**	Klebsiella_383	PDS000085592.11	2019	Tunisia	PRJNA1160997
GCA 043905505	**HBH_990_22**	Klebsiella_383	PDS000085592.11	2022	Tunisia	PRJNA1160997
GCA 043905385	**HBH_211_21**	Klebsiella_383	PDS000085592.11	2021	Tunisia	PRJNA1160997
GCA 043905345	**HBH_1362_21**	Klebsiella_383	PDS000085592.11	2021	Tunisia	PRJNA1160997
GCA 043905355	**HBH_3959_20**	Klebsiella_383	PDS000085592.11	2020	Tunisia	PRJNA1160997
GCA 043905325	**HBH_3788_20**	Klebsiella_383	PDS000085592.11	2020	Tunisia	PRJNA1160997
GCA 043905185	**HBH_1789_20**	Klebsiella_383	PDS000098809.4	2020	Tunisia	PRJNA1160997
GCA 021898295	PDT001172748.1	Klebsiella_383	PDS000098809.4	2020	USA	PRJNA288601
GCA 021898135	PDT001174156.3	Klebsiella_383	PDS000098809.4	2020	USA	PRJNA288601
GCA 035082745	PDT002045985.1	Klebsiella_383		2023	USA	PRJNA288601
GCA 043904905	**HBH_487_19**	Klebsiella_383	PDS000085592.11	2019	Tunisia	PRJNA1160997
GCA 043905405	**HBH_3269_21**	Klebsiella_383		2021	Tunisia	PRJNA1160997
GCA 043904845	**HBH_1312_19**	Klebsiella_383	PDS000085592.11	2019	Tunisia	PRJNA1160997
GCA 043905605	**HBH_1879_22**	Klebsiella_383		2022	Tunisia	PRJNA1160997
GCA 032721505	PDT001927025.1	Klebsiella_383	PDS000217883.1	2019	Italy	PRJNA1026096
GCA 047501205	PDT002615048.1	Klebsiella_383	PDS000217883.1	2021	Tunisia	PRJNA1151248
GCA 047501255	PDT002615051.1	Klebsiella_383	PDS000217883.1	2023	Tunisia	PRJNA1151248
GCA 047501245	PDT002615049.1	Klebsiella_383	PDS000217883.1	2023	Tunisia	PRJNA1151248
GCA 032721475	PDT001927026.1	Klebsiella_383	PDS000085592.11	2019	Italy	PRJNA1026096
GCA 025892815	PDT001469195.1	Klebsiella_383	PDS000085592.11	2019	Italy	PRJNA787449
GCA 035017125	PDT002045096.1	Klebsiella_383		2022	Libya	PRJNA998204
GCA 035017085	PDT002045094.1	Klebsiella_383	PDS000171211.1	2022	Libya	PRJNA998204
GCA 035016965	PDT002045089.1	Klebsiella_383	PDS000171211.1	2022	Libya	PRJNA998204
GCA 039516715	PDT002152585.1	Klebsiella_383		2024	USA	PRJNA288601
GCA 964277065	PDT002423785.1	Klebsiella_383	PDS000192050.8	2024	Slovakia	PRJEB72521
GCA 964277015	PDT002423787.1	Klebsiella_383	PDS000192050.8	2024	Slovakia	PRJEB72521
GCA 964339425	PDT002468759.1	Klebsiella_383	PDS000192050.8	2024	Slovakia	PRJEB72521
GCA 964338835	PDT002461347.1	Klebsiella_383	PDS000192050.8	2024	Slovakia	PRJEB72521
GCA 964338735	PDT002462501.1	Klebsiella_383	PDS000192050.8	2024	Slovakia	PRJEB72521
GCA 964339405	PDT002468761.1	Klebsiella_383	PDS000192050.8	2024	Slovakia	PRJEB72521
GCA 964339365	PDT002468763.1	Klebsiella_383	PDS000192050.8	2024	Slovakia	PRJEB72521
GCA 964339305	PDT002468760.1	Klebsiella_383	PDS000192050.8	2024	Slovakia	PRJEB72521
GCA 964417265	PDT002518810.1	Klebsiella_383	PDS000192050.8	2024	Slovakia	PRJEB72521
GCA 964276885	PDT002423793.1	Klebsiella_383	PDS000192050.8	2024	Slovakia	PRJEB72521
GCA 964339465	PDT002468764.1	Klebsiella_383	PDS000192050.8	2024	Slovakia	PRJEB72521
GCA 964417235	PDT002518801.1	Klebsiella_383	PDS000192050.8	2024	Slovakia	PRJEB72521
GCA 964417105	PDT002518807.1	Klebsiella_383	PDS000192050.8	2024	Slovakia	PRJEB72521

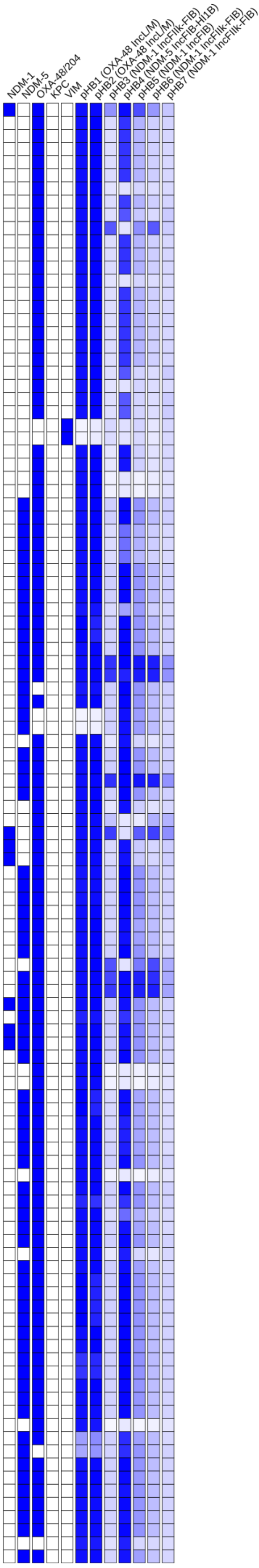

Supplement: Data S1B — Phylogenetic trees (14–17) generated in this study. [file aac.00142-26-s0002.pdf]
