# Supplementary material for: Leveraging the global genomic epidemiology of carbapenemase-producing Klebsiella pneumoniae to inform infection prevention in Tunisian hospitals
Source: Antimicrob Agents Chemother. 2026 May 6;70(6):e00142-26. doi: 10.1128/aac.00142-26 (PMC13231914; doi:10.1128/aac.00142-26)

Tree 28: GCA_043904865

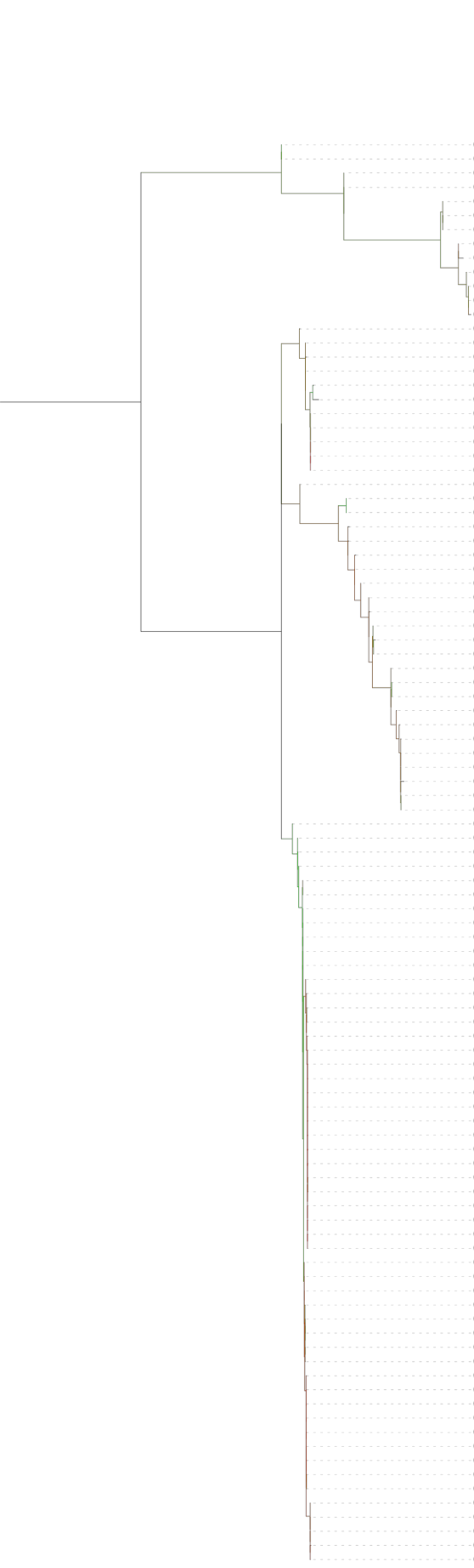

	Isolate	MLST	SNP Cluster	Collection Year	Country	BioProject
GCA 027615765	PDT001558318.1	Klebsiella_340	PDS000083373.14	2006	Bangladesh	PRJNA845975
GCA 027615465	PDT001558345.1	Klebsiella_340	PDS000083373.14	2007	Bangladesh	PRJNA845975
GCA 900505565	PDT000370452.1	Klebsiella_340	PDS000036321.13	2013	Serbia	PRJEB10018
GCA 900505585	PDT000370454.1	Klebsiella_340	PDS000036321.13	2013	Serbia	PRJEB10018
GCA 900505405	PDT000370438.1	Klebsiella_340	PDS000036321.13	2014	Serbia	PRJEB10018
GCA 900505465	PDT000370444.1	Klebsiella_340	PDS000036321.13	2014	Serbia	PRJEB10018
GCA 900502515	PDT000370030.1	Klebsiella_340	PDS000036321.13	2013	Serbia	PRJEB10018
GCA 900505455	PDT000370442.1	Klebsiella_340	PDS000036321.13	2014	Serbia	PRJEB10018
GCA 900507085	PDT000370461.1	Klebsiella_340	PDS000036321.13	2013	Serbia	PRJEB10018
GCA 900505115	PDT000370394.1	Klebsiella_340	PDS000036321.13	2014	Serbia	PRJEB10018
GCA 900505105	PDT000370395.1	Klebsiella_340	PDS000036321.13	2013	Serbia	PRJEB10018
GCA 900503495	PDT000370420.1	Klebsiella_340	PDS000036321.13	2013	Serbia	PRJEB10018
GCA 902155965	PDT000663175.1	Klebsiella_340	PDS000036321.13	2014	Serbia	PRJEB33308
GCA 022297255	PDT000423562.1	Klebsiella_340	PDS000083373.14	nan	NA	PRJEB17615
GCA 022296975	PDT000423578.1	Klebsiella_340	PDS000083373.14	nan	NA	PRJEB17615
GCA 022297095	PDT000423571.1	Klebsiella_340	PDS000083373.14	nan	NA	PRJEB17615
GCA 022298895	PDT000423473.1	Klebsiella_340	PDS000083373.14	nan	NA	PRJEB17615
GCA 022244755	PDT000441138.1	Klebsiella_340	PDS000017086.10	2012	Nepal	PRJEB1800
GCA 027827735	PDT001558013.1	Klebsiella_-	PDS000083373.14	2010	Bangladesh	PRJNA845975
GCA 022275725	PDT000432627.1	Klebsiella_340	PDS000083373.14	2008	Ireland	PRJNA396729
GCA 022275665	PDT000432629.1	Klebsiella_340	PDS000083373.14	2008	Ireland	PRJNA396729
GCA 022275605	PDT000432633.1	Klebsiella_340	PDS000083373.14	2008	Ireland	PRJNA396729
GCA 022275645	PDT000432632.1	Klebsiella_340	PDS000083373.14	2008	Ireland	PRJNA396729
GCA 022275705	PDT000432628.1	Klebsiella_340	PDS000083373.14	2008	Ireland	PRJNA396729
GCA 047058785	PDT002566001.1	Klebsiella_340		2018	Spain	PRJNA1150980
GCA 021922125	PDT001092057.1	Klebsiella_340	PDS000083373.14	2013	Norway	PRJEB27256
GCA 041860575	PDT002369885.2	Klebsiella_340	PDS000083373.14	2013	Norway	PRJNA1098507
GCA 043904865	**HBH_866_10**	Klebsiella_340		2010	Tunisia	PRJNA1160997
GCA 032153575	PDT001902908.1	Klebsiella_340	PDS000083373.14	2017	Norway	PRJEB48268
GCA 022287605	PDT000427672.1	Klebsiella_340	PDS000036321.13	nan	NA	PRJEB10018
GCA 900505545	PDT000370451.1	Klebsiella_340	PDS000036321.13	2013	Serbia	PRJEB10018
GCA 021921885	PDT001092076.1	Klebsiella_340	PDS000036321.13	2014	Norway	PRJEB27256
GCA 956480655	PDT001865600.1	Klebsiella_340	PDS000036321.13	2018	Serbia	PRJEB63349
GCA 956480695	PDT001865601.1	Klebsiella_340	PDS000036321.13	2018	Serbia	PRJEB63349
GCA 022234485	PDT000442756.1	Klebsiella_340	PDS000036321.13	2013	Serbia	PRJNA449293
GCA 900503575	PDT000370443.1	Klebsiella_340	PDS000036321.13	2014	Serbia	PRJEB10018
GCA 041508645	PDT002341334.1	Klebsiella_340	PDS000036321.13	2019	USA	PRJNA288601
GCA 022016735	PDT000948692.1	Klebsiella_340		2019	Qatar	PRJNA667192
GCA 027616425	PDT001558233.1	Klebsiella_340	PDS000130841.1	2021	Bangladesh	PRJNA845975
GCA 027617505	PDT001558154.1	Klebsiella_340	PDS000130841.1	2021	Bangladesh	PRJNA845975
GCA 047276595	PDT002596157.1	Klebsiella_340	PDS000083373.14	2018	Australia	PRJNA674961
GCA 022298635	PDT000423487.1	Klebsiella_340	PDS000083373.14	nan	NA	PRJEB17615
GCA 022245285	PDT000441101.1	Klebsiella_340		2012	Nepal	PRJEB1800
GCA 045010645	PDT002049725.1	Klebsiella_340	PDS000083373.14	2019	Bangladesh	PRJNA962904
GCA 027615785	PDT001558310.1	Klebsiella_340	PDS000083373.14	2007	Bangladesh	PRJNA845975
GCA 021982955	PDT001003396.1	Klebsiella_340	PDS000083373.14	2016	Thailand	PRJDB5929
GCA 027884925	PDT001557976.1	Klebsiella_340	PDS000130869.1	2013	Bangladesh	PRJNA845975
GCA 027828495	PDT001557977.1	Klebsiella_340	PDS000130869.1	2015	Bangladesh	PRJNA845975
GCA 022302195	PDT000193289.2	Klebsiella_340	PDS000083373.14	2008	Thailand	PRJNA252957
GCA 022302155	PDT000193290.2	Klebsiella_340	PDS000083373.14	2008	Thailand	PRJNA252957
GCA 027828055	PDT001557998.1	Klebsiella_340	PDS000083373.14	2007	Bangladesh	PRJNA845975
GCA 022302215	PDT000193287.2	Klebsiella_340	PDS000083373.14	2008	Thailand	PRJNA252957
GCA 022008995	PDT000986849.1	Klebsiella_340	PDS000083373.14	2015	Thailand	PRJDB5929
GCA 022027375	PDT000925078.1	Klebsiella_340	PDS000083373.14	2016	Singapore	PRJNA674942
GCA 022002555	PDT000986958.1	Klebsiella_340	PDS000083373.14	2015	Thailand	PRJDB5929
GCA 021981655	PDT001008094.1	Klebsiella_340	PDS000083373.14	2017	Thailand	PRJDB5929
GCA 021982775	PDT001007733.1	Klebsiella_340	PDS000083373.14	2017	Thailand	PRJDB5929
GCA 021986135	PDT001003173.1	Klebsiella_340	PDS000083373.14	2017	Thailand	PRJDB5929
GCA 022005075	PDT000986950.1	Klebsiella_340	PDS000083373.14	2015	Thailand	PRJDB5929
GCA 021988075	PDT001003051.1	Klebsiella_340	PDS000083373.14	2016	Thailand	PRJDB5929
GCA 022005085	PDT000986954.1	Klebsiella_340	PDS000083373.14	2015	Thailand	PRJDB5929
GCA 021983895	PDT001003349.1	Klebsiella_340	PDS000083373.14	2017	Thailand	PRJDB5929
GCA 021985815	PDT001003194.1	Klebsiella_340	PDS000083373.14	2017	Thailand	PRJDB5929
GCA 021987755	PDT001003070.1	Klebsiella_340	PDS000083373.14	2017	Thailand	PRJDB5929
GCA 021981755	PDT001008086.1	Klebsiella_340	PDS000083373.14	2016	Thailand	PRJDB5929
GCA 021984375	PDT001003311.1	Klebsiella_340	PDS000083373.14	2017	Thailand	PRJDB5929
GCA 021984755	PDT001003273.1	Klebsiella_340	PDS000083373.14	2016	Thailand	PRJDB5929
GCA 021984995	PDT001003252.1	Klebsiella_340	PDS000083373.14	2016	Thailand	PRJDB5929
GCA 021988055	PDT001003053.1	Klebsiella_340	PDS000083373.14	2016	Thailand	PRJDB5929
GCA 021984315	PDT001003314.1	Klebsiella_340	PDS000083373.14	2017	Thailand	PRJDB5929
GCA 021984975	PDT001003253.1	Klebsiella_340	PDS000083373.14	2016	Thailand	PRJDB5929
GCA 022002725	PDT000986956.1	Klebsiella_340	PDS000083373.14	2015	Thailand	PRJDB5929
GCA 021982995	PDT001003394.1	Klebsiella_340	PDS000083373.14	2016	Thailand	PRJDB5929
GCA 021987215	PDT001003098.1	Klebsiella_340	PDS000083373.14	2016	Thailand	PRJDB5929
GCA 021986215	PDT001003169.1	Klebsiella_340	PDS000083373.14	2017	Thailand	PRJDB5929
GCA 022005575	PDT000986937.1	Klebsiella_340	PDS000083373.14	2015	Thailand	PRJDB5929
GCA 021987135	PDT001003104.1	Klebsiella_340	PDS000083373.14	2017	Thailand	PRJDB5929
GCA 022005735	PDT000986933.1	Klebsiella_340	PDS000083373.14	2015	Thailand	PRJDB5929
GCA 021982635	PDT001008025.1	Klebsiella_340	PDS000083373.14	2016	Thailand	PRJDB5929
GCA 022001335	PDT000987001.1	Klebsiella_340	PDS000083373.14	2016	Thailand	PRJDB5929
GCA 022005825	PDT000986932.1	Klebsiella_340	PDS000083373.14	2015	Thailand	PRJDB5929
GCA 022005535	PDT000986940.1	Klebsiella_340	PDS000083373.14	2015	Thailand	PRJDB5929
GCA 022005955	PDT000986929.1	Klebsiella_340	PDS000083373.14	2015	Thailand	PRJDB5929
GCA 021984275	PDT001003316.1	Klebsiella_340	PDS000083373.14	2017	Thailand	PRJDB5929
GCA 022001365	PDT000987003.1	Klebsiella_340	PDS000083373.14	2016	Thailand	PRJDB5929
GCA 022005555	PDT000986935.1	Klebsiella_340	PDS000083373.14	2015	Thailand	PRJDB5929
GCA 021986745	PDT001003136.1	Klebsiella_340	PDS000083373.14	2016	Thailand	PRJDB5929
GCA 021981295	PDT001008138.1	Klebsiella_340	PDS000083373.14	2017	Thailand	PRJDB5929
GCA 021985035	PDT001003251.1	Klebsiella_340	PDS000083373.14	2016	Thailand	PRJDB5929
GCA 021982835	PDT001003422.1	Klebsiella_340	PDS000083373.14	2017	Thailand	PRJDB5929
GCA 022000985	PDT000987027.1	Klebsiella_340	PDS000083373.14	2016	Thailand	PRJDB5929
GCA 021984355	PDT001003312.1	Klebsiella_340	PDS000083373.14	2017	Thailand	PRJDB5929
GCA 021987295	PDT001003095.1	Klebsiella_340	PDS000083373.14	2016	Thailand	PRJDB5929
GCA 021986015	PDT001003179.1	Klebsiella_340	PDS000083373.14	2017	Thailand	PRJDB5929
GCA 021984255	PDT001003315.1	Klebsiella_340	PDS000083373.14	2017	Thailand	PRJDB5929
GCA 021987795	PDT001003069.1	Klebsiella_340	PDS000083373.14	2017	Thailand	PRJDB5929
GCA 021981775	PDT001008085.1	Klebsiella_340	PDS000083373.14	2016	Thailand	PRJDB5929
GCA 021987115	PDT001003106.1	Klebsiella_340	PDS000083373.14	2017	Thailand	PRJDB5929
GCA 022005135	PDT000986951.1	Klebsiella_340	PDS000083373.14	2015	Thailand	PRJDB5929
GCA 021985075	PDT001003249.1	Klebsiella_340	PDS000083373.14	2016	Thailand	PRJDB5929
GCA 022000655	PDT000987028.1	Klebsiella_340	PDS000083373.14	2016	Thailand	PRJDB5929

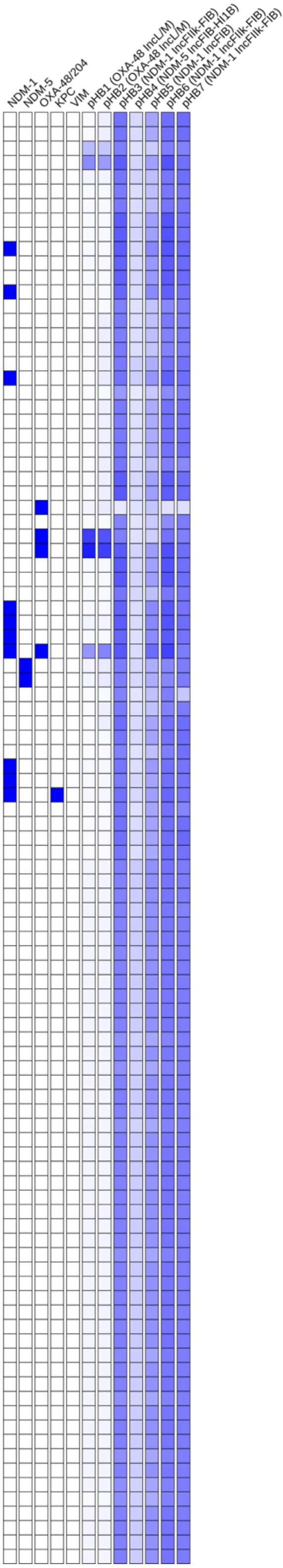

Tree 29: GCA_043904885

Tree scale: 0.01

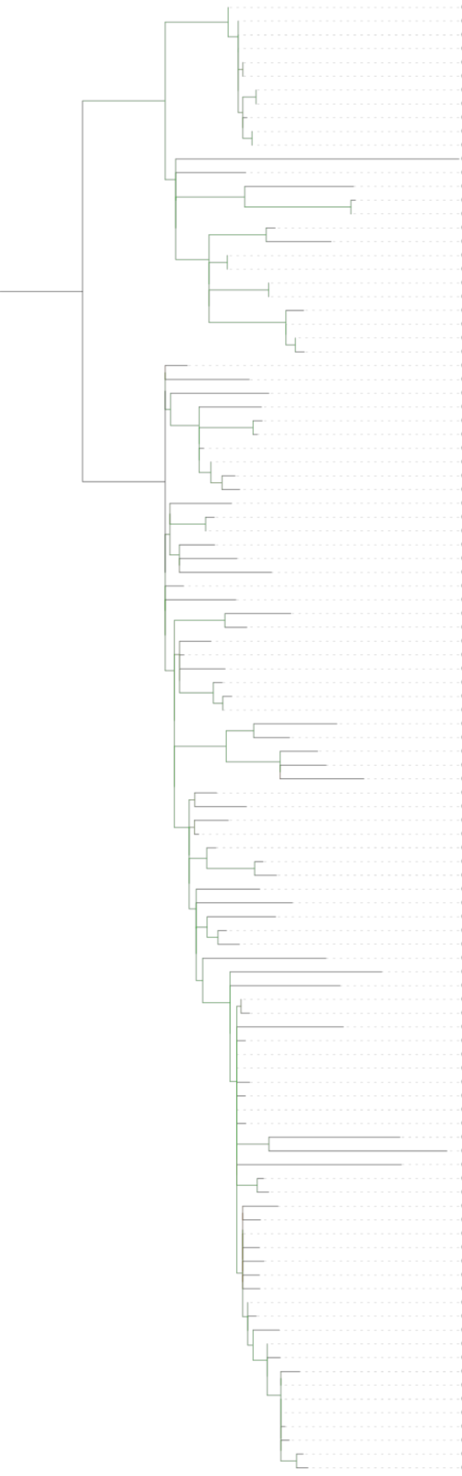

Accession	Isolate	MLST	SNP Cluster	Collection Year	Country	BioProject
GCA 022265795	PDT000435277.1	Klebsiella_147	PDS000071979.8	2014	Singapore	PRJNA342893
GCA 025113045	PDT001411016.1	Klebsiella_147	PDS000071979.8	2014	Singapore	PRJNA577535
GCA 022265755	PDT000435279.1	Klebsiella_147	PDS000071979.8	2014	Singapore	PRJNA342893
GCA 023457995	PDT001307874.1	Klebsiella_147	PDS000071979.8	nan	Singapore	PRJNA765801
GCA 022265955	PDT000435263.1	Klebsiella_147	PDS000071979.8	2014	Singapore	PRJNA342893
GCA 001855315	PDT000155119.1	Klebsiella_147	PDS000071979.8	2014	Singapore	PRJNA286828
GCA 023367875	PDT001308138.1	Klebsiella_147	PDS000071979.8	nan	Singapore	PRJNA765801
GCA 022265875	PDT000435270.1	Klebsiella_147	PDS000071979.8	2014	Singapore	PRJNA342893
GCA 023366555	PDT001307947.1	Klebsiella_147	PDS000071979.8	nan	Singapore	PRJNA765801
GCA 029494475	PDT001672736.1	Klebsiella_147	PDS000071979.8	nan	Singapore	PRJNA757551
GCA 022265575	PDT000435328.1	Klebsiella_147	PDS000071979.8	2015	Singapore	PRJNA342893
GCA 022159145	PDT001243662.1	Klebsiella_147	PDS000104516.1	2016	United Kingdom	PRJNA788733
GCA 000817855	PDT000048072.1	Klebsiella_147		2012	Australia	PRJNA270953
GCA 047277415	PDT002596061.1	Klebsiella_147	PDS000092779.3	2017	Australia	PRJNA674961
GCA 025112985	PDT001411347.1	Klebsiella_147	PDS000026421.12	2014	Singapore	PRJNA577535
GCA 025077115	PDT001411348.1	Klebsiella_147	PDS000026421.12	2014	Singapore	PRJNA577535
GCA 900511365	PDT000369134.1	Klebsiella_147	PDS000009779.14	2014	Poland	PRJEB10018
GCA 900511505	PDT000369153.1	Klebsiella_147	PDS000009779.14	2013	Poland	PRJEB10018
GCA 034651715	PDT002040257.1	Klebsiella_147	PDS000009779.14	nan	NA	PRJNA1017778
GCA 022320785	PDT001251452.1	Klebsiella_147	PDS000009779.14	nan	United Kingdom	PRJNA787440
GCA 003181055	PDT000323199.1	Klebsiella_147	PDS000045262.1	nan	Sweden	PRJNA472712
GCA 003856595	PDT000414016.1	Klebsiella_147	PDS000045262.1	2009	Sweden	PRJNA413669
GCA 022295635	PDT000423657.1	Klebsiella_147	PDS000009779.14	nan	NA	PRJEB17615
GCA 022295855	PDT000423646.1	Klebsiella_147	PDS000009779.14	nan	NA	PRJEB17615
GCA 022298135	PDT000423515.1	Klebsiella_147	PDS000009779.14	nan	NA	PRJEB17615
GCA 022298095	PDT000423521.1	Klebsiella_147	PDS000009779.14	nan	NA	PRJEB17615
GCA 022122365	PDT001244529.1	Klebsiella_147	PDS000045135.24	2015	United Kingdom	PRJNA788733
GCA 022502665	PDT001261546.1	Klebsiella_147		2019	Spain	PRJNA800400
GCA 022132725	PDT001244317.1	Klebsiella_147	PDS000045135.24	2016	United Kingdom	PRJNA788733
GCA 021934075	PDT001087300.1	Klebsiella_147	PDS000045135.24	2018	Australia	PRJNA529744
GCA 947660055	PDT001594206.1	Klebsiella_147	PDS000045135.24	2016	Greece	PRJEB58216
GCA 028090455	PDT001585570.1	Klebsiella_147	PDS000045135.24	2016	Greece	PRJEB58216
GCA 028090935	PDT001585542.1	Klebsiella_147	PDS000045135.24	2014	Greece	PRJEB58216
GCA 947660715	PDT001594132.1	Klebsiella_147	PDS000045135.24	2014	Greece	PRJEB58216
GCA 956471375	PDT001861454.1	Klebsiella_147	PDS000045135.24	2018	Greece	PRJEB63349
GCA 956479645	PDT001861601.1	Klebsiella_147	PDS000045135.24	2017	Greece	PRJEB63349
GCA 022985945	PDT001286496.1	Klebsiella_147	PDS000045135.24	2017	Germany	PRJNA789142
GCA 024371215	PDT001363427.1	Klebsiella_147	PDS000045135.24	2017	Germany	PRJNA845217
GCA 024350865	PDT001363428.1	Klebsiella_147	PDS000045135.24	2017	Germany	PRJNA845217
GCA 004803035	PDT000495879.1	Klebsiella_147	PDS000045135.24	nan	Egypt	PRJNA508509
GCA 043905685	PDT002391412.1	Klebsiella_147	PDS000045135.24	2019	Netherlands	PRJNA1143178
GCA 947575175	PDT001769426.1	Klebsiella_147	PDS000045135.24	2022	Germany	PRJEB58018
GCA 043861575	**HBH_1449_15**	Klebsiella_147	PDS000045135.24	2015	Tunisia	PRJNA1160997
GCA 014877695	PDT000860271.1	Klebsiella_147	PDS000045135.24	2019	Poland	PRJNA666795
GCA 021937495	PDT001078572.1	Klebsiella_147	PDS000045135.24	2016/2018	France	PRJNA738476
GCA 024609285	PDT001385474.1	Klebsiella_147	PDS000045135.24	2016	Tunisia	PRJNA841857
GCA 009856915	PDT000663244.1	Klebsiella_147	PDS000045135.24	2016	France	PRJNA596440
GCA 022104755	PDT001244567.1	Klebsiella_147	PDS000045135.24	2014	United Kingdom	PRJNA788733
GCA 043863475	**HBH_1966_18**	Klebsiella_147	PDS000045135.24	2018	Tunisia	PRJNA1160997
GCA 014877635	PDT000860270.1	Klebsiella_147	PDS000045135.24	2018	Poland	PRJNA666795
GCA 014877845	PDT000860279.1	Klebsiella_147	PDS000045135.24	2019	Poland	PRJNA666795
GCA 014877855	PDT000860278.1	Klebsiella_147	PDS000045135.24	2018	Poland	PRJNA666795
GCA 956479455	PDT001861756.1	Klebsiella_147	PDS000045135.24	2017	Romania	PRJEB63349
GCA 022100115	PDT001243969.1	Klebsiella_147	PDS000045135.24	2014	United Kingdom	PRJNA788733
GCA 022186125	PDT000716604.1	Klebsiella_147	PDS000045135.24	2018	France	PRJEB35890
GCA 022185705	PDT000716601.1	Klebsiella_147	PDS000045135.24	2018	France	PRJEB35890
GCA 022185495	PDT000716626.1	Klebsiella_147	PDS000080114.2	2019	Netherlands	PRJEB35890
GCA 003123945	PDT000318267.1	Klebsiella_147	PDS000045135.24	2017	France	PRJNA436343
GCA 022185605	PDT000716612.1	Klebsiella_147	PDS000045135.24	2019	France	PRJEB35890
GCA 043860395	**HBH_3187_14**	Klebsiella_147	PDS000045135.24	2014	Tunisia	PRJNA1160997
GCA 021806665	PDT001227331.1	Klebsiella_147	PDS000045135.24	2014	United Kingdom	PRJNA788733
GCA 021808745	PDT001227264.1	Klebsiella_147	PDS000045135.24	2014	United Kingdom	PRJNA788733
GCA 043904885	**HBH_2381_19**	Klebsiella_147	PDS000045135.24	2019	Tunisia	PRJNA1160997
GCA 032721275	PDT001927037.1	Klebsiella_147	PDS000045135.24	2019	Italy	PRJNA1026096
GCA 025041995	PDT001386176.1	Klebsiella_147	PDS000045135.24	2019	Canada	PRJNA855907
GCA 022186345	PDT000716553.1	Klebsiella_147		2015	United Kingdom	PRJEB35890
GCA 021840155	PDT001216465.1	Klebsiella_147	PDS000045135.24	2019	Switzerland	PRJNA744003
GCA 043863085	**HBH_3373_15**	Klebsiella_147	PDS000045135.24	2015	Tunisia	PRJNA1160997
GCA 043861665	**HBH_3234_15**	Klebsiella_147	PDS000045135.24	2015	Tunisia	PRJNA1160997
GCA 014877895	PDT000860280.1	Klebsiella_147		2015	Poland	PRJNA666795
GCA 014877815	PDT000860277.1	Klebsiella_147		2015	Poland	PRJNA666795
GCA 014880015	PDT000863718.1	Klebsiella_147		2015	Poland	PRJNA666795
GCA 014876995	PDT000860252.1	Klebsiella_147	PDS000045135.24	2017	Poland	PRJNA666795
GCA 014879995	PDT000863720.1	Klebsiella_147	PDS000045135.24	2017	Poland	PRJNA666795
GCA 014877435	PDT000860266.1	Klebsiella_147	PDS000045135.24	2016	Poland	PRJNA666795
GCA 014876925	PDT000860248.1	Klebsiella_147	PDS000045135.24	2015	Poland	PRJNA666795
GCA 014876815	PDT000860244.1	Klebsiella_147	PDS000045135.24	2015	Poland	PRJNA666795
GCA 014877105	PDT000860253.1	Klebsiella_147	PDS000045135.24	2017	Poland	PRJNA666795
GCA 014876895	PDT000860245.1	Klebsiella_147	PDS000045135.24	2016	Poland	PRJNA666795
GCA 014876795	PDT000860243.1	Klebsiella_147	PDS000045135.24	2016	Poland	PRJNA666795
GCA 014877025	PDT000860250.1	Klebsiella_147	PDS000045135.24	2015	Poland	PRJNA666795
GCA 014877935	PDT000860281.1	Klebsiella_147	PDS000045135.24	2015	Poland	PRJNA666795
GCA 014877325	PDT000860263.1	Klebsiella_147		2016	Poland	PRJNA666795
GCA 014877395	PDT000860265.1	Klebsiella_147		2016	Poland	PRJNA666795
GCA 014877955	PDT000860282.1	Klebsiella_147		2016	Poland	PRJNA666795
GCA 014877755	PDT000860274.1	Klebsiella_147	PDS000045135.24	2019	Poland	PRJNA666795
GCA 014880035	PDT000863721.1	Klebsiella_147	PDS000045135.24	2018	Poland	PRJNA666795
GCA 014876905	PDT000860247.1	Klebsiella_147	PDS000045135.24	2017	Poland	PRJNA666795
GCA 014877195	PDT000860257.1	Klebsiella_147	PDS000045135.24	2017	Poland	PRJNA666795
GCA 014877295	PDT000860261.1	Klebsiella_147	PDS000045135.24	2017	Poland	PRJNA666795
GCA 014877205	PDT000860258.1	Klebsiella_147	PDS000045135.24	2018	Poland	PRJNA666795
GCA 014877125	PDT000860255.1	Klebsiella_147	PDS000045135.24	2018	Poland	PRJNA666795
GCA 014877265	PDT000860260.1	Klebsiella_147	PDS000045135.24	2017	Poland	PRJNA666795
GCA 014877115	PDT000860254.1	Klebsiella_147	PDS000045135.24	2017	Poland	PRJNA666795
GCA 014877215	PDT000860259.1	Klebsiella_147	PDS000045135.24	2017	Poland	PRJNA666795
GCA 014877015	PDT000860251.1	Klebsiella_147	PDS000045135.24	2017	Poland	PRJNA666795
GCA 014876935	PDT000860249.1	Klebsiella_147	PDS000045135.24	2017	Poland	PRJNA666795
GCA 014877315	PDT000860262.1	Klebsiella_147	PDS000045135.24	2017	Poland	PRJNA666795
GCA 014880155	PDT000863723.1	Klebsiella_147	PDS000045135.24	2019	Poland	PRJNA666795
GCA 014878005	PDT000860284.1	Klebsiella_147	PDS000045135.24	2019	Poland	PRJNA666795
GCA 014880095	PDT000863722.1	Klebsiella_147	PDS000045135.24	2017	Poland	PRJNA666795
GCA 014877745	PDT000860275.1	Klebsiella_147	PDS000045135.24	2017	Poland	PRJNA666795
GCA 014877715	PDT000860272.1	Klebsiella_147	PDS000045135.24	2017	Poland	PRJNA666795
GCA 014877645	PDT000860269.1	Klebsiella_147	PDS000045135.24	2017	Poland	PRJNA666795
GCA 014877625	PDT000860268.1	Klebsiella_147	PDS000045135.24	2017	Poland	PRJNA666795
GCA 014877995	PDT000860283.1	Klebsiella_147	PDS000045135.24	2017	Poland	PRJNA666795
GCA 014877725	PDT000860273.1	Klebsiella_147	PDS000045135.24	2017	Poland	PRJNA666795

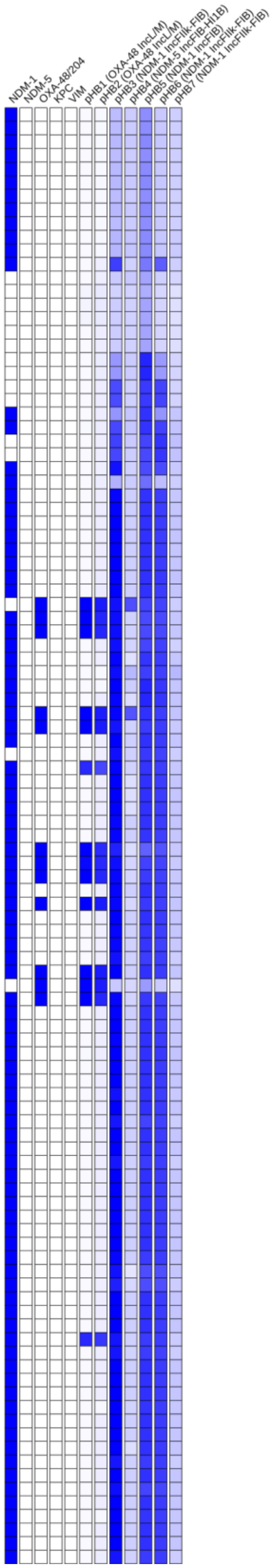

Tree 30: GCA_043905265

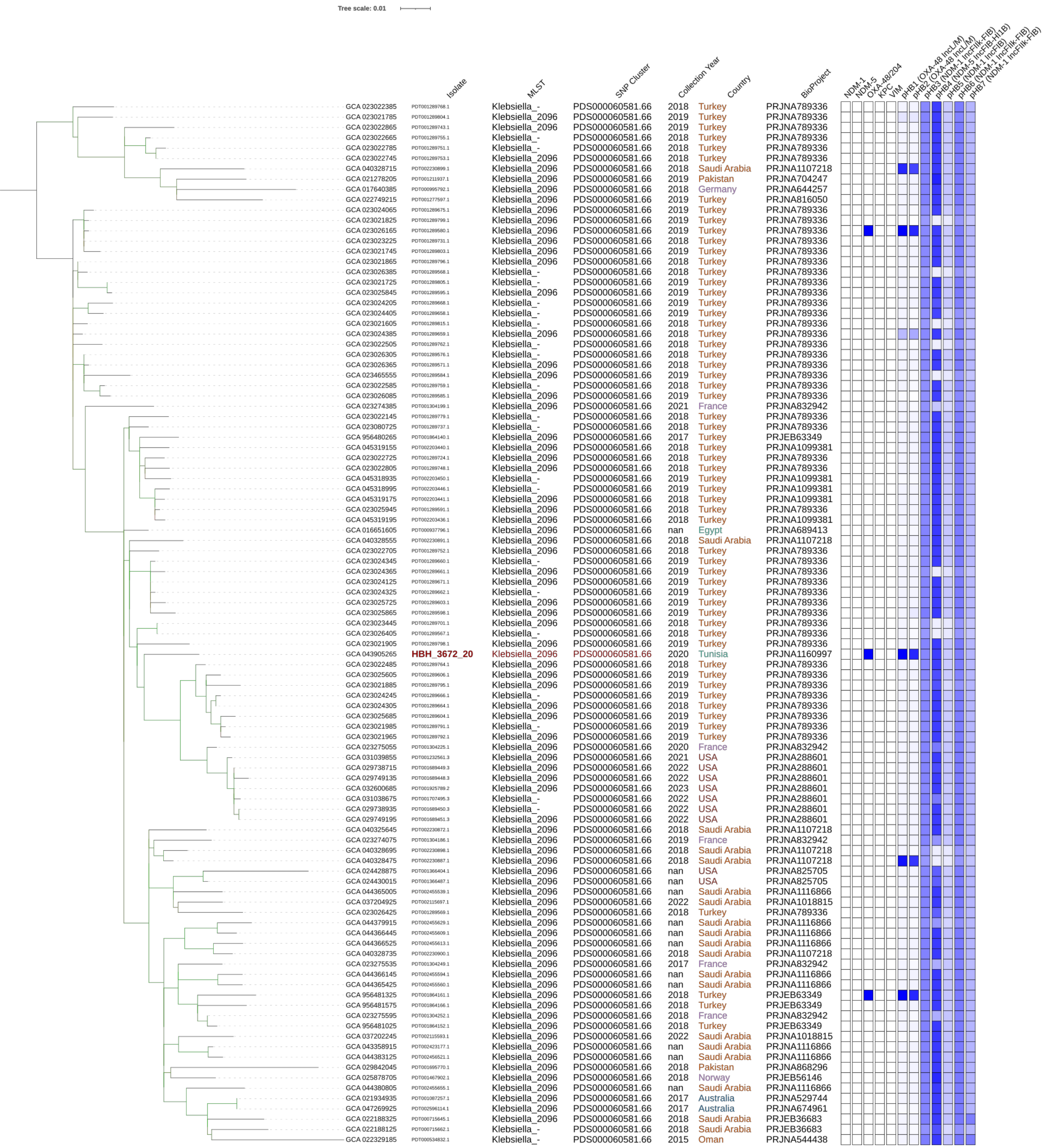

Tree 31: GCA_043905305

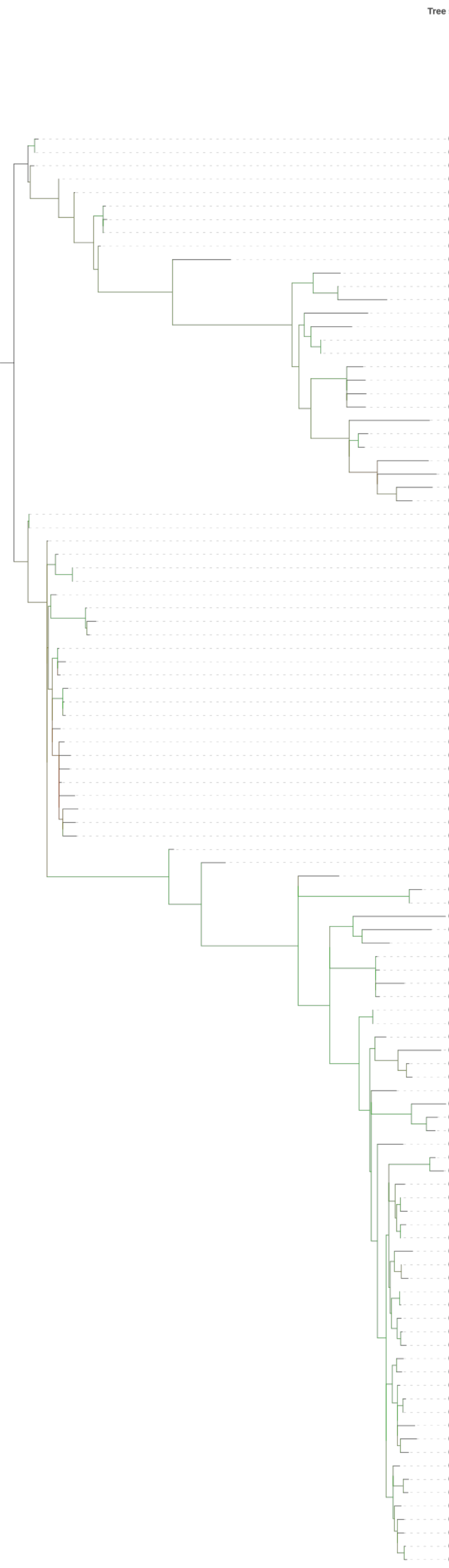

	Isolate	MLST	SNP Cluster	Collection Year	Country	BioProject
GCA 014285715	PDT000822630.1	Klebsiella_383	PDS000070357.5	2014	China	PRJNA657553
GCA 014285775	PDT000822634.1	Klebsiella_383	PDS000070357.5	2014	China	PRJNA657553
GCA 046465065	PDT002556478.1	Klebsiella_383	PDS000070357.5	nan	NA	PRJNA786399
GCA 046466655	PDT002556557.1	Klebsiella_383	PDS000070357.5	nan	NA	PRJNA786399
GCA 046466735	PDT002556561.1	Klebsiella_383	PDS000070357.5	nan	NA	PRJNA786399
GCA 046466415	PDT002556545.1	Klebsiella_383	PDS000070357.5	nan	NA	PRJNA786399
GCA 043109335	PDT002420644.1	Klebsiella_383	PDS000070357.5	2014	China	PRJNA1170063
GCA 046466035	PDT002556526.1	Klebsiella_383	PDS000070357.5	nan	NA	PRJNA786399
GCA 017310305	PDT000984751.1	Klebsiella_383	PDS000070357.5	2013	China	PRJNA648010
GCA 022104735	PDT001244769.1	Klebsiella_383		2016	United Kingdom	PRJNA788733
GCA 004803085	PDT000495882.1	Klebsiella_383		nan	Egypt	PRJNA508509
GCA 022186025	PDT000716574.1	Klebsiella_383	PDS000060640.1	2019	Germany	PRJEB35890
GCA 022186005	PDT000716575.1	Klebsiella_383	PDS000060640.1	2019	Germany	PRJEB35890
GCA 022104535	PDT001243313.1	Klebsiella_383	PDS000106324.3	2016	United Kingdom	PRJNA788733
GCA 040201255	PDT002208472.1	Klebsiella_-		nan	Saudi Arabia	PRJNA1116866
GCA 022550215	PDT001263624.1	Klebsiella_383	PDS000106324.3	2019	Egypt	PRJNA720263
GCA 022550175	PDT001263622.1	Klebsiella_383	PDS000106324.3	2018	Egypt	PRJNA720263
GCA 043905145	**HBH_3731_19**	Klebsiella_383	PDS000085592.11	2019	Tunisia	PRJNA1160997
GCA 043904905	**HBH_487_19**	Klebsiella_383	PDS000085592.11	2019	Tunisia	PRJNA1160997
GCA 025892815	PDT001469195.1	Klebsiella_383	PDS000085592.11	2019	Italy	PRJNA787449
GCA 021971055	PDT001019316.3	Klebsiella_383	PDS000085592.11	2021	USA	PRJNA288601
GCA 037000505	PDT002098839.1	Klebsiella_383		2015	Egypt	PRJNA1079765
GCA 025144525	PDT001416171.1	Klebsiella_383	PDS000108392.5	2016	Qatar	PRJNA656934
GCA 024087515	PDT001350138.1	Klebsiella_383	PDS000108392.5	2016	Qatar	PRJNA656934
GCA 025037975	PDT001386067.1	Klebsiella_383		2019	Canada	PRJNA855907
GCA 922831335	PDT001211077.1	Klebsiella_383	PDS000085592.11	2019	United Kingdom	PRJEB48990
GCA 016651765	PDT000937804.1	Klebsiella_383	PDS000106324.3	nan	Egypt	PRJNA689413
GCA 023080795	PDT001289734.1	Klebsiella_383	PDS000108392.5	2018	Turkey	PRJNA789336
GCA 014285835	PDT000822636.1	Klebsiella_383	PDS000070357.5	2014	China	PRJNA657553
GCA 046465045	PDT002556479.1	Klebsiella_383	PDS000070357.5	nan	NA	PRJNA786399
GCA 014285675	PDT000822629.1	Klebsiella_383	PDS000070357.5	2013	China	PRJNA657553
GCA 014284855	PDT000822587.1	Klebsiella_383	PDS000070357.5	2015	China	PRJNA657553
GCA 014285545	PDT000822621.1	Klebsiella_383	PDS000070357.5	2015	China	PRJNA657553
GCA 014285615	PDT000822625.1	Klebsiella_383	PDS000070357.5	2015	China	PRJNA657553
GCA 014286025	PDT000822646.1	Klebsiella_383	PDS000070357.5	2013	China	PRJNA657553
GCA 037052525	PDT002102101.1	Klebsiella_383	PDS000176495.1	2013	Jordan	PRJNA1076682
GCA 037052245	PDT002102087.1	Klebsiella_383	PDS000176495.1	2015	Jordan	PRJNA1076682
GCA 037052285	PDT002102089.1	Klebsiella_383	PDS000176495.1	2013	Jordan	PRJNA1076682
GCA 014285735	PDT000822631.1	Klebsiella_383	PDS000070357.5	2014	China	PRJNA657553
GCA 014288455	PDT000822723.1	Klebsiella_383	PDS000070357.5	2014	China	PRJNA657553
GCA 046465235	PDT002556486.1	Klebsiella_383	PDS000070357.5	nan	NA	PRJNA786399
GCA 017311535	PDT000984759.1	Klebsiella_383	PDS000070357.5	2015	China	PRJNA648010
GCA 017311595	PDT000984760.1	Klebsiella_383	PDS000070357.5	2014	China	PRJNA648010
GCA 014285665	PDT000822628.1	Klebsiella_383	PDS000070357.5	2014	China	PRJNA657553
GCA 014285645	PDT000822627.1	Klebsiella_383	PDS000070357.5	2015	China	PRJNA657553
GCA 014289355	PDT000822768.1	Klebsiella_383	PDS000070357.5	2013	China	PRJNA657553
GCA 014285595	PDT000822624.1	Klebsiella_383	PDS000070357.5	2015	China	PRJNA657553
GCA 017311635	PDT000984762.1	Klebsiella_383	PDS000070357.5	2014	China	PRJNA648010
GCA 017311605	PDT000984761.1	Klebsiella_383	PDS000070357.5	2013	China	PRJNA648010
GCA 014288555	PDT000822728.1	Klebsiella_383	PDS000070357.5	2014	China	PRJNA657553
GCA 017311675	PDT000984764.1	Klebsiella_383	PDS000070357.5	2017	China	PRJNA648010
GCA 017311735	PDT000984767.1	Klebsiella_383	PDS000070357.5	2013	China	PRJNA648010
GCA 017311645	PDT000984763.1	Klebsiella_383	PDS000070357.5	2014	China	PRJNA648010
GCA 017311685	PDT000984765.1	Klebsiella_383	PDS000070357.5	2017	China	PRJNA648010
GCA 043949915	PDT002438925.1	Klebsiella_383	PDS000070357.5	2015	China	PRJNA1173368
GCA 021798805	PDT001227454.1	Klebsiella_383		2014	United Kingdom	PRJNA788733
GCA 019781185	PDT001118308.1	Klebsiella_-	PDS000202475.1	2019	Germany	PRJNA634442
GCA 043832345	PDT002430492.1	Klebsiella_-	PDS000202475.1	2019	Germany	PRJNA1070536
GCA 900513485	PDT000369524.1	Klebsiella_383		2013	Israel	PRJEB10018
GCA 916161735	PDT001141241.1	Klebsiella_383		2015	Netherlands	PRJEB41042
GCA 900504425	PDT000370281.1	Klebsiella_383		2014	Romania	PRJEB10018
GCA 043859635	**HBH_233_09**	Klebsiella_383	PDS000199419.2	2009	Tunisia	PRJNA1160997
GCA 043905305	**HBH_416_10**	Klebsiella_383	PDS000199419.2	2010	Tunisia	PRJNA1160997
GCA 043904745	**HBH_30_10**	Klebsiella_383	PDS000199419.2	2010	Tunisia	PRJNA1160997
GCA 043858235	**HBH_116_09**	Klebsiella_383	PDS000199419.2	2009	Tunisia	PRJNA1160997
GCA 028092175	PDT001585477.1	Klebsiella_383	PDS000133910.8	2007	Greece	PRJEB58216
GCA 947662285	PDT001590394.1	Klebsiella_383	PDS000133910.8	2007	Greece	PRJEB58216
GCA 022158485	PDT001243735.1	Klebsiella_383	PDS000133910.8	2014	United Kingdom	PRJNA788733
GCA 001611055	PDT000123366.1	Klebsiella_383		2011	Greece	PRJNA295651
GCA 022099655	PDT001243972.1	Klebsiella_383	PDS000133910.8	2014	United Kingdom	PRJNA788733
GCA 916161915	PDT001141253.1	Klebsiella_383	PDS000133910.8	2013	Netherlands	PRJEB41042
GCA 022268295	PDT000434786.1	Klebsiella_383		2012	Greece	PRJDB4948
GCA 900500925	PDT000369958.1	Klebsiella_383	PDS000007455.4	2014	Greece	PRJEB10018
GCA 001701935	PDT000144375.1	Klebsiella_383	PDS000007455.4	2012	Greece	PRJNA307517
GCA 001701985	PDT000144378.1	Klebsiella_383	PDS000007455.4	2013	Greece	PRJNA307517
GCA 916161705	PDT001141240.1	Klebsiella_383		2012	Netherlands	PRJEB41042
GCA 028090635	PDT001585554.1	Klebsiella_383	PDS000133910.8	2015	Greece	PRJEB58216
GCA 947660785	PDT001592146.1	Klebsiella_383	PDS000133910.8	2015	Greece	PRJEB58216
GCA 916161845	PDT001141248.1	Klebsiella_383	PDS000133910.8	2013	Netherlands	PRJEB41042
GCA 947660355	PDT001594171.1	Klebsiella_383	PDS000133910.8	2012	Greece	PRJEB58216
GCA 028091395	PDT001585517.1	Klebsiella_383	PDS000133910.8	2012	Greece	PRJEB58216
GCA 947660315	PDT001594129.1	Klebsiella_383	PDS000133910.8	2012	Greece	PRJEB58216
GCA 028094055	PDT001585432.1	Klebsiella_383	PDS000133910.8	2012	Greece	PRJEB58216
GCA 900502315	PDT000369990.1	Klebsiella_383	PDS000133910.8	2014	Greece	PRJEB10018
GCA 900500905	PDT000369957.1	Klebsiella_383	PDS000133910.8	2014	Greece	PRJEB10018
GCA 902156555	PDT000663220.1	Klebsiella_383	PDS000133910.8	2014	Greece	PRJEB33308
GCA 028090855	PDT001585545.1	Klebsiella_383	PDS000133910.8	2014	Greece	PRJEB58216
GCA 947663335	PDT001592164.1	Klebsiella_383	PDS000133910.8	2014	Greece	PRJEB58216
GCA 033900045	PDT002000540.1	Klebsiella_383	PDS000133910.8	nan	Germany	PRJNA1036598
GCA 947662625	PDT001592161.1	Klebsiella_383	PDS000133910.8	2018	Greece	PRJEB58216
GCA 028089315	PDT001585621.1	Klebsiella_383	PDS000133910.8	2018	Greece	PRJEB58216
GCA 028094275	PDT001585420.1	Klebsiella_383	PDS000133910.8	2011	Greece	PRJEB58216
GCA 947660045	PDT001592162.1	Klebsiella_383	PDS000133910.8	2011	Greece	PRJEB58216
GCA 916161185	PDT001141222.1	Klebsiella_383	PDS000133910.8	2013	Netherlands	PRJEB41042
GCA 028091215	PDT001585526.1	Klebsiella_383	PDS000133910.8	2013	Greece	PRJEB58216
GCA 028091235	PDT001585524.1	Klebsiella_383	PDS000133910.8	2013	Greece	PRJEB58216
GCA 001611095	PDT000123367.1	Klebsiella_383	PDS000133910.8	2013	Greece	PRJNA295651
GCA 947662095	PDT001594164.1	Klebsiella_383	PDS000133910.8	2013	Greece	PRJEB58216
GCA 947662275	PDT001594191.1	Klebsiella_383	PDS000133910.8	2013	Greece	PRJEB58216
GCA 028094175	PDT001585425.1	Klebsiella_383	PDS000133910.8	2011	Greece	PRJEB58216
GCA 028091295	PDT001585521.1	Klebsiella_383	PDS000133910.8	2013	Greece	PRJEB58216
GCA 947662185	PDT001592188.1	Klebsiella_383	PDS000133910.8	2013	Greece	PRJEB58216
GCA 947660105	PDT001592152.1	Klebsiella_383	PDS000133910.8	2011	Greece	PRJEB58216
GCA 947660445	PDT001594195.1	Klebsiella_383	PDS000133910.8	2012	Greece	PRJEB58216
GCA 947662295	PDT001592215.1	Klebsiella_383	PDS000133910.8	2012	Greece	PRJEB58216
GCA 028094135	PDT001585429.1	Klebsiella_383	PDS000133910.8	2012	Greece	PRJEB58216
GCA 028094075	PDT001585431.1	Klebsiella_383	PDS000133910.8	2012	Greece	PRJEB58216

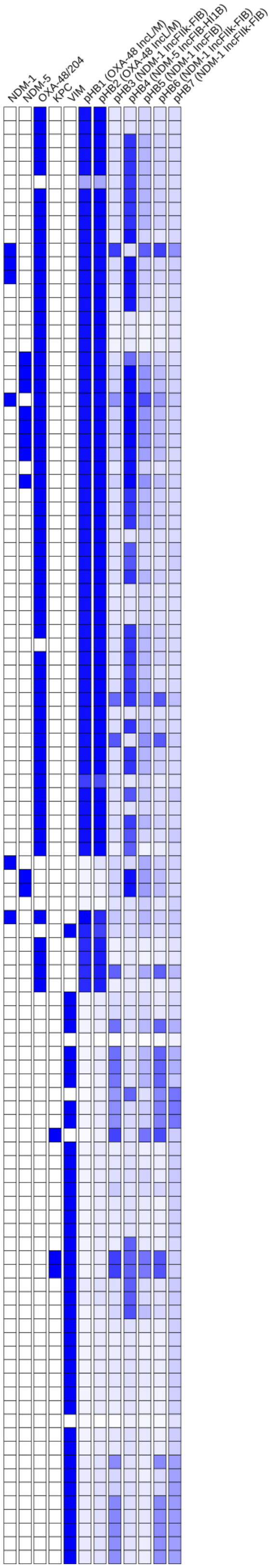

Tree 32: GCA_043905425

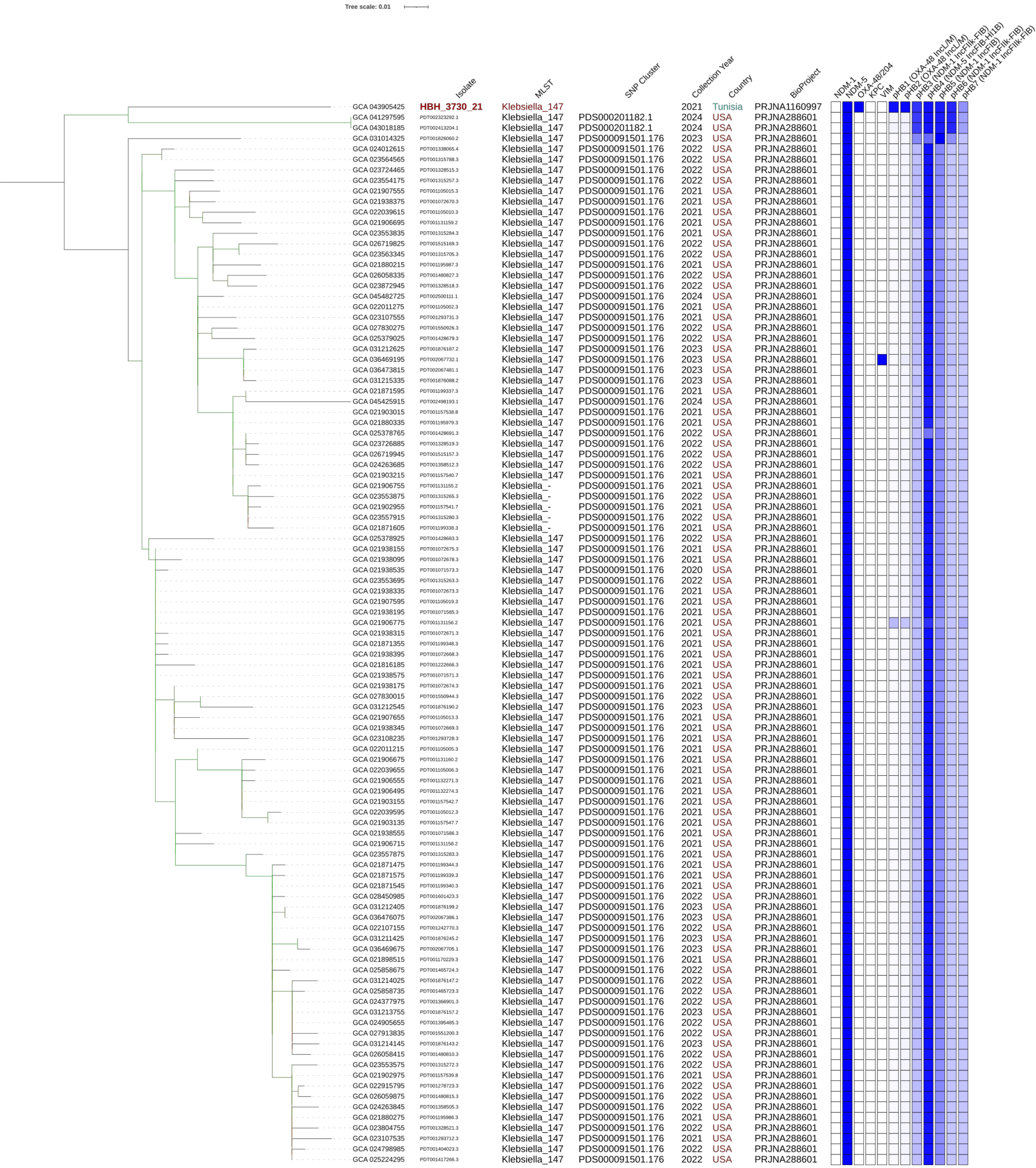

Tree 33: GCA_043905485

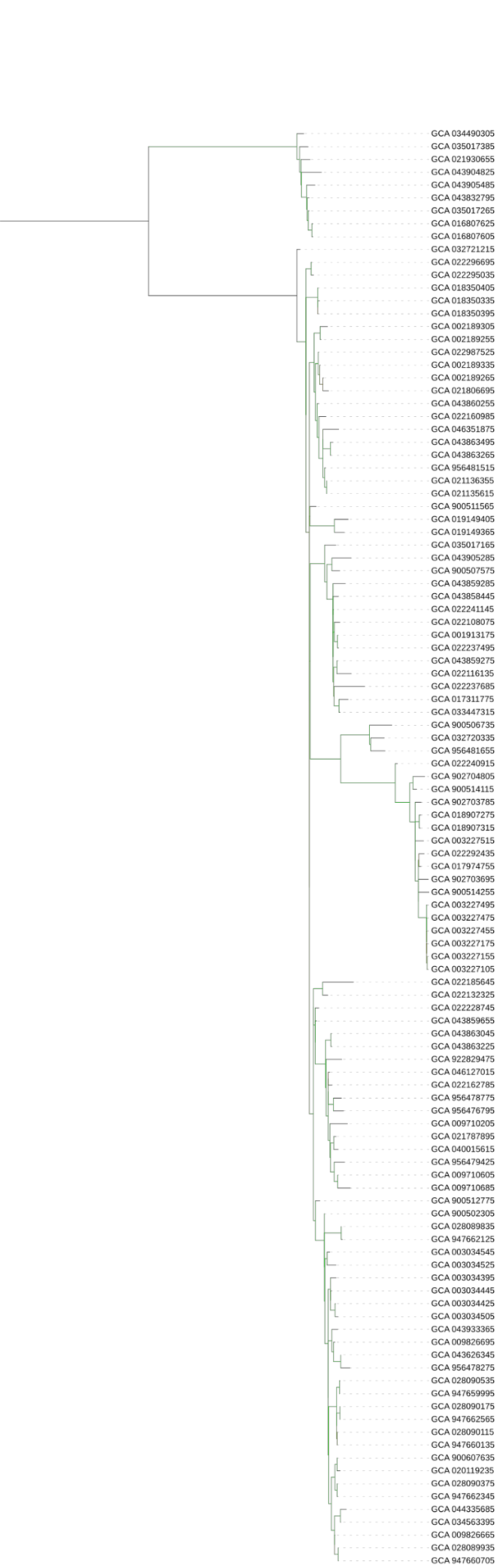

	Isolate	MLST	SNP Cluster	Collection Year	Country	BioProject
GCA 034490305	PDT002038556.1	Klebsiella_101		2019	France	PRJNA876630
GCA 035017385	PDT002045108.1	Klebsiella_101		2022	Libya	PRJNA998204
GCA 021930655	PDT001091033.3	Klebsiella_101		2021	USA	PRJNA288601
GCA 043904825	**HBH_4008_18**	Klebsiella_101		2018	Tunisia	PRJNA1160997
GCA 043905485	**HBH_957_22**	Klebsiella_101	PDS000080305.6	2022	Tunisia	PRJNA1160997
GCA 043832795	PDT002430498.1	Klebsiella_101	PDS000080305.6	2019	Germany	PRJNA1070536
GCA 035017265	PDT002045103.1	Klebsiella_101	PDS000080305.6	2022	Libya	PRJNA998204
GCA 016807625	PDT000949476.1	Klebsiella_101	PDS000080305.6	2020	Italy	PRJNA648931
GCA 016807605	PDT000949475.1	Klebsiella_101	PDS000080305.6	2020	Italy	PRJNA648931
GCA 032721215	PDT001927038.1	Klebsiella_101		2019	Italy	PRJNA1026096
GCA 022296695	PDT000423598.1	Klebsiella_-	PDS000199425.1	nan	NA	PRJEB17615
GCA 022295035	PDT000423690.1	Klebsiella_-	PDS000199425.1	nan	NA	PRJEB17615
GCA 018350405	PDT001033284.1	Klebsiella_101	PDS000088521.1	2014	Italy	PRJNA685961
GCA 018350335	PDT001033281.1	Klebsiella_101	PDS000088521.1	2014	Italy	PRJNA685961
GCA 018350395	PDT001033283.1	Klebsiella_101	PDS000088521.1	2014	Italy	PRJNA685961
GCA 002189305	PDT000217889.1	Klebsiella_101	PDS000199425.1	2015	Tunisia	PRJNA360369
GCA 002189255	PDT000217887.1	Klebsiella_101	PDS000199425.1	2015	Tunisia	PRJNA360320
GCA 022987525	PDT001286506.1	Klebsiella_101	PDS000199425.1	2013	Germany	PRJNA789142
GCA 002189335	PDT000217891.1	Klebsiella_101	PDS000199425.1	2014	Tunisia	PRJNA360296
GCA 002189265	PDT000217888.1	Klebsiella_101	PDS000199425.1	2014	Tunisia	PRJNA360291
GCA 021806695	PDT001227317.1	Klebsiella_101	PDS000199425.1	2015	United Kingdom	PRJNA788733
GCA 043860255	**HBH_417_14**	Klebsiella_101	PDS000199425.1	2014	Tunisia	PRJNA1160997
GCA 022160985	PDT001243464.1	Klebsiella_-	PDS000199425.1	2015	United Kingdom	PRJNA788733
GCA 046351875	PDT002549677.1	Klebsiella_101		2024	USA	PRJNA288601
GCA 043863495	**HBH_26_17**	Klebsiella_101	PDS000199425.1	2017	Tunisia	PRJNA1160997
GCA 043863265	**HBH_4479_16**	Klebsiella_101	PDS000199425.1	2016	Tunisia	PRJNA1160997
GCA 956481515	PDT001864169.1	Klebsiella_101	PDS000199425.1	2016	Turkey	PRJEB63349
GCA 021136355	PDT001201698.1	Klebsiella_101	PDS000199425.1	2018	Switzerland	PRJNA758223
GCA 021135615	PDT001201675.1	Klebsiella_101	PDS000199425.1	2018	Switzerland	PRJNA759850
GCA 900511565	PDT000369164.1	Klebsiella_101	PDS000199425.1	2014	France	PRJEB10018
GCA 019149405	PDT001085509.1	Klebsiella_101		2019	India	PRJNA548120
GCA 019149365	PDT001085507.1	Klebsiella_101		2019	India	PRJNA548120
GCA 035017165	PDT002045098.1	Klebsiella_101	PDS000041735.6	2022	Libya	PRJNA998204
GCA 043905285	**HBH_3900_20**	Klebsiella_101		2020	Tunisia	PRJNA1160997
GCA 900507575	PDT000370572.1	Klebsiella_101		2014	Turkey	PRJEB10018
GCA 043859285	**HBH_3025_12**	Klebsiella_101	PDS000041735.6	2012	Tunisia	PRJNA1160997
GCA 043858445	**HBH_95_11**	Klebsiella_101	PDS000041735.6	2011	Tunisia	PRJNA1160997
GCA 022241145	PDT000441621.1	Klebsiella_101	PDS000041735.6	2010	Germany	PRJEB18621
GCA 022108075	PDT001244416.1	Klebsiella_-	PDS000041735.6	2014	United Kingdom	PRJNA788733
GCA 001913175	PDT000174387.1	Klebsiella_101	PDS000041735.6	2013	Germany	PRJNA329409
GCA 022237495	PDT000441695.1	Klebsiella_101	PDS000041735.6	2013	Germany	PRJEB18621
GCA 043859275	**HBH_722_12**	Klebsiella_101	PDS000041735.6	2012	Tunisia	PRJNA1160997
GCA 022116135	PDT001245102.1	Klebsiella_101		nan	United Kingdom	PRJNA788733
GCA 022237685	PDT000441692.1	Klebsiella_101		2013	Germany	PRJEB18621
GCA 017311775	PDT000984769.1	Klebsiella_101		2017	China	PRJNA648010
GCA 033447315	PDT001974683.1	Klebsiella_101	PDS000041735.6	2012	China	PRJNA1035291
GCA 900506735	PDT000370529.1	Klebsiella_101		2014	Belgium	PRJEB10018
GCA 032720335	PDT001927077.1	Klebsiella_101	PDS000199425.1	2020	Italy	PRJNA1026096
GCA 956481655	PDT001864141.1	Klebsiella_101		2017	Turkey	PRJEB63349
GCA 022240915	PDT000441625.1	Klebsiella_101	PDS000041735.6	2011	Germany	PRJEB18621
GCA 902704805	PDT000662758.1	Klebsiella_101		2016	Italy	PRJEB22890
GCA 900514115	PDT000369585.1	Klebsiella_101	PDS000045320.1	2013	Italy	PRJEB10018
GCA 902703785	PDT000662659.1	Klebsiella_101	PDS000045311.30	2016	Italy	PRJEB22890
GCA 018907275	PDT001072374.1	Klebsiella_101	PDS000045311.30	2012	Italy	PRJNA608247
GCA 018907315	PDT001072375.1	Klebsiella_101	PDS000045311.30	2013	Italy	PRJNA608247
GCA 003227515	PDT000331482.1	Klebsiella_101	PDS000045312.1	2016	Italy	PRJNA431724
GCA 022292435	PDT000427105.1	Klebsiella_101	PDS000045311.30	nan	NA	PRJEB10018
GCA 017974755	PDT001007066.1	Klebsiella_101	PDS000045311.30	2017	Italy	PRJNA687927
GCA 902703695	PDT000662653.1	Klebsiella_101	PDS000055633.53	2016	Italy	PRJEB22890
GCA 900514255	PDT000369440.1	Klebsiella_101	PDS000045311.30	2013	Italy	PRJEB10018
GCA 003227495	PDT000331481.1	Klebsiella_2502	PDS000045311.30	2016	Italy	PRJNA431724
GCA 003227475	PDT000331480.1	Klebsiella_2502	PDS000045311.30	2016	Italy	PRJNA431724
GCA 003227455	PDT000331479.1	Klebsiella_2502	PDS000045311.30	2016	Italy	PRJNA431724
GCA 003227175	PDT000331467.1	Klebsiella_2502	PDS000045311.30	2016	Italy	PRJNA431724
GCA 003227155	PDT000331465.1	Klebsiella_2502	PDS000045311.30	2016	Italy	PRJNA431724
GCA 003227105	PDT000331462.1	Klebsiella_2502	PDS000045311.30	2016	Italy	PRJNA431724
GCA 022185645	PDT000716609.1	Klebsiella_101		2018	France	PRJEB35890
GCA 022132325	PDT001244810.1	Klebsiella_101		2014	United Kingdom	PRJNA788733
GCA 022228745	PDT000446086.1	Klebsiella_101	PDS000199425.1	2015	Germany	PRJEB18059
GCA 043859655	**HBH_2166_13**	Klebsiella_101	PDS000199425.1	2013	Tunisia	PRJNA1160997
GCA 043863045	**HBH_4442_15**	Klebsiella_101	PDS000188993.3	2015	Tunisia	PRJNA1160997
GCA 043863225	**HBH_275_16**	Klebsiella_101	PDS000188993.3	2016	Tunisia	PRJNA1160997
GCA 922829475	PDT001211067.1	Klebsiella_101	PDS000188993.3	2015	United Kingdom	PRJEB48990
GCA 046127015	PDT002538802.1	Klebsiella_101	PDS000188993.3	2015	New Zealand	PRJNA1102395
GCA 022162785	PDT001243259.1	Klebsiella_101	PDS000188993.3	2015	United Kingdom	PRJNA788733
GCA 956478775	PDT001861761.1	Klebsiella_3367		2017	Romania	PRJEB63349
GCA 956476795	PDT001861753.1	Klebsiella_101		2017	Romania	PRJEB63349
GCA 009710205	PDT000637199.1	Klebsiella_101		2018/2019	Romania	PRJNA579879
GCA 021787895	PDT001227768.1	Klebsiella_101	PDS000188993.3	2015	United Kingdom	PRJNA788733
GCA 040015615	PDT002187526.1	Klebsiella_101	PDS000188993.3	2015	Netherlands	PRJNA1116077
GCA 956479425	PDT001863967.2	Klebsiella_101		2017	Romania	PRJEB63349
GCA 009710605	PDT000637218.1	Klebsiella_101	PDS000188993.3	2018/2019	Romania	PRJNA579879
GCA 009710685	PDT000637223.1	Klebsiella_101	PDS000188993.3	2018/2019	Romania	PRJNA579879
GCA 900512775	PDT000369255.1	Klebsiella_101	PDS000199425.1	2014	Germany	PRJEB10018
GCA 900502305	PDT000369989.1	Klebsiella_101	PDS000199424.2	2014	Greece	PRJEB10018
GCA 028089835	PDT001585588.1	Klebsiella_101	PDS000199424.2	2017	Greece	PRJEB58216
GCA 947662125	PDT001592247.1	Klebsiella_101	PDS000199424.2	2017	Greece	PRJEB58216
GCA 003034545	PDT000304395.1	Klebsiella_101	PDS000199424.2	2015	Greece	PRJNA419295
GCA 003034525	PDT000304394.1	Klebsiella_101	PDS000199424.2	2015	Greece	PRJNA419295
GCA 003034395	PDT000304387.1	Klebsiella_101	PDS000199424.2	2015	Greece	PRJNA419295
GCA 003034445	PDT000304391.1	Klebsiella_101	PDS000199424.2	2015	Greece	PRJNA419295
GCA 003034425	PDT000304389.1	Klebsiella_101	PDS000199424.2	2016	Greece	PRJNA419295
GCA 003034505	PDT000304393.1	Klebsiella_101	PDS000199424.2	2016	Greece	PRJNA419295
GCA 043933365	PDT002412058.1	Klebsiella_101	PDS000199424.2	2016	Canada	PRJNA855907
GCA 009826695	PDT000663153.1	Klebsiella_101	PDS000199424.2	2016	Greece	PRJNA597427
GCA 043626345	PDT002332935.1	Klebsiella_101	PDS000199424.2	nan	Netherlands	PRJEB55414
GCA 956478275	PDT001861514.1	Klebsiella_101	PDS000199424.2	2018	Greece	PRJEB63349
GCA 028090535	PDT001585562.1	Klebsiella_101	PDS000199424.2	2015	Greece	PRJEB58216
GCA 947659995	PDT001594126.1	Klebsiella_101	PDS000199424.2	2015	Greece	PRJEB58216
GCA 028090175	PDT001585563.1	Klebsiella_101	PDS000199424.2	2016	Greece	PRJEB58216
GCA 947662565	PDT001594138.1	Klebsiella_101	PDS000199424.2	2016	Greece	PRJEB58216
GCA 028090115	PDT001585559.1	Klebsiella_101	PDS000199424.2	2015	Greece	PRJEB58216
GCA 947660135	PDT001594175.1	Klebsiella_101	PDS000199424.2	2015	Greece	PRJEB58216
GCA 900607635	PDT000411391.1	Klebsiella_101	PDS000199424.2	2016	Switzerland	PRJEB28660
GCA 020119235	PDT001137053.1	Klebsiella_101	PDS000199424.2	2016	Switzerland	PRJNA543274
GCA 028090375	PDT001585579.1	Klebsiella_101	PDS000199424.2	2016	Greece	PRJEB58216
GCA 947662345	PDT001592169.1	Klebsiella_101	PDS000199424.2	2016	Greece	PRJEB58216
GCA 044335685	PDT002305468.1	Klebsiella_101	PDS000199424.2	nan	USA	PRJNA271899
GCA 034563395	PDT002037997.1	Klebsiella_101	PDS000199424.2	2023	USA	PRJNA288601
GCA 009826665	PDT000663152.1	Klebsiella_101	PDS000199424.2	2015	Greece	PRJNA597427
GCA 028089935	PDT001585574.1	Klebsiella_101	PDS000199424.2	2016	Greece	PRJEB58216
GCA 947660705	PDT001590391.1	Klebsiella_101	PDS000199424.2	2016	Greece	PRJEB58216

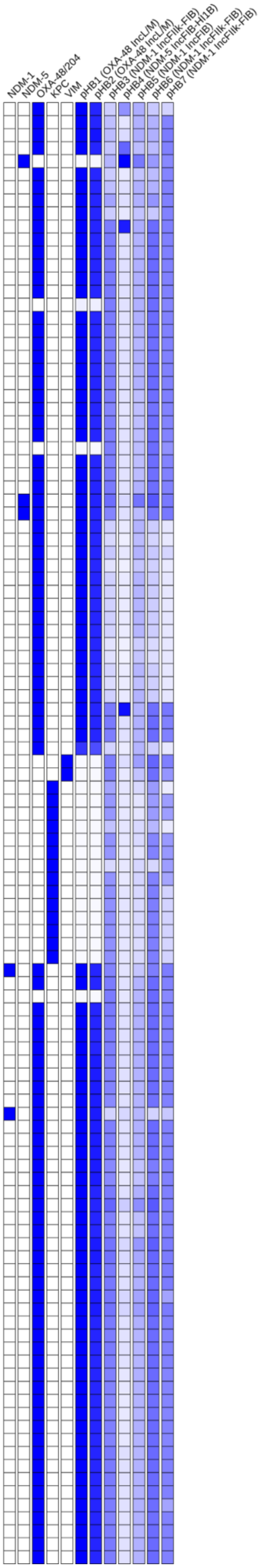

Tree 34: GCA_043905525

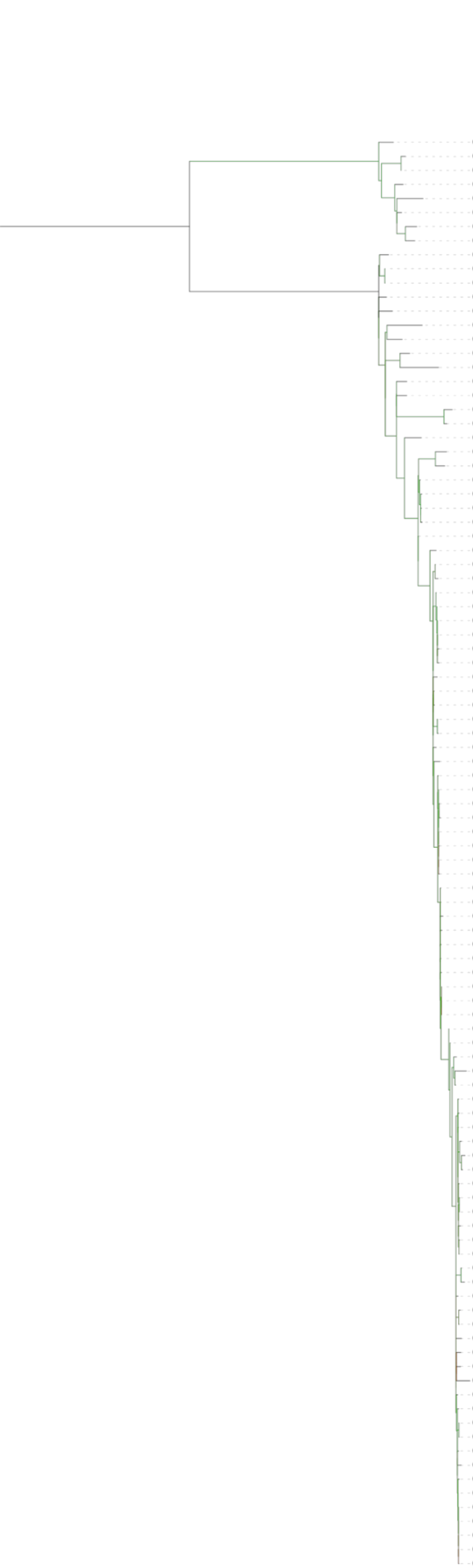

	Isolate	MLST	SNP Cluster	Collection Year	Country	BioProject
GCA 022306535	PDT000176097.2	Klebsiella_13		nan	NA	PRJEB5065
GCA 900503065	PDT000370229.1	Klebsiella_13	PDS000098778.2	2014	United Kingdom	PRJEB10018
GCA 022194405	PDT000715140.1	Klebsiella_-	PDS000098778.2	2014	United Kingdom	PRJNA564424
GCA 045752805	PDT002512410.1	Klebsiella_13	PDS000173925.11	2024	USA	PRJNA288601
GCA 036811575	PDT002084576.1	Klebsiella_13	PDS000053017.24	2024	USA	PRJNA288601
GCA 024430215	PDT001366426.1	Klebsiella_13	PDS000053017.24	nan	USA	PRJNA825705
GCA 900508405	PDT000370632.1	Klebsiella_13	PDS000053017.24	2013	Turkey	PRJEB10018
GCA 045662225	PDT002505954.1	Klebsiella_13	PDS000211070.1	2024	USA	PRJNA288601
GCA 024489195	PDT001373413.1	Klebsiella_13		2019	Spain	PRJEB50822
GCA 041765315	PDT002366259.1	Klebsiella_13	PDS000197052.1	2015	Norway	PRJNA1098507
GCA 033122965	PDT001958059.1	Klebsiella_13	PDS000197052.1	2015	Norway	PRJEB27256
GCA 031038295	PDT001717196.3	Klebsiella_13		2023	USA	PRJNA288601
GCA 013604195	PDT000797598.1	Klebsiella_13		nan	Spain	PRJNA609897
GCA 021816085	PDT001219145.1	Klebsiella_13		2018	Germany	PRJEB43549
GCA 043905525	**HBH_1304_11**	Klebsiella_13		2011	Tunisia	PRJNA1160997
GCA 022313435	PDT000136299.2	Klebsiella_13		2010	United Kingdom	PRJNA306133
GCA 905331375	PDT001185214.2	Klebsiella_13		2018	Spain	PRJEB42440
GCA 044382055	PDT002456491.1	Klebsiella_13		nan	Saudi Arabia	PRJNA1116866
GCA 900513595	PDT000369532.1	Klebsiella_13		2014	Israel	PRJEB10018
GCA 041423215	PDT002335078.1	Klebsiella_13	PDS000194059.1	2017	Portugal	PRJNA986835
GCA 041422095	PDT002335021.1	Klebsiella_13	PDS000194059.1	2017	Portugal	PRJNA986835
GCA 008374255	PDT000586403.1	Klebsiella_13		2018	France	PRJNA563544
GCA 008374425	PDT000586411.1	Klebsiella_13	PDS000056122.9	2018	France	PRJNA563544
GCA 008374895	PDT000586854.1	Klebsiella_13	PDS000056122.9	2018	France	PRJNA563544
GCA 041422315	PDT002335032.1	Klebsiella_13	PDS000056122.9	2017	Portugal	PRJNA986835
GCA 041421545	PDT002334995.1	Klebsiella_13	PDS000056122.9	2017	Portugal	PRJNA986835
GCA 041421595	PDT002334998.1	Klebsiella_13	PDS000056122.9	2017	Portugal	PRJNA986835
GCA 041422115	PDT002335022.1	Klebsiella_13	PDS000056122.9	2017	Portugal	PRJNA986835
GCA 041423635	PDT002335122.1	Klebsiella_13	PDS000056122.9	2017	Portugal	PRJNA986835
GCA 008374475	PDT000586413.1	Klebsiella_13	PDS000056122.9	2018	France	PRJNA563544
GCA 041423075	PDT002335070.1	Klebsiella_13	PDS000056122.9	2017	Portugal	PRJNA986835
GCA 041422815	PDT002335057.1	Klebsiella_13	PDS000056122.9	2017	Portugal	PRJNA986835
GCA 041424975	PDT002335179.1	Klebsiella_13	PDS000056122.9	2017	Portugal	PRJNA986835
GCA 041424775	PDT002335169.1	Klebsiella_13	PDS000056122.9	2017	Portugal	PRJNA986835
GCA 041421345	PDT002334985.1	Klebsiella_13	PDS000056122.9	2017	Portugal	PRJNA986835
GCA 041424935	PDT002335177.1	Klebsiella_13	PDS000056122.9	2017	Portugal	PRJNA986835
GCA 041422035	PDT002335018.1	Klebsiella_13	PDS000056122.9	2017	Portugal	PRJNA986835
GCA 041421445	PDT002334990.1	Klebsiella_13	PDS000056122.9	2017	Portugal	PRJNA986835
GCA 041421695	PDT002335003.1	Klebsiella_-	PDS000056122.9	2017	Portugal	PRJNA986835
GCA 016643445	PDT000933591.1	Klebsiella_13	PDS000056122.9	nan	Portugal	PRJNA602991
GCA 016643345	PDT000933586.1	Klebsiella_13	PDS000056122.9	nan	Portugal	PRJNA602991
GCA 025878685	PDT001467903.1	Klebsiella_13	PDS000056122.9	2018	Norway	PRJEB56146
GCA 947044045	PDT001449714.1	Klebsiella_13	PDS000056122.9	nan	NA	PRJEB41590
GCA 041421935	PDT002335013.1	Klebsiella_13	PDS000056122.9	2017	Portugal	PRJNA986835
GCA 041423615	PDT002335121.1	Klebsiella_13	PDS000056122.9	2017	Portugal	PRJNA986835
GCA 041421995	PDT002335016.1	Klebsiella_13	PDS000056122.9	2017	Portugal	PRJNA986835
GCA 041422935	PDT002335063.1	Klebsiella_13	PDS000056122.9	2017	Portugal	PRJNA986835
GCA 041422895	PDT002335061.1	Klebsiella_13	PDS000056122.9	2017	Portugal	PRJNA986835
GCA 947044275	PDT001449702.1	Klebsiella_13	PDS000056122.9	nan	NA	PRJEB41590
GCA 041422995	PDT002335066.1	Klebsiella_13	PDS000056122.9	2017	Portugal	PRJNA986835
GCA 947043995	PDT001449707.1	Klebsiella_13	PDS000056122.9	nan	NA	PRJEB41590
GCA 041422875	PDT002335060.1	Klebsiella_13	PDS000056122.9	2017	Portugal	PRJNA986835
GCA 947044465	PDT001449703.1	Klebsiella_13	PDS000056122.9	nan	NA	PRJEB41590
GCA 041422755	PDT002335054.1	Klebsiella_13	PDS000056122.9	2017	Portugal	PRJNA986835
GCA 016643325	PDT000933585.1	Klebsiella_13	PDS000056122.9	nan	Portugal	PRJNA602991
GCA 947044485	PDT001449716.1	Klebsiella_13	PDS000056122.9	nan	NA	PRJEB41590
GCA 041422695	PDT002335051.1	Klebsiella_13	PDS000056122.9	2017	Portugal	PRJNA986835
GCA 041422655	PDT002335049.1	Klebsiella_13	PDS000056122.9	2017	Portugal	PRJNA986835
GCA 041424895	PDT002335176.1	Klebsiella_13	PDS000056122.9	2017	Portugal	PRJNA986835
GCA 041423095	PDT002335071.1	Klebsiella_13	PDS000056122.9	2017	Portugal	PRJNA986835
GCA 947044315	PDT001449713.1	Klebsiella_13	PDS000056122.9	nan	NA	PRJEB41590
GCA 041422475	PDT002335040.1	Klebsiella_13	PDS000056122.9	2017	Portugal	PRJNA986835
GCA 947044035	PDT001449717.1	Klebsiella_13	PDS000056122.9	nan	NA	PRJEB41590
GCA 041422495	PDT002335041.1	Klebsiella_13	PDS000056122.9	2017	Portugal	PRJNA986835
GCA 041423155	PDT002335074.1	Klebsiella_13	PDS000056122.9	2017	Portugal	PRJNA986835
GCA 041422675	PDT002335050.1	Klebsiella_13	PDS000056122.9	2017	Portugal	PRJNA986835
GCA 008374405	PDT000586410.1	Klebsiella_13	PDS000056122.9	2018	France	PRJNA563544
GCA 008374205	PDT000586400.1	Klebsiella_13	PDS000056122.9	2018	France	PRJNA563544
GCA 041423735	PDT002335129.1	Klebsiella_13	PDS000056122.9	2017	Portugal	PRJNA986835
GCA 041423715	PDT002335128.1	Klebsiella_13	PDS000056122.9	2017	Portugal	PRJNA986835
GCA 041423515	PDT002335110.1	Klebsiella_13	PDS000056122.9	2017	Portugal	PRJNA986835
GCA 041423695	PDT002335127.1	Klebsiella_13	PDS000056122.9	2017	Portugal	PRJNA986835
GCA 008374515	PDT000586415.1	Klebsiella_13	PDS000056122.9	2018	France	PRJNA563544
GCA 041421915	PDT002335012.1	Klebsiella_13	PDS000056122.9	2017	Portugal	PRJNA986835
GCA 041423495	PDT002335108.1	Klebsiella_13	PDS000056122.9	2017	Portugal	PRJNA986835
GCA 041422375	PDT002335039.1	Klebsiella_13	PDS000056122.9	2017	Portugal	PRJNA986835
GCA 041423395	PDT002335098.1	Klebsiella_13	PDS000056122.9	2017	Portugal	PRJNA986835
GCA 041422415	PDT002335036.1	Klebsiella_13	PDS000056122.9	2017	Portugal	PRJNA986835
GCA 041422255	PDT002335029.1	Klebsiella_13	PDS000056122.9	2017	Portugal	PRJNA986835
GCA 041422355	PDT002335034.1	Klebsiella_13	PDS000056122.9	2017	Portugal	PRJNA986835
GCA 041423035	PDT002335068.1	Klebsiella_13	PDS000056122.9	2017	Portugal	PRJNA986835
GCA 947044455	PDT001449709.1	Klebsiella_13	PDS000056122.9	nan	NA	PRJEB41590
GCA 016643475	PDT000933593.1	Klebsiella_13	PDS000056122.9	nan	Portugal	PRJNA602991
GCA 041422975	PDT002335065.1	Klebsiella_13	PDS000056122.9	2017	Portugal	PRJNA986835
GCA 041422775	PDT002335055.1	Klebsiella_13	PDS000056122.9	2017	Portugal	PRJNA986835
GCA 041424655	PDT002335163.1	Klebsiella_13	PDS000056122.9	2017	Portugal	PRJNA986835
GCA 041423815	PDT002335133.1	Klebsiella_13	PDS000056122.9	2017	Portugal	PRJNA986835
GCA 016643375	PDT000933588.1	Klebsiella_13	PDS000056122.9	nan	Portugal	PRJNA602991
GCA 041425055	PDT002335183.1	Klebsiella_13	PDS000056122.9	2017	Portugal	PRJNA986835
GCA 041422905	PDT002335062.1	Klebsiella_13	PDS000056122.9	2017	Portugal	PRJNA986835
GCA 041422955	PDT002335064.1	Klebsiella_13	PDS000056122.9	2017	Portugal	PRJNA986835
GCA 947044055	PDT001449700.1	Klebsiella_13	PDS000056122.9	nan	NA	PRJEB41590
GCA 041422555	PDT002335044.1	Klebsiella_13	PDS000056122.9	2017	Portugal	PRJNA986835
GCA 041423055	PDT002335069.1	Klebsiella_13	PDS000056122.9	2017	Portugal	PRJNA986835
GCA 947044015	PDT001449706.1	Klebsiella_13	PDS000056122.9	nan	NA	PRJEB41590
GCA 041422515	PDT002335042.1	Klebsiella_13	PDS000056122.9	2017	Portugal	PRJNA986835
GCA 041422835	PDT002335058.1	Klebsiella_13	PDS000056122.9	2017	Portugal	PRJNA986835
GCA 041422795	PDT002335056.1	Klebsiella_13	PDS000056122.9	2017	Portugal	PRJNA986835
GCA 041422635	PDT002335048.1	Klebsiella_13	PDS000056122.9	2017	Portugal	PRJNA986835
GCA 041422725	PDT002335053.1	Klebsiella_13	PDS000056122.9	2017	Portugal	PRJNA986835
GCA 041422715	PDT002335052.1	Klebsiella_13	PDS000056122.9	2017	Portugal	PRJNA986835
GCA 947044345	PDT001449710.1	Klebsiella_13	PDS000056122.9	nan	NA	PRJEB41590

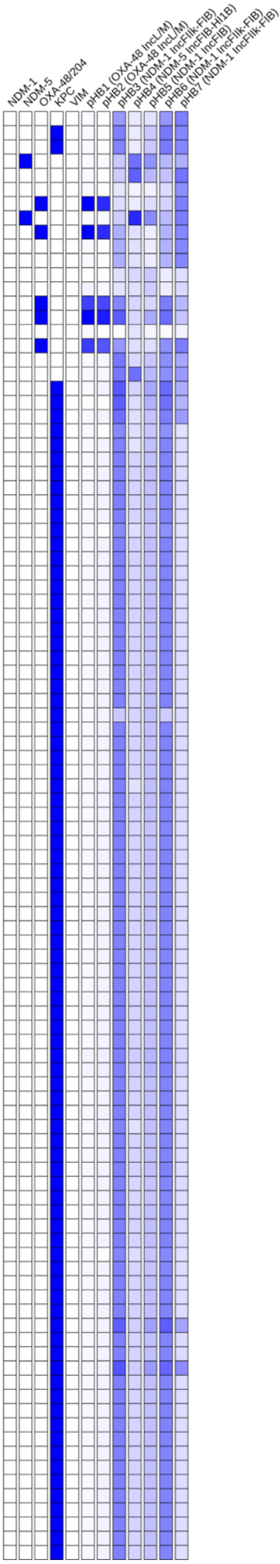

Tree 35: GCA_043905545

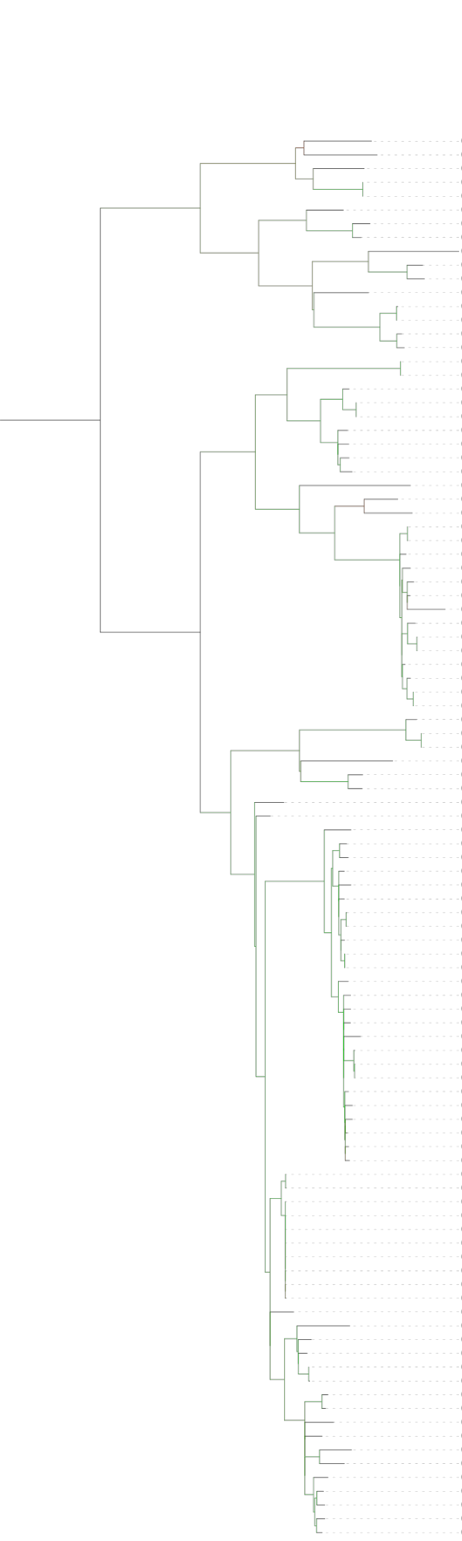

	Isolate	MLST	SNP Cluster	Collection Year	Country	BioProject
GCA 021977355	PDT001011395.1	Klebsiella_29	PDS000198821.1	2015	Norway	PRJEB42350
GCA 900508655	PDT000370652.1	Klebsiella_29		2014	Turkey	PRJEB10018
GCA 022203885	PDT000674649.1	Klebsiella_-		2018	Italy	PRJEB27342
GCA 027949505	PDT001580435.1	Klebsiella_5832	PDS000133597.1	2020	Norway	PRJNA922900
GCA 027948585	PDT001580434.1	Klebsiella_5832	PDS000133597.1	2020	Norway	PRJNA922900
GCA 021918425	PDT001092322.1	Klebsiella_29		2009	Norway	PRJEB27256
GCA 900085545	PDT000131510.1	Klebsiella_29	PDS000101853.1	2010	United Kingdom	PRJEB5065
GCA 031068225	PDT001702682.1	Klebsiella_29	PDS000143192.1	2020	Japan	PRJDB10842
GCA 022308535	PDT000160695.2	Klebsiella_29		nan	NA	PRJEB3255
GCA 021925205	PDT001091910.1	Klebsiella_29		2005	Norway	PRJEB27256
GCA 033336335	PDT001971216.1	Klebsiella_29		2021	Uganda	PRJNA1015582
GCA 045487145	PDT002191133.1	Klebsiella_29		2018	USA	PRJNA1054115
GCA 023030525	PDT001289249.1	Klebsiella_29	PDS000108354.1	2020	Australia	PRJNA777643
GCA 023032605	PDT001289252.1	Klebsiella_29	PDS000108354.1	2020	Australia	PRJNA777643
GCA 031066225	PDT001703026.1	Klebsiella_29		2019	Japan	PRJDB10842
GCA 034686605	PDT001704214.1	Klebsiella_-		2019	Japan	PRJDB10842
GCA 040200635	PDT002208441.1	Klebsiella_29	PDS000201454.1	nan	Saudi Arabia	PRJNA1116866
GCA 043358195	PDT002423142.1	Klebsiella_29	PDS000201454.1	nan	Saudi Arabia	PRJNA1116866
GCA 022353805	PDT001253425.1	Klebsiella_29		2018	China	PRJNA603241
GCA 030413005	PDT001813072.1	Klebsiella_29	PDS000156350.1	2016	USA	PRJNA981468
GCA 030845755	PDT001858214.1	Klebsiella_29	PDS000156350.1	2016	USA	PRJNA981468
GCA 031800875	PDT001893992.1	Klebsiella_29		2021	China	PRJNA870270
GCA 036912695	PDT002094567.1	Klebsiella_29		2021	China	PRJNA983945
GCA 030874045	PDT001701745.1	Klebsiella_29		2020	Japan	PRJDB10842
GCA 021532695	PDT001228190.1	Klebsiella_29		2021	China	PRJNA795018
GCA 902509305	PDT000663049.1	Klebsiella_29		2017	China	PRJEB34922
GCA 042769255	PDT002054255.1	Klebsiella_29		2020	Colombia	PRJNA937403
GCA 022332205	PDT000537781.1	Klebsiella_29		2012	United Kingdom	PRJEB12817
GCA 026005715	PDT001481600.1	Klebsiella_29	PDS000122861.1	2016	Japan	PRJDB12116
GCA 024451885	PDT001370202.1	Klebsiella_29	PDS000122861.1	2016	Japan	PRJDB12116
GCA 023740955	PDT001336572.1	Klebsiella_29		2019	China	PRJNA833902
GCA 029523555	PDT001676485.1	Klebsiella_29		2018	China	PRJNA810290
GCA 026156585	PDT001485826.1	Klebsiella_29		2016	China	PRJNA844536
GCA 038376465	PDT002142127.1	Klebsiella_29		2021	China	PRJNA1081623
GCA 034730205	PDT002041891.1	Klebsiella_29		2021	China	PRJNA1050895
GCA 021850305	PDT001214154.1	Klebsiella_29		2019	Japan	PRJDB11378
GCA 026005415	PDT001481585.1	Klebsiella_29	PDS000122913.1	2016	Japan	PRJDB12116
GCA 024476745	PDT001370188.1	Klebsiella_29	PDS000122913.1	2016	Japan	PRJDB12116
GCA 041038705	PDT002292051.1	Klebsiella_29		nan	China	PRJNA1021041
GCA 034726355	PDT002041759.1	Klebsiella_29		2021	China	PRJNA1050895
GCA 039843045	PDT002178271.1	Klebsiella_29	PDS000183535.1	2023	NA	PRJNA1113824
GCA 039610635	PDT002160672.1	Klebsiella_29	PDS000183535.1	2022	NA	PRJNA1100710
GCA 043905545	**HBH_1200_22**	Klebsiella_29		2022	Tunisia	PRJNA1160997
GCA 039608295	PDT002160555.1	Klebsiella_29	PDS000183538.1	2022	NA	PRJNA1100710
GCA 039843015	PDT002178269.1	Klebsiella_29	PDS000183538.1	2023	NA	PRJNA1113824
GCA 027828275	PDT001557989.1	Klebsiella_29		2013	Bangladesh	PRJNA845975
GCA 044041975	PDT002329503.1	Klebsiella_29		2021	Egypt	PRJEB76311
GCA 020684865	PDT001166301.1	Klebsiella_29		2012	Germany	PRJNA772925
GCA 032334535	PDT001913343.1	Klebsiella_714		2020	India	PRJNA852664
GCA 043993255	PDT002376544.1	Klebsiella_-		2017	Japan	PRJDB14656
GCA 004312085	PDT000471473.1	Klebsiella_29		2013	Japan	PRJDB7163
GCA 900494685	PDT000354614.1	Klebsiella_29		2016	Thailand	PRJEB19226
GCA 021982735	PDT001007730.1	Klebsiella_-		2016	Thailand	PRJDB5929
GCA 041032855	PDT002291098.1	Klebsiella_29		nan	China	PRJNA1021041
GCA 039607595	PDT002160520.1	Klebsiella_29	PDS000183548.1	2023	NA	PRJNA1100710
GCA 040691525	PDT002251078.1	Klebsiella_29		2021	China	PRJNA1131666
GCA 046464485	PDT002556449.1	Klebsiella_29	PDS000214440.1	nan	NA	PRJNA786399
GCA 046465035	PDT002556476.1	Klebsiella_29	PDS000214440.1	nan	NA	PRJNA786399
GCA 034356675	PDT002026158.1	Klebsiella_-	PDS000169967.2	2013	China	PRJNA1050414
GCA 963900435	PDT002108114.1	Klebsiella_29	PDS000169967.2	nan	NA	PRJEB71012
GCA 902507975	PDT000662920.1	Klebsiella_29	PDS000169967.2	2017	China	PRJEB34922
GCA 020478985	PDT001151298.1	Klebsiella_29		2019	China	PRJNA612328
GCA 003286975	PDT000343321.1	Klebsiella_29		2013	China	PRJNA477655
GCA 036329825	PDT002071150.1	Klebsiella_29	PDS000173086.1	2017	China	PRJNA930978
GCA 040989565	PDT002279952.1	Klebsiella_29		nan	China	PRJNA1021041
GCA 014283645	PDT000820994.1	Klebsiella_29	PDS000070366.1	2016	China	PRJNA657553
GCA 039613575	PDT002160772.1	Klebsiella_29	PDS000182318.2	2018	China	PRJNA1105228
GCA 008320705	PDT000837127.1	Klebsiella_29	PDS000182318.2	2018	China	PRJNA529337
GCA 045946195	PDT002531218.1	Klebsiella_29	PDS000182318.2	2018	China	PRJNA1190631
GCA 044443205	PDT002456657.1	Klebsiella_29		2020	China	PRJNA1056658
GCA 034727375	PDT002041805.1	Klebsiella_29		2021	China	PRJNA1050895
GCA 023742395	PDT001336644.1	Klebsiella_29		2020	China	PRJNA833902
GCA 028825795	PDT001641532.1	Klebsiella_29		2022	China	PRJNA903849
GCA 044444005	PDT002456679.1	Klebsiella_29		2019	China	PRJNA1056658
GCA 023742125	PDT001336631.1	Klebsiella_29		2020	China	PRJNA833902
GCA 040537895	PDT002244846.1	Klebsiella_29	PDS000188027.1	2011	Taiwan	PRJNA1090858
GCA 038593475	PDT002148605.1	Klebsiella_29	PDS000188027.1	2011	Taiwan	PRJNA1090858
GCA 040538425	PDT002244872.1	Klebsiella_29	PDS000188010.2	2010	Taiwan	PRJNA1090858
GCA 040538495	PDT002244874.1	Klebsiella_29	PDS000188010.2	2010	Taiwan	PRJNA1090858
GCA 040538525	PDT002244875.1	Klebsiella_29	PDS000188010.2	2010	Taiwan	PRJNA1090858
GCA 038689005	PDT002152188.1	Klebsiella_29	PDS000188010.2	1997/2010	Taiwan	PRJNA1090858
GCA 040538415	PDT002244873.1	Klebsiella_29	PDS000188010.2	2010	Taiwan	PRJNA1090858
GCA 040538465	PDT002244871.1	Klebsiella_29	PDS000188010.2	2010	Taiwan	PRJNA1090858
GCA 040538405	PDT002244870.1	Klebsiella_29	PDS000188010.2	2010	Taiwan	PRJNA1090858
GCA 040798025	PDT002262789.1	Klebsiella_29	PDS000188010.2	2010	Taiwan	PRJNA1090858
GCA 014840655	PDT000855238.1	Klebsiella_29		1998	Pakistan	PRJNA308116
GCA 030875965	PDT001701673.1	Klebsiella_29		2019	Japan	PRJDB10842
GCA 021872495	PDT001198342.1	Klebsiella_29		2016	Japan	PRJDB12075
GCA 021874755	PDT001198230.1	Klebsiella_29		2016	Japan	PRJDB12075
GCA 031067365	PDT001702868.1	Klebsiella_29	PDS000143149.1	2019	Japan	PRJDB10842
GCA 030810015	PDT001705085.1	Klebsiella_29	PDS000143149.1	2019	Japan	PRJDB10842
GCA 034739595	PDT002042385.1	Klebsiella_29		2021	China	PRJNA1050895
GCA 034733275	PDT002042085.1	Klebsiella_29		2020	China	PRJNA1050895
GCA 022233205	PDT000442997.1	Klebsiella_29		2014	Vietnam	PRJEB26814
GCA 021853415	PDT001214087.1	Klebsiella_29		2018	Japan	PRJDB11378
GCA 030872255	PDT001701841.1	Klebsiella_29		2019	Japan	PRJDB10842
GCA 009184885	PDT000615293.1	Klebsiella_29		2018	Japan	PRJNA577544
GCA 034731235	PDT002042012.1	Klebsiella_29		2021	China	PRJNA1050895
GCA 041198135	PDT002313554.1	Klebsiella_29		2015	China	PRJNA844536
GCA 036995805	PDT002098830.1	Klebsiella_29		2015	China	PRJNA1003408
GCA 014285475	PDT000822618.1	Klebsiella_29		2015	China	PRJNA657553
GCA 046468255	PDT002556637.1	Klebsiella_29		nan	NA	PRJNA786399

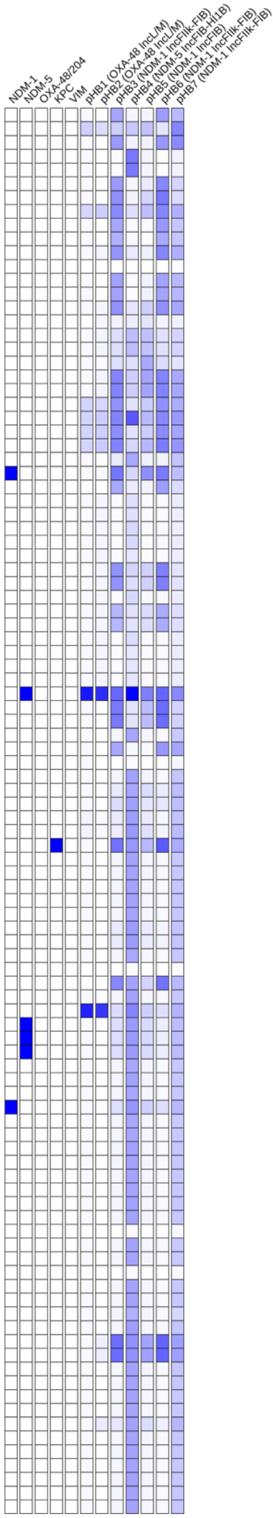

Tree 36: GCA_043905565

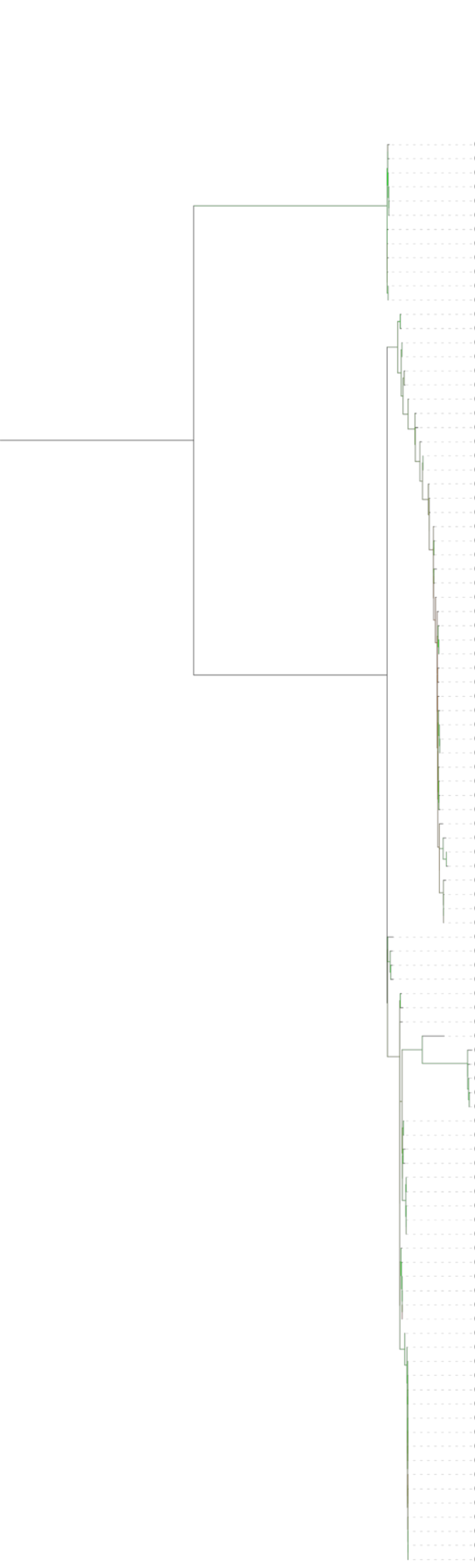

	Isolate	MLST	SNP Cluster	Collection Year	Country	BioProject
GCA 002186835	PDT000217809.1	Klebsiella_1418		2011	Nigeria	PRJNA351846
GCA 026672225	PDT001518703.1	Klebsiella_1418		2022	Ghana	PRJNA906096
GCA 021136215	PDT001201693.1	Klebsiella_1418	PDS000075208.5	2017	Switzerland	PRJNA758223
GCA 037051645	PDT002102057.1	Klebsiella_1418	PDS000075208.5	2013	Jordan	PRJNA1076682
GCA 022055125	PDT000879595.1	Klebsiella_1418	PDS000075208.5	2017	Qatar	PRJNA656934
GCA 042914675	PDT002097740.1	Klebsiella_1418	PDS000075208.5	2022	Netherlands	PRJNA1076808
GCA 040430465	PDT002237476.1	Klebsiella_1418		2021	Ireland	PRJNA1126319
GCA 043905565	**HBH_61_21**	Klebsiella_1418		2021	Tunisia	PRJNA1160997
GCA 900513125	PDT000369333.1	Klebsiella_1418		2013	Italy	PRJEB10018
GCA 015731075	PDT000903683.1	Klebsiella_1418		2019	China	PRJNA665928
GCA 033547775	PDT001982204.1	Klebsiella_1418	PDS000208562.1	2022	Saudi Arabia	PRJNA1018815
GCA 045063005	PDT002487922.1	Klebsiella_1418	PDS000208562.1	2022	Saudi Arabia	PRJNA1018815
GCA 021940255	PDT001049085.1	Klebsiella_3098	PDS000074703.6	2018	Australia	PRJNA857533
GCA 026256345	PDT001849263.1	Klebsiella_3098	PDS000074703.6	2013	Australia	PRJNA646837
GCA 041920645	PDT002382459.2	Klebsiella_45	PDS000198705.1	2007	Norway	PRJNA1098507
GCA 043384065	PDT002281230.1	Klebsiella_45	PDS000198705.1	2007	Norway	PRJEB27256
GCA 000826795	PDT000049482.1	Klebsiella_45		2012	USA	PRJNA261775
GCA 004322955	PDT000473852.1	Klebsiella_45		2014	USA	PRJNA279669
GCA 033103885	PDT001961101.1	Klebsiella_-	PDS000106393.3	2007	Spain	PRJEB41835
GCA 046126295	PDT002538872.1	Klebsiella_45	PDS000212921.2	2018	New Zealand	PRJNA1102395
GCA 022248895	PDT000440765.1	Klebsiella_-		nan	NA	PRJEB21605
GCA 033104365	PDT001961051.1	Klebsiella_45		2019	USA	PRJNA648389
GCA 046448265	PDT002554279.1	Klebsiella_45	PDS000214277.1	2014	Russia	PRJNA1196851
GCA 046449325	PDT002554332.1	Klebsiella_45	PDS000214277.1	2018	Russia	PRJNA1196851
GCA 036995725	PDT002098826.1	Klebsiella_45		2016	China	PRJNA1003408
GCA 022184005	PDT000723800.1	Klebsiella_45		2018	Italy	PRJEB27342
GCA 044322325	PDT002306240.1	Klebsiella_45		nan	USA	PRJNA271899
GCA 964300705	PDT002500299.1	Klebsiella_45	PDS000106675.2	2019	Germany	PRJEB81699
GCA 021975415	PDT001011543.1	Klebsiella_45		2015	Norway	PRJEB42350
GCA 956480335	PDT001864109.1	Klebsiella_45		2018	Spain	PRJEB63349
GCA 022098395	PDT001096792.3	Klebsiella_45		2021	USA	PRJNA288601
GCA 023739135	PDT001336493.1	Klebsiella_45		2021	China	PRJNA833902
GCA 022987545	PDT001286508.1	Klebsiella_45		2012	Germany	PRJNA789142
GCA 032134355	PDT001903162.1	Klebsiella_45		2017	Norway	PRJEB48268
GCA 043446965	PDT002421688.1	Klebsiella_45	PDS000179055.2	2019	USA	PRJNA288601
GCA 022211745	PDT000630652.1	Klebsiella_45	PDS000052944.18	2013	Canada	PRJNA564992
GCA 030019695	PDT001737461.1	Klebsiella_45	PDS000052944.18	2013	USA	PRJNA395086
GCA 016903895	PDT000967411.1	Klebsiella_45		nan	USA	PRJNA231221
GCA 022430345	PDT001256202.1	Klebsiella_45	PDS000105716.1	2020	USA	PRJNA325243
GCA 022312935	PDT000136333.2	Klebsiella_45		2011	United Kingdom	PRJNA306133
GCA 022285805	PDT000431395.1	Klebsiella_45		2017	USA	PRJNA396774
GCA 043331735	PDT002278350.1	Klebsiella_45	PDS000178660.3	2019	USA	PRJNA1106484
GCA 046015795	PDT002530113.1	Klebsiella_45	PDS000178660.3	2018	USA	PRJNA288601
GCA 022033615	PDT000901821.3	Klebsiella_45	PDS000178660.3	2018	USA	PRJNA288601
GCA 043569885	PDT002347492.1	Klebsiella_45	PDS000100594.2	2009	Norway	PRJEB27256
GCA 900511045	PDT000369071.1	Klebsiella_45		2013	Czechia	PRJEB10018
GCA 032046115	PDT001903439.1	Klebsiella_45		2018	Norway	PRJEB48268
GCA 032042675	PDT001903028.1	Klebsiella_45		2018	Norway	PRJEB48268
GCA 043709025	PDT002376631.1	Klebsiella_45		2018	Japan	PRJDB14656
GCA 900509075	PDT000370712.1	Klebsiella_45		2013	Turkey	PRJEB10018
GCA 916160865	PDT001141212.1	Klebsiella_45	PDS000127598.3	2019	Netherlands	PRJEB41042
GCA 026888235	PDT001525297.3	Klebsiella_-	PDS000127598.3	2022	USA	PRJNA288601
GCA 022284885	PDT000431439.1	Klebsiella_45	PDS000060037.37	2017	USA	PRJNA396774
GCA 022286675	PDT000431353.1	Klebsiella_45	PDS000060037.37	2017	USA	PRJNA396774
GCA 022199725	PDT000707850.1	Klebsiella_-	PDS000060037.37	nan	NA	PRJEB22903
GCA 044380335	PDT002305393.1	Klebsiella_45	PDS000060037.37	nan	USA	PRJNA271899
GCA 021922285	PDT001092054.1	Klebsiella_45		2013	Norway	PRJEB27256
GCA 022141525	PDT000870424.1	Klebsiella_45		2015	Romania	PRJNA396838
GCA 900515965	PDT000369667.1	Klebsiella_45		2014	Italy	PRJEB10018
GCA 900085275	PDT000131593.1	Klebsiella_45		2008	United Kingdom	PRJEB5065
GCA 019798025	PDT001120287.1	Klebsiella_2954		2015	Norway	PRJNA723772
GCA 021918225	PDT001092348.1	Klebsiella_45	PDS000093208.1	2009	Norway	PRJEB27256
GCA 018441205	PDT001046596.1	Klebsiella_45		2019	Germany	PRJNA692829
GCA 002192315	PDT000218722.1	Klebsiella_45	PDS000006905.10	nan	NA	PRJNA292902
GCA 026674905	PDT001518814.1	Klebsiella_45		2022	Ghana	PRJNA906096
GCA 032076315	PDT001904954.1	Klebsiella_45		2018	Norway	PRJEB48268
GCA 038593465	PDT002148604.1	Klebsiella_45	PDS000213518.2	2020	Malawi	PRJNA1102521
GCA 964207685	PDT002279439.1	Klebsiella_45	PDS000190165.1	2021	Tanzania	PRJEB78367
GCA 964207185	PDT002279454.1	Klebsiella_45	PDS000190165.1	2021	Tanzania	PRJEB78367
GCA 026256265	PDT001849267.1	Klebsiella_45	PDS000074716.5	2013	Australia	PRJNA646837
GCA 003432285	PDT000371002.1	Klebsiella_45	PDS000074716.5	2013	Australia	PRJNA486877
GCA 029577515	PDT001677826.1	Klebsiella_45		2019	Italy	PRJNA949542
GCA 043992935	PDT002376563.1	Klebsiella_45		2016	Japan	PRJDB14656
GCA 022276145	PDT000432070.1	Klebsiella_45	PDS000054827.4	2015	Japan	PRJDB6565
GCA 022207045	PDT000651374.1	Klebsiella_45	PDS000054827.4	2015	Japan	PRJDB6565
GCA 022207445	PDT000651336.1	Klebsiella_45	PDS000054827.4	2015	Japan	PRJDB6565
GCA 022207425	PDT000651334.1	Klebsiella_45	PDS000054827.4	2015	Japan	PRJDB6565
GCA 022204645	PDT000668791.1	Klebsiella_45	PDS000054827.4	2015	Japan	PRJDB6565
GCA 037052165	PDT002102083.1	Klebsiella_45		2015	Jordan	PRJNA1076682
GCA 964303885	PDT002500340.1	Klebsiella_45		2020	Germany	PRJEB81699
GCA 021865655	PDT001207408.1	Klebsiella_45	PDS000101897.1	2019	France	PRJEB34643
GCA 021865675	PDT001207409.1	Klebsiella_45	PDS000101897.1	2019	France	PRJEB34643
GCA 021866675	PDT001207293.1	Klebsiella_45	PDS000101897.1	2019	France	PRJEB34643
GCA 021865695	PDT001207404.1	Klebsiella_45	PDS000101897.1	2019	France	PRJEB34643
GCA 036476045	PDT002067384.1	Klebsiella_45	PDS000060037.37	2023	USA	PRJNA288601
GCA 031212105	PDT001876221.2	Klebsiella_45	PDS000060037.37	2023	USA	PRJNA288601
GCA 031211445	PDT001876244.2	Klebsiella_-	PDS000060037.37	2023	USA	PRJNA288601
GCA 045633225	PDT002500126.1	Klebsiella_-	PDS000060037.37	2024	USA	PRJNA288601
GCA 045720915	PDT002512352.1	Klebsiella_45	PDS000060037.37	2024	USA	PRJNA288601
GCA 045430815	PDT002498217.1	Klebsiella_45	PDS000060037.37	2023	USA	PRJNA288601
GCA 031209215	PDT001876301.2	Klebsiella_45	PDS000060037.37	2023	USA	PRJNA288601
GCA 031214895	PDT001876109.2	Klebsiella_45	PDS000060037.37	2023	USA	PRJNA288601
GCA 045731845	PDT002512390.1	Klebsiella_45	PDS000060037.37	2024	USA	PRJNA288601
GCA 036474115	PDT002067466.1	Klebsiella_45	PDS000060037.37	2023	USA	PRJNA288601
GCA 031208815	PDT001876309.2	Klebsiella_45	PDS000060037.37	2023	USA	PRJNA288601
GCA 026057255	PDT001480835.3	Klebsiella_-	PDS000060037.37	2022	USA	PRJNA288601
GCA 024905435	PDT001395499.3	Klebsiella_-	PDS000060037.37	2022	USA	PRJNA288601
GCA 031208865	PDT001876308.2	Klebsiella_-	PDS000060037.37	2023	USA	PRJNA288601
GCA 031211925	PDT001876219.2	Klebsiella_45	PDS000060037.37	2023	USA	PRJNA288601
GCA 031212885	PDT001876173.2	Klebsiella_45	PDS000060037.37	2023	USA	PRJNA288601
GCA 036693205	PDT002080954.1	Klebsiella_45	PDS000060037.37	2023	USA	PRJNA288601

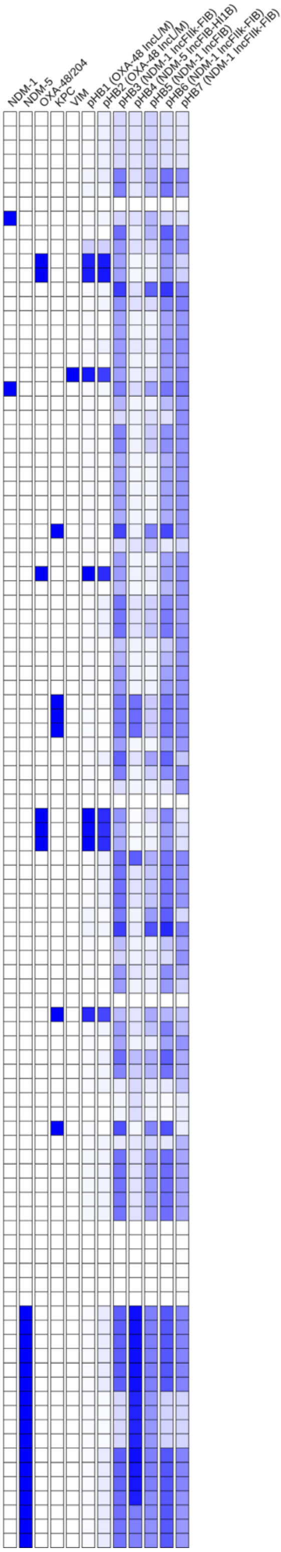

Tree 37: GCA_043905585

Tree scale: 0.01

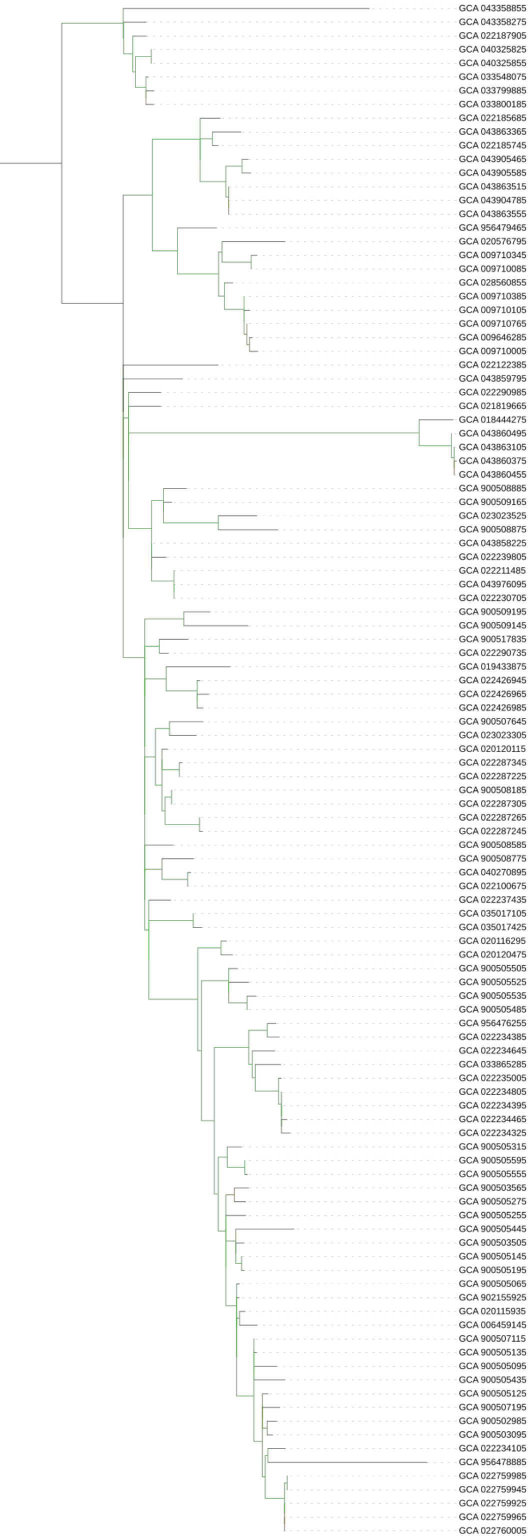

	Isolate	MLST	SNP Cluster	Collection Year	Country	BioProject
GCA 043358855	PDT002423174.1	Klebsiella_101	PDS000166495.5	nan	Saudi Arabia	PRJNA1116866
GCA 043358275	PDT002423146.1	Klebsiella_101	PDS000166495.5	nan	Saudi Arabia	PRJNA1116866
GCA 022187905	PDT000715723.1	Klebsiella_101	PDS000166495.5	2018	Saudi Arabia	PRJEB36683
GCA 040325825	PDT002230876.1	Klebsiella_-	PDS000166495.5	2018	Saudi Arabia	PRJNA1107218
GCA 040325855	PDT002230877.1	Klebsiella_-	PDS000166495.5	2018	Saudi Arabia	PRJNA1107218
GCA 033548075	PDT001982219.1	Klebsiella_-	PDS000166495.5	2022	Saudi Arabia	PRJNA1018815
GCA 033799885	PDT001991616.1	Klebsiella_-	PDS000166495.5	2021	Saudi Arabia	PRJNA1018815
GCA 033800185	PDT001991631.1	Klebsiella_-	PDS000166495.5	2021	Saudi Arabia	PRJNA1018815
GCA 022185685	PDT000716605.1	Klebsiella_101	PDS000060649.2	2018	France	PRJEB35890
GCA 043863365	**HBH_3230_17**	Klebsiella_101	PDS000060649.2	2017	Tunisia	PRJNA1160997
GCA 022185745	PDT000716602.1	Klebsiella_101	PDS000060649.2	2018	France	PRJEB35890
GCA 043905465	**HBH_859_21**	Klebsiella_101	PDS000060649.2	2021	Tunisia	PRJNA1160997
GCA 043905585	**HBH_534_22**	Klebsiella_101	PDS000060649.2	2022	Tunisia	PRJNA1160997
GCA 043863515	**HBH_2052_18**	Klebsiella_101	PDS000060649.2	2018	Tunisia	PRJNA1160997
GCA 043904785	**HBH_2493_18**	Klebsiella_101	PDS000060649.2	2018	Tunisia	PRJNA1160997
GCA 043863555	**HBH_2343_18**	Klebsiella_101	PDS000060649.2	2018	Tunisia	PRJNA1160997
GCA 956479465	PDT001863965.2	Klebsiella_101	PDS000054005.14	2017	Romania	PRJEB63349
GCA 020576795	PDT001161735.1	Klebsiella_101	PDS000054005.14	2018	Spain	PRJNA718833
GCA 009710345	PDT000637205.1	Klebsiella_101	PDS000054005.14	2018/2019	Romania	PRJNA579879
GCA 009710085	PDT000637192.1	Klebsiella_101	PDS000054005.14	2018/2019	Romania	PRJNA579879
GCA 028560855	PDT001610689.1	Klebsiella_101	PDS000054005.14	2020	Spain	PRJEB57245
GCA 009710385	PDT000637207.1	Klebsiella_101	PDS000054005.14	2018/2019	Romania	PRJNA579879
GCA 009710105	PDT000637193.1	Klebsiella_101	PDS000054005.14	2018/2019	Romania	PRJNA579879
GCA 009710765	PDT000637224.1	Klebsiella_101	PDS000054005.14	2018/2019	Romania	PRJNA579879
GCA 009646285	PDT000626675.1	Klebsiella_101	PDS000054005.14	2018/2019	Romania	PRJNA579879
GCA 009710005	PDT000637189.1	Klebsiella_101	PDS000054005.14	2018/2019	Romania	PRJNA579879
GCA 022122385	PDT001244283.1	Klebsiella_101		2014	United Kingdom	PRJNA788733
GCA 043859795	**HBH_3284_13**	Klebsiella_101		2013	Tunisia	PRJNA1160997
GCA 022290985	PDT000427324.1	Klebsiella_2502	PDS000045324.14	nan	NA	PRJEB10018
GCA 021819665	PDT001219141.1	Klebsiella_101	PDS000045324.14	2018	Germany	PRJEB43549
GCA 018444275	PDT001046631.1	Klebsiella_101		2018	Germany	PRJNA692829
GCA 043860495	**HBH_170_15**	Klebsiella_101	PDS000199426.1	2015	Tunisia	PRJNA1160997
GCA 043863105	**HBH_2082_15**	Klebsiella_101	PDS000199426.1	2015	Tunisia	PRJNA1160997
GCA 043860375	**HBH_4306_14**	Klebsiella_101	PDS000199426.1	2014	Tunisia	PRJNA1160997
GCA 043860455	**HBH_84_15**	Klebsiella_101	PDS000199426.1	2015	Tunisia	PRJNA1160997
GCA 900508885	PDT000370690.1	Klebsiella_101	PDS000045324.14	2013	Turkey	PRJEB10018
GCA 900509165	PDT000370720.1	Klebsiella_101	PDS000045324.14	2013	Turkey	PRJEB10018
GCA 023023525	PDT001289697.1	Klebsiella_-	PDS000045324.14	2018	Turkey	PRJNA789336
GCA 900508875	PDT000370684.1	Klebsiella_101	PDS000045324.14	2014	Turkey	PRJEB10018
GCA 043858225	**HBH_835_11**	Klebsiella_101	PDS000045324.14	2011	Tunisia	PRJNA1160997
GCA 022239805	PDT000441634.1	Klebsiella_101	PDS000045324.14	2012	Germany	PRJEB18621
GCA 022211485	PDT000631361.1	Klebsiella_101	PDS000045324.14	2014	Canada	PRJNA564992
GCA 043976095	PDT002412006.1	Klebsiella_-	PDS000045324.14	2014	Canada	PRJNA855907
GCA 022230705	PDT000445958.1	Klebsiella_101	PDS000045324.14	2014	Canada	PRJNA390933
GCA 900509195	PDT000370721.1	Klebsiella_101	PDS000045328.1	2013	Turkey	PRJEB10018
GCA 900509145	PDT000370717.1	Klebsiella_101		2014	Turkey	PRJEB10018
GCA 900517835	PDT000369898.1	Klebsiella_101	PDS000045324.14	2013	Greece	PRJEB10018
GCA 022290735	PDT000427340.1	Klebsiella_-	PDS000045324.14	nan	NA	PRJEB10018
GCA 019433875	PDT001101052.1	Klebsiella_101		2018	Iran	PRJNA749021
GCA 022426945	PDT001256971.1	Klebsiella_101	PDS000080193.2	2015	Turkey	PRJNA809139
GCA 022426965	PDT001256972.1	Klebsiella_101	PDS000080193.2	2015	Turkey	PRJNA809150
GCA 022426985	PDT001256973.1	Klebsiella_101	PDS000080193.2	2015	Turkey	PRJNA809151
GCA 900507645	PDT000370574.1	Klebsiella_101	PDS000045324.14	2014	Turkey	PRJEB10018
GCA 023023305	PDT001289727.1	Klebsiella_101	PDS000045324.14	2019	Turkey	PRJNA789336
GCA 020120115	PDT001137084.1	Klebsiella_101	PDS000045324.14	2013	Switzerland	PRJNA543274
GCA 022287345	PDT000427831.1	Klebsiella_-	PDS000045324.14	nan	NA	PRJEB10018
GCA 022287225	PDT000427835.1	Klebsiella_-	PDS000045324.14	nan	NA	PRJEB10018
GCA 900508185	PDT000370728.1	Klebsiella_101	PDS000045324.14	2013	Turkey	PRJEB10018
GCA 022287305	PDT000427833.1	Klebsiella_101	PDS000045324.14	nan	NA	PRJEB10018
GCA 022287265	PDT000427836.1	Klebsiella_-	PDS000045324.14	nan	NA	PRJEB10018
GCA 022287245	PDT000427837.1	Klebsiella_101	PDS000045324.14	nan	NA	PRJEB10018
GCA 900508585	PDT000370639.1	Klebsiella_101	PDS000045324.14	2014	Turkey	PRJEB10018
GCA 900508775	PDT000370667.1	Klebsiella_101	PDS000045324.14	2014	Turkey	PRJEB10018
GCA 040270895	PDT002227167.1	Klebsiella_101	PDS000045324.14	2015	United Kingdom	PRJNA1123654
GCA 022100675	PDT001243870.1	Klebsiella_101	PDS000045324.14	2014	United Kingdom	PRJNA788733
GCA 022237435	PDT000441697.1	Klebsiella_101	PDS000045324.14	2013	Germany	PRJEB18621
GCA 035017105	PDT002045095.1	Klebsiella_101	PDS000045324.14	2022	Libya	PRJNA998204
GCA 035017425	PDT002045110.1	Klebsiella_101	PDS000045324.14	2022	Libya	PRJNA998204
GCA 020116295	PDT001137019.1	Klebsiella_101	PDS000104479.25	2013	Switzerland	PRJNA543274
GCA 020120475	PDT001137102.1	Klebsiella_101	PDS000104479.25	2013	Switzerland	PRJNA543274
GCA 900505505	PDT000370447.1	Klebsiella_101	PDS000104479.25	2014	Serbia	PRJEB10018
GCA 900505525	PDT000370449.1	Klebsiella_101	PDS000104479.25	2014	Serbia	PRJEB10018
GCA 900505535	PDT000370450.1	Klebsiella_101	PDS000104479.25	2014	Serbia	PRJEB10018
GCA 900505485	PDT000370448.1	Klebsiella_101	PDS000104479.25	2014	Serbia	PRJEB10018
GCA 956476255	PDT001863970.2	Klebsiella_101	PDS000104479.25	2016	Serbia	PRJEB63349
GCA 022234385	PDT000442763.1	Klebsiella_-	PDS000104479.25	2016	Serbia	PRJNA449293
GCA 022234645	PDT000442748.1	Klebsiella_101	PDS000104479.25	2015	Serbia	PRJNA449293
GCA 033865285	PDT001992447.1	Klebsiella_101	PDS000104479.25	2016	Canada	PRJNA532924
GCA 022235005	PDT000442736.1	Klebsiella_101	PDS000104479.25	2016	Serbia	PRJNA449293
GCA 022234805	PDT000442744.1	Klebsiella_101	PDS000104479.25	2015	Serbia	PRJNA449293
GCA 022234395	PDT000442759.1	Klebsiella_101	PDS000104479.25	2015	Serbia	PRJNA449293
GCA 022234465	PDT000442753.1	Klebsiella_101	PDS000104479.25	2016	Serbia	PRJNA449293
GCA 022234325	PDT000442762.1	Klebsiella_-	PDS000104479.25	2015	Serbia	PRJNA449293
GCA 900505315	PDT000370421.1	Klebsiella_101	PDS000104479.25	2014	Serbia	PRJEB10018
GCA 900505595	PDT000370458.1	Klebsiella_101	PDS000104479.25	2014	Serbia	PRJEB10018
GCA 900505555	PDT000370456.1	Klebsiella_101	PDS000104479.25	2014	Serbia	PRJEB10018
GCA 900503565	PDT000370439.1	Klebsiella_101	PDS000104479.25	2013	Serbia	PRJEB10018
GCA 900505275	PDT000370412.1	Klebsiella_101	PDS000104479.25	2013	Serbia	PRJEB10018
GCA 900505255	PDT000370411.1	Klebsiella_101	PDS000104479.25	2014	Serbia	PRJEB10018
GCA 900505445	PDT000370441.1	Klebsiella_101	PDS000104479.25	2014	Serbia	PRJEB10018
GCA 900503505	PDT000370423.1	Klebsiella_101	PDS000104479.25	2014	Serbia	PRJEB10018
GCA 900505145	PDT000370398.1	Klebsiella_101	PDS000104479.25	2013	Serbia	PRJEB10018
GCA 900505195	PDT000370399.1	Klebsiella_101	PDS000104479.25	2013	Serbia	PRJEB10018
GCA 900505065	PDT000370390.1	Klebsiella_101	PDS000104479.25	2014	Serbia	PRJEB10018
GCA 902155925	PDT000663180.1	Klebsiella_101	PDS000104479.25	2014	Serbia	PRJEB33308
GCA 020115935	PDT001137010.1	Klebsiella_101	PDS000104479.25	2014	Switzerland	PRJNA543274
GCA 006459145	PDT000532615.1	Klebsiella_101	PDS000104479.25	nan	NA	PRJNA549322
GCA 900507115	PDT000370462.1	Klebsiella_101	PDS000104479.25	2014	Serbia	PRJEB10018
GCA 900505135	PDT000370392.1	Klebsiella_101	PDS000104479.25	2014	Serbia	PRJEB10018
GCA 900505095	PDT000370391.1	Klebsiella_101	PDS000104479.25	2014	Serbia	PRJEB10018
GCA 900505435	PDT000370437.1	Klebsiella_101	PDS000104479.25	2014	Serbia	PRJEB10018
GCA 900505125	PDT000370397.1	Klebsiella_101	PDS000104479.25	2014	Serbia	PRJEB10018
GCA 900507195	PDT000370466.1	Klebsiella_101	PDS000104479.25	2014	Serbia	PRJEB10018
GCA 900502985	PDT000370139.1	Klebsiella_101	PDS000104479.25	2013	Slovenia	PRJEB10018
GCA 900503095	PDT000370151.1	Klebsiella_101	PDS000104479.25	2013	Slovenia	PRJEB10018
GCA 022234105	PDT000442775.1	Klebsiella_101	PDS000104479.25	2017	Serbia	PRJNA449293
GCA 956478885	PDT001863982.2	Klebsiella_101	PDS000104479.25	2017	Serbia	PRJEB63349
GCA 022759985	PDT001277612.1	Klebsiella_101	PDS000104479.25	2013	Switzerland	PRJNA769291
GCA 022759945	PDT001277610.1	Klebsiella_101	PDS000104479.25	2013	Switzerland	PRJNA769291
GCA 022759925	PDT001277609.1	Klebsiella_101	PDS000104479.25	2013	Switzerland	PRJNA769291
GCA 022759965	PDT001277611.1	Klebsiella_101	PDS000104479.25	2013	Switzerland	PRJNA769291
GCA 022760005	PDT001277613.1	Klebsiella_101	PDS000104479.25	2013	Switzerland	PRJNA769291

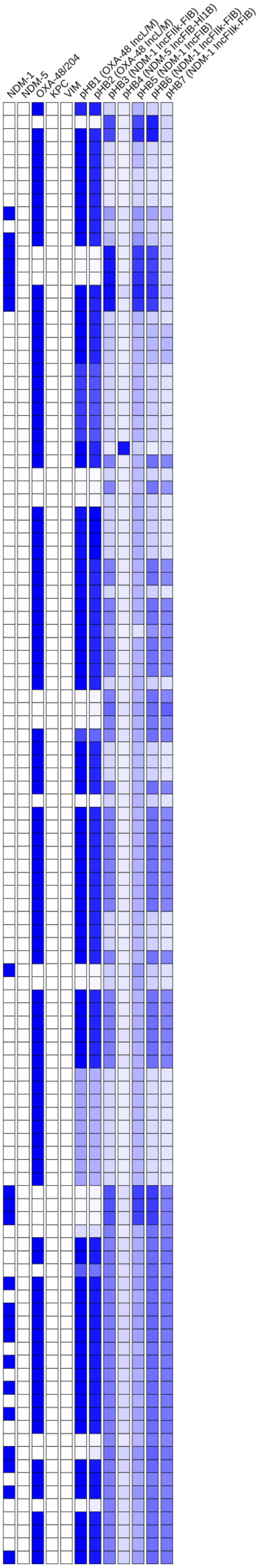

Tree 38: GCA_043905605

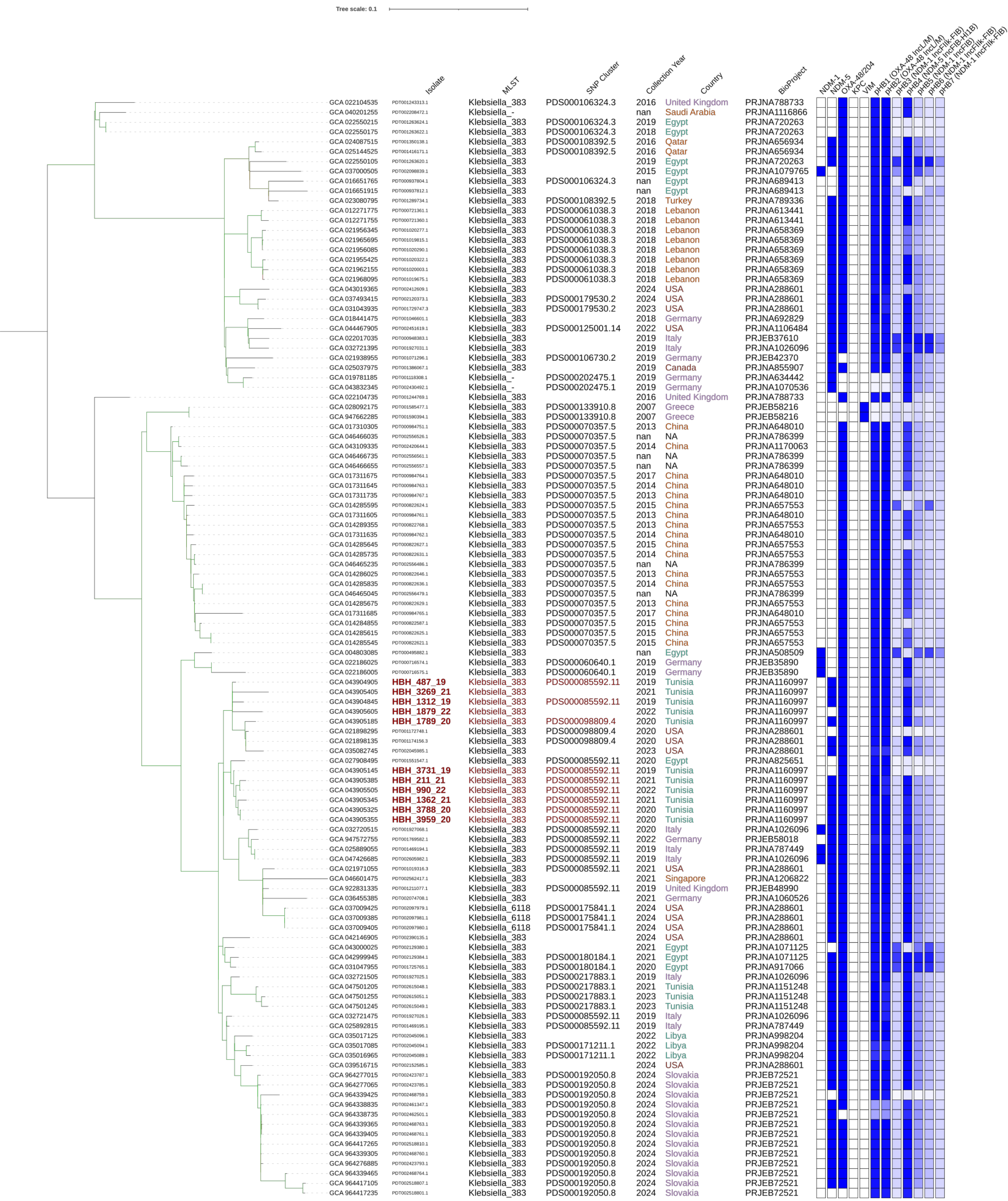

Tree 39: GCA_043905625

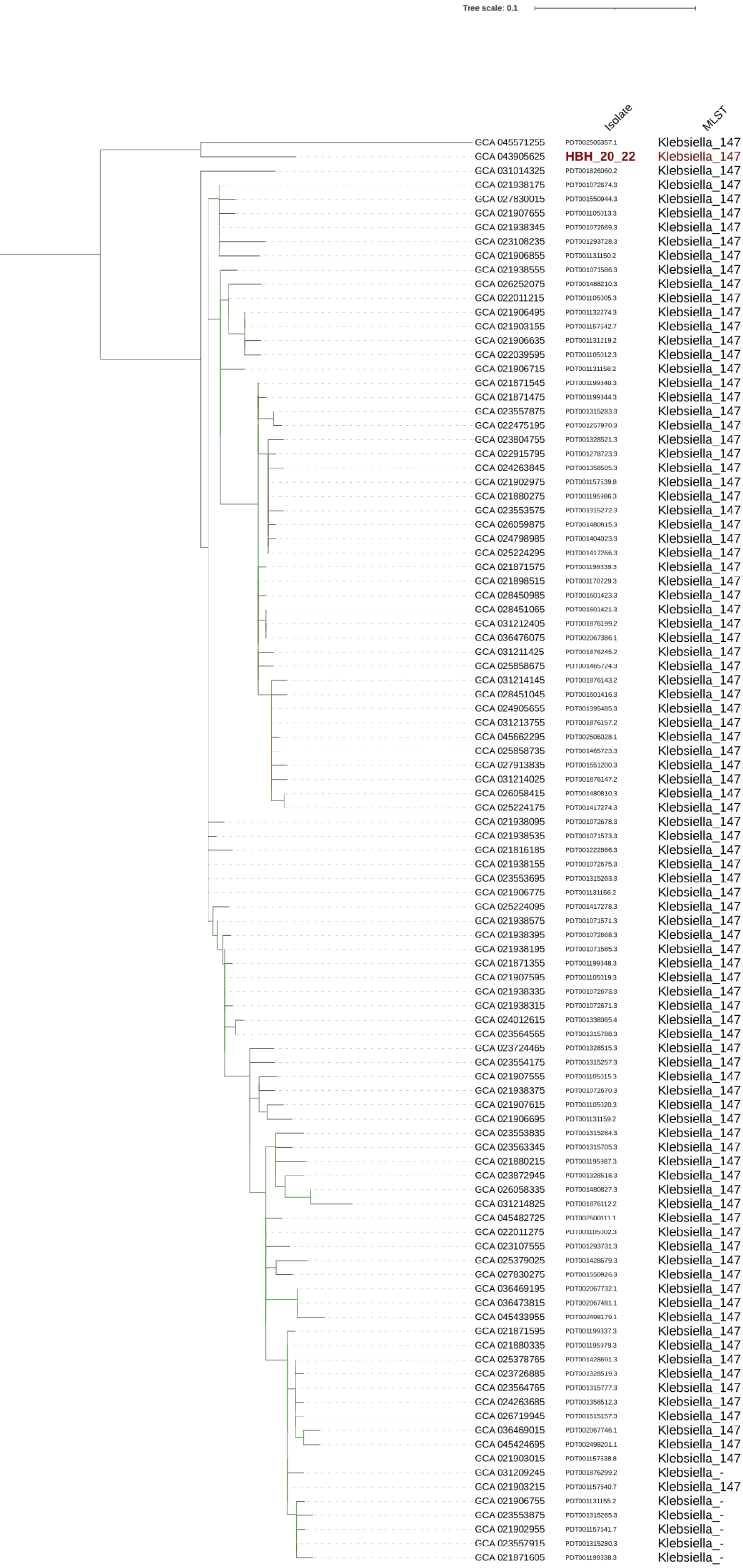

	Isolate	MLST	SNP Cluster	Collection Year	Country	BioProject
GCA 045571255	PDT002505357.1	Klebsiella_147	PDS000210545.1	2024	USA	PRJNA288601
GCA 043905625	**HBH_20_22**	Klebsiella_147	PDS000199417.1	2022	Tunisia	PRJNA1160997
GCA 031014325	PDT001826060.2	Klebsiella_147	PDS000091501.176	2023	USA	PRJNA288601
GCA 021938175	PDT001072674.3	Klebsiella_147	PDS000091501.176	2021	USA	PRJNA288601
GCA 027830015	PDT001550944.3	Klebsiella_147	PDS000091501.176	2022	USA	PRJNA288601
GCA 021907655	PDT001105013.3	Klebsiella_147	PDS000091501.176	2021	USA	PRJNA288601
GCA 021938345	PDT001072669.3	Klebsiella_147	PDS000091501.176	2021	USA	PRJNA288601
GCA 023108235	PDT001293728.3	Klebsiella_147	PDS000091501.176	2021	USA	PRJNA288601
GCA 021906855	PDT001131150.2	Klebsiella_147	PDS000091501.176	2021	USA	PRJNA288601
GCA 021938555	PDT001071586.3	Klebsiella_147	PDS000091501.176	2021	USA	PRJNA288601
GCA 026252075	PDT001488210.3	Klebsiella_147	PDS000091501.176	2022	USA	PRJNA288601
GCA 022011215	PDT001105005.3	Klebsiella_147	PDS000091501.176	2021	USA	PRJNA288601
GCA 021906495	PDT001132274.3	Klebsiella_147	PDS000091501.176	2021	USA	PRJNA288601
GCA 021903155	PDT001157542.7	Klebsiella_147	PDS000091501.176	2021	USA	PRJNA288601
GCA 021906635	PDT001131219.2	Klebsiella_147	PDS000091501.176	2021	USA	PRJNA288601
GCA 022039595	PDT001105012.3	Klebsiella_147	PDS000091501.176	2021	USA	PRJNA288601
GCA 021906715	PDT001131158.2	Klebsiella_147	PDS000091501.176	2021	USA	PRJNA288601
GCA 021871545	PDT001199340.3	Klebsiella_147	PDS000091501.176	2021	USA	PRJNA288601
GCA 021871475	PDT001199344.3	Klebsiella_147	PDS000091501.176	2021	USA	PRJNA288601
GCA 023557875	PDT001315283.3	Klebsiella_147	PDS000091501.176	2021	USA	PRJNA288601
GCA 022475195	PDT001257970.3	Klebsiella_147	PDS000091501.176	2022	USA	PRJNA288601
GCA 023804755	PDT001328521.3	Klebsiella_147	PDS000091501.176	2022	USA	PRJNA288601
GCA 022915795	PDT001278723.3	Klebsiella_147	PDS000091501.176	2022	USA	PRJNA288601
GCA 024263845	PDT001358505.3	Klebsiella_147	PDS000091501.176	2022	USA	PRJNA288601
GCA 021902975	PDT001157539.8	Klebsiella_147	PDS000091501.176	2021	USA	PRJNA288601
GCA 021880275	PDT001195986.3	Klebsiella_147	PDS000091501.176	2021	USA	PRJNA288601
GCA 023553575	PDT001315272.3	Klebsiella_147	PDS000091501.176	2022	USA	PRJNA288601
GCA 026059875	PDT001480815.3	Klebsiella_147	PDS000091501.176	2022	USA	PRJNA288601
GCA 024798985	PDT001404023.3	Klebsiella_147	PDS000091501.176	2022	USA	PRJNA288601
GCA 025224295	PDT001417266.3	Klebsiella_147	PDS000091501.176	2022	USA	PRJNA288601
GCA 021871575	PDT001199339.3	Klebsiella_147	PDS000091501.176	2021	USA	PRJNA288601
GCA 021898515	PDT001170229.3	Klebsiella_147	PDS000091501.176	2021	USA	PRJNA288601
GCA 028450985	PDT001601423.3	Klebsiella_147	PDS000091501.176	2022	USA	PRJNA288601
GCA 028451065	PDT001601421.3	Klebsiella_147	PDS000091501.176	2022	USA	PRJNA288601
GCA 031212405	PDT001876199.2	Klebsiella_147	PDS000091501.176	2023	USA	PRJNA288601
GCA 036476075	PDT002067386.1	Klebsiella_147	PDS000091501.176	2023	USA	PRJNA288601
GCA 031211425	PDT001876245.2	Klebsiella_147	PDS000091501.176	2023	USA	PRJNA288601
GCA 025858675	PDT001465724.3	Klebsiella_147	PDS000091501.176	2022	USA	PRJNA288601
GCA 031214145	PDT001876143.2	Klebsiella_147	PDS000091501.176	2023	USA	PRJNA288601
GCA 028451045	PDT001601416.3	Klebsiella_147	PDS000091501.176	2022	USA	PRJNA288601
GCA 024905655	PDT001395485.3	Klebsiella_147	PDS000091501.176	2022	USA	PRJNA288601
GCA 031213755	PDT001876157.2	Klebsiella_147	PDS000091501.176	2023	USA	PRJNA288601
GCA 045662295	PDT002506028.1	Klebsiella_147	PDS000091501.176	2024	USA	PRJNA288601
GCA 025858735	PDT001465723.3	Klebsiella_147	PDS000091501.176	2022	USA	PRJNA288601
GCA 027913835	PDT001551200.3	Klebsiella_147	PDS000091501.176	2022	USA	PRJNA288601
GCA 031214025	PDT001876147.2	Klebsiella_147	PDS000091501.176	2022	USA	PRJNA288601
GCA 026058415	PDT001480810.3	Klebsiella_147	PDS000091501.176	2022	USA	PRJNA288601
GCA 025224175	PDT001417274.3	Klebsiella_147	PDS000091501.176	2022	USA	PRJNA288601
GCA 021938095	PDT001072678.3	Klebsiella_147	PDS000091501.176	2021	USA	PRJNA288601
GCA 021938535	PDT001071573.3	Klebsiella_147	PDS000091501.176	2020	USA	PRJNA288601
GCA 021816185	PDT001222666.3	Klebsiella_147	PDS000091501.176	2021	USA	PRJNA288601
GCA 021938155	PDT001072675.3	Klebsiella_147	PDS000091501.176	2021	USA	PRJNA288601
GCA 023553695	PDT001315263.3	Klebsiella_147	PDS000091501.176	2022	USA	PRJNA288601
GCA 021906775	PDT001131156.2	Klebsiella_147	PDS000091501.176	2021	USA	PRJNA288601
GCA 025224095	PDT001417278.3	Klebsiella_147	PDS000091501.176	2022	USA	PRJNA288601
GCA 021938575	PDT001071571.3	Klebsiella_147	PDS000091501.176	2021	USA	PRJNA288601
GCA 021938395	PDT001072668.3	Klebsiella_147	PDS000091501.176	2021	USA	PRJNA288601
GCA 021938195	PDT001071585.3	Klebsiella_147	PDS000091501.176	2021	USA	PRJNA288601
GCA 021871355	PDT001199348.3	Klebsiella_147	PDS000091501.176	2021	USA	PRJNA288601
GCA 021907595	PDT001105019.3	Klebsiella_147	PDS000091501.176	2021	USA	PRJNA288601
GCA 021938335	PDT001072673.3	Klebsiella_147	PDS000091501.176	2021	USA	PRJNA288601
GCA 021938315	PDT001072671.3	Klebsiella_147	PDS000091501.176	2021	USA	PRJNA288601
GCA 024012615	PDT001338065.4	Klebsiella_147	PDS000091501.176	2022	USA	PRJNA288601
GCA 023564565	PDT001315788.3	Klebsiella_147	PDS000091501.176	2022	USA	PRJNA288601
GCA 023724465	PDT001328515.3	Klebsiella_147	PDS000091501.176	2022	USA	PRJNA288601
GCA 023554175	PDT001315257.3	Klebsiella_147	PDS000091501.176	2022	USA	PRJNA288601
GCA 021907555	PDT001105015.3	Klebsiella_147	PDS000091501.176	2021	USA	PRJNA288601
GCA 021938375	PDT001072670.3	Klebsiella_147	PDS000091501.176	2021	USA	PRJNA288601
GCA 021907615	PDT001105020.3	Klebsiella_147	PDS000091501.176	2021	USA	PRJNA288601
GCA 021906695	PDT001131159.2	Klebsiella_147	PDS000091501.176	2021	USA	PRJNA288601
GCA 023553835	PDT001315284.3	Klebsiella_147	PDS000091501.176	2021	USA	PRJNA288601
GCA 023563345	PDT001315705.3	Klebsiella_147	PDS000091501.176	2022	USA	PRJNA288601
GCA 021880215	PDT001195987.3	Klebsiella_147	PDS000091501.176	2021	USA	PRJNA288601
GCA 023872945	PDT001328518.3	Klebsiella_147	PDS000091501.176	2022	USA	PRJNA288601
GCA 026058335	PDT001480827.3	Klebsiella_147	PDS000091501.176	2022	USA	PRJNA288601
GCA 031214825	PDT001876112.2	Klebsiella_147	PDS000091501.176	2023	USA	PRJNA288601
GCA 045482725	PDT002500111.1	Klebsiella_147	PDS000091501.176	2024	USA	PRJNA288601
GCA 022011275	PDT001105002.3	Klebsiella_147	PDS000091501.176	2021	USA	PRJNA288601
GCA 023107555	PDT001293731.3	Klebsiella_147	PDS000091501.176	2021	USA	PRJNA288601
GCA 025379025	PDT001428679.3	Klebsiella_147	PDS000091501.176	2022	USA	PRJNA288601
GCA 027830275	PDT001550926.3	Klebsiella_147	PDS000091501.176	2022	USA	PRJNA288601
GCA 036469195	PDT002067732.1	Klebsiella_147	PDS000091501.176	2023	USA	PRJNA288601
GCA 036473815	PDT002067481.1	Klebsiella_147	PDS000091501.176	2023	USA	PRJNA288601
GCA 045433955	PDT002498179.1	Klebsiella_147	PDS000091501.176	2024	USA	PRJNA288601
GCA 021871595	PDT001199337.3	Klebsiella_147	PDS000091501.176	2021	USA	PRJNA288601
GCA 021880335	PDT001195979.3	Klebsiella_147	PDS000091501.176	2021	USA	PRJNA288601
GCA 025378765	PDT001428691.3	Klebsiella_147	PDS000091501.176	2022	USA	PRJNA288601
GCA 023726885	PDT001328519.3	Klebsiella_147	PDS000091501.176	2022	USA	PRJNA288601
GCA 023564765	PDT001315777.3	Klebsiella_147	PDS000091501.176	2022	USA	PRJNA288601
GCA 024263685	PDT001358512.3	Klebsiella_147	PDS000091501.176	2022	USA	PRJNA288601
GCA 026719945	PDT001515157.3	Klebsiella_147	PDS000091501.176	2022	USA	PRJNA288601
GCA 036469015	PDT002067746.1	Klebsiella_147	PDS000091501.176	2023	USA	PRJNA288601
GCA 045424695	PDT002498201.1	Klebsiella_147	PDS000091501.176	2024	USA	PRJNA288601
GCA 021903015	PDT001157538.8	Klebsiella_147	PDS000091501.176	2021	USA	PRJNA288601
GCA 031209245	PDT001876299.2	Klebsiella_-	PDS000091501.176	2023	USA	PRJNA288601
GCA 021903215	PDT001157540.7	Klebsiella_147	PDS000091501.176	2021	USA	PRJNA288601
GCA 021906755	PDT001131155.2	Klebsiella_-	PDS000091501.176	2021	USA	PRJNA288601
GCA 023553875	PDT001315265.3	Klebsiella_-	PDS000091501.176	2022	USA	PRJNA288601
GCA 021902955	PDT001157541.7	Klebsiella_-	PDS000091501.176	2021	USA	PRJNA288601
GCA 023557915	PDT001315280.3	Klebsiella_-	PDS000091501.176	2022	USA	PRJNA288601
GCA 021871605	PDT001199338.3	Klebsiella_-	PDS000091501.176	2021	USA	PRJNA288601

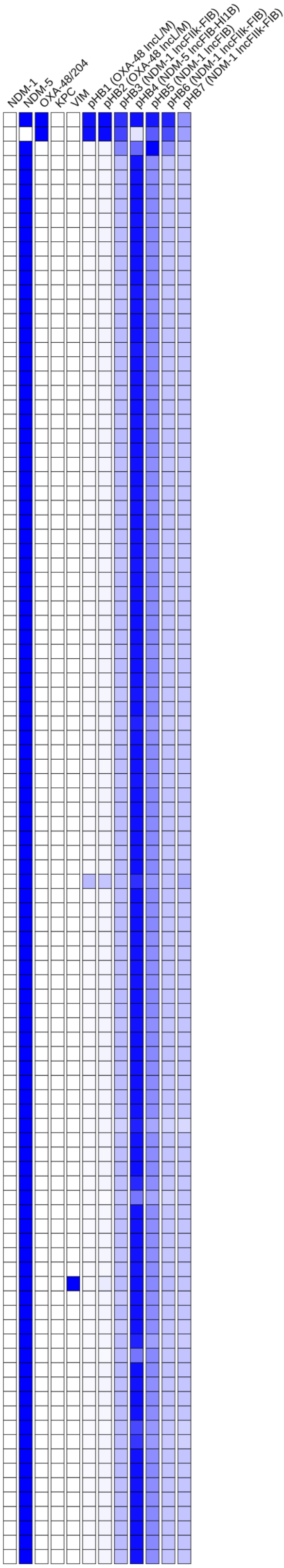

Tree 40: GCA_043905645

Tree scale: 0.1

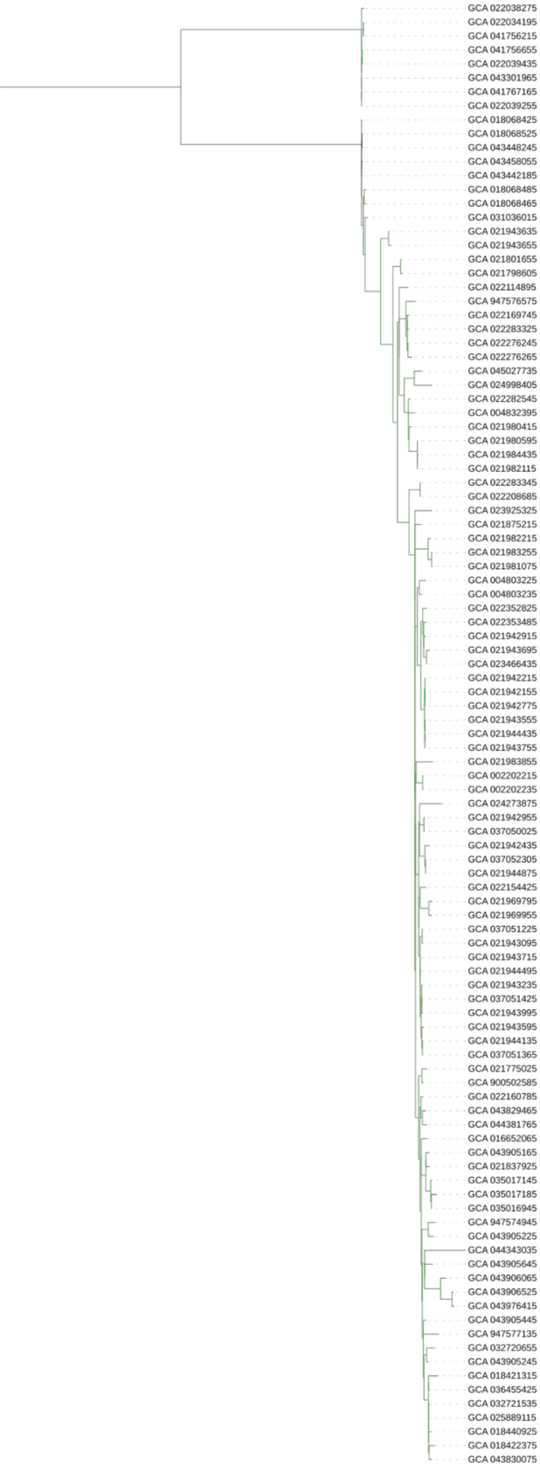

	Isolate	MLST	SNP Cluster	Collection Year	Country	BioProject
GCA 022038275	PDT000893789.3	Klebsiella_4843	PDS000052089.184	2019	USA	PRJNA288601
GCA 022034195	PDT000899437.1	Klebsiella_4843	PDS000052089.184	2019	USA	PRJNA288601
GCA 041756215	PDT002363661.1	Klebsiella_4843	PDS000052089.184	2019	USA	PRJNA288601
GCA 041756655	PDT002363660.1	Klebsiella_4843	PDS000052089.184	2019	USA	PRJNA288601
GCA 022039435	PDT000893652.1	Klebsiella_4843	PDS000052089.184	2019	USA	PRJNA288601
GCA 043301965	PDT002265360.1	Klebsiella_4843	PDS000052089.184	2018	USA	PRJNA1106484
GCA 041767165	PDT002364337.1	Klebsiella_4843	PDS000052089.184	2018	USA	PRJNA288601
GCA 022039255	PDT000893665.3	Klebsiella_4843	PDS000052089.184	2019	USA	PRJNA288601
GCA 018068425	PDT001009219.1	Klebsiella_147	PDS000052089.184	2019	USA	PRJNA288601
GCA 018068525	PDT001009222.1	Klebsiella_147	PDS000052089.184	2019	USA	PRJNA288601
GCA 043448245	PDT002421413.1	Klebsiella_147	PDS000052089.184	2019	USA	PRJNA288601
GCA 043458055	PDT002421633.1	Klebsiella_147	PDS000052089.184	2019	USA	PRJNA288601
GCA 043442185	PDT002421417.1	Klebsiella_147	PDS000052089.184	2019	USA	PRJNA288601
GCA 018068485	PDT001009221.1	Klebsiella_147	PDS000052089.184	2019	USA	PRJNA288601
GCA 018068465	PDT001009220.1	Klebsiella_147	PDS000052089.184	2019	USA	PRJNA288601
GCA 031036015	PDT001726355.3	Klebsiella_147	PDS000052089.184	2018	USA	PRJNA288601
GCA 021943635	PDT001042195.1	Klebsiella_147	PDS000052089.184	2016	USA	PRJNA725484
GCA 021943655	PDT001042194.1	Klebsiella_147	PDS000052089.184	2016	Germany	PRJNA725484
GCA 021801655	PDT001227339.1	Klebsiella_147	PDS000156049.11	2014	United Kingdom	PRJNA788733
GCA 021798605	PDT001227461.1	Klebsiella_147	PDS000156049.11	2014	United Kingdom	PRJNA788733
GCA 022114895	PDT001245144.1	Klebsiella_147	PDS000156049.11	2014	United Kingdom	PRJNA788733
GCA 947576575	PDT001769496.1	Klebsiella_147	PDS000156049.11	2022	Germany	PRJEB58018
GCA 022169745	PDT000847257.1	Klebsiella_147	PDS000156049.11	2018	Myanmar	PRJDB5126
GCA 022283325	PDT000431873.1	Klebsiella_147	PDS000156049.11	2016	Myanmar	PRJDB5126
GCA 022276245	PDT000432066.1	Klebsiella_147	PDS000156049.11	2015	Myanmar	PRJDB5126
GCA 022276265	PDT000432048.1	Klebsiella_147	PDS000156049.11	2016	Myanmar	PRJDB5126
GCA 045027735	PDT002474493.1	Klebsiella_147	PDS000156049.11	2017	India	PRJNA939406
GCA 024998405	PDT001412313.1	Klebsiella_147	PDS000156049.11	2020	India	PRJNA738274
GCA 022282545	PDT000431951.1	Klebsiella_147	PDS000156049.11	2016	Thailand	PRJDB5929
GCA 004832395	PDT000495993.1	Klebsiella_147	PDS000156049.11	2018	Thailand	PRJNA532291
GCA 021980415	PDT001008159.1	Klebsiella_147	PDS000156049.11	2016	Thailand	PRJDB5929
GCA 021980595	PDT001008111.1	Klebsiella_147	PDS000156049.11	2017	Thailand	PRJDB5929
GCA 021984435	PDT001003302.1	Klebsiella_147	PDS000156049.11	2017	Thailand	PRJDB5929
GCA 021982115	PDT001008059.1	Klebsiella_147	PDS000156049.11	2016	Thailand	PRJDB5929
GCA 022283345	PDT000431869.1	Klebsiella_147	PDS000156049.11	2016	Myanmar	PRJDB5126
GCA 022208685	PDT000650074.1	Klebsiella_147	PDS000156049.11	2016	Myanmar	PRJDB8975
GCA 023925325	PDT001345521.1	Klebsiella_147	PDS000106697.6	2019	Israel	PRJEB53419
GCA 021875215	PDT001197766.1	Klebsiella_147	PDS000156049.11	2013	Singapore	PRJNA577535
GCA 021982215	PDT001008055.1	Klebsiella_147	PDS000056180.7	2016	Thailand	PRJDB5929
GCA 021983255	PDT001003383.1	Klebsiella_147	PDS000056180.7	2016	Thailand	PRJDB5929
GCA 021981075	PDT001008160.1	Klebsiella_147	PDS000056180.7	2016	Thailand	PRJDB5929
GCA 004803225	PDT000495886.1	Klebsiella_147	PDS000077015.34	nan	Egypt	PRJNA508509
GCA 004803235	PDT000495887.1	Klebsiella_147	PDS000077015.34	nan	Egypt	PRJNA508509
GCA 022352825	PDT001118267.2	Klebsiella_147	PDS000077015.34	2019	Italy	PRJNA643814
GCA 022353485	PDT001118270.2	Klebsiella_147	PDS000077015.34	2019	Italy	PRJNA643814
GCA 021942915	PDT001042232.1	Klebsiella_147	PDS000077015.34	2019	Italy	PRJNA725484
GCA 021943695	PDT001042193.1	Klebsiella_147	PDS000077015.34	2019	Italy	PRJNA725484
GCA 023466435	PDT000906262.2	Klebsiella_147	PDS000077015.34	2019	Italy	PRJNA643814
GCA 021942215	PDT001042253.1	Klebsiella_147	PDS000077015.34	2019	Italy	PRJNA725484
GCA 021942155	PDT001042257.1	Klebsiella_147	PDS000077015.34	2019	Italy	PRJNA725484
GCA 021942775	PDT001042238.1	Klebsiella_147	PDS000077015.34	2019	Italy	PRJNA725484
GCA 021943555	PDT001042198.1	Klebsiella_147	PDS000077015.34	2020	Italy	PRJNA725484
GCA 021944435	PDT001042156.1	Klebsiella_147	PDS000077015.34	2019	Italy	PRJNA725484
GCA 021943755	PDT001042189.1	Klebsiella_147	PDS000077015.34	2019	Italy	PRJNA725484
GCA 021983855	PDT001003356.1	Klebsiella_147	PDS000104520.1	2016	Thailand	PRJDB5929
GCA 002202215	PDT000219969.1	Klebsiella_147	PDS000156049.11	nan	NA	PRJNA292902
GCA 002202235	PDT000219970.1	Klebsiella_147	PDS000156049.11	nan	NA	PRJNA292902
GCA 024273875	PDT001345527.1	Klebsiella_147	PDS000156049.11	2018	Israel	PRJEB53419
GCA 021942955	PDT001042230.1	Klebsiella_147	PDS000156049.11	nan	USA	PRJNA725484
GCA 037050025	PDT002101976.1	Klebsiella_147	PDS000156049.11	2016	Jordan	PRJNA1076682
GCA 021942435	PDT001042255.1	Klebsiella_147	PDS000156049.11	nan	USA	PRJNA725484
GCA 037052305	PDT002102090.1	Klebsiella_147	PDS000156049.11	2013	Jordan	PRJNA1076682
GCA 021944875	PDT001042137.1	Klebsiella_147	PDS000156049.11	nan	USA	PRJNA725484
GCA 022154425	PDT000858721.1	Klebsiella_147	PDS000156049.11	2017	USA	PRJNA658369
GCA 021969795	PDT001019592.1	Klebsiella_147	PDS000156049.11	2017	USA	PRJNA658369
GCA 021969955	PDT001019579.1	Klebsiella_147	PDS000156049.11	2018	USA	PRJNA658369
GCA 037051225	PDT002102036.1	Klebsiella_147	PDS000156049.11	2017	Jordan	PRJNA1076682
GCA 021943095	PDT001042208.1	Klebsiella_147	PDS000156049.11	nan	USA	PRJNA725484
GCA 021943715	PDT001042191.1	Klebsiella_147	PDS000156049.11	nan	USA	PRJNA725484
GCA 021944495	PDT001042155.1	Klebsiella_147	PDS000156049.11	nan	USA	PRJNA725484
GCA 021943235	PDT001042219.1	Klebsiella_147	PDS000156049.11	nan	USA	PRJNA725484
GCA 037051425	PDT002102046.1	Klebsiella_147	PDS000156049.11	2015	Jordan	PRJNA1076682
GCA 021943995	PDT001042177.1	Klebsiella_147	PDS000156049.11	nan	USA	PRJNA725484
GCA 021943595	PDT001042197.1	Klebsiella_147	PDS000156049.11	nan	USA	PRJNA725484
GCA 021944135	PDT001042166.1	Klebsiella_147	PDS000156049.11	nan	USA	PRJNA725484
GCA 037051365	PDT002102043.1	Klebsiella_147	PDS000156049.11	2015	Jordan	PRJNA1076682
GCA 021775025	PDT001227850.1	Klebsiella_147	PDS000156049.11	2016	United Kingdom	PRJNA788733
GCA 900502585	PDT000370025.1	Klebsiella_147	PDS000156049.11	2014	Denmark	PRJEB10018
GCA 022160785	PDT001243475.1	Klebsiella_147	PDS000156049.11	2014	United Kingdom	PRJNA788733
GCA 043829465	PDT002430473.1	Klebsiella_147	PDS000103185.4	2020	Germany	PRJNA1070536
GCA 044381765	PDT002456479.1	Klebsiella_147	PDS000201444.2	nan	Saudi Arabia	PRJNA1116866
GCA 016652065	PDT000937820.1	Klebsiella_147		nan	Egypt	PRJNA689413
GCA 043905165	**HBH_556_19**	Klebsiella_147	PDS000090167.14	2019	Tunisia	PRJNA1160997
GCA 021837925	PDT001216482.1	Klebsiella_147	PDS000090167.14	2019	Switzerland	PRJNA744003
GCA 035017145	PDT002045097.1	Klebsiella_147	PDS000090167.14	2022	Libya	PRJNA998204
GCA 035017185	PDT002045099.1	Klebsiella_147	PDS000090167.14	2022	Libya	PRJNA998204
GCA 035016945	PDT002045088.1	Klebsiella_147	PDS000090167.14	2022	Libya	PRJNA998204
GCA 947574945	PDT001769393.1	Klebsiella_147	PDS000199427.1	2022	Germany	PRJEB58018
GCA 043905225	**HBH_3009_20**	Klebsiella_147	PDS000199427.1	2020	Tunisia	PRJNA1160997
GCA 044343035	PDT002455478.1	Klebsiella_147	PDS000205531.1	2022	Senegal	PRJNA1073865
GCA 043905645	**HBH_3065_22**	Klebsiella_147		2022	Tunisia	PRJNA1160997
GCA 043906065	PDT002391396.1	Klebsiella_147	PDS000136743.8	2021	Netherlands	PRJNA1143178
GCA 043906525	PDT002391374.1	Klebsiella_147	PDS000136743.8	2023	Netherlands	PRJNA1143178
GCA 043976415	PDT002391348.1	Klebsiella_147	PDS000136743.8	2023	Netherlands	PRJNA1143178
GCA 043905445	**HBH_1520_21**	Klebsiella_147	PDS000090167.14	2021	Tunisia	PRJNA1160997
GCA 947577135	PDT001769510.1	Klebsiella_147	PDS000129332.6	2022	Germany	PRJEB58018
GCA 032720655	PDT001927061.1	Klebsiella_147	PDS000090167.14	2020	Italy	PRJNA1026096
GCA 043905245	**HBH_110_20**	Klebsiella_147	PDS000090167.14	2020	Tunisia	PRJNA1160997
GCA 018421315	PDT001044123.1	Klebsiella_147	PDS000090167.14	2019	Germany	PRJNA692829
GCA 036455425	PDT002074710.1	Klebsiella_147	PDS000090167.14	2020	Germany	PRJNA1060526
GCA 032721535	PDT001927023.1	Klebsiella_147	PDS000090167.14	2019	Italy	PRJNA1026096
GCA 025889115	PDT001469197.1	Klebsiella_147	PDS000090167.14	2019	Italy	PRJNA787449
GCA 018440925	PDT001046592.1	Klebsiella_147	PDS000090167.14	2019	Germany	PRJNA692829
GCA 018422375	PDT001044139.1	Klebsiella_147	PDS000090167.14	2019	Germany	PRJNA692829
GCA 043830075	PDT002430479.1	Klebsiella_147	PDS000090167.14	2019	Germany	PRJNA1070536

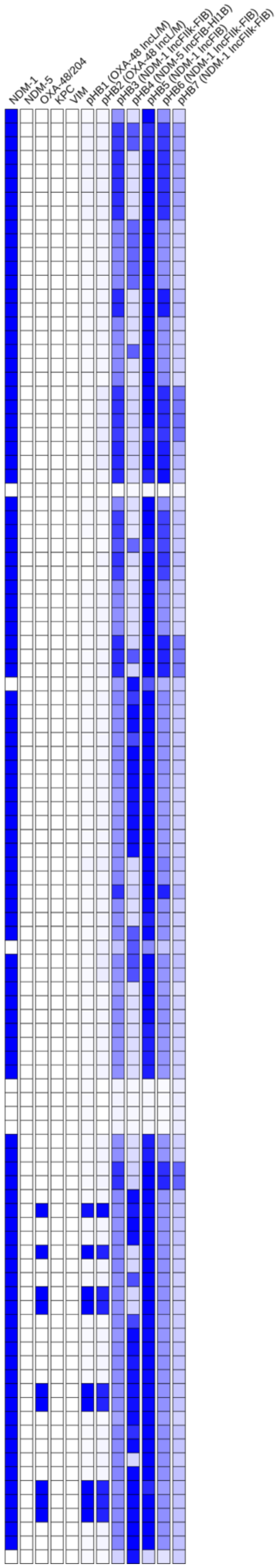

Tree 41: GCA_043905665

Tree scale: 0.01

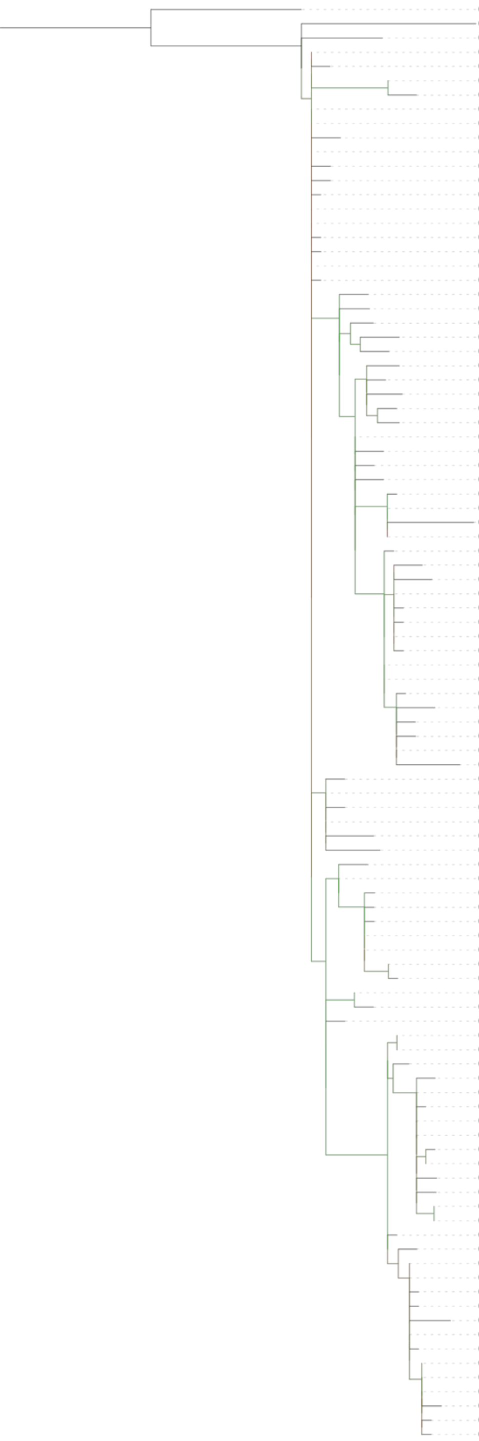

	Isolate	MLST	SNP Cluster	Collection Year	Country	BioProject
GCA 043905665	HBH_2460_22	Klebsiella_147		2022	Tunisia	PRJNA1160997
GCA 033800165	PDT001991630.1	Klebsiella_147	PDS000091501.176	2021	Saudi Arabia	PRJNA1018815
GCA 031014325	PDT001826060.2	Klebsiella_147	PDS000091501.176	2023	USA	PRJNA288601
GCA 021938575	PDT001071571.3	Klebsiella_147	PDS000091501.176	2021	USA	PRJNA288601
GCA 023564565	PDT001315788.3	Klebsiella_147	PDS000091501.176	2022	USA	PRJNA288601
GCA 024263765	PDT001358509.3	Klebsiella_147	PDS000091501.176	2022	USA	PRJNA288601
GCA 026252015	PDT001488211.3	Klebsiella_147	PDS000091501.176	2022	USA	PRJNA288601
GCA 021938335	PDT001072673.3	Klebsiella_147	PDS000091501.176	2021	USA	PRJNA288601
GCA 021938195	PDT001071585.3	Klebsiella_147	PDS000091501.176	2021	USA	PRJNA288601
GCA 021816185	PDT001222666.3	Klebsiella_147	PDS000091501.176	2021	USA	PRJNA288601
GCA 021906775	PDT001131156.2	Klebsiella_147	PDS000091501.176	2021	USA	PRJNA288601
GCA 021938095	PDT001072678.3	Klebsiella_147	PDS000091501.176	2021	USA	PRJNA288601
GCA 025224095	PDT001417278.3	Klebsiella_147	PDS000091501.176	2022	USA	PRJNA288601
GCA 021871355	PDT001199348.3	Klebsiella_147	PDS000091501.176	2021	USA	PRJNA288601
GCA 023553695	PDT001315263.3	Klebsiella_147	PDS000091501.176	2022	USA	PRJNA288601
GCA 021907595	PDT001105019.3	Klebsiella_147	PDS000091501.176	2021	USA	PRJNA288601
GCA 021938535	PDT001071573.3	Klebsiella_147	PDS000091501.176	2020	USA	PRJNA288601
GCA 021938315	PDT001072671.3	Klebsiella_147	PDS000091501.176	2021	USA	PRJNA288601
GCA 021938155	PDT001072675.3	Klebsiella_147	PDS000091501.176	2021	USA	PRJNA288601
GCA 021938395	PDT001072668.3	Klebsiella_147	PDS000091501.176	2021	USA	PRJNA288601
GCA 023724465	PDT001328515.3	Klebsiella_147	PDS000091501.176	2022	USA	PRJNA288601
GCA 023554175	PDT001315257.3	Klebsiella_147	PDS000091501.176	2022	USA	PRJNA288601
GCA 021907555	PDT001105015.3	Klebsiella_147	PDS000091501.176	2021	USA	PRJNA288601
GCA 022039615	PDT001105010.3	Klebsiella_147	PDS000091501.176	2021	USA	PRJNA288601
GCA 021906695	PDT001131159.2	Klebsiella_147	PDS000091501.176	2021	USA	PRJNA288601
GCA 023553835	PDT001315284.3	Klebsiella_147	PDS000091501.176	2021	USA	PRJNA288601
GCA 023563345	PDT001315705.3	Klebsiella_147	PDS000091501.176	2022	USA	PRJNA288601
GCA 021880215	PDT001195987.3	Klebsiella_147	PDS000091501.176	2021	USA	PRJNA288601
GCA 024263905	PDT001358502.3	Klebsiella_147	PDS000091501.176	2022	USA	PRJNA288601
GCA 023872945	PDT001328518.3	Klebsiella_147	PDS000091501.176	2022	USA	PRJNA288601
GCA 022011275	PDT001105002.3	Klebsiella_147	PDS000091501.176	2021	USA	PRJNA288601
GCA 027830275	PDT001550926.3	Klebsiella_147	PDS000091501.176	2022	USA	PRJNA288601
GCA 045482725	PDT002500111.1	Klebsiella_147	PDS000091501.176	2024	USA	PRJNA288601
GCA 023107555	PDT001293731.3	Klebsiella_147	PDS000091501.176	2021	USA	PRJNA288601
GCA 031212625	PDT001876187.2	Klebsiella_147	PDS000091501.176	2023	USA	PRJNA288601
GCA 036473815	PDT002067481.1	Klebsiella_147	PDS000091501.176	2023	USA	PRJNA288601
GCA 045718025	PDT002512538.1	Klebsiella_147	PDS000091501.176	2024	USA	PRJNA288601
GCA 036469195	PDT002067732.1	Klebsiella_147	PDS000091501.176	2023	USA	PRJNA288601
GCA 021871595	PDT001199337.3	Klebsiella_147	PDS000091501.176	2021	USA	PRJNA288601
GCA 036469015	PDT002067746.1	Klebsiella_147	PDS000091501.176	2023	USA	PRJNA288601
GCA 025223535	PDT001417280.3	Klebsiella_147	PDS000091501.176	2022	USA	PRJNA288601
GCA 023564765	PDT001315777.3	Klebsiella_147	PDS000091501.176	2022	USA	PRJNA288601
GCA 024263685	PDT001358512.3	Klebsiella_147	PDS000091501.176	2022	USA	PRJNA288601
GCA 026719945	PDT001515157.3	Klebsiella_147	PDS000091501.176	2022	USA	PRJNA288601
GCA 025378765	PDT001428691.3	Klebsiella_147	PDS000091501.176	2022	USA	PRJNA288601
GCA 023726885	PDT001328519.3	Klebsiella_147	PDS000091501.176	2022	USA	PRJNA288601
GCA 021903015	PDT001157538.8	Klebsiella_147	PDS000091501.176	2021	USA	PRJNA288601
GCA 021903215	PDT001157540.7	Klebsiella_147	PDS000091501.176	2021	USA	PRJNA288601
GCA 021906755	PDT001131155.2	Klebsiella_-	PDS000091501.176	2021	USA	PRJNA288601
GCA 023563665	PDT001315722.3	Klebsiella_-	PDS000091501.176	2022	USA	PRJNA288601
GCA 023553875	PDT001315265.3	Klebsiella_-	PDS000091501.176	2022	USA	PRJNA288601
GCA 021871605	PDT001199338.3	Klebsiella_-	PDS000091501.176	2021	USA	PRJNA288601
GCA 023557915	PDT001315280.3	Klebsiella_-	PDS000091501.176	2022	USA	PRJNA288601
GCA 023726865	PDT001328522.3	Klebsiella_-	PDS000091501.176	2022	USA	PRJNA288601
GCA 021907655	PDT001105013.3	Klebsiella_147	PDS000091501.176	2021	USA	PRJNA288601
GCA 021938175	PDT001072674.3	Klebsiella_147	PDS000091501.176	2021	USA	PRJNA288601
GCA 027830015	PDT001550944.3	Klebsiella_147	PDS000091501.176	2022	USA	PRJNA288601
GCA 021938345	PDT001072669.3	Klebsiella_147	PDS000091501.176	2021	USA	PRJNA288601
GCA 021906855	PDT001131150.2	Klebsiella_147	PDS000091501.176	2021	USA	PRJNA288601
GCA 023108235	PDT001293728.3	Klebsiella_147	PDS000091501.176	2021	USA	PRJNA288601
GCA 023804735	PDT001328524.3	Klebsiella_147	PDS000091501.176	2022	USA	PRJNA288601
GCA 022011215	PDT001105005.3	Klebsiella_147	PDS000091501.176	2021	USA	PRJNA288601
GCA 021906675	PDT001131160.2	Klebsiella_147	PDS000091501.176	2021	USA	PRJNA288601
GCA 022039655	PDT001105006.3	Klebsiella_147	PDS000091501.176	2021	USA	PRJNA288601
GCA 021906895	PDT001131151.2	Klebsiella_147	PDS000091501.176	2021	USA	PRJNA288601
GCA 021906495	PDT001132274.3	Klebsiella_147	PDS000091501.176	2021	USA	PRJNA288601
GCA 021903155	PDT001157542.7	Klebsiella_147	PDS000091501.176	2021	USA	PRJNA288601
GCA 021903135	PDT001157547.7	Klebsiella_147	PDS000091501.176	2021	USA	PRJNA288601
GCA 022039595	PDT001105012.3	Klebsiella_147	PDS000091501.176	2021	USA	PRJNA288601
GCA 021906715	PDT001131158.2	Klebsiella_147	PDS000091501.176	2021	USA	PRJNA288601
GCA 023553895	PDT001315261.3	Klebsiella_147	PDS000091501.176	2022	USA	PRJNA288601
GCA 021938555	PDT001071586.3	Klebsiella_147	PDS000091501.176	2021	USA	PRJNA288601
GCA 031212405	PDT001876199.2	Klebsiella_147	PDS000091501.176	2023	USA	PRJNA288601
GCA 036476075	PDT002067386.1	Klebsiella_147	PDS000091501.176	2023	USA	PRJNA288601
GCA 025858675	PDT001465724.3	Klebsiella_147	PDS000091501.176	2022	USA	PRJNA288601
GCA 028451045	PDT001601416.3	Klebsiella_147	PDS000091501.176	2022	USA	PRJNA288601
GCA 024377975	PDT001366901.3	Klebsiella_147	PDS000091501.176	2022	USA	PRJNA288601
GCA 045662295	PDT002506028.1	Klebsiella_147	PDS000091501.176	2024	USA	PRJNA288601
GCA 024905655	PDT001395485.3	Klebsiella_147	PDS000091501.176	2022	USA	PRJNA288601
GCA 031213755	PDT001876157.2	Klebsiella_147	PDS000091501.176	2023	USA	PRJNA288601
GCA 026252155	PDT001488204.3	Klebsiella_147	PDS000091501.176	2022	USA	PRJNA288601
GCA 025858735	PDT001465723.3	Klebsiella_147	PDS000091501.176	2022	USA	PRJNA288601
GCA 031214145	PDT001876143.2	Klebsiella_147	PDS000091501.176	2023	USA	PRJNA288601
GCA 031214025	PDT001876147.2	Klebsiella_147	PDS000091501.176	2022	USA	PRJNA288601
GCA 025224175	PDT001417274.3	Klebsiella_147	PDS000091501.176	2022	USA	PRJNA288601
GCA 026058415	PDT001480810.3	Klebsiella_147	PDS000091501.176	2022	USA	PRJNA288601
GCA 028450985	PDT001601423.3	Klebsiella_147	PDS000091501.176	2022	USA	PRJNA288601
GCA 031211425	PDT001876245.2	Klebsiella_147	PDS000091501.176	2023	USA	PRJNA288601
GCA 021871545	PDT001199340.3	Klebsiella_147	PDS000091501.176	2021	USA	PRJNA288601
GCA 021898515	PDT001170229.3	Klebsiella_147	PDS000091501.176	2021	USA	PRJNA288601
GCA 021871475	PDT001199344.3	Klebsiella_147	PDS000091501.176	2021	USA	PRJNA288601
GCA 021871575	PDT001199339.3	Klebsiella_147	PDS000091501.176	2021	USA	PRJNA288601
GCA 028451265	PDT001601419.3	Klebsiella_147	PDS000091501.176	2022	USA	PRJNA288601
GCA 023557875	PDT001315283.3	Klebsiella_147	PDS000091501.176	2021	USA	PRJNA288601
GCA 022475195	PDT001257970.3	Klebsiella_147	PDS000091501.176	2022	USA	PRJNA288601
GCA 021902975	PDT001157539.8	Klebsiella_147	PDS000091501.176	2021	USA	PRJNA288601
GCA 021880275	PDT001195986.3	Klebsiella_147	PDS000091501.176	2021	USA	PRJNA288601
GCA 025224295	PDT001417266.3	Klebsiella_147	PDS000091501.176	2022	USA	PRJNA288601
GCA 024263845	PDT001358505.3	Klebsiella_147	PDS000091501.176	2022	USA	PRJNA288601
GCA 022915795	PDT001278723.3	Klebsiella_147	PDS000091501.176	2022	USA	PRJNA288601
GCA 026059875	PDT001480815.3	Klebsiella_147	PDS000091501.176	2022	USA	PRJNA288601

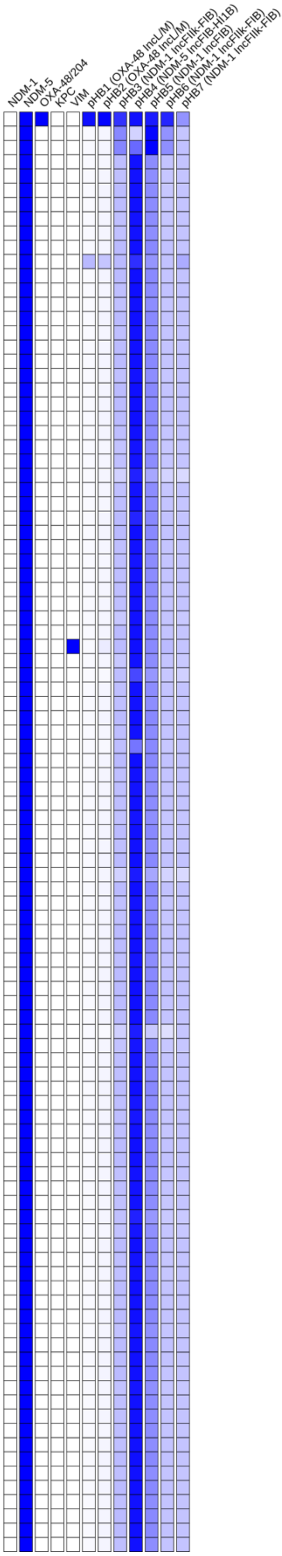

Supplement: Data S1C — Phylogenetic trees (28–41) generated in this study. [file aac.00142-26-s0003.pdf]
