# Supplementary figures and images for: Leveraging the global genomic epidemiology of carbapenemase-producing Klebsiella pneumoniae to inform infection prevention in Tunisian hospitals
Source: Antimicrob Agents Chemother. 2026 May 6;70(6):e00142-26. doi: 10.1128/aac.00142-26 (PMC13231914; doi:10.1128/aac.00142-26)

# Tree 42: MLST383\_top400.raxml.support

Tree scale: 0.1

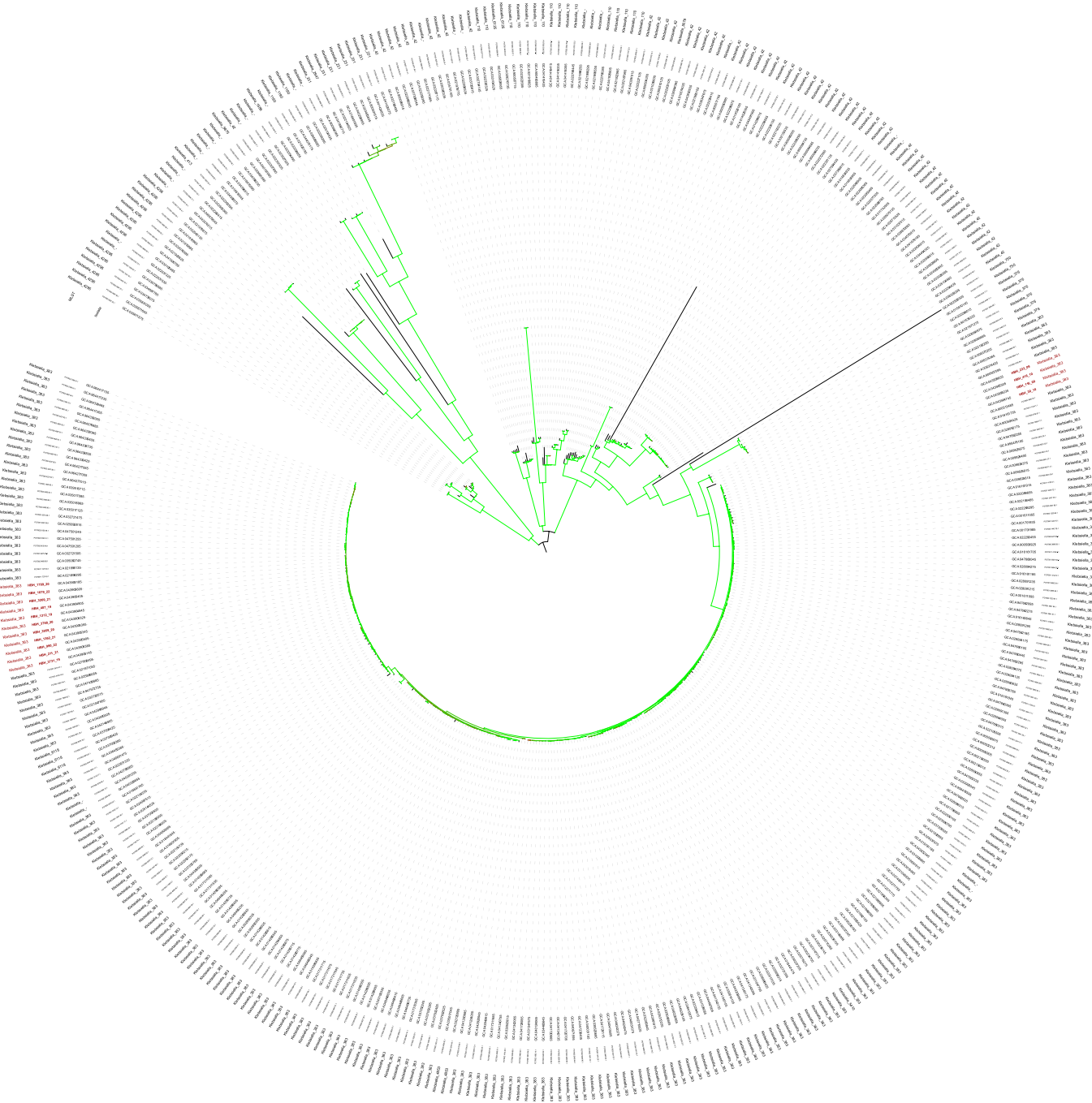

# Tree 43: ST383\_ST376

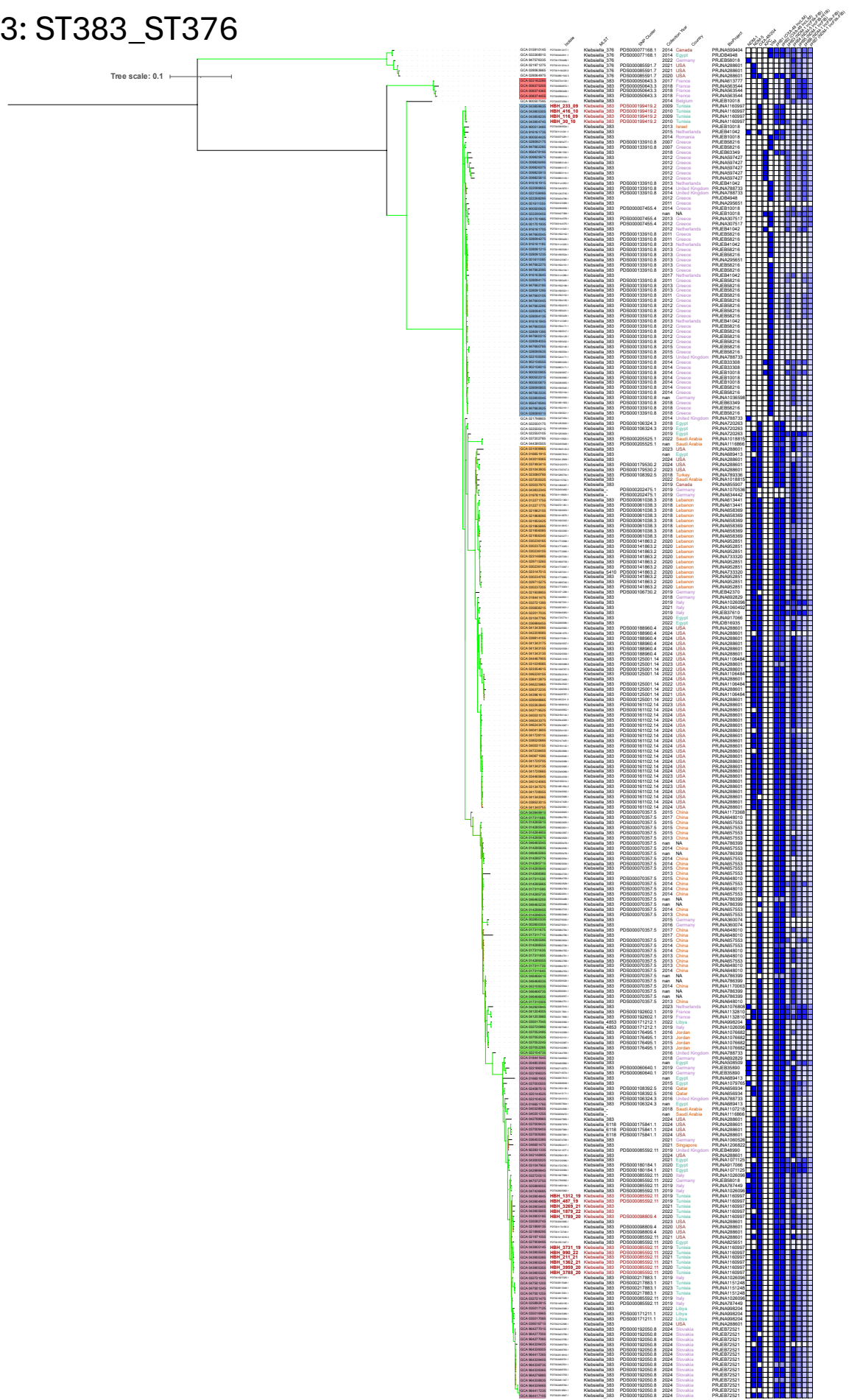

Supplement: Data S1D — Phylogenetic trees (42–43) generated in this study. [file aac.00142-26-s0004.pdf]
