# Supplementary material for: Leveraging the global genomic epidemiology of carbapenemase-producing Klebsiella pneumoniae to inform infection prevention in Tunisian hospitals
Source: Antimicrob Agents Chemother. 2026 May 6;70(6):e00142-26. doi: 10.1128/aac.00142-26 (PMC13231914; doi:10.1128/aac.00142-26)
Supplement: Supplemental material — Fig. S1 to S12; Tables S1 and S2, S4 to S7, and S10. [file aac.00142-26-s0005.pdf]

Supplementary

Leveraging the global genomic epidemiology of carbapenemase-producing *Klebsiella pneumoniae* to inform infection prevention and control in Tunisian hospitals

Basma Menif[1,2,3,4*], Jay N. Worley[3,5], Noura Ben Mansour[1], Faouzia Mahjoubi[1,2], Adnene Hammami[2], Lynn Bry[3,4,6]

Content

Supplementary Figures 2
Figure S1 2
Figure S2 3
Figure S3 4
Figure S4 5
Figure S5 6
Figure S6 7
Figure S7 8
Figure S8 9
Figure S9 10
Figure S10 11
Figure S11 12
Figure S12 13
Supplementary Tables 14
Table S1 14
Table S2 15
Table S3 15
Table S4 16
Table S5 17
Table S6 18
Table S7 19
Table S8 20
Table S9 20
Table S10 21
Table S11 24
Supplementary Trees (Supplementary Data 1 A-D) 24
Supplementary References 25

Supplementary Figures

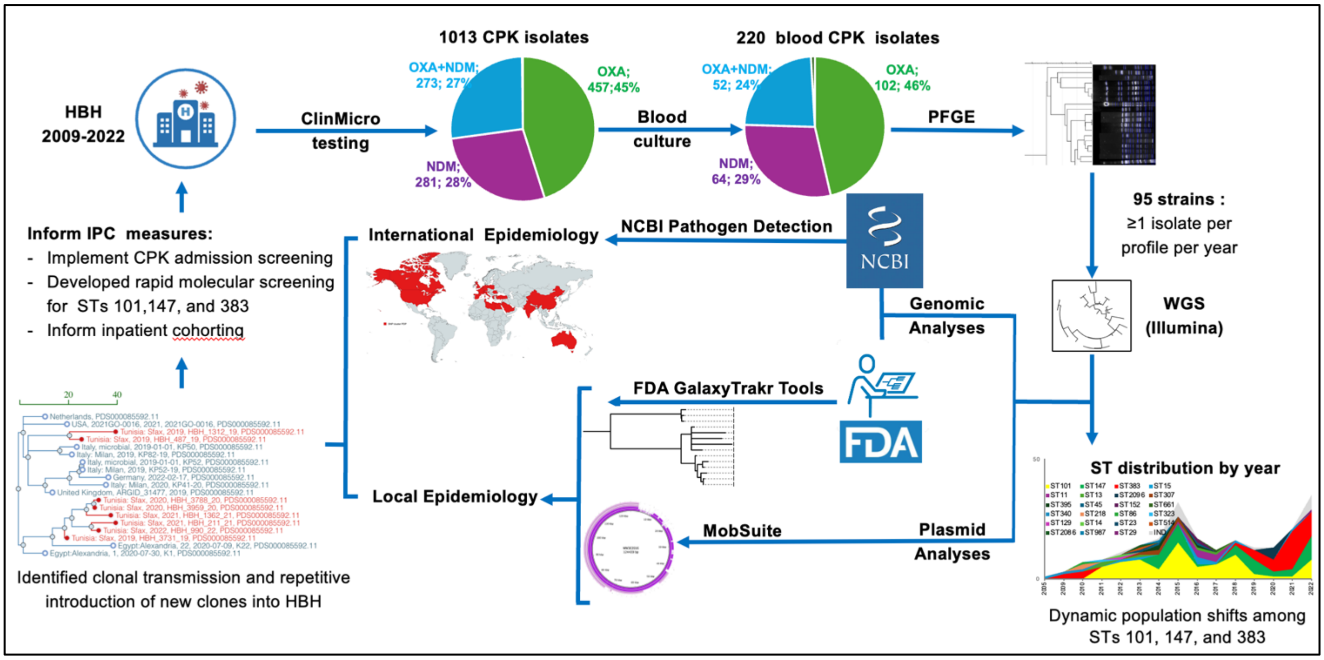

Fig. S1: Study flowchart

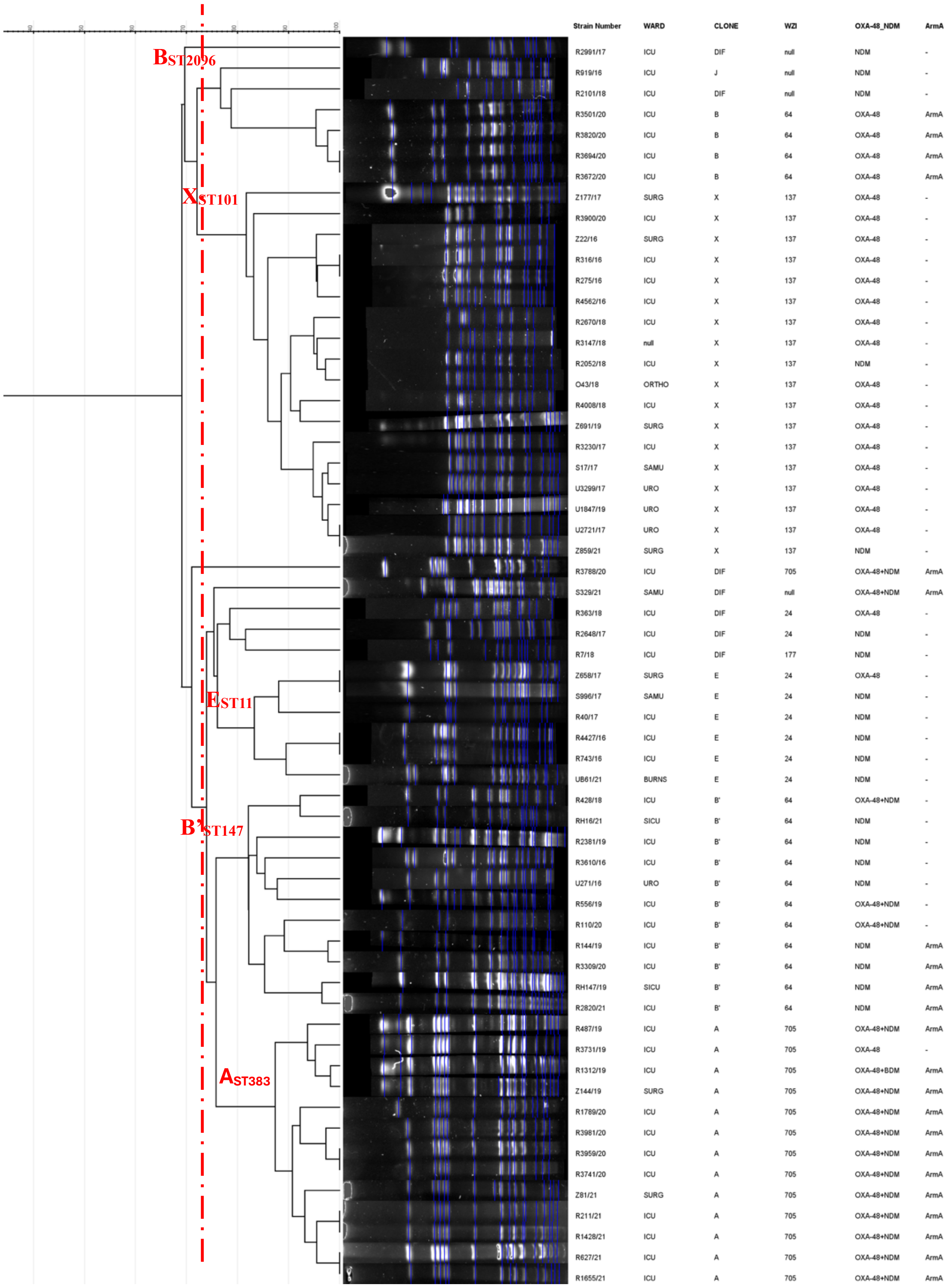

Strain Number	WARD	CLONE	WZI	OXA-48_NDM	ArmA
R2991/17	ICU	DIF	null	NDM	-
R919/16	ICU	J	null	NDM	-
R2101/18	ICU	DIF	null	NDM	-
R3501/20	ICU	B	64	OXA-48	ArmA
R3820/20	ICU	B	64	OXA-48	ArmA
R3694/20	ICU	B	64	OXA-48	ArmA
R3672/20	ICU	B	64	OXA-48	ArmA
Z177/17	SURG	X	137	OXA-48	-
R3900/20	ICU	X	137	OXA-48	-
Z22/16	SURG	X	137	OXA-48	-
R316/16	ICU	X	137	OXA-48	-
R275/16	ICU	X	137	OXA-48	-
R4562/16	ICU	X	137	OXA-48	-
R2670/18	ICU	X	137	OXA-48	-
R3147/18	null	X	137	OXA-48	-
R2052/18	ICU	X	137	NDM	-
O43/18	ORTHO	X	137	OXA-48	-
R4008/18	ICU	X	137	OXA-48	-
Z691/19	SURG	X	137	OXA-48	-
R3230/17	ICU	X	137	OXA-48	-
S17/17	SAMU	X	137	OXA-48	-
U3299/17	URO	X	137	OXA-48	-
U1847/19	URO	X	137	OXA-48	-
U2721/17	URO	X	137	OXA-48	-
Z859/21	SURG	X	137	NDM	-
R3788/20	ICU	DIF	705	OXA-48+NDM	ArmA
S329/21	SAMU	DIF	null	OXA-48+NDM	ArmA
R363/18	ICU	DIF	24	OXA-48	-
R2648/17	ICU	DIF	24	NDM	-
R7/18	ICU	DIF	177	NDM	-
Z658/17	SURG	E	24	OXA-48	-
S996/17	SAMU	E	24	NDM	-
R40/17	ICU	E	24	NDM	-
R4427/16	ICU	E	24	NDM	-
R743/16	ICU	E	24	NDM	-
UB61/21	BURNS	E	24	NDM	-
R428/18	ICU	B'	64	OXA-48+NDM	-
RH16/21	SICU	B'	64	NDM	-
R2381/19	ICU	B'	64	NDM	-
R3610/16	ICU	B'	64	NDM	-
U271/16	URO	B'	64	NDM	-
R556/19	ICU	B'	64	OXA-48+NDM	-
R110/20	ICU	B'	64	OXA-48+NDM	-
R144/19	ICU	B'	64	NDM	ArmA
R3309/20	ICU	B'	64	NDM	ArmA
RH147/19	SICU	B'	64	NDM	ArmA
R2820/21	ICU	B'	64	NDM	ArmA
R487/19	ICU	A	705	OXA-48+NDM	ArmA
R3731/19	ICU	A	705	OXA-48	-
R1312/19	ICU	A	705	OXA-48+BDM	ArmA
Z144/19	SURG	A	705	OXA-48+NDM	ArmA
R1789/20	ICU	A	705	OXA-48+NDM	ArmA
R3981/20	ICU	A	705	OXA-48+NDM	ArmA
R3959/20	ICU	A	705	OXA-48+NDM	ArmA
R3741/20	ICU	A	705	OXA-48+NDM	ArmA
Z81/21	SURG	A	705	OXA-48+NDM	ArmA
R211/21	ICU	A	705	OXA-48+NDM	ArmA
R1428/21	ICU	A	705	OXA-48+NDM	ArmA
R627/21	ICU	A	705	OXA-48+NDM	ArmA
R1655/21	ICU	A	705	OXA-48+NDM	ArmA

Fig. S2: PFGE profiles of Bloodstream CPK isolates

Dendrogram of PFGE profiles with corresponding strain metadata, including strain number/isolation year, ward, PFGE clone, wzi type, carbapenemase genotype (OXA-48, NDM), and armA. The analysis highlights the circulation and persistence of dominant clones. Ward abbreviations: ICU (Intensive Care Unit), SAMU (Emergency Unit), SURG (General Surgery), BURNS (Burn Unit).

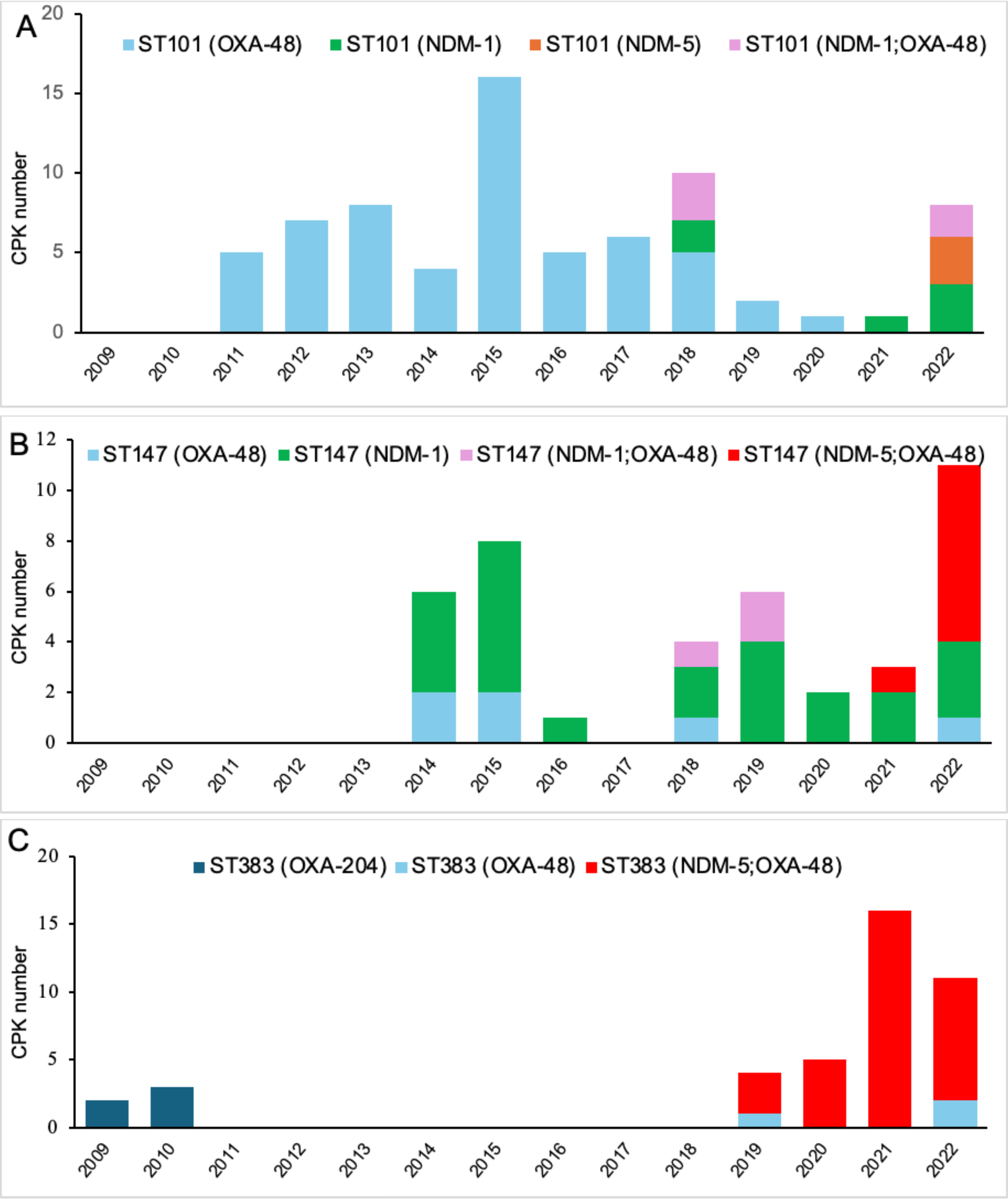

Fig. S3: Temporal trends of ST101, ST147, and ST383 in HBH, 2009–2022.
Legend: Frequency of dominant bloodstream CPK clones over time, A: ST101, B, ST147, C: ST383, in Habib Bourguiba Hospital, Sfax, Tunisia. X axis shows years; Y axis shows the number of CPK isolated by year. Sample key shows the ST-carbapenemase type association.

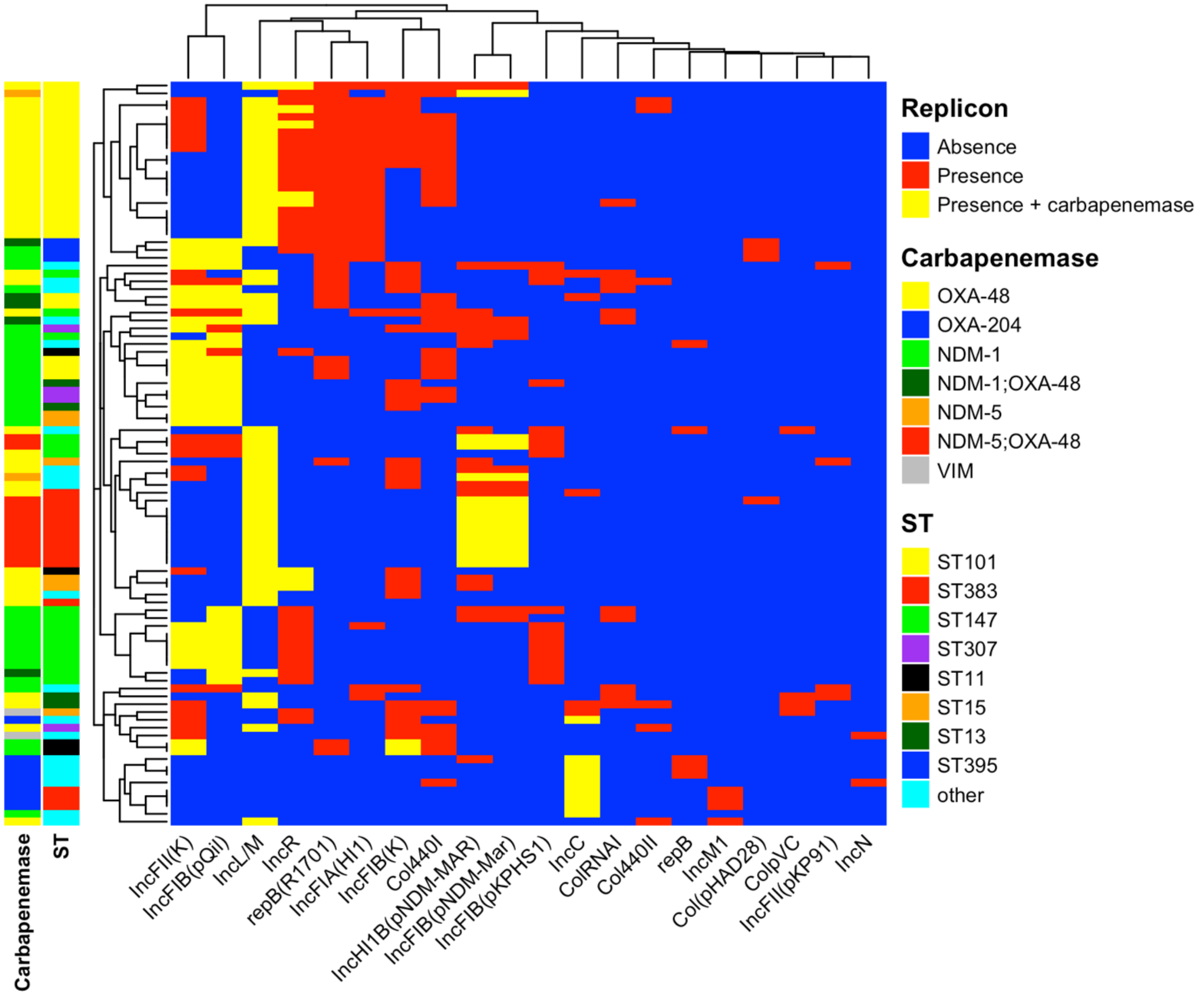

Fig. S4: Heatmap demonstrating replicon distribution across CPK isolates by sequence type

(ST - right-most column on the left-hand side) and carbapenemase type (left-most column on the left-hand side). Color key shows the main STs and carbapenemases. Red boxes in the 2D matrix represent the presence of the replicon noted along the bottom axis, yellow the replicon harboring the carbapenemases, and blue the absence of the given replicon.

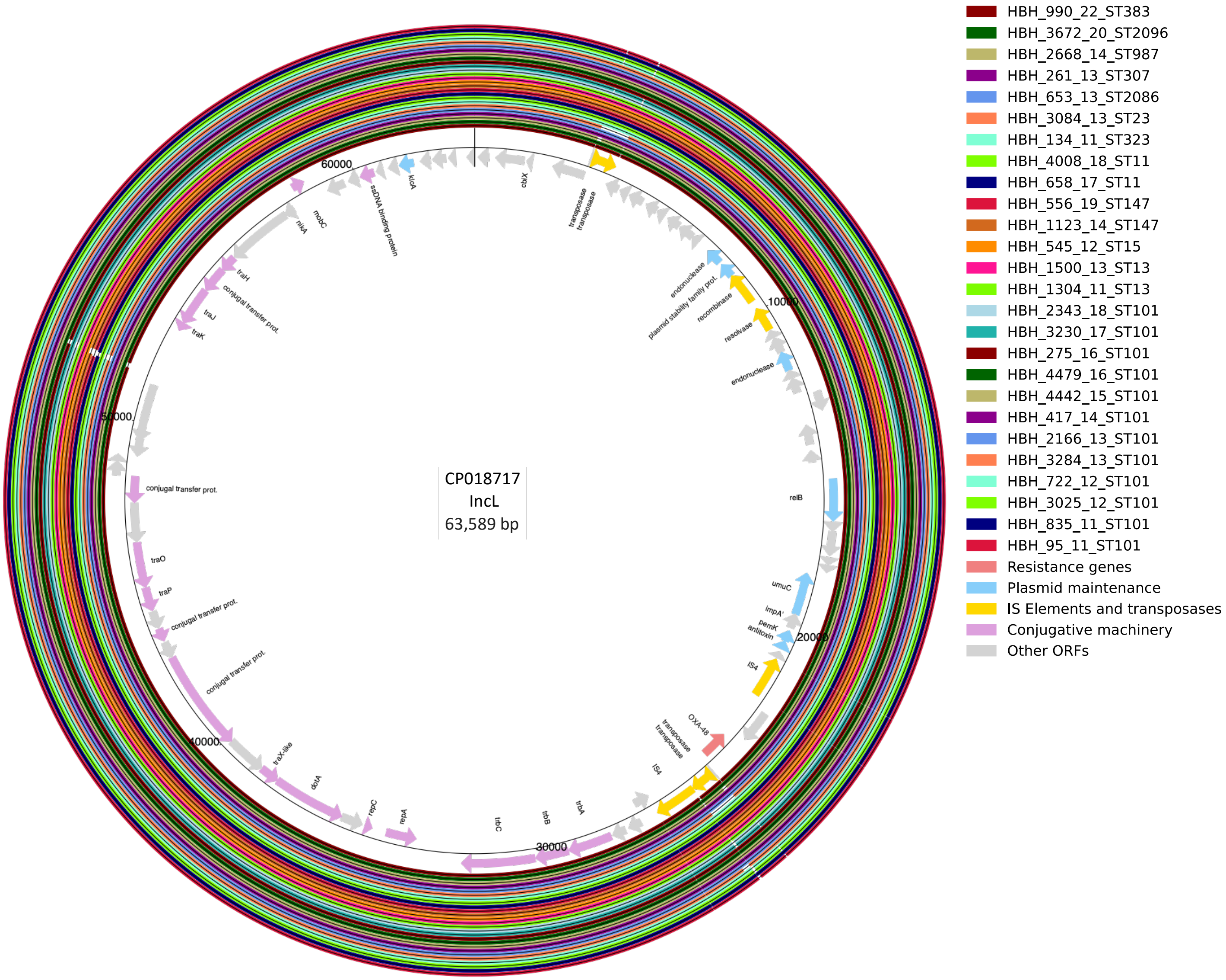

Fig. S5: *bla*$_{OXA-48}$ IncL (Mobsuite ID AA018/AH562) plasmids from study isolates.
Each ring corresponds to a plasmid from HBH CPK **(n=26 plasmids)**, identified on the right side of the figure along with the color code indicating, in order: strain identification, year of isolation, and sequence type (ST). Plasmid CP018717 was used as the reference. The genes are shown in the inner ring, represented by arrows indicating the direction of transcription.
PlasMap analyses show reads with >99% coverage and >99% identity for the IncL CP018717 plasmid.

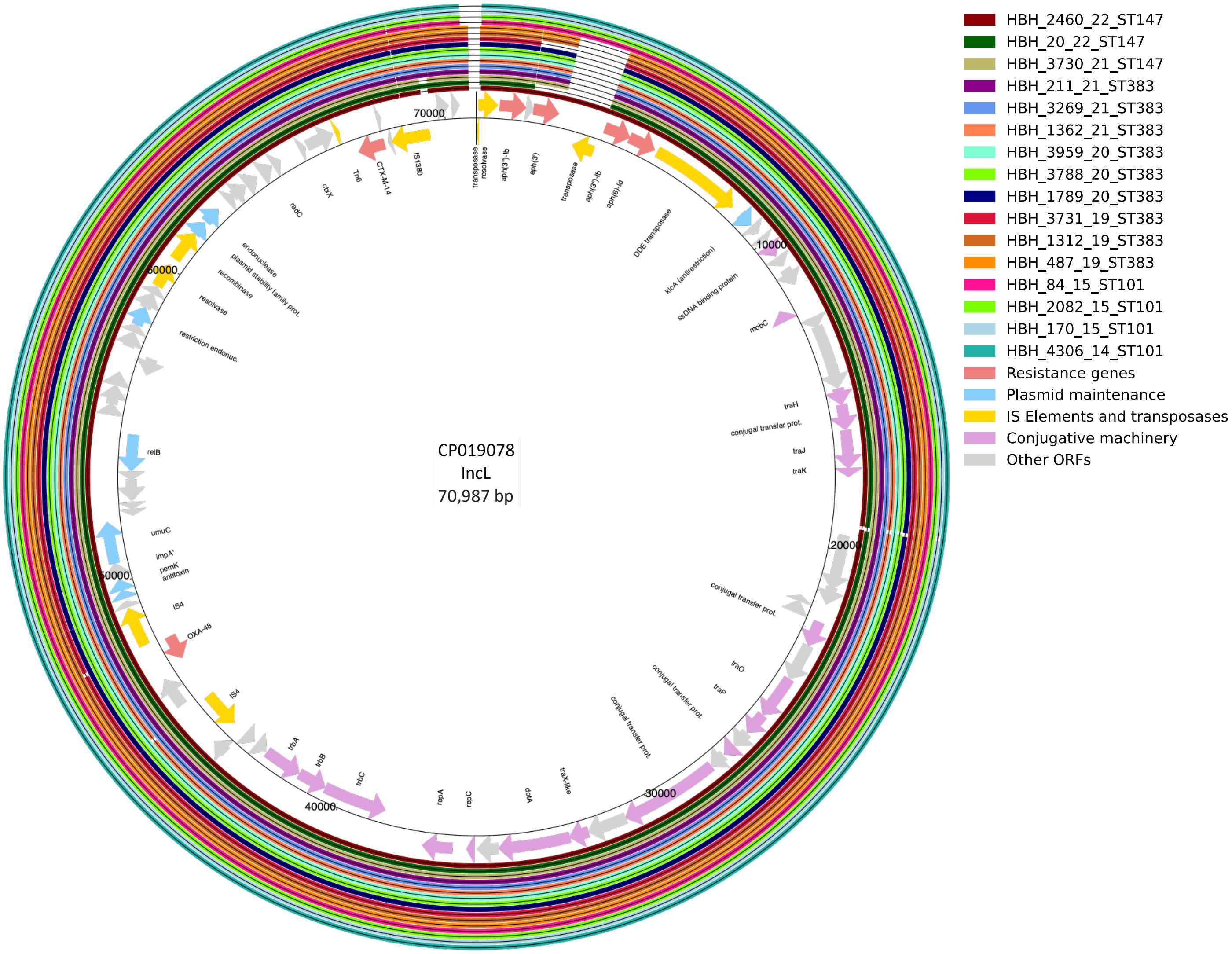

Fig. S6: *bla*$_{OXA-48}$ IncL (Mobsuite ID AA002/AH529) plasmids from study isolates.
Each ring corresponds to a plasmid from HBH CPK **(n=16 plasmids)**, identified on the right side of the figure along with the color code indicating, in order: strain identification, year of isolation, and sequence type (ST). Plasmid CP019078 was used as the reference. The genes are shown in the inner ring, represented by arrows indicating the direction of transcription. PlasMap analyses show reads with >99% coverage and >99% identity for the IncL CP019078 plasmid.

A

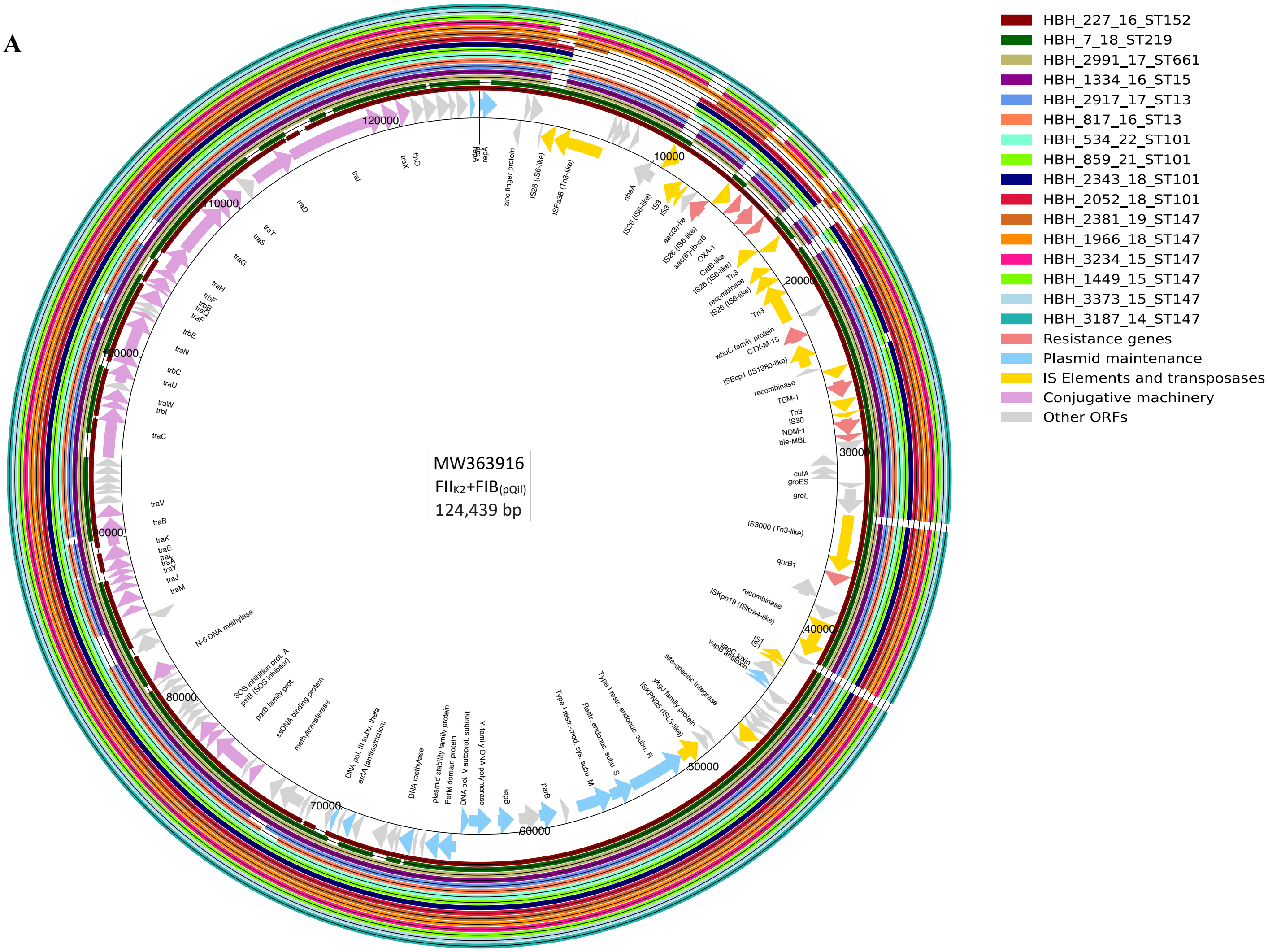

B

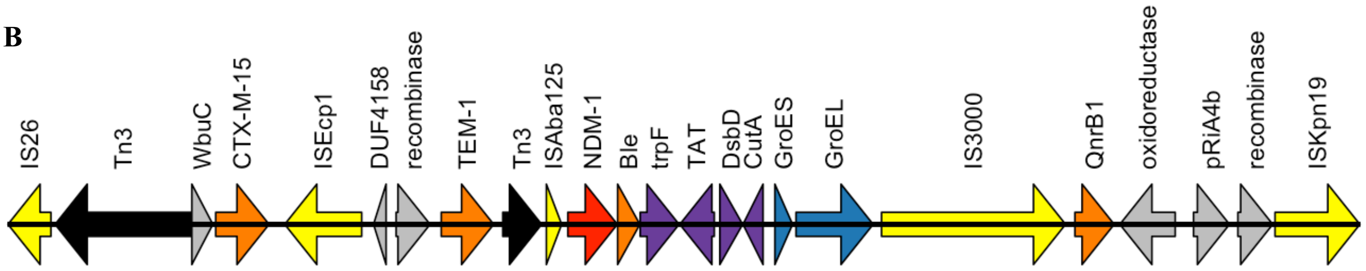

Fig. S7: FII$_{K2}$+FIB$_{(pQil)}$ *bla*$_{NDM-1}$ plasmids (Mobsuite ID AA018/AH562) from study isolates.
A: FII$_{K2}$+FIB$_{(pQil)}$ *bla*NDM-1 plasmids (n=16 plasmids). Each ring corresponds to a plasmid from HBH CPK, identified on the right side of the figure along with the color code indicating, in order: strain identification, year of isolation, and sequence type (ST). Plasmid **MW363916** was used as the reference. The genes are shown in the inner ring, represented by arrows indicating the direction of transcription. **B: Genetic context of *bla*$_{NDM-1}$ in FII$_{K2}$+FIB$_{(pQil)}$ plasmids (AA018/AH562)**
PlasMap analyses show reads with >90% coverage and >99% identity for the IncFII$_{K2}$+FIB$_{K}$ MW363916 plasmid.

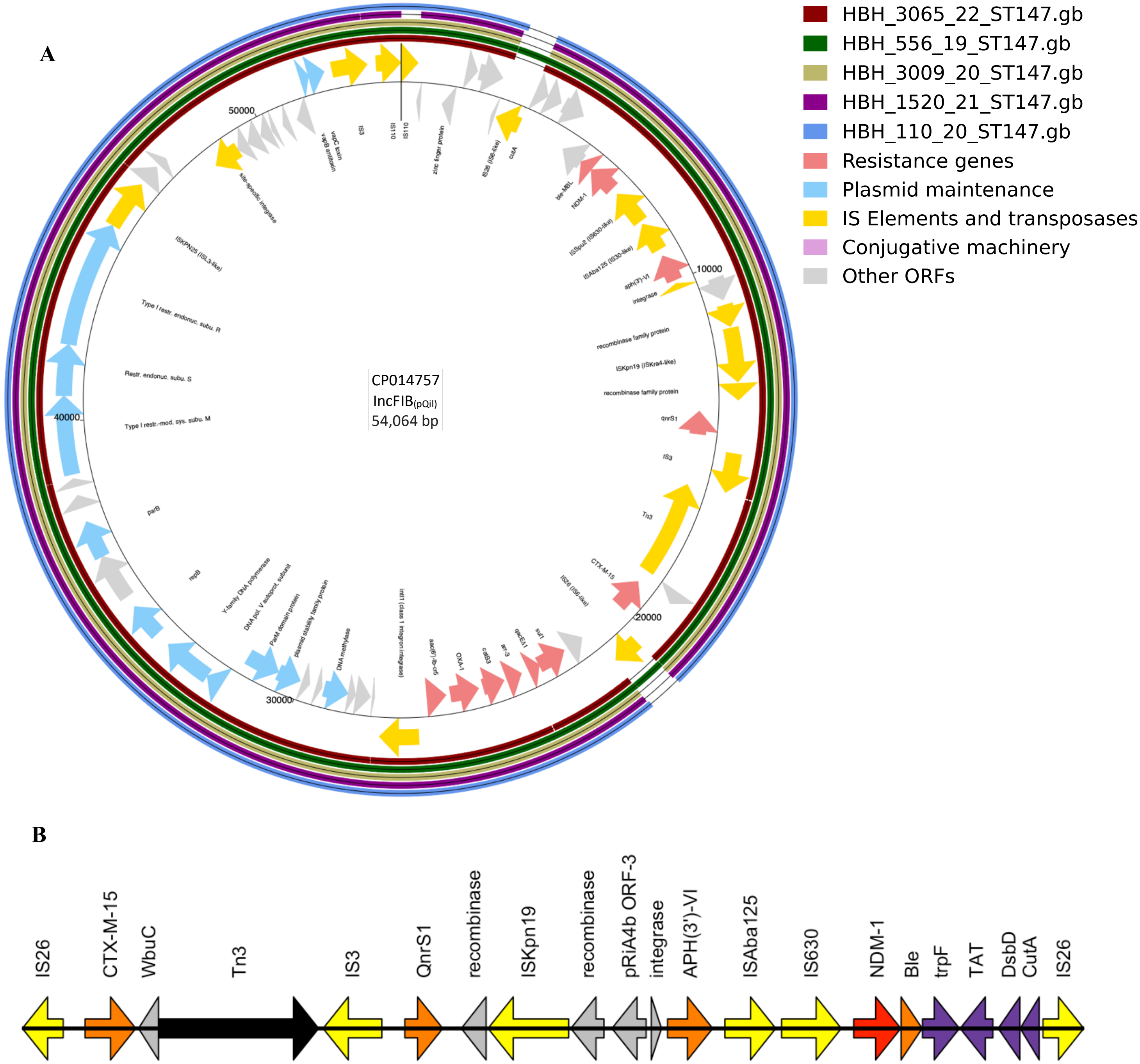

Fig. S8: *bla*$_{NDM-1}$ IncFIB$_{(pQil)}$ (AA019/AH565) from study isolates.
A: *bla*$_{NDM-1}$ IncFIB$_{(pQil)}$ (n=5 plasmids). Each ring corresponds to a plasmid from HBH CPK, identified on the right side of the figure along with the color code indicating, in order: strain identification, year of isolation, and sequence type (ST). Plasmid **CP014757** was used as the reference. The genes are shown in the inner ring, represented by arrows indicating the direction of transcription. **B: Genetic context of *bla*$_{NDM-1}$ in IncFIB(pQil) plasmids.**
PlasMap analyses show reads with >96% coverage and >99% identity for the IncFIB$_{pQil}$ CP014757 plasmid.

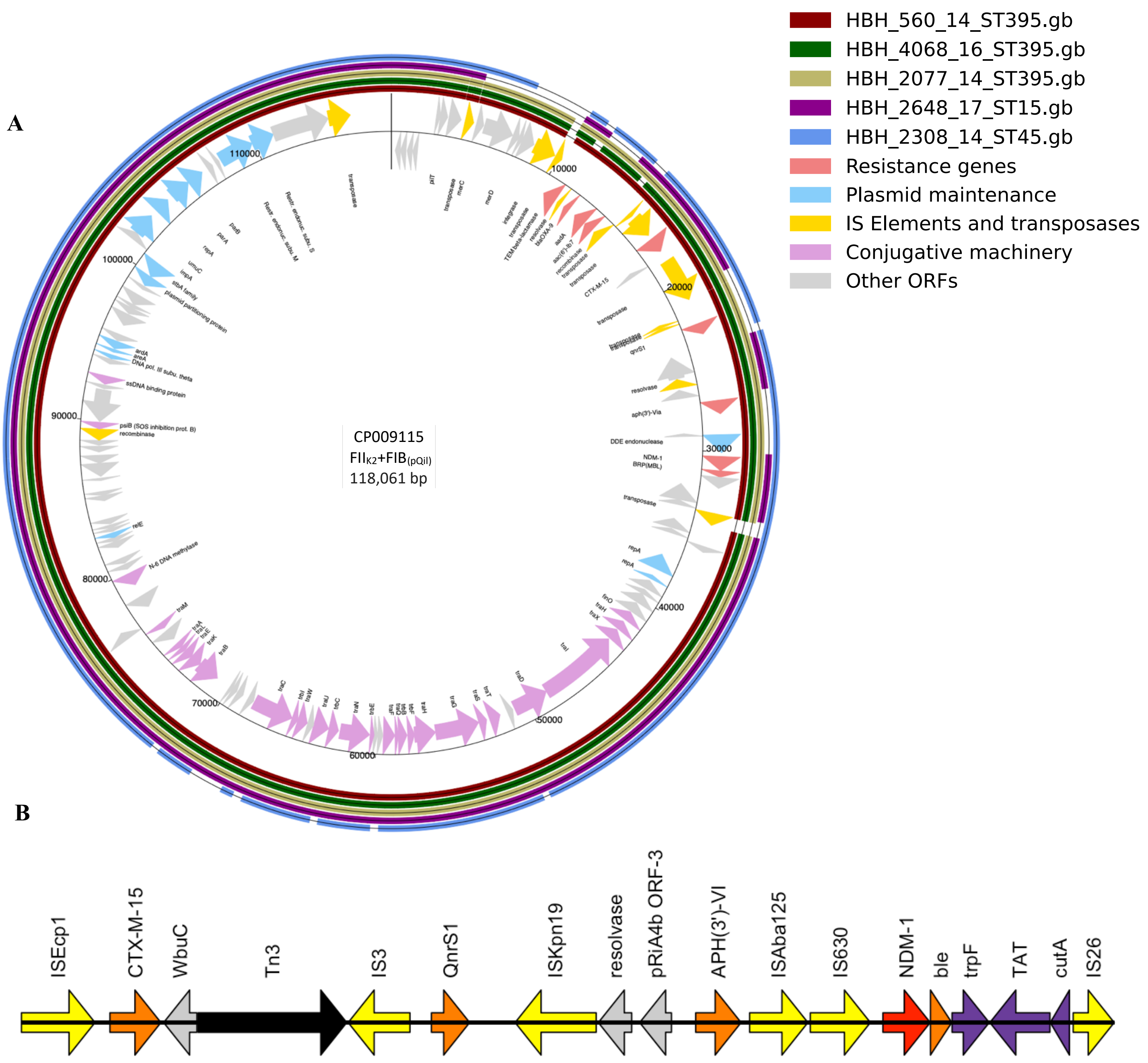

Fig. S9: FII$_{K2}$+FIB$_{(pQil)}$ *bla*$_{NDM-1}$ plasmids (Mobsuite ID AA018/AH560) from study isolates.
A: FII$_{K2}$+FIB$_{(pQil)}$ *bla*$_{NDM-1}$ plasmids (n=5 plasmids). Each ring corresponds to a plasmid from HBH CPK, identified on the right side of the figure along with the color code indicating, in order: strain identification, year of isolation, and sequence type (ST). Plasmid **CP009115** (inner ring) was used as the reference. The genes are shown in the inner ring, represented by arrows indicating the direction of transcription. **B: Genetic context of *bla*$_{NDM-1}$ in FII$_{K2}$+FIB$_{(pQil)}$ plasmids (AA018/AH560)**
PlasMap analyses show reads with >87% coverage and >99% identity for the IncFII$_{K2}$+ FIB$_{(pQil)}$ CP009115 plasmid.

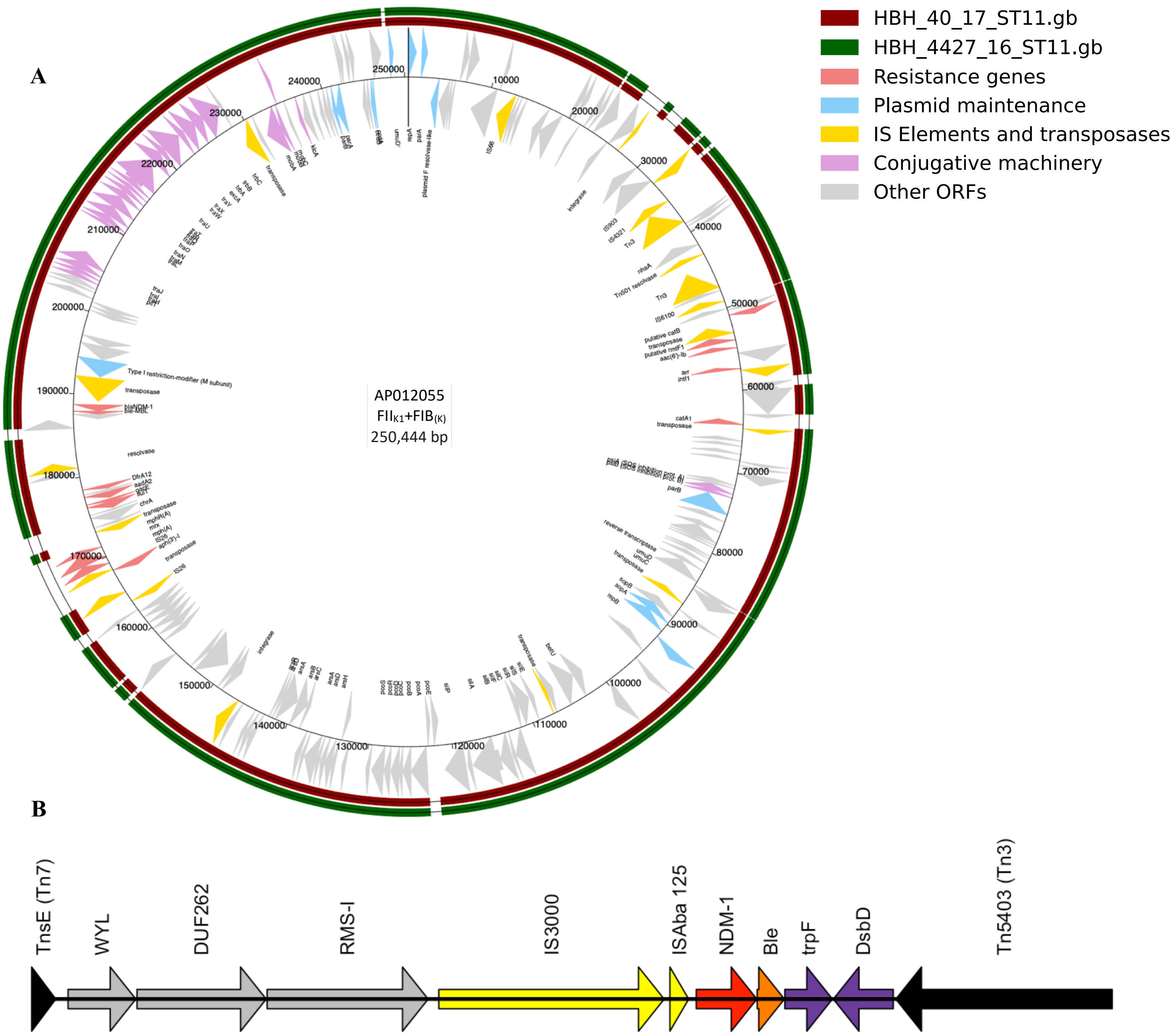

Fig. S10: FII$_{K1}$+FIB$_{(K)}$ *bla*$_{NDM-1}$ plasmids (Mobsuite ID AA274/AI071) from study isolates.

A: FII$_{K1}$+FIB$_{(K)}$ *bla*$_{NDM-1}$ plasmids (n=2 plasmids). Each ring corresponds to a plasmid from HBH CPK, identified on the right side of the figure along with the color code indicating, in order: strain identification, year of isolation, and sequence type (ST). Plasmid **AP012055** (inner ring) was used as the reference. The genes are shown in the inner ring, represented by arrows indicating the direction of transcription.

B: Genetic context of *bla*$_{NDM-1}$ in FII$_{K1}$+FIB$_{(K)}$ plasmids (AA274/AI071)

PlasMap analyses show reads with >91% coverage and >99% identity for the IncFII$_{K2}$+FIB$_{K}$ AP012055 plasmid.

A

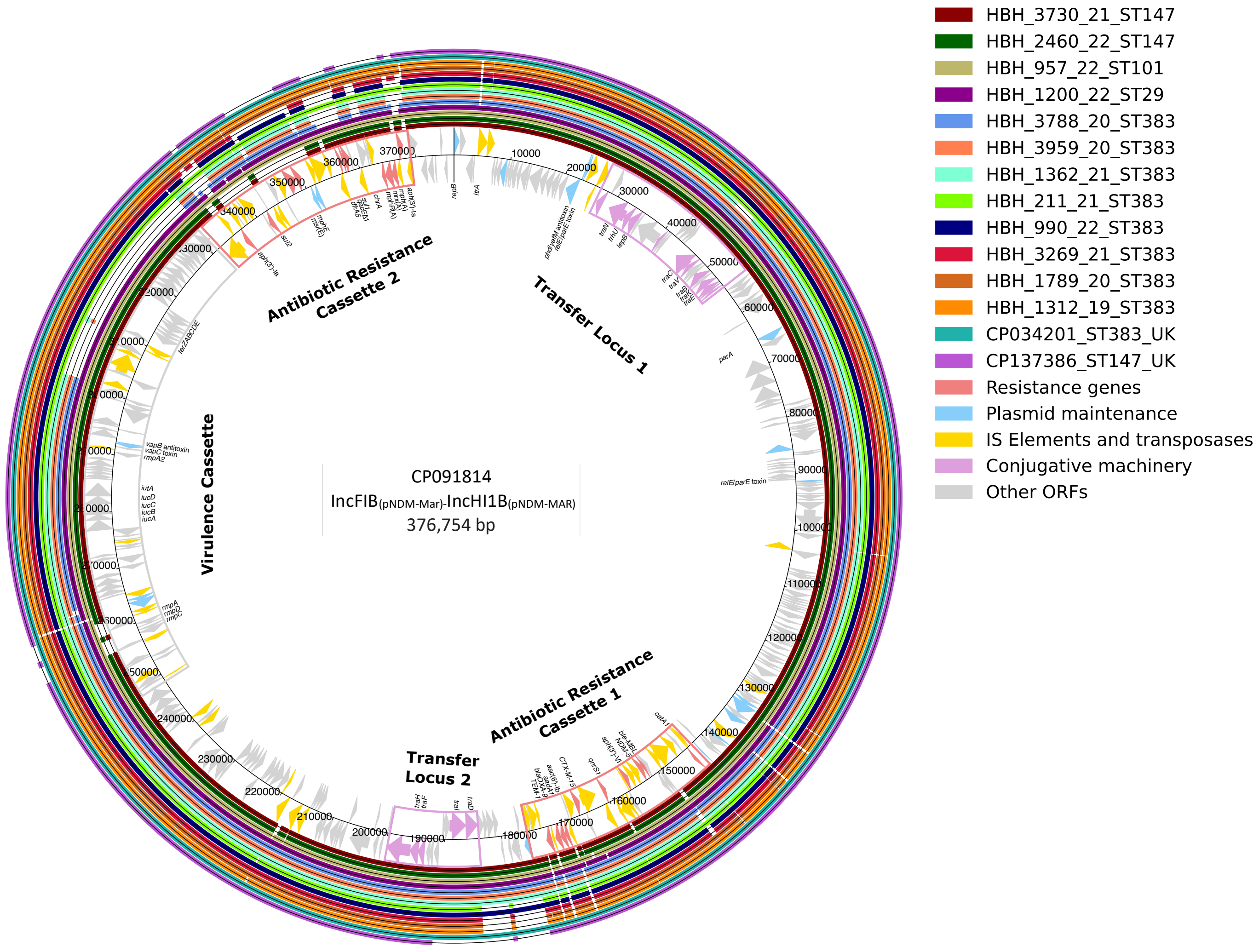

B

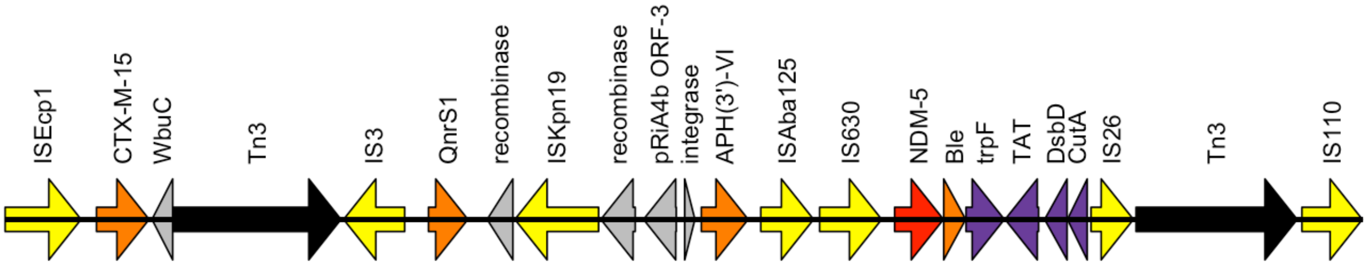

Fig. S11: *bla*$_{\text{NDM-5}}$ IncFIB(pNDM-Mar)-IncHI1B(pNDM-MAR) plasmids from study isolates.
A: *bla*$_{\text{NDM-5}}$ IncFIB(pNDM-Mar)-IncHI1B(pNDM-MAR) plasmids (n=12 plasmids). Each ring corresponds to a plasmid from HBH CPK, identified on the right side of the figure along with the color code indicating, in order: strain identification, year of isolation, and sequence type (ST). Plasmid **CP091814** (inner ring) was used as the reference. The genes are shown in the inner ring, represented by arrows indicating the direction of transcription.
B: Genetic context of *bla*$_{\text{NDM-5}}$ in IncFIB(pNDM-Mar)-IncHI1B(pNDM-MAR) plasmids
PlasMap analyses show reads with >86% coverage and >98% identity for the IncFIB-IncHI1B CP091814 plasmid.

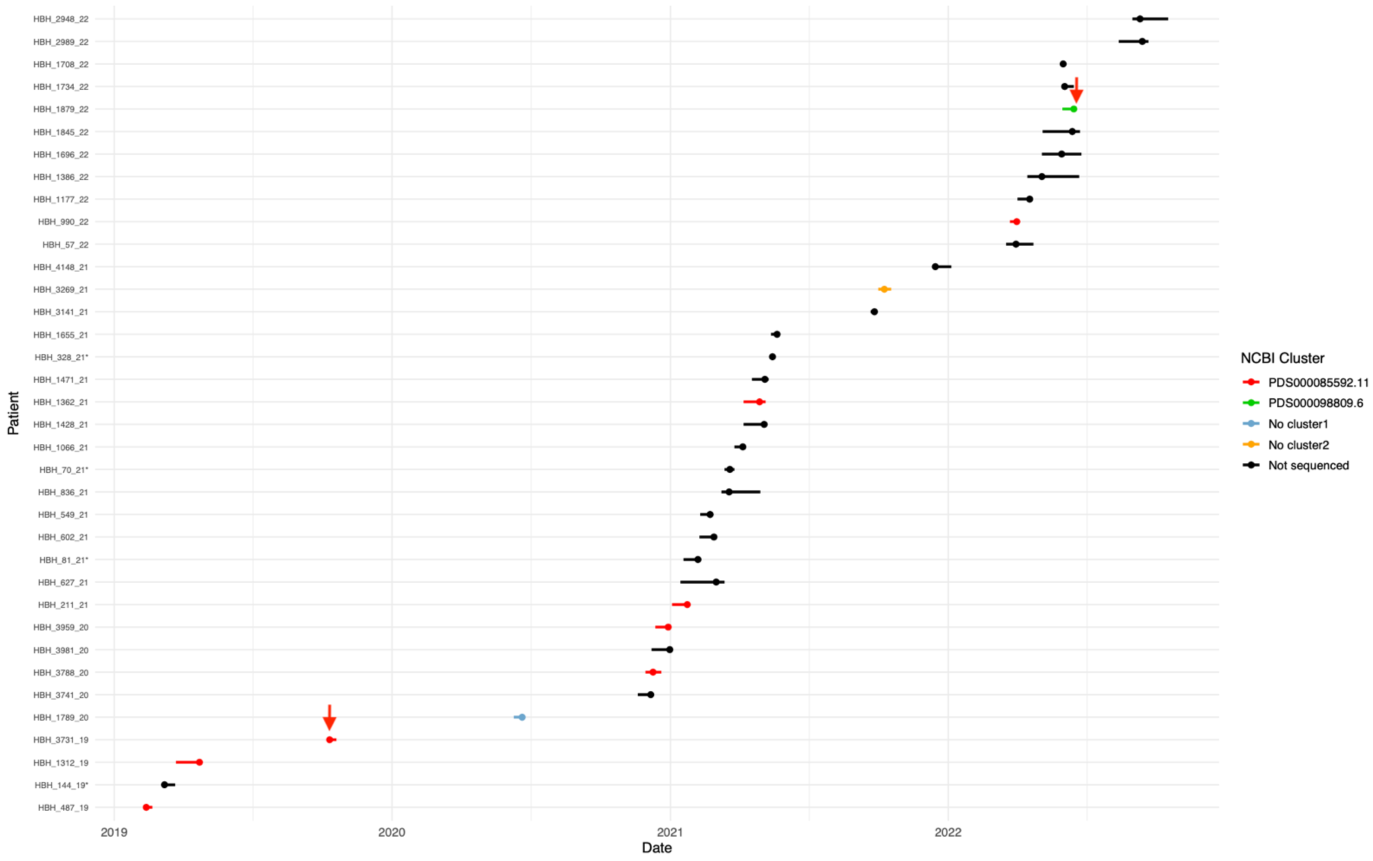

Fig S12: Hospitalization timelines and sampling dates of ST383 CPK bloodstream cases belonging to PFGE profile A at HBH

Horizontal lines show hospitalization periods and dots the sampling dates. Color coding indicates NCBI SNP cluster assignments: red, cluster PDS000085592.11 (6 isolates); green, cluster PDS000098809.6 (1 isolate); blue and orange, isolates sequenced but not assigned to a defined NCBI cluster; and black, isolates that were not sequenced. All cases were documented in the ICU, except those marked with an asterisk (*). The figure shows overlapping hospitalizations, suggesting possible cross-transmission events. Arrows denote the reintroduction of a novel clone showing stronger genomic relatedness to international strains than to local ones, based on NCBI Pathogen Detection analyses.

Supplementary Tables

Table S1: Carbapenemase and virulence gene distribution by culture site

	Total	Carbapenemase genes				Virulence genes		
		bla$_{NDM}$	***bla***$_{OXA48}$	***bla***$_{NDM}$ **+ *bla***$_{OXA4}$	***bla***$_{VIM}$	***rmpA2***	***rmpA***	***iuc***
Urine	380	117 (30.8)	156 (41.1)	107 (28.2)		90 (23.7)	66 (17.4)	86 (22.6)
Blood	220	64 (29.1)	102 (46.4)	52 (23.2)	2	61 (27.7)	45 (20.5)	56 (25.5)
Sputum	152	23 (15.1)	80 (52.6)	49 (32.2)		50 (32.9)	36 (23.7)	46 (30.3)
Skin, soft tissue	189	50 (25.3)	97 (49.0)	51 (25.8)		45 (23.8)	34 (18.0)	39 (20.6)
Other	63	27 (42.9)	22 (34.9)	14 (22.2)		17 (27.0)	15 (23.8)	15 (23.8)
P value		0.0002	0.03901	0.3603		0.1782	0.3591	0.2039

P-values show the comparison of carbapenemase or virulence genes between the different types of culture sites.

Table S2: PFGE pulsotypes of bloodstream CPK isolates

Year	CKP Number	Number of strains per PFGE profile (number of sequenced strains)	Number of sequenced strains per year
**2009**	**3**	**2 F$_{ST383}$ (1), 1 V$_{ST340}$ (1)**	2
**2010**	**7**	**3 F$_{ST383}$ (1), 2H $_{ST218}$ (2), 1 V$_{ST340}$ (1), 1 unique (1)**	5
**2011**	**8**	**3 A'$_{ST101}$ (1), 2 D$_{ST101}$ (1), 2 S (1), unique (1)**	4
**2012**	**11**	**7 A'$_{ST101}$ (2), 3 unique (2), 1 NT (1)**	5
**2013**	**15**	**4 A$_{ST101}$ (1), 4 D$_{ST101}$ (2), 6 unique (6)**	9
**2014**	**17**	**3 D$_{ST101}$ (1), 5 B'$_{ST147}$ (1), 2 K$_{ST395}$ (2), 3 unique (2), 4 NT (2)**	9
**2015**	**32**	**12 D$_{ST101}$ (3), 3 A$_{ST101}$ (1), 7 B'$_{ST147}$ (3), 2 Q$_{ST11}$ (1), 6 unique (3), 2 NT**	10
**2016**	**17**	**3 A$_{ST101}$ (1) , 2 X"$_{ST101}$ (1), B'$_{ST147}$ (1), 2 H$_{ST152}$ (1), 3 E $_{ST11}$ (1), 3 J$_{ST15}$ (1), 1 K $_{ST395}$ (1), 2 unique (2)**	8
**2017**	**12**	**5 X$_{ST101}$ (1), 1 X"$_{ST101}$ (1), 2 E$_{ST11}$ (1), 1 E'$_{ST11}$ (1), 1 J$_{ST15}$ (1), 2 unique (2)**	7
**2018**	**16**	**7 X$_{ST101}$ (2), 1 X"$_{ST101}$ (1), 2 B'$_{ST147}$ (1), 3 unique (2), 3 NT (2)**	8
**2019**	**13**	**6 B'$_{ST147}$ (2), 4 A$_{ST383}$ (3), 2 X$_{ST101}$, 1 NT**	5
**2020**	**13**	**5 A$_{ST383}$ (3), 5 B$_{ST1096}$ (1) , 2 B'$_{ST147}$ (2), 1 unique (1)**	7
**2021**	**21**	**15 A$_{ST383}$ (3) , 2 B'$_{ST147}$ (1), 1 D$_{ST147}$ (1), 1 X$_{ST101}$ (1), 1 unique (1), 1 NT**	7
**2022**	**35**	**11 A$_{ST383}$ (2), 3 B'$_{ST147}$ (1), 8 D$_{ST147}$ (2), 3 X"$_{ST101}$ (1), 5 X$_{ST101}$ (1), 5 unique (1)**	8

The table summarizes the main PFGE profiles, the number of strains per profile, and the unique pulsotypes, indicating in parentheses the number of sequenced strains per PFGE. NT: non-typable

Table S3 (Supplementary Data 2, p.1): PFGE Profiles, antimicrobial resistance, and virulence features of the 220 bloodstream CPK Isolates

Table S4: Features of *bla*$_{OXA-48}$ plasmids in bloodstream CPK isolates

Rep_type(s)	IncL* (pHB1)	IncL (pHB2)	IncL (+/- IncR,IncHI1B) (pHB8)	IncL	IncL	IncL	IncL,IncR	IncM1
**Rep_type_accession(s)**	JN626286	JN626286	JN626286 (+/- 000204_CP008701_00115, JN420336)	JN626286	JN626286	JN626286	JN626286, 000204_CP008701_00115	U27345
**Mash_nearest_ neighbor**	**CP018717**	**CP019078**	**KX523901**	**KY215945**	**KY213890**	**KX636096**	**LN864819**	**KP025948**
**Mash distance mean (SD)**	0,0023 (0.003)	0.0031 (0.0027)	0.0064 (0.0055)	0.012 (0.002)	0.01355	0.0007	0.0113	0.0015
**Primary/**	AA002	AA002	AA002	AA002	AA002	AA002	AA002	AA002
**Secondary_cluster_id**	/AH539	/AH529	/AH539	/AH539	/AH539	/AH539	/AH539	/AH529
**Relaxase_type**	MOBP	MOBP	MOBP	MOBP	MOBP	MOBP	MOBP	MOBP
**MPF_type**	MPF_I	MPF_I	MPF_I	MPF_I	MPF_I	MPF_I	MPF_I	MPF_I
**Predicted_mobility**	conjugative	conjugative	conjugative	conjugative	conjugative	conjugative	conjugative	conjugative
**Plasmid size pb Mean (SD)**	65917.1 (9438.8)	71823.3 (8111.1)	79955.6 (17049.9)	44511 (5545.1)	41882	59211	92377	71108
**Resistance genes in plasmids**	-	CTX-M-14	-	-	-	-	-	-
**Years of identification**	2011-2022	2014-2022	2013-2020	2018	2022	2013	2017	2013
**STs (number)**	**ST101 (13)**, ST147 (2), ST13 (2), ST15 (1), ST383 (1), ST11 (1), ST307 (1), ST2086 (1), ST2096 (1), ST23 (1), ST323 (1), ST987 (1)	**ST383 (8)**, ST101(4), ST147 (3)	ST15 (2), ST101(1), ST395 (1), ST45 (1)	ST147 (1), ST15 (1)	ST383 (1)	ST101 (1)	ST101 (1)	ST514 (1)
**Total Number**	**27**	**15**	**5**	**1**	**1**	**1**	**1**	**1**

*: 3 IncL plasmids were associated with IncR replicon: 000204_CP008701_00115

The table summarizes the features of the study plasmids identified by MOB-suite, which groups plasmids into primary and secondary clusters with pairwise Mash distances. Values obtained from MOB-suite indicate the replicon types (rep_type(s)), the Mash nearest neighbor, and the corresponding mash distance. MOB-suite classifies the Mobility of plasmids based on the presence of relaxase (mobilizable) and/or MPF proteins (conjugative) or absence of both (non-mobilizable), and plasmid size. Additional resistance or virulence genes were detected within the plasmid contigs using CGE tools.

Table S5: Distribution of *bla*$_{OXA-48}$ plasmids transconjugants by replicon type and sequence type (ST) of blood CPK isolates

	No. of transconjugants (No. of ST)							
**Mash_nearest_ neighbor**	**CP018717**	**CP019078**	**KX523901**	**KY215945**	**KY213890**	**KX636096**	**LN864819**	**KP025948**
**Rep-type**	**(pHB1)**	**(pHB2)**	**(pHB8)**					
**ST donor**	**IncL**	**IncL**	**IncL (+/- IncR,IncHI1B)**	**IncL**	**IncL**	**IncL**	**IncL,IncR**	**IncM1**
**ST147**	1 (2)	1 (3)		0 (1)				
**ST101**	**12 (13)**	3 (4)	1 (1)			1 (1)	1 (1)	
**ST13**	2 (2)							
**ST15**	1 (1)		2 (2)	1 (1)				
**ST383**	2 (2)	**1 (8)**			0 (1)			
**ST11**	1 (1)							
**ST395**			1 (1)					
**ST307**	1 (1)							
**ST45**			1 (1)					
**ST2086**	1 (1)							
**ST2096**	1 (1)							
**ST23**	0 (1)							
**ST323**	1 (1)							
**ST514**								1 (1)
**ST987**	1 (1)							
**Total**	**24 (27)**	**5 (15)**	**5 (5)**	**1 (2)**	**0 (1)**	**1 (1)**	**1 (1)**	**1 (1)**

Values indicate the number of transconjugants, with the number of donor STs shown in parentheses. Columns correspond to the Mash nearest-neighbor reference plasmids and their associated replicon types.

Table S6: Features of *bla*$_{NDM-1.}$ plasmids in bloodstream CPK isolates

**Rep_type(s)**	**IncFIB(pQil); IncFII(K:2) (pHB3)**	**IncFIB(pQil); IncFII(K:2)**	**IncFIB(pQil); IncFII(K:2) (pHB6)**	**IncFIB(pQil) (pHB5)**	**IncFIB(K); IncFII(K:1) (pHB7)**	**IncC:3**	**IncC:3**	IND
**Mash_nearest_neighbor**	**MW363916**	**MW363914**	**CP009115**	**CP014757**	**AP012055**	**MG450360**	**CP043190**	
**Mash distance mean (SD)**	0.00208 (0.0014)	0.0069 (0.0015)	0.00266 (0.00198)	0.0077 (0.006)	0.01039	0.00528	0.01104	
**Primary/secondary_cluster_ID**	AA018/AH562	AA018/AH562	AA018/AH560	AA019/AH565	AA274/AI071	AA860/AJ275	AA860/AJ275	
**Relaxase_type**	MOBF,MOBF	MOBF,MOBF	MOBF,MOBF	-	MOBF,MOBF,MOBP	MOBH,MOBH	MOBH,MOBH	
**MPF_type**	MPF_F	MPF_F	MPF_F	-	MPF_F	MPF_F	MPF_F	
**Predicted_mobility**	conjugative	conjugative	conjugative	non-mobilizable	conjugative	conjugative	conjugative	
**Plasmid size pb Mean (SD)**	112717 (8315.2)	82143.5 (4891.1)	113096.2 (8720.7)	40651.2 (10936.2)	298673.5 (2935.2)	202974	54109	
**Years of identification**	2014-2022	2014-2015	2014-2017	2019-2022	2016-2017	2018	2021	2015-2016
**Resistance genes in plasmids**	NDM1-M15-AAC6-AAC3-OXA1-TEM1-CATB-QNRB1	NDM1-QNRB1	NDM1-M15-TEM-APH3VI-OXA9-AAD-QNRS1	NDM1-M15-QNRS1-AAR3-CATB-AAC6-APH3	NDM1-M15-OXA1-RMTF-CATB-ARR-SUL1-DFR-AAC3-AAD-AAC6	NDM1-CMY-AAC-AAD-ARM-APH3-APH6-OX1-CATB-SUL1-SUL2-TET-QNRA6-AAR		
**STs (Number)**	ST147 (6), ST101(4), ST13(2), ST15 (1), ST152 (1), ST219 (1), ST661 (1)	ST307 (2)	ST15 (1), ST395 (3), ST45 (1)	ST147 (5)	ST11 (2)	ST101 (1)	ST1418 (1)	ST11 (1), ST307 (1), ST45 (1)
**Total**	**16 (13)**	**2 (1)**	**5 (2)**	**5 (0)**	**2 (0)**	**1 (1)**	**1(0)**	**3 (2)**

The table summarizes the features of the study plasmids identified by MOB-suite, which groups plasmids into primary and secondary clusters with pairwise Mash distances. Values obtained from MOB-suite indicate the replicon types (rep_type(s)), the Mash nearest neighbor, and the corresponding mash distance. MOB-suite classifies the Mobility of plasmids based on the presence of relaxase (mobilizable) and/or MPF proteins (conjugative) or absence of both (non-mobilizable), and plasmid size. Additional resistance or virulence genes were detected within the plasmid contigs using CGE tools.

Table S7: Distribution of *bla*$_{NDM-1}$ plasmids transconjugants by replicon type and sequence type (ST) of blood CPK isolates

	**No. of transconjugants (No. of ST)**							
**Mash_nearest_neighbor**	**MW363916 (pHB3)**	**MW363914**	**CP009115 (pHB6)**	**CP014757 (pHB5)**	**AP012055 (pHB7)**	**MG450360**	**CP043190**	IND
**Rep_type(s)** / **ST donor**	**IncFIB(pQil); IncFII(K:2)**			**IncFIB(pQil)**	**IncFIB(K); IncFII(K:1)**	**IncC:3**		IND
**ST147**	**5 (6)**			**0 (5)**				
**ST101**	**2 (4)**					1 (1)		
**ST13**	2 (2)							
**ST15**	1 (1)		1 (1)					
**ST152**	1 (1)							
**ST219**	1 (1)							
**ST661**	1 (1)							
**ST11**					0 (2)			1 (1)
**ST395**			0 (3)					
**ST307**		1 (2)						1 (1)
**ST45**			1 (1)					0 (1)
**ST1418**							0 (1)	
**Total**	**13 (16)**	**1 (2)**	**2 (5)**	**0 (5)**	**0 (2)**	**1 (1)**	**0 (1)**	**2 (3)**

Values indicate the number of transconjugants, with the number of donor STs shown in parentheses. Columns correspond to the Mash nearest-neighbor reference plasmids and their associated replicon types.

Table S8: (Supplementary Data 2, p1): Sequence records of the NCBI Reference Plasmids used in the study.

Table S9: (Supplementary Data 2, p2): Alignment summaries of the study plasmids to the reference plasmids

Results showed as percentages of coverage and identity to the reference plasmids.

Table S10: Characteristics of the ST383 *K. pneumoniae* isolates reported in the 42 studies published in PubMed by June 5, 2025

Reference	Country	Setting	Study isolates	Year	Collection site	ST383 isolates					
						Number	ST383 % among CRK	ST383 % among all KPN	ST383 Outbreak	Carbapenemase types	Virulence markers (*rmpA/iuc*)
**Rotondo 2024** [1]	**Italy**	**19 hospitals in the Lazio region**	**126 NDM-Kpn isolates**	**2020 and 2023**	**diverse**	**6**	**4.8%**	**-**	**-**	**NDM5 (2); NDM1 (4)**	**1 (16 %)**
Padovani 2023 [2]	Italy	Brescia	6 Kpn resistant to cefiderocol	2021 and 2022	diverse	2	-	-	-	NDM1-OXA48 (2)	0
Spaziante 2021 [3]	Italy	Lazio region hospitals	all inpatients with NDM-Kpn strain (17 NDM-Kpn)	January 2019 and June 2020	diverse	4	23.5% among NDM-Kpn	-	-	NDM5 (1), NDM1-OXA48 (3)	NS
**Lorenzin 2022** [4]	**Italy**	**IRCCS San Raffaele Scientific Institute, Milan**	**4 XDR Hyperviruent Kpn isolates**	**2019**	**recta swab**	**2**	**-**	**-**	**-**	**NDM5-OXA48 (1) ; NDM1-OXA48 (1)**	**2 (100 %)**
**Ventura 2022** [5]	**Italy**	**Verona Hospital**	**19 hypermucoviscous Kpn isolates**	**2021**	**blood cultures and abscesses**	**1**	**-**	**-**	**-**	**VIM1 + NDM5 (1)**	**1 (100 %)**
**Turton 2019** [6]	**UK**	**South East England**	**12 CPK with hybrid virulence plasmids**	**2016-2017**	**diverse**	**3**	**-**	**-**	**-**	**NDM5-OXA48 (2), OXA48 (1)**	**3 (100 %)**
**Hammad 2025** [7]	**Egypt**	**ICU at Assiut University Hospital**	**case report**	**2015**	**endotracheal aspirates**	**1**	**-**	**-**	**-**	**NDM5-OXA48 (1)**	**1 (100 %)**
**Attalla 2023** [8]	**Egypt**	**ICUs in Alexandria**	**17 colistin-resistant Kpn isolates**	**2020 (6 months)**	**diverse**	**7**	**-**	**-**	**-**	**NDM5-OXA48 (7)**	**6 (85 %)**
**Attalla 2024** [9]	**Egypt**	**Alexandria Main University Hospita**	**7 seleceted colistin-resistant Kpn isolates**	**2021**	**diverse**	**2**	**-**	**-**	**-**	**NDM5-OXA48 (2)**	**NS**
**Abdelsalam 2024** [10]	**Egypt**	**Microbiology laboratory in Alexandria**	**19 selected CRK**	**August 2020, and April 2021**	**diverse**	**5**	**26.3 %**	**-**	**-**	**NDM5-OXA48 (3) ; NDM1-OXA48 (2)**	**5 (100%)**
**Gamaleldin 2024** [11]	**Egypt**	**Alexandria Main University Hospital**	**27 sequenced among 56 MDR Kpn isolates**	**2019 et 2021**	**diverse**	**4**	**-**	**14.8 % among MDR-Kpn**	**-**	**NDM1 (1); OXA48 (1); NDM5-OXA48 (1)**	**NS**

Edward 2022 [12]	Egypt	Mabaret Al-Asafra Hospitals	23 3GC resistant Kpn isolates, one selected for WGS	2020	diverse	1	-	-	-	OXA48 (1); NDM5-OXA48 (1)	NS
AhmedMAEE 2021 [13]	Egypt	Demerdash Hospital (Cairo, Egypt)	34 Kpn isolates	June 2017 and March 2018	Blood	5		14.7 % among sepsis-Kpn	-	NP	NS
Osman 2023 [14]	Sudan	5 hospitals in Khartoum	86 Kpn isolates (68 MDR)	2016- 2020	diverse	5	-	-	-	NDM5-OXA48 (4)	2 (20 %)
Sobh 2024 [15]	Lebanon	American University of Beirut Medical Center	34 ceftazidime-avibactam resistant Kpn isolates (17 sequenced)	2019 and 2021	diverse	12	-	-	-	NDM5-OXA48 (12)	3 (25 %)
Dagher 2019 [16]	Lebanon	Saint George Hospital in Beirut	5 cases of MDR Kpn isolates	2017	diverse	5	-	-	-	NDM5 (5)	NS
Elgriw 2023 [17]	Lybia	1 hospital (TUH)	44 CRK	2019 and 2021	diverse	6	13.6 %	-	-	OXA48 (2); NDM5-OXA48 (3)	yes (2 sequenced)
Eltai 2020 [18]	Qatar	Hamad Medical Corporation	18 colistin-resistant Kpn isolates	NP	diverse	3	-	-	-	OXA48 (1); NDM5-OXA48 (1)	
Tsui 2023 [19]	Qatar	Hamad Medical Corporation	95 CPK	April 2016 to October 2017	diverse	4	4.2 %	-	-	4 NDM5-OXA48 (4)	4 (100 %)
Abid 2021 [20]	Qatar	Hamad Medical Corporation	81 CPK	April 2014 toNovember 2017	diverse	4	4.9 %	-	-	OXA48 (1); NDM5 + OXA48 (3)	NS
SidAhmed 2024 [21]	Qatar	Hamad Medical Corporation	3 XDR Kpn isolates	NP	urine and Respiratory tract	1	-	-	YES	NDM5-OXA48 (1)	NS
Huang 2024 [22]	Saudi Arabia	34 KSA hospitals	352 MDR Kpn isolates	January 2022 and April 2023	Blood and urine	2	-	0.6 % among MDR-Kpn	-	NDM5-OXA48 (2)	2 (100 %)
Al-Zahrani [23]	Saudi Arabia	tertiary hospital in Jeddah	29 selected CPK	NA	diverse	2	-	-	-	NDM5-OXA48 (2)	2 (100 %)
Alghoribi 2020 [24]	Saudi Arabia	King Khalid University Hospital	case report	NP	wound	1	-	-	-	KPC2 (1)	2 (100 %)

Chiarelli 2020 [25]	France	Bicetre Hospital	one strain selected for in vitro studies	2017	Blood	1	-	-	-	KPC2 (1)	0
Bonnin 2020 [26]	France	France's National Reference Center for Antimicrobial Resistance	63 nonduplicate KPC-*Kp*	2018	diverse	4	6.4% among KPC-Kpn	-	-	KPC2 (4)	0
Sabirova JS [27]	Greece	Tzaneio General Hospital (TGH)	12 CPK	2010–13	diverse	12	-	-	-	KPC2 (7); VIM19 (11)	0
Mavroidi 2016 [28]	Greece	Konstantopouleio-Patission hospital	19 colistin and carbapenem-resistant Kpn isolates among 135 Kpn	July 2012 to December 2013	diverse	1	-	-	-	KPC2 (1)	0
Papagiannitsis 2016 [29]	Greece	NP	Case report of VIM-19 in IncA/C	2015	NP	1	-	-	-	VIM19 (1)	NS
Pitt 2018 [30]	Greece and Brazil	Hygeia General Hospital, Athen and Instituto Dante Pazzanese de Cardiologia, Brazil	19 colistin-resistant Kpn isolates	2012-2014	diverse	2	-	-	-	-	0
Xanthopoulou 2022 [31]	Germany	6 hospitals	39 CRK	2016–2018	diverse	1	2.6 %	-	-	VIM-19 (1)	0
Giakkoupi 2010 [32]	Greece	40 Greek hospitals	378 KPC-2 Kpn isolates	January 2009-April 2010	diverse	9	2.4 % among KPC-Kpn	-	-	KPC2 (2)	NP
Papagiannitsis 2010 [33]	Greece	Greece	Case report of ST383 producing VIM-4, KPC-2 and CMY-4	2009	NP	1	-	-	-	VIM4 + KPC2 (1)	NS
Afolayan 2023 [34]	Greece	tertiary hospital in Athens	211 CRK	2003 and 2018	diverse	13	6.2 %	-	-	VIM-19 (13)	0
Baraniak 2015 [35]	Europe and Israel	multicentre project	Colonization with 110 KPC-Kp	2008-2011	fecal swabs	3	2.7 %		-	KPC2 (3)	NS
Wang 2021 [36]	China	multicentre resistance monitoring project	34 selected OX48-Kpn isolates	2013–2018	diverse	10	29.4% among OXA48-Kpn	-	-	OXA48 (10)	6 (60 %)
Guo 2016 [37]	China	respiratory ICU in Beijing	37 OXA48 Kpn isolates	2013 and 2014	diverse	27	-	-	**YES**	OXA48 (10)	NS

Palmieri 2019 [38]	China	4000-bed Hospital in Beijing	200 Kpn isolates	2002–2016	diverse	16	-	8%	**YES**	OXA48	8 (50 %)
Gan 2022 [39]	China	9 provinces of China	232 Kpn isolates	2013 to 2020	liver abscess and pneumonia	8	-	3.5%	-	OXA48 (6)	8 (100 %)
Potron 2013 [40]	France	NP	one patient who had been hospitalized in Tunis	2013	urine	1	-	-	-	OXA204 (1)	NS
Österblad 2012 [41]	Finland	National Institute for Health and Welfare	All CPE send to the center (26 Kpn isolates)	2008-11	diverse	1	-	-	-	VIM (1)	NS
Samuelsen 2011 [42]	Scandinavia	Scandinavia	8 VIM-Kpn isolates	2005-2008	diverse	1	-	-	-	VIM1 (1)	NS

NP: not specified, Kpn: *K. pneumoniae*

Table S11: (Supplementary Data 2, p3): Sequence records of the NCBI *Klebsiella pneumoniae* isolates used in the study.

Supplementary Data 1A–D contain the phylogenetic trees (1–43) generated in this study.

Supplementary References

1. Rotondo, C. *et al.* Molecular Characterization of Multidrug-Resistant and Hypervirulent New Delhi Metallo-Beta-Lactamase Klebsiella pneumoniae in Lazio, Italy: A Five-Year Retrospective Study. *Antibiotics* **13**, 1045 (2024).
2. Padovani, M. *et al.* In Vitro Activity of Cefiderocol on Multiresistant Bacterial Strains and Genomic Analysis of Two Cefiderocol Resistant Strains. *Antibiotics* **12**, 785 (2023).
3. Spaziante, M. *et al.* Importance of Surveillance of New Delhi Metallo-Beta-Lactamase Klebsiella pneumoniae: Molecular Characterization and Clonality of Strains Isolated in the Lazio Region, Italy. *Infect Drug Resist* **14**, 3659–3665 (2021).
4. Lorenzin, G. *et al.* Detection of NDM-1/5 and OXA-48 co-producing extensively drug-resistant hypervirulent Klebsiella pneumoniae in Northern Italy. *J Glob Antimicrob Resist* **28**, 146–150 (2022).
5. Ventura, A., Addis, E., Bertoncelli, A. & Mazzariol, A. Multiple detection of hypermucoviscous and hypervirulent strains of Klebsiella pneumoniae: An emergent health care threat. https://doi.org/10.1556/030.2022.01908 (2022) doi:10.1556/030.2022.01908.
6. Turton, J. *et al.* Hybrid Resistance and Virulence Plasmids in ‘High-Risk’ Clones of Klebsiella pneumoniae, Including Those Carrying blaNDM-5. *Microorganisms* **7**, 326 (2019).
7. Hammad, H. A., Abdelwahab, R., Browning, D. F. & Aly, S. A. Genome Characterization of Carbapenem-Resistant Hypervirulent Klebsiella pneumoniae Strains, Carrying Hybrid Resistance-Virulence IncHI1B/FIB Plasmids, Isolated from an Egyptian Pediatric ICU. *Microorganisms* **13**, 1058 (2025).
8. Attalla, E. T., Khalil, A. M., Zakaria, A. S., Baker, D. J. & Mohamed, N. M. Genomic characterization of colistin-resistant Klebsiella pneumoniae isolated from intensive care unit patients in Egypt. *Ann Clin Microbiol Antimicrob* **22**, 82 (2023).
9. Attalla, E. T. *et al.* Efficacy of colistin-based combinations against pandrug-resistant whole-genome-sequenced Klebsiella pneumoniae isolated from hospitalized patients in Egypt: an in vitro/vivo comparative study. *Gut Pathog* **16**, 73 (2024).

10. Abdelsalam, N. A. *et al.* Genomic dynamics of high-risk carbapenem-resistant Klebsiella pneumoniae clones carrying hypervirulence determinants in Egyptian clinical settings. *BMC Infectious Diseases* **24**, 1193 (2024).
11. Gamaleldin, P., Alseqely, M., Evans, B. A., Omar, H. & Abouelfetouh, A. Comparison of genotypic features between two groups of antibiotic resistant Klebsiella pneumoniae clinical isolates obtained before and after the COVID-19 pandemic from Egypt. *BMC Genomics* **25**, 983 (2024).
12. Edward, E. A., Mohamed, N. M. & Zakaria, A. S. Whole Genome Characterization of the High-Risk Clone ST383 Klebsiella pneumoniae with a Simultaneous Carriage of blaCTX-M-14 on IncL/M Plasmid and blaCTX-M-15 on Convergent IncHI1B/IncFIB Plasmid from Egypt. *Microorganisms* **10**, 1097 (2022).
13. Ahmed, M. A. E.-G. E.-S. *et al.* Emergence of Hypervirulent Carbapenem-Resistant Klebsiella pneumoniae Coharboring a blaNDM-1-Carrying Virulent Plasmid and a blaKPC-2-Carrying Plasmid in an Egyptian Hospital. *mSphere* **6**, 10.1128/msphere.00088-21 (2021).
14. Osman, E. A. *et al.* Klebsiella pneumonia in Sudan: Multidrug Resistance, Polyclonal Dissemination, and Virulence. *Antibiotics* **12**, 233 (2023).
15. Sobh, G. *et al.* Molecular characterization of carbapenem and ceftazidime-avibactam-resistant Enterobacterales and horizontal spread of bla NDM-5 gene at a Lebanese medical center. *Front Cell Infect Microbiol* **14**, 1407246 (2024).
16. Nawfal Dagher, T., Azar, E., Al-Bayssari, C., Chamieh, A. S. & Rolain, J.-M. First Detection of Colistin-Resistant Klebsiella pneumoniae in Association with NDM-5 Carbapenemase Isolated from Clinical Lebanese Patients. *Microbial Drug Resistance* **25**, 925–930 (2019).
17. Elgriw, N. *et al.* Clonal, Plasmidic and Genetic Diversity of Multi-Drug-Resistant Enterobacterales from Hospitalized Patients in Tripoli, Libya. *Antibiotics* **12**, 1430 (2023).
18. Eltai, N. O. *et al.* Identification of mcr-8 in Clinical Isolates From Qatar and Evaluation of Their Antimicrobial Profiles. *Front. Microbiol.* **11**, (2020).
19. Tsui, C. K.-M. *et al.* Genomic Epidemiology of Carbapenem-Resistant Klebsiella in Qatar: Emergence and Dissemination of Hypervirulent Klebsiella pneumoniae Sequence Type 383 Strains. *Antimicrobial Agents and Chemotherapy* **67**, e00030-23 (2023).

20. Abid, F. B. *et al.* Molecular characterization of clinical carbapenem-resistant Enterobacterales from Qatar. *Eur J Clin Microbiol Infect Dis* **40**, 1779–1785 (2021).

21. Sid Ahmed, M. A. *et al.* Phenotypic and Genotypic Characterization of Pan-Drug-Resistant Klebsiella pneumoniae Isolated in Qatar. *Antibiotics* **13**, 275 (2024).

22. Huang, J. *et al.* The dissemination of multidrug-resistant and hypervirulent Klebsiella pneumoniae clones across the Kingdom of Saudi Arabia. *Emerg Microbes Infect* **13**, 2427793.

23. Al-Zahrani, I. A. *et al.* Genomic analysis of extensively drug resistant (XDR) *Klebsiella pneumoniae* high-risk clone ST14 co-harboring *bla*NDM and *bla*OXA-48 recovered from Saudi Arabia. *Journal of Infection and Public Health* **17**, 669–675 (2024).

24. Alghoribi, M. F. *et al.* Genomic analysis of the first KPC-producing *Klebsiella pneumoniae* isolated from a patient in Riyadh: A new public health concern in Saudi Arabia. *Journal of Infection and Public Health* **13**, 647–650 (2020).

25. Chiarelli, A. *et al.* Diversity of mucoid to non-mucoid switch among carbapenemase-producing Klebsiella pneumoniae. *BMC Microbiology* **20**, 325 (2020).

26. Bonnin, R. A. *et al.* Emergence of New Non–Clonal Group 258 High-Risk Clones among Klebsiella pneumoniae Carbapenemase–Producing K. pneumoniae Isolates, France. *Emerg Infect Dis* **26**, 1212–1220 (2020).

27. Sabirova, J. S. *et al.* Whole-genome typing and characterization of blaVIM19-harbouring ST383 Klebsiella pneumoniae by PFGE, whole-genome mapping and WGS. *Journal of Antimicrobial Chemotherapy* **71**, 1501–1509 (2016).

28. Mavroidi, A. *et al.* Characterization of ST258 Colistin-Resistant, blaKPC-Producing Klebsiella pneumoniae in a Greek Hospital. *Microbial Drug Resistance* **22**, 392–398 (2016).

29. Papagiannitsis, C. C. *et al.* Characterisation of IncA/C2 plasmids carrying an In*416*-like integron with the *bla*VIM-19 gene from *Klebsiella pneumoniae* ST383 of Greek origin. *International Journal of Antimicrobial Agents* **47**, 158–162 (2016).

30. Pitt, M. E. *et al.* Multifactorial chromosomal variants regulate polymyxin resistance in extensively drug-resistant Klebsiella pneumoniae. *Microbial Genomics* **4**, e000158 (2018).

31. Xanthopoulou, K. *et al.* Surveillance and Genomic Analysis of Third-Generation Cephalosporin-Resistant and Carbapenem-Resistant Klebsiella pneumoniae Complex in Germany. *Antibiotics* **11**, 1286 (2022).
32. Giakkoupi, P. *et al.* An update of the evolving epidemic of blaKPC-2-carrying Klebsiella pneumoniae in Greece (2009–10). *Journal of Antimicrobial Chemotherapy* **66**, 1510–1513 (2011).
33. Papagiannitsis, C. C. *et al.* Emergence of *Klebsiella pneumoniae* of a novel sequence type (ST383) producing VIM-4, KPC-2 and CMY-4 β-lactamases. *International Journal of Antimicrobial Agents* **36**, 573–574 (2010).
34. Afolayan, A. O. *et al.* Three Klebsiella pneumoniae lineages causing bloodstream infections variably dominated within a Greek hospital over a 15 year period. *Microbial Genomics* **9**, 001082 (2023).
35. Baraniak, A. *et al.* KPC-Like Carbapenemase-Producing Enterobacteriaceae Colonizing Patients in Europe and Israel. *Antimicrobial Agents and Chemotherapy* **60**, 1912–1917 (2016).
36. Wang, L., Guo, L., Ye, K. & Yang, J. Genetic characteristics of OXA-48-producing Enterobacterales from China. *Journal of Global Antimicrobial Resistance* **26**, 285–291 (2021).
37. Guo, L. *et al.* Nosocomial Outbreak of OXA-48-Producing Klebsiella pneumoniae in a Chinese Hospital: Clonal Transmission of ST147 and ST383. *PLOS ONE* **11**, e0160754 (2016).
38. Palmieri, M. *et al.* Genomic evolution and local epidemiology of Klebsiella pneumoniae from a major hospital in Beijing, China, over a 15 year period: dissemination of known and novel high-risk clones. *Microb Genom* **7**, 000520 (2021).
39. Gan, L. *et al.* Genetic Diversity and Pathogenic Features in Klebsiella pneumoniae Isolates from Patients with Pyogenic Liver Abscess and Pneumonia. *Microbiology Spectrum* **10**, e02646-21 (2022).
40. Potron, A., Nordmann, P. & Poirel, L. Characterization of OXA-204, a Carbapenem-Hydrolyzing Class D β-Lactamase from Klebsiella pneumoniae. *Antimicrob Agents Chemother* **57**, 633–636 (2013).
41. Österblad, M. *et al.* Carbapenemase-producing Enterobacteriaceae in Finland: the first years (2008–11). *Journal of Antimicrobial Chemotherapy* **67**, 2860–2864 (2012).
42. Samuelsen, Ø. *et al.* Molecular characterization of VIM-producing Klebsiella pneumoniae from Scandinavia reveals genetic relatedness with international clonal complexes encoding transferable multidrug resistance. *Clinical Microbiology and Infection* **17**, 1811–1816 (2011).
